# Causal Effects of Lifestyle and Dietary Factors on Rheumatoid Arthritis: An Integrated Analysis Combining Mendelian Randomization, Machine Learning, and Evaluation of Burden Dynamics and Health Inequality

**DOI:** 10.1002/fsn3.71584

**Published:** 2026-02-22

**Authors:** Yan Gao, Guangxin Gu, Ruiwen Wang, Wenfeng Han, Bin Zheng, Aoxiang Yang, Ning Wang, Hailong Yu, Chen Jia, Yu Wang

**Affiliations:** ^1^ Department of Disease Prevention and Control General Hospital of Northern Theater Command Shenyang China; ^2^ Key Laboratory of Environmental Stress and Chronic Disease Control & Prevention China Medical University, Ministry of Education Shenyang China; ^3^ Department of Epidemiology, School of Public Health China Medical University Shenyang China; ^4^ Department of Orthopedics General Hospital of Northern Theater Command Shenyang China

**Keywords:** dietary factors, global burden of disease study, lifestyle, machine learning, mendelian randomization, rheumatoid arthritis

## Abstract

Rheumatoid arthritis (RA) is a chronic autoimmune disease contributing to global morbidity and disability. Despite its growing burden, causal links between lifestyle, dietary factors, and RA remain unclear. This study investigates causal links between lifestyle, dietary factors, and RA using Mendelian randomization (MR) and machine learning (ML). Two‐sample MR analyzed 42 lifestyle and dietary exposures, while an RA risk prediction model was developed using nationally representative data and nine ML algorithms. Global Burden of Disease data assessed health inequalities and RA burden trends through frontier, decomposition, and Bayesian Age‐Period‐Cohort analyses. MR identified obesity, current smoking, sleeplessness, poultry intake, and salt added to food as RA risk factors, while never smoking, pork consumption, and cheese intake were protective. Random forest showed superior predictive performance, with age as the most influential predictor; seven features exhibited nonlinear RA risk associations. RA burden revealed gender and regional disparities, with frontier analysis indicating potential for burden reduction in multiple countries. Between 1990 and 2021, global RA burden rose due to population growth and aging, with projections suggesting continued increases through 2050. These findings highlight the importance of targeted lifestyle and dietary interventions to reduce RA burden and address health inequities in high‐risk populations.

## Graphical abstract

Using two‐sample Mendelian randomization, higher BMI, obesity class 2, current smoking, sleeplessness, poultry intake, and salt added to food were causally associated with increased rheumatoid arthritis (RA) risk, while never smoking, pork intake, and cheese intake showed protective associations. A machine‐learning framework identified seven key predictors and achieved robust discrimination (random forest AUROC = 0.908) with good calibration and net benefit. Global burden analyses revealed increasing RA prevalence and DALYs from 1990 to 2021 with persistent health inequalities, and projections suggest continued growth through 2050.

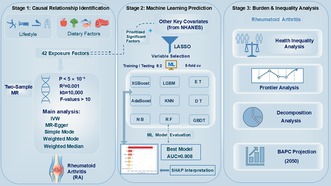

**Abbreviations**
ASDR: age‐standardized DALYs rates
ASPR: age‐standardized prevalence rates
ASR: age‐standardized rates
AUROC: area under the receiver operating characteristic curve
BAPC: Bayesian Age‐Period‐Cohort
BMI: body mass index
CI: concentration index
DALYs: Disability‐Adjusted Life Years
DT: Decision Tree
EAPC: estimated annual percentage change
ET: Extremely Randomized Trees
GBD: Global Burden of Disease
GBDT: Gradient Boosting Decision Tree
GWAS: Genome‐Wide Association Study
IVs: instrumental variables
IVW: inverse‐variance weighted
KNN: k‐Nearest Neighbors
LASSO: Least Absolute Shrinkage and Selection Operator
LD: linkage disequilibrium
LGBM: LightGBM
ML: machine learning
MR: Mendelian randomization
NB: Naïve Bayes
NHANES: National Health and Nutrition Examination Survey
PIR: poverty income ratio
RA: Rheumatoid arthritis
RF: Random Forest
SDI: Sociodemographic Index
SHAP: Shapley Additive Explanations
SII: slope inequality index
SNP: Single Nucleotide Polymorphism
UI: uncertainty interval

## 1 Introduction

Rheumatoid arthritis (RA) is a chronic autoimmune disease, with one of its key clinical manifestations being symmetrical polyarthritis, characterized by joint pain, swelling, and stiffness (Wu et al. 2025; Zhang et al. 2024). The global prevalence of RA ranges from 0.24% to 1% (Almoallim et al. 2021). As the disease progresses and with increasing age, the chronic pain and joint deformities caused by RA have a growing impact on quality of life and productivity, representing an increasingly serious public health challenge (Zhao et al. 2024). Although the exact etiology of RA remains unclear, substantial evidence indicates that lifestyle factors (such as smoking, obesity, and sleep disorders) and dietary habits (including high salt intake and micronutrient consumption) are crucial modifiable factors influencing both the risk of developing RA and its progression (Corrado et al. 2025; George et al. 2025; Zhang et al. 2025). Interventions targeting single lifestyle factors, such as Mediterranean or plant‐based diets and regular physical activity, have shown positive effects on RA management (Papandreou et al. 2023; Hurkmans et al. 2009). Despite the important associations identified between lifestyle and dietary factors and RA, most of the studies rely on observational designs (Ro et al. 2022; Hahn et al. 2023; Ni et al. 2024; Fang et al. 2023; Kronzer et al. 2022; He et al. 2016), which makes it challenging to draw definitive causal inferences, thus limiting the reliability of the findings.

Mendelian randomization (MR) analysis, as a powerful complement to randomized controlled trials, has been widely used in epidemiological research to assess the causal effects of exposures on specific outcomes through genetic variables (Skrivankova et al. 2021). Compared to observational studies, MR reduces confounding bias through the random allocation of genetic variants, thereby helping to further clarify the causal relationship between RA and lifestyle and dietary factors. Building on this, the integration of high‐quality, large‐scale, nationally representative data from the National Health and Nutrition Examination Survey (NHANES) to develop multiple machine learning (ML) predictive models holds great promise in enhancing the early identification and personalized intervention of RA.

Clarifying the causal relationship between lifestyle and dietary factors and RA, as well as exploring the burden differences at the population level, carries significant public health implications. Despite notable advances in global medical technology in recent years, which have reduced the disability rates caused by RA, the overall burden of the disease remains substantial due to unequal distribution of healthcare resources and insufficient public health awareness (Safiri et al. 2019). The 2021 Global Burden of Disease (GBD) study provides key indicators such as incidence, prevalence, and DALYs (Yan et al. 2025), serving as an important basis for assessing the burden differences of RA across different regions and populations, as well as the phenomenon of health inequalities. Based on this, the present study innovatively integrates MR methods, NHANES data, and the GBD database to systematically evaluate the causal impact of lifestyle and dietary factors on RA incidence. It also analyzes the disease burden characteristics at the global level, offering theoretical and data‐driven support for early intervention, precision prevention, and policy formulation for RA.

## 2 Methods

### 2.1 Study Design

**FIGURE 1:**
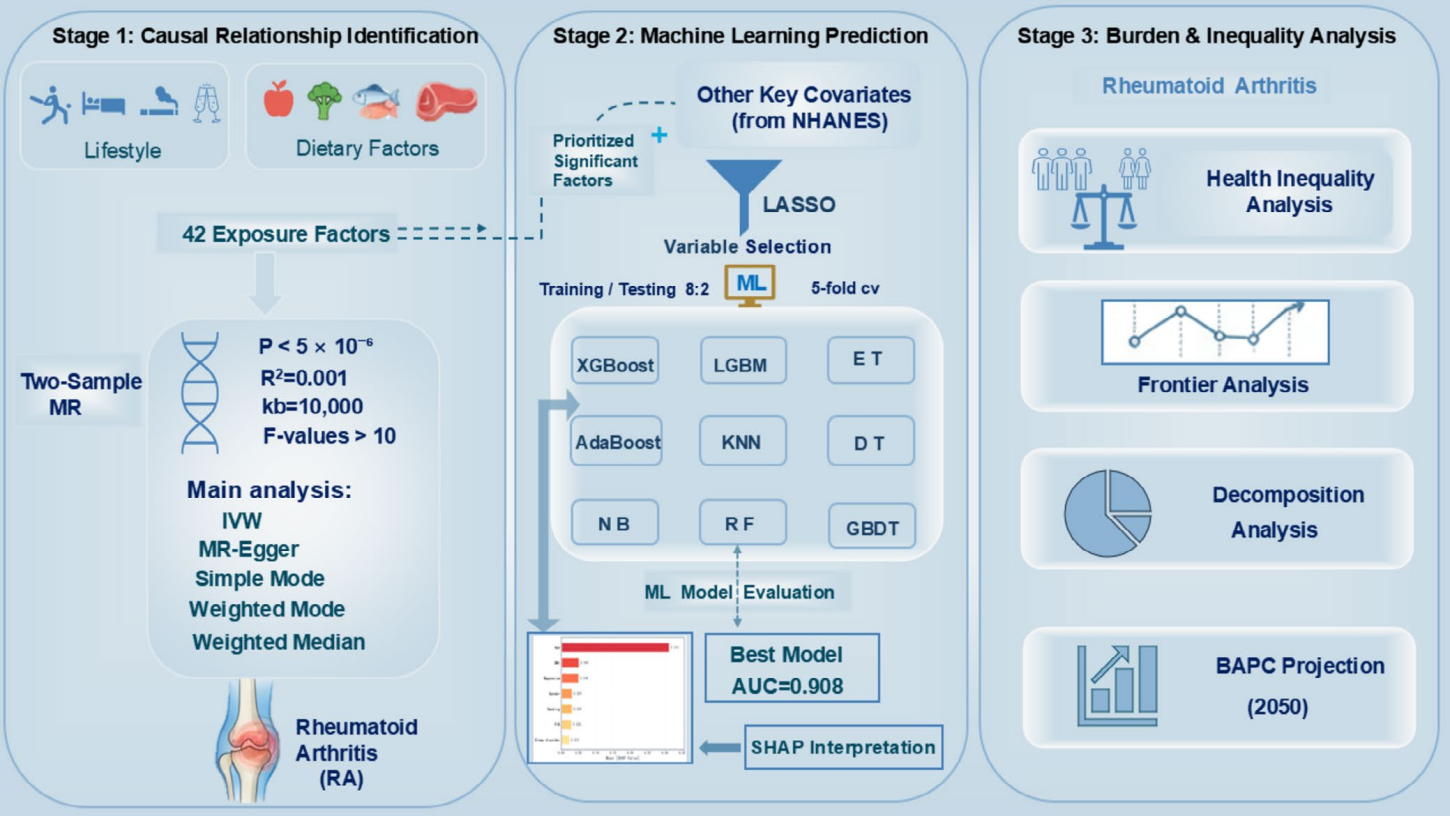
A three‐stage analytical framework for investigating the causes, prediction, and burden of rheumatoid arthritis.

This study constructs a three‐stage analytical framework that integrates multisource data to systematically evaluate the potential causal relationships between lifestyle and dietary factors and RA, while exploring trends in disease burden and health inequality (Figure 1). First, a univariate two‐sample MR approach is employed to identify the causal relationships between 42 exposure factors and RA. The lifestyle factors include body mass index (BMI), different levels of obesity, smoking, alcohol and coffee consumption, salt added to food, physical activity, sedentary behavior, and sleeplessness. The dietary factors encompass the intake of red meat, fish, vegetables, fruits, dairy products, as well as the intake of micronutrients such as copper, magnesium, potassium, folate, vitamins A, B6, B12, C, D, and E. Second, factors significantly associated with RA are prioritized for inclusion in the NHANES prediction model. Key variables are selected using Least Absolute Shrinkage and Selection Operator (LASSO) regression, and the performance of 9 ML models is constructed and evaluated. The importance of the features is interpreted using the Shapley Additive ExPlanations (SHAP) method. Third, an evaluation of health inequality is introduced, along with frontier analysis, decomposition analysis, and Bayesian age‐period‐cohort (BAPC) modeling. These methods are used to dynamically assess the disease burden of RA, identify health inequality characteristics and their driving factors across different populations, and predict trends in burden changes and potential improvements over the next 30 years.

### 2.2 Data Sources and Statistical Analysis

#### 2.2.1 Mendelian Randomization Analysis

The lifestyle and dietary factors, as well as the genetic instrumental variables (IVs) for RA required in this study, were obtained from the publicly available IEU Open Genome‐Wide Association Study (GWAS) database (https://gwas.mrcieu.ac.uk/) (Buniello et al. 2019). For such publicly available aggregated data, no additional ethical review is necessary. Detailed information on the specific variables is provided in Table S1.

We first integrated the candidate genetic instrumental variables from each GWAS and retained only those Single Nucleotide Polymorphisms (SNPs) that satisfied the three core assumptions of MR as IVs. The specific selection criteria included: candidate SNPs required to meet the genome‐wide significance level (*p* < 5 × 10^−6^), linkage disequilibrium (LD) threshold set at *r* ^2^ < 0.001, and clumping distance of 10,000 kb (Zou et al. 2021). Simultaneously, SNPs with *F*‐values < 10 were considered weak instrumental variables and were excluded (Pierce et al. 2011).

The primary analysis used inverse‐variance weighted (IVW) method for MR inference. Additionally, MR‐Egger regression, weighted median method, simple mode method, and weighted mode method were employed as supplementary analyses (Xu et al. 2022). To assess the robustness of the results, sensitivity analyses were conducted, including: Cochran's *Q* test to evaluate heterogeneity, MR‐Egger regression intercept test to assess directional pleiotropy (Qian et al. 2020), and leave‐one‐out analysis to identify individual SNPs that might drive bias (Zheng et al. 2017).

#### 2.2.2 Machine Learning Strategy

This study was based on data from four cycles (2007–2014) of NHANES, including a total of 8446 participants (RA: 823; non‐RA: 7623), encompassing baseline characteristics, lifestyle factors, and dietary variables. Variable selection was performed using LASSO regression, and the seven selected variables were subsequently incorporated into model development (Tibshirani 1996).

Given the substantial class imbalance in the original dataset (non‐case to case ratio of 9.26:1), a class weight adjustment strategy was applied, assigning higher weights to the minority class during model training to mitigate bias. The dataset was stratified and split into training and testing sets at an 8:2 ratio. We evaluated nine ML algorithms: Random Forest (RF), Gradient Boosting Decision Tree (GBDT), XGBoost, LightGBM (LGBM), Extremely Randomized Trees (ET), AdaBoost, k‐Nearest Neighbors (KNN), Decision Tree (DT), and Naïve Bayes (NB). All models underwent hyperparameter tuning and five‐fold cross‐validation, with the primary evaluation metric being the area under the receiver operating characteristic curve (AUROC). To enhance model interpretability, we employed SHAP to quantify the contribution of each feature to the predictive outcomes, providing both local and global explanations (Tibshirani 1996; Wolters 2023). Details of the ML methodology are provided in the Methods in Appendix S1.

#### 2.2.3 Global Trends and Health Inequality in RA Burden

This study extracted epidemiological indicators such as the prevalence and Disability‐Adjusted Life Years (DALYs) of RA from the GBD study results tool (https://vizhub.healthdata.org/gbd‐results/) (Naghavi et al. 2024). A 95% uncertainty interval (UI) was employed to reflect the reliability range of the estimates. Temporal trends in the age‐standardized prevalence (ASPR) and DALYs (ASDR) rates of RA from 1990 to 2021 were analyzed by calculating the estimated annual percentage change (EAPCs) of the age‐standardized rates (ASR) (Ding et al. 2022).

In the socioeconomic dimension, this study employed the Sociodemographic Index (SDI) to assess disparities in the burden of RA across countries and regions at different levels of development (Cousin et al. 2022). Based on health inequality analyses, the slope inequality index (SII) and concentration index (CI) were calculated to quantify the extent of inequitable distribution of disease burden among countries with varying degrees of social development (World Health Organization 2013; Xie et al. 2018). Furthermore, frontier analysis was applied to identify benchmark countries that achieved the lowest disease burden at each SDI level, thereby establishing best practice reference points for RA prevention and control (Gupta 1994). In addition, decomposition analysis was conducted to partition changes in RA burden into three contributing effects: population growth, population aging, and epidemiological changes, with the aim of elucidating the drivers of temporal trends (Riebler and Held 2017). Finally, a BAPC model was used to project epidemiological patterns of RA in male and female populations worldwide, including the predicted number of cases and ASRs in trends through 2050 (Tu et al. 2012). The detailed methodology of the GBD analysis is described in the Supplementary Methods. All data were analyzed using R software version 4.4.2 and Python software version 3.9.13. A *p*‐value of < 0.05 was considered statistically significant.

## 3 Results

### 3.1 Causal Association Between Lifestyle and Dietary Factors and RA

Using a two‐sample MR approach, we analyzed the causal associations between 3674 SNPs related to lifestyle and dietary factors in relation to the risk of RA, encompassing 553 BMI, 14 obesity, 36 obesity class 1, 28 obesity class 2, 10 obesity class 3, 192 smoking initiation, 121 current tobacco smoking, 189 smoking status: never, 158 alcohol intake frequency, 92 average weekly beer plus cider intake, 77 average weekly red wine intake, 103 coffee intake, 121 tea intake, 81 moderate to vigorous physical activity levels, 19 active to sedentary transition probability, 136 sleeplessness, 11 bacon intake, 87 beef intake, 109 lamb mutton intake, 69 pork intake, 75 poultry intake, 106 processed meat intake, 141 oily fish intake, 61 non‐oily fish intake, 127 fresh fruit intake, 138 dried fruit intake, 83 cooked vegetable intake, 86 salad/raw vegetable intake, 19 milk intake, 171 cheese intake, 145 cereal intake, 203 salt added to food, 6 copper, 17 magnesium, 12 potassium, 13 folate, 13 vitamin A, 15 vitamin B6, 8 vitamin B12, 9 vitamin C, 9 vitamin D, and 11 vitamin E (Table S2).

**FIGURE 2:**
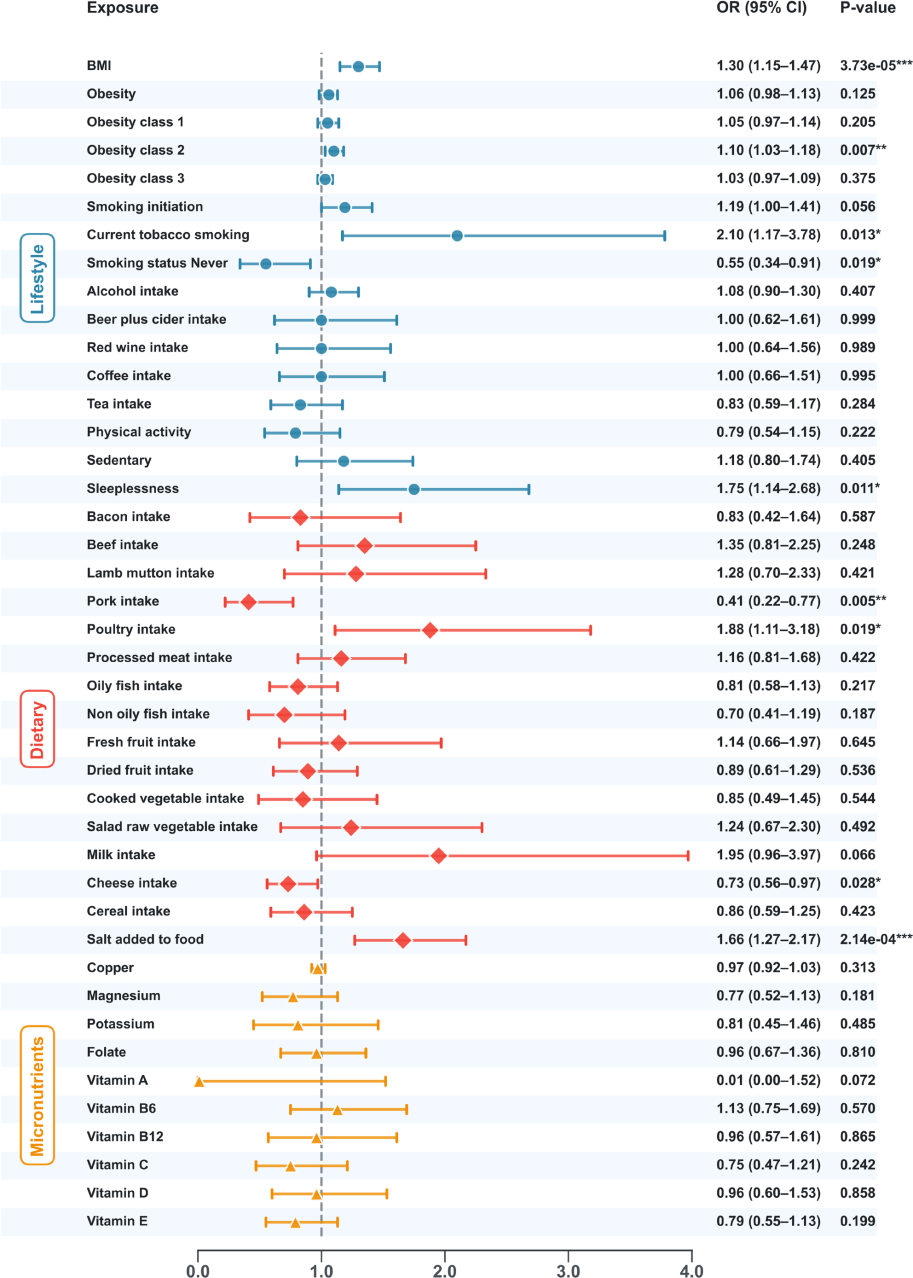
Causal effects of lifestyle and dietary factors on rheumatoid arthritis.

All SNPs included in this study had *F*‐statistics greater than 10, indicating the absence of weak instrumental variables (Table S2). The IVW method revealed significant positive causal associations between RA and the following exposures: BMI (OR = 1.299, 95% CI: 1.147–1.472, *p* = 3.73 × 10^−5^), obesity class 2 (OR = 1.100, 95% CI: 1.027–1.179, *p* = 0.007), current tobacco smoking (OR = 2.100, 95% CI: 1.166–3.781, *p* = 0.013), sleeplessness (OR = 1.747, 95% CI: 1.139–2.679, *p* = 0.011), poultry intake (OR = 1.881, 95% CI: 1.111–3.185, *p* = 0.019), and salt added to food (OR = 1.657, 95% CI: 1.268–2.615, *p* = 2.14 × 10^−4^). Conversely, never smoking (OR = 0.555, 95% CI: 0.338–0.909, *p* = 0.019), pork intake (OR = 0.413, 95% CI: 0.221–0.770, *p* = 0.005), and cheese intake (OR = 0.733, 95% CI: 0.555–0.967, *p* = 0.028) were significantly associated with a reduced risk of RA (Figure 2). However, these associations were not observed when using the other four MR methods. No evidence of heterogeneity or horizontal pleiotropy was detected in these analyses (Table S3).

### 3.2 Machine Learning‐Based RA Risk Prediction

A total of 8446 participants were enrolled in the study, comprising 823 individuals with RA and 7623 non‐RA controls; the participant selection process, study design, and detailed baseline characteristics are presented in the Appendix S1 (Table S4, Figure S1). Feature selection was conducted using the LASSO regression method, which ultimately identified seven key variables: age, sex, family poverty income ratio (PIR), BMI, smoking status, presence of sleep disorders, and dietary magnesium intake (Figure S2).

To address the pronounced class imbalance in our dataset, we benchmarked nine machine‐learning algorithms. Conventional classifiers, exemplified by support vector machines (SVM), showed limited generalizability, achieving an extremely low recall of 0.021. In contrast, tree‐based ensemble approaches exhibited markedly stronger discriminative performance, with the random forest model emerging as the optimal classifier. Although the Extra Trees model delivered higher sensitivity, this improvement came at the cost of reduced precision (0.457), indicating a substantial false‐positive burden. RF achieved the most favorable balance between sensitivity and specificity, yielding the highest F1 score (0.558) and Matthews correlation coefficient (MCC, 0.509), while maintaining a recall of 0.661 and a specificity of 0.923. Critically, to address concerns regarding overfitting, we compared cross‐validation (CV) and independent test‐set performance. The RF model demonstrated exceptional stability, with only a marginal difference between CV‐AUC (0.916) and test AUC (0.908; gap = 0.008), supporting that the observed high AUC reflects true predictive capability rather than overfitting. Consistent with these findings (Table S5), RF retained a relatively balanced precision–recall profile. While Extra Trees reached a sensitivity of 0.715, its lower precision suggests persistent inflation of false positives. Gradient‐boosting models, including XGBoost and LightGBM, achieved F1 scores of 0.535 and 0.520, respectively, indicating limited ability to identify a subset of RA individuals, whereas AdaBoost, Gradient Boosting, and decision tree models yielded F1 scores below 0.550, reflecting overall constrained classification performance.

To further assess clinical reliability, we evaluated model calibration and net benefit. The RF model exhibited acceptable calibration, with a Brier score of 0.079. As shown by the calibration curve (Figure S3), the model displayed a slight conservative tendency in risk estimation, while maintaining superior overall stability compared with other classifiers. For clinical implementation, the optimal classification threshold derived from the Youden index was 0.62, at which specificity reached 92.3%. However, given the low prevalence of RA in our cohort (approximately 10%), it is essential to evaluate clinical benefit across a range of risk thresholds rather than relying on a single operating point. Decision curve analysis (DCA; Figure S4) demonstrated that the RF model provided a positive net benefit across threshold probabilities of approximately 2%–40%, outperforming both “treat‐all” and “treat‐none” strategies within this clinically relevant window. Although net benefit attenuated at higher thresholds due to the low disease prevalence, the broad effectiveness in the low‐probability region (< 40%) highlights the model's potential utility as a rule‐out screening tool, enabling efficient identification of high‐risk individuals for referral while safely minimizing unnecessary interventions among low‐risk populations.

**FIGURE 3:**
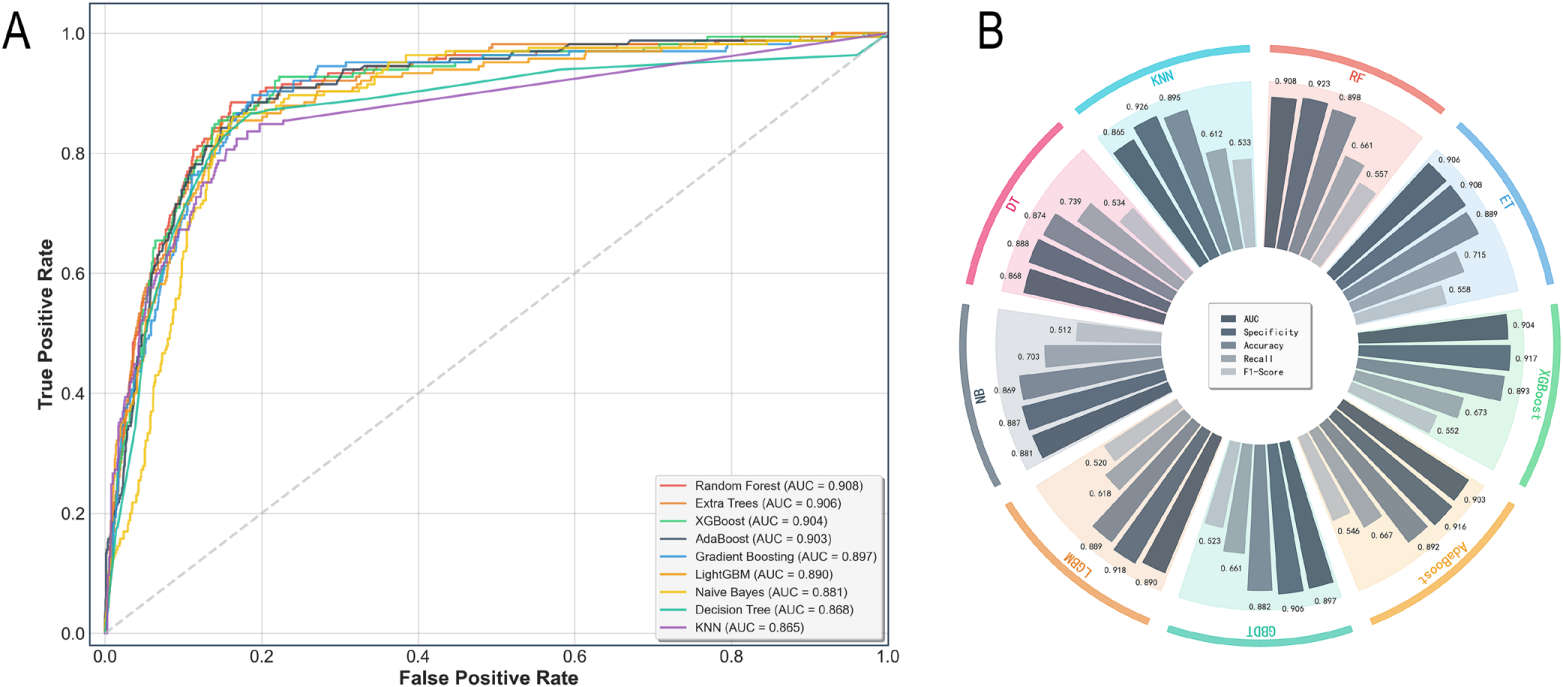
Performance comparison of nine machine learning models on the test set. (A) ROC Curve Plot: It illustrates the performance of the nine machine learning models across all classification thresholds. A curve closer to the top‐left corner indicates better model performance. The area under the curve (AUC) value, shown in the legend, is a key metric that quantifies the model's overall performance. (B) Radial Bar Chart: It visually compares the nine models across five specific performance metrics: AUC, Specificity, Accuracy, Recall, and F1‐Score. For each model, the five bars represent its scores on these respective indicators.

This study employed nine machine learning models (RF, GBDT, XGBoost, LGBM, ET, AdaBoost, KNN, DT, and NB) to perform predictions based on the same set of seven selected features. Performance evaluation showed that RF achieved superior results across multiple metrics, including an AUROC of 0.908, and was therefore selected as the optimal model for this study (Figure 3A). The performance of the nine models was comparatively evaluated on the test set using performance metrics and confusion matrices (Figure 3B; Figures S3–S7).

**FIGURE 4:**
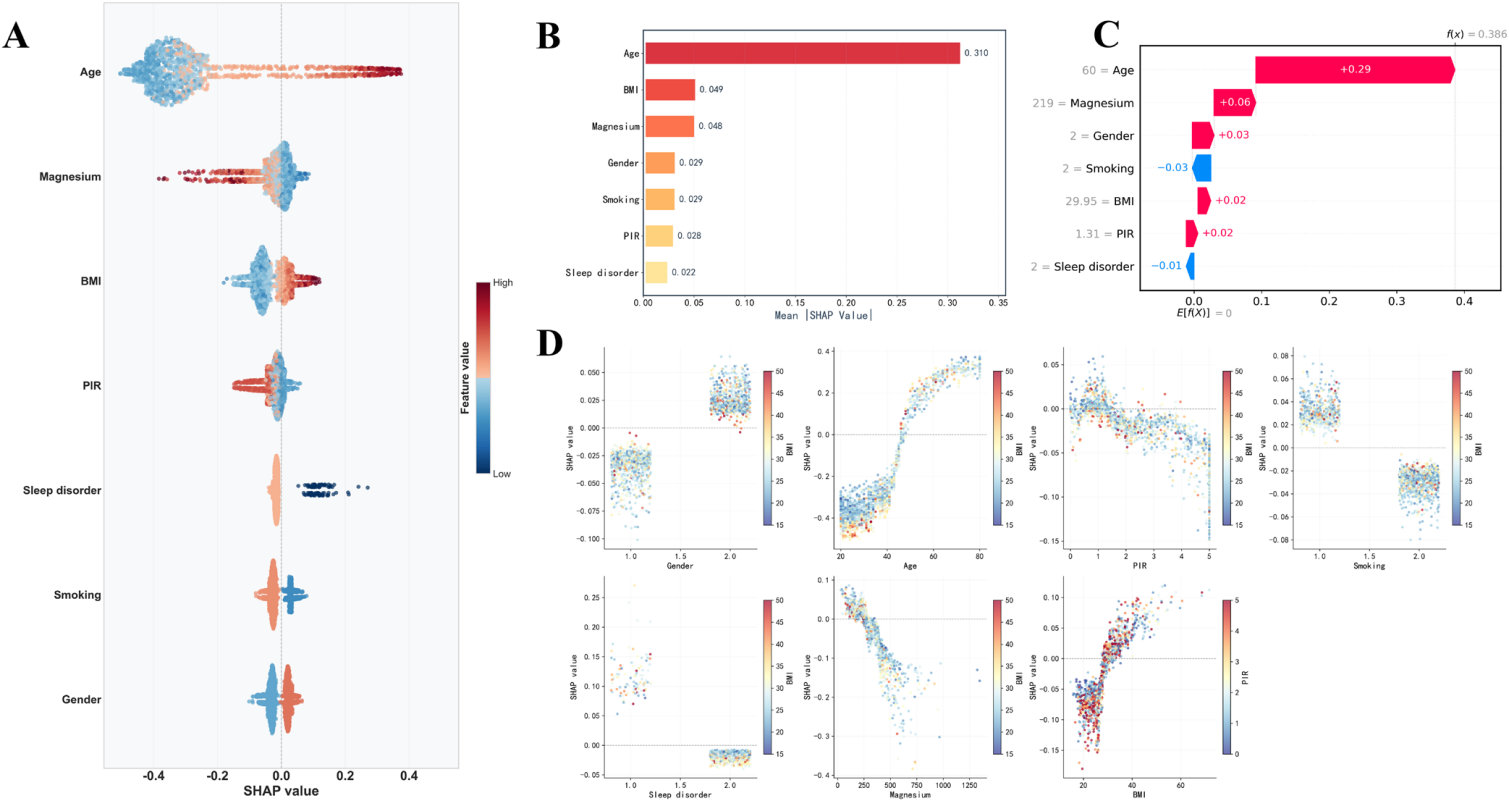
Evaluation of feature importance and contribution in the random forest model using SHAP. (A) SHAP summary plot: Shows the impact and direction of each feature on predictions, ranking them by importance. (B) Global feature importance plot: Ranks features by their average impact (mean absolute SHAP value). (C) SHAP waterfall plot: Explains a single prediction by showing how each feature value contributes to the final output score. (D) SHAP dependence plots: Shows how a feature's value affects its prediction impact, using color to reveal interaction effects with another feature.

We applied the SHAP method to interpret the optimal RF model, quantifying the contribution of each variable to RA prediction in the test dataset. The SHAP importance ranking plot indicated that age was the most influential factor in the prediction, with the largest positive impact. Female gender, insufficient magnesium intake, sleep disorders, abnormal BMI, smoking, and low PIR were associated with a lower risk of RA. Partial dependence plots further revealed the nonlinear relationships between these variables and the prediction outcomes, suggesting the potential for complex interactions among variables that may influence the accuracy of predictions in certain cases. The variation in the contribution of these features to RA prediction across individuals underscores the importance of personalized factors in disease prediction (Figure 4).

### 3.3 Global Trends and Health Inequality in RA Burden

**FIGURE 5:**
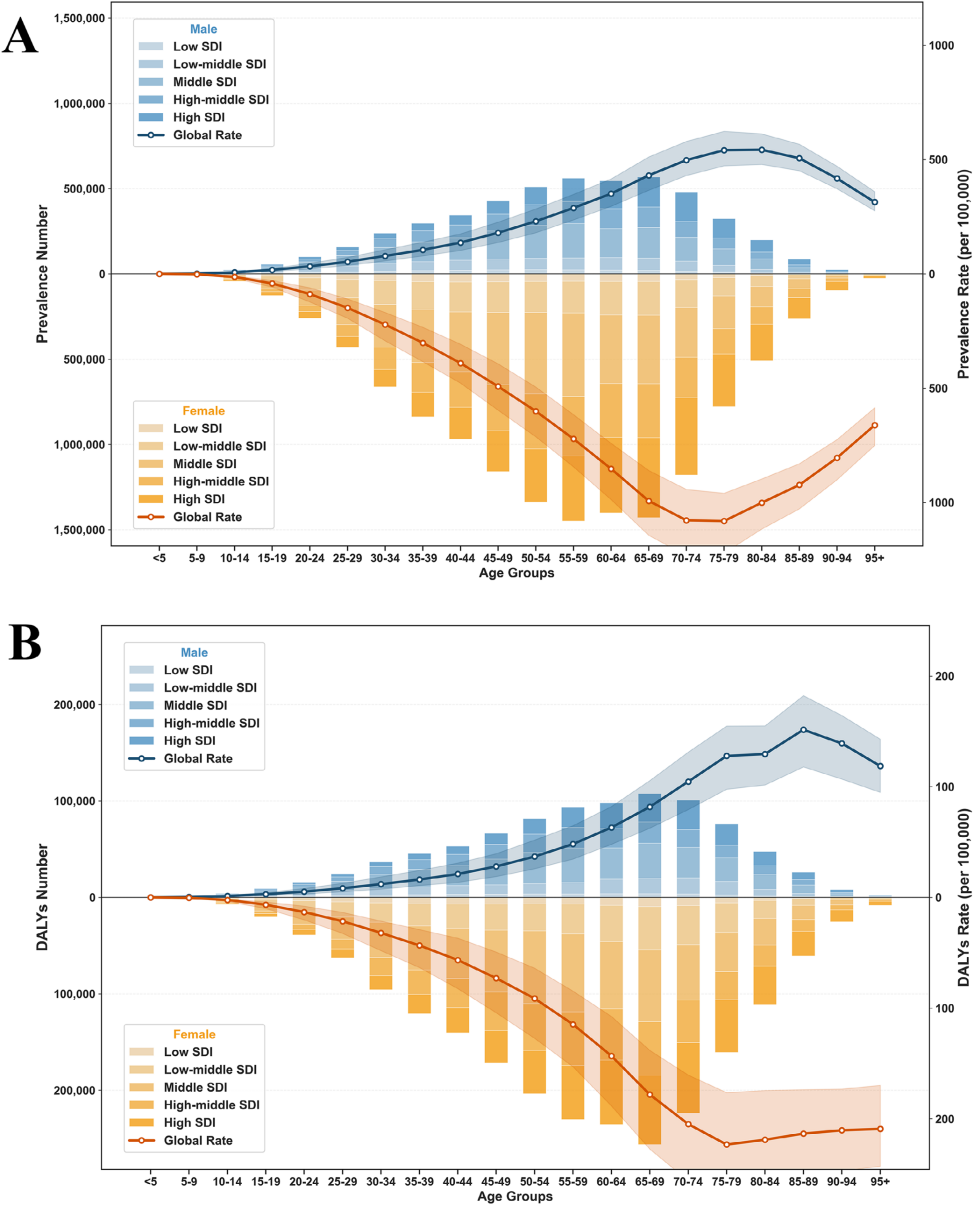
Global prevalence (A) and DALYs (B) of rheumatoid arthritis by age, gender, and SDI in 2021. DALYs, disability‐adjusted life years; SDI, socio‐demographic index.

**FIGURE 6:**
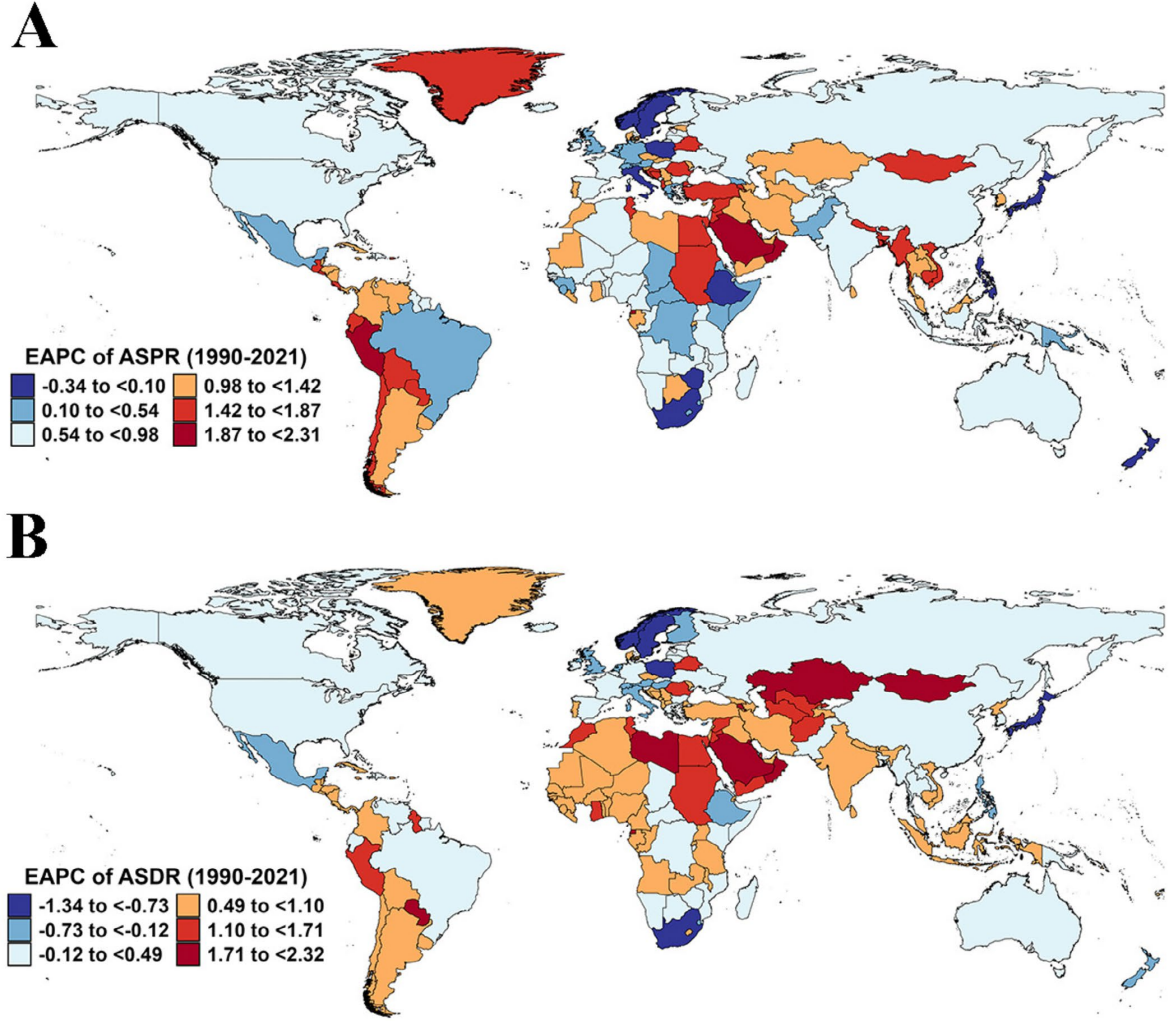
EAPC of ASRs for rheumatoid arthritis in 204 countries and territories (1990–2021). (A) EAPC of ASPR. (B) EAPC of ASDR. ASDR, age‐standardized disability‐adjusted life years rate; ASPR, age‐standardized prevalence rate; ASR, age‐standardized rate; EAPC, estimated annual percentage change.

In 2021, the global number of RA cases was 17.92 million (95% UI: 15.97, 20.30), and the number of DALYs was 3.08 million (95% UI: 2.31, 3.97). Between 1990 and 2021, both the ASPR and ASDR showed an increasing trend, with respective EAPCs of 0.53 (95% CI: 0.49, 0.57) and 0.05 (95% CI: 0.01, 0.10) (Tables S5 and S6). Age‐ and gender‐specific analysis revealed that in 2021, the highest number of RA cases and DALYs occurred in the 55–59 and 65–69 age groups, with females significantly higher than males (Figure 5). In 2021, the regions with the highest number of cases for both prevalence and DALYs were East Asia, while the region with the fastest increase in ASRs over the past 30 years was Andean Latin America (Tables S5 and S6). Among the 204 countries and territories, the highest number of cases for both prevalence and DALYs were in China, India, and the United States, while the countries with the fastest growth in ASPR and ASDR were Equatorial Guinea and Bahrain (Figure 6; Tables S7–S9).

**FIGURE 7:**
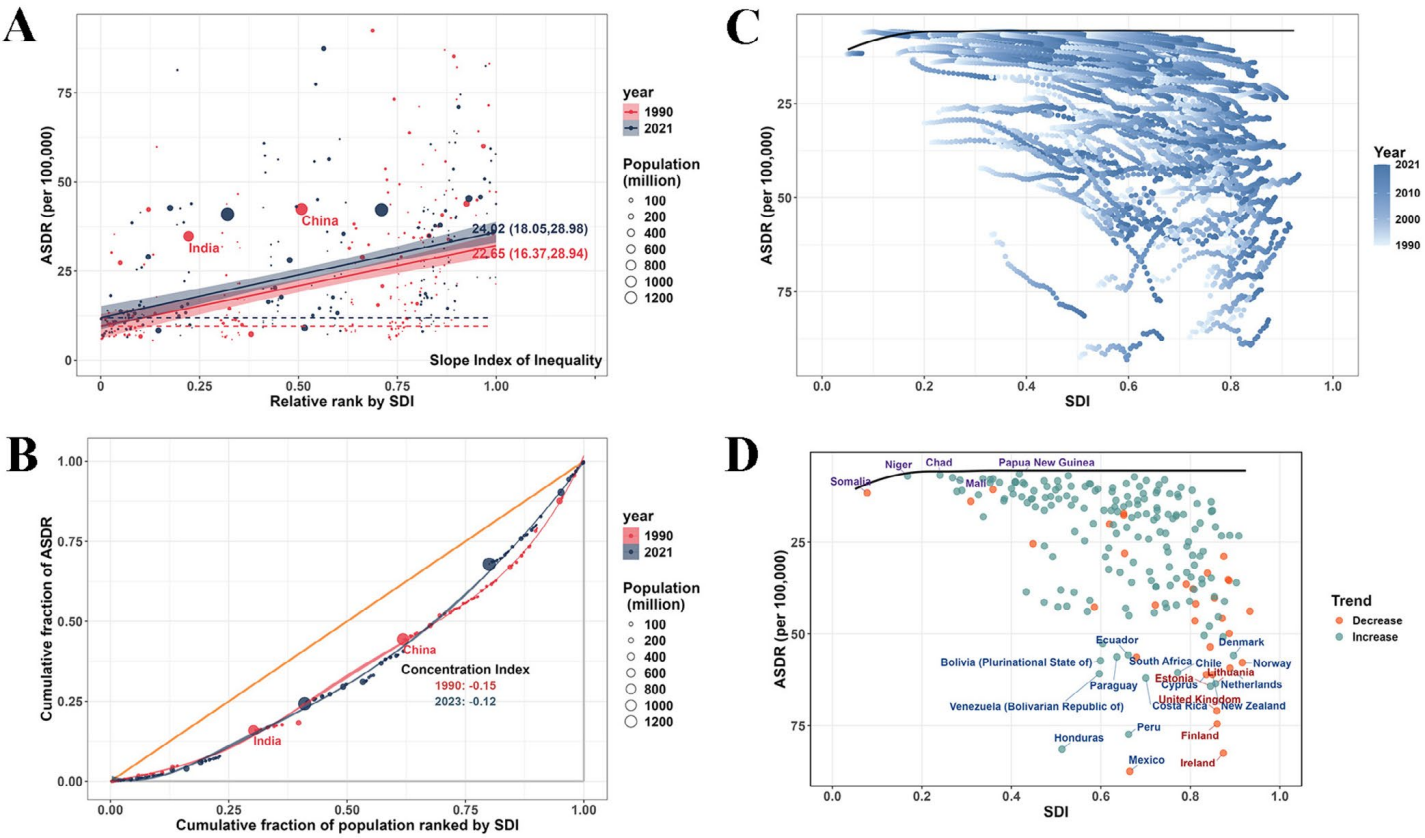
Health inequality and frontier analysis of rheumatoid arthritis in 204 countries and territories. (A, B) Health inequality. (C, D) Frontier analysis. ASDR, age‐standardized disability‐adjusted life years rate; SDI, socio‐demographic index.

**FIGURE 8:**
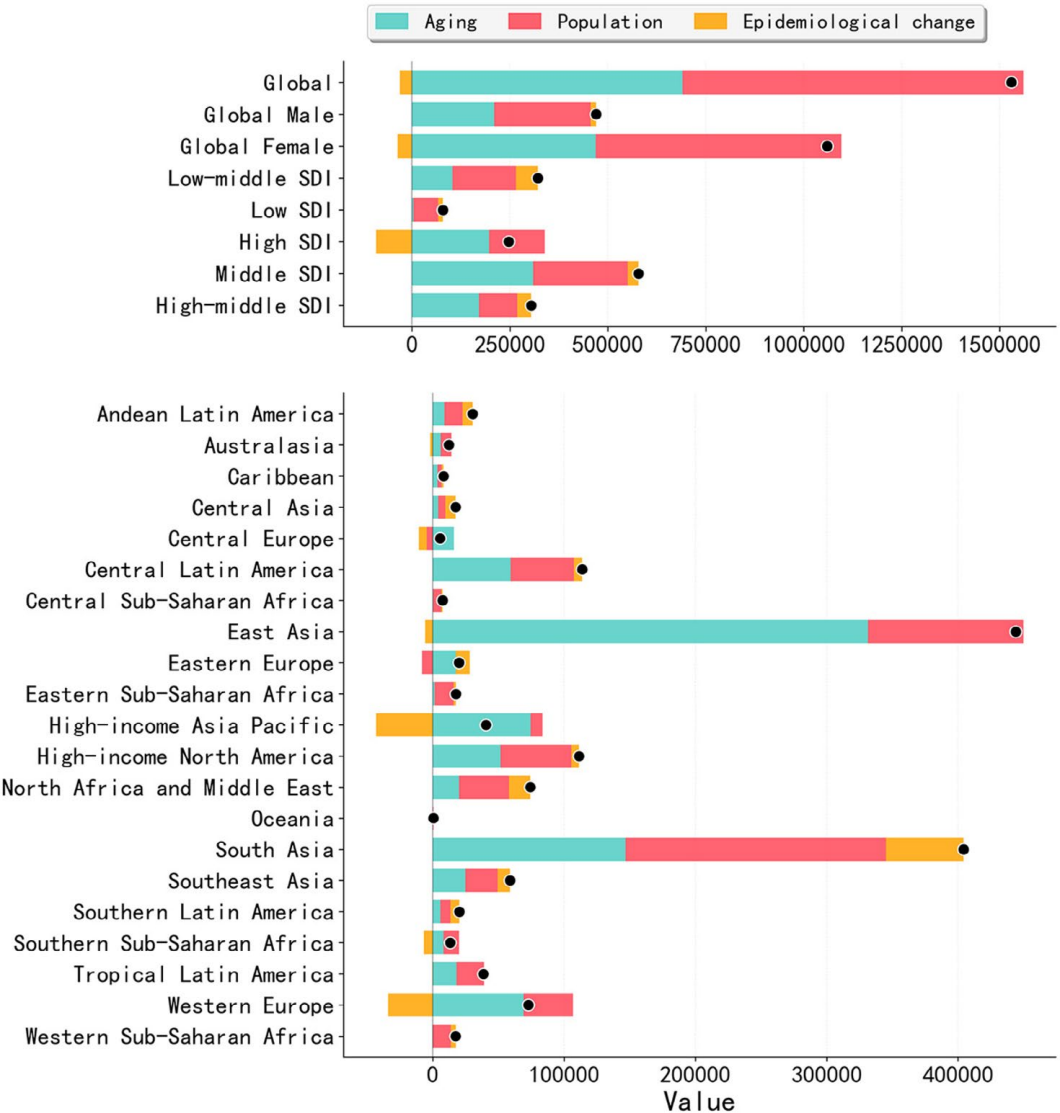
Decomposition of change in the number of DALYs due to rheumatoid arthritis by driver, 1990–2021. DLAY, disability‐adjusted life years; SDI, socio‐demographic index.

Health inequality analysis showed that the SII increased from 22.65 per 100,000 in 1990 to 24.02 per 100,000 in 2021. The CI was −0.15 in 1990 and −0.12 in 2021, indicating the presence of both absolute and relative inequalities in the RA burden across countries with different SDI levels (Figure 7A,B). Frontier analysis based on ASDR and SDI in 204 countries and territories revealed that, in 1990, many low‐SDI countries exhibited higher ASDR. Over time, particularly in higher‐SDI countries, ASDR declined substantially. High‐SDI countries such as Ireland showed a considerable gap from the frontier, while low‐SDI countries such as Somalia were closer to it. Although the RA burden declined over the past three decades in high‐SDI countries such as New Zealand and Norway, a substantial distance from the frontier persisted (Figure 7C,D; Table S10). Decomposition analysis indicated that the global increase in RA burden between 1990 and 2021 was mainly driven by population growth (56.89%) and population aging (45.13%), while epidemiological factors contributed −2.03%. The most notable increases in DALYs burden occurred in the Low‐middle SDI and East Asia regions (Figure 8). Projections based on the BAPC model suggest a continued increase in global RA prevalence and DALYs through 2050, reaching 21.11 million (95% UI: 9.97, 32.25) and 3.36 million (95% UI: 0.64, 6.09), respectively, with the burden remaining higher among individuals of female gender (Tables S10–S12).

## 4 Discussion

This study established a systematic and hierarchical analytical framework that, for the first time, comprehensively investigates the role of lifestyle and dietary factors in the development and epidemiology of RA across multiple dimensions, including causal inference, risk prediction, and burden assessment. By employing a two‐sample MR approach, the study effectively mitigated common issues in traditional observational research, such as confounding bias and reverse causation, thereby providing more robust causal evidence for the association between lifestyle, dietary exposures, and RA. Building upon these findings, individual‐level data from NHANES were integrated to refine the selection of causal variables and develop predictive models. Multiple machine learning algorithms were employed to construct a robust RA risk prediction tool. Furthermore, population‐level data from the GBD study were incorporated to assess disease burden and health inequality, thereby establishing a cross‐level research system. The analytical components were seamlessly integrated, forming a closed‐loop research pathway from causal identification to risk prediction and burden projection. This comprehensive approach offers novel insights and multidimensional evidence to better elucidate the pathogenesis of RA and to inform the development of targeted prevention and control strategies.

MR analysis indicated that obesity increases the risk of RA by inducing low‐grade systemic inflammation and elevating levels of proinflammatory cytokines (Hayer et al. 2022; Martin et al. 2021). While cumulative exposure to long‐term smoking poses a greater risk, even moderate smoking can significantly increase the likelihood of developing RA, and the elevated risk may persist for several years after cessation (Liu et al. 2019). This study also found a positive association between insomnia and RA; however, this may be attributed to factors such as joint pain, physical limitations, and medication use in active RA rather than indicating that sleep disorders are an independent etiological factor (Gao et al. 2022). However, Michale's research demonstrates that patients with RA, particularly those with comorbid conditions, exhibit elevated levels of systemic inflammation. Excessive inflammatory afferent signaling disrupts sleep architecture, while the concomitant activation of autonomic excitatory pathways in RA further exacerbates sleep disturbances. Importantly, effective treatment of insomnia may enhance patients' capacity to adapt to the physiological burden imposed by chronic inflammatory disease, thereby mitigating inflammation‐related functional impairment in RA (Irwin et al. 2023). In addition, excessive salt intake may activate proinflammatory pathways, potentially exacerbating inflammatory responses in autoimmune diseases (Afsar and Afsar 2023). Therefore, promoting public awareness of physical activity and implementing smoking cessation interventions may serve as primary prevention and non‐pharmacological strategies for RA. Improving sleep quality and moderating salt intake could function as supportive interventions, offering a more comprehensive approach to RA prevention and management.

This study identified a negative causal relationship between pork consumption and RA, whereas poultry intake showed a positive association. These findings differ from the Mediterranean dietary framework, which emphasizes poultry while limiting red meat. Although a systematic review has suggested that the Mediterranean diet may benefit patients with RA (Forsyth et al. 2018), the focus was on the overall dietary pattern rather than the effects of individual food items. Pork may exert a protective effect through nutrients such as zinc, selenium, and B vitamins, potentially acting as mediating components within broader dietary patterns (Agarwal and Fulgoni III 2023), thereby contributing to a reduced risk of RA. In contrast, poultry, particularly when consumed in forms involving high‐temperature frying or heavy seasoning, may promote chronic inflammatory responses (Szypowska et al. 2023). Genetic instruments used in MR analyses typically capture an individual's long‐term propensity for consuming a given category of foods, rather than distinguishing specific cooking methods or degrees of processing (e.g., processed versus unprocessed meats), factors that may play a critical role in shaping inflammatory responses (Davies et al. 2018). Moreover, diet‐associated genetic variants may partially reflect broader behavioral or social environmental characteristics, such as socioeconomic status, overall dietary patterns, or lifestyle factors, thereby introducing the potential for horizontal pleiotropy (Brumpton et al. 2020). Although MR offers a powerful approach to mitigating confounding and reverse causation, its findings should be interpreted with caution until corroborated by well‐designed prospective studies. We also observed a protective association between cheese consumption and RA, which may be attributable to the probiotic content of cheese (Ciccia et al. 2016; Reyes‐Castillo et al. 2021). A 3‐month double‐blind, placebo‐controlled trial reported significant clinical improvement in patients with RA following supplementation with *Lactobacillus rhamnosus* and *Lactobacillus reuteri* (Pineda et al. 2011). Consistently, another study demonstrated that probiotic administration ameliorated RA across multiple animal models, an effect likely mediated by the enrichment of CD4^+^Foxp3^+^ regulatory T cells within inflammatory microenvironments (Kwon et al. 2010). These findings highlight the need for caution when interpreting causal relationships between individual food items and disease outcomes; dietary patterns and genetic background should be considered in future research. Additionally, no significant causal associations were found between RA and micronutrients such as magnesium, potassium, or vitamin D. This may be due to the GWAS datasets used, which were primarily based on European populations and may not fully represent low‐income settings.

In this study, a RA risk prediction model was developed based on nationally representative individual‐level data from the NHANES, thereby validating and extending the practical applicability of findings obtained from prior causal inference analyses. The seven variables selected through LASSO regression were all consistent with epidemiological and biological mechanisms previously reported in RA‐related studies (Fang et al. 2023; Liu et al. 2024). Although Mendelian randomization analyses identified several factors with putative causal associations with RA, such as cheese and pork consumption, these variables were not incorporated into the final predictive model because their measurement in NHANES was incomplete or their information content was limited for individual‐level prediction. SHAP analysis indicated that age had the strongest positive influence on model predictions, which may be attributed to age‐related decline in immune cell function—such as reduced T cell activity—that contributes to RA development (van Onna and Boonen 2016). Although magnesium intake did not exhibit a clear causal relationship in the MR analysis, it emerged as an important predictive factor in the model, suggesting that it may influence RA risk through nongenetic mechanisms, such as regulating inflammatory mediators (e.g., prostaglandin E2), thereby indirectly contributing to disease onset (Arablou et al. 2019). Additionally, it should be noted that certain dietary factors with significant causal associations identified in the MR analysis (such as intake of pork, poultry, and cheese) were not included in the machine learning model due to missing or insufficient measurements in the NHANES dataset. This limitation underscores the differences in variable coverage and comparability between genetic and observational datasets, and further highlights the importance of integrating multisource data to improve the interpretability and predictive performance of RA risk models.

We observed notable gender and age disparities in the RA burden. Globally and across most regions, both ASRs and total case numbers were higher in individuals of female gender compared to males, and the burden was disproportionately greater among older age groups. Previous studies have explored this gender difference, proposing potential explanations such as hormonal and genetic factors, as well as lower treatment efficacy in females compared to males (Alpízar‐Rodríguez et al. 2017; Kvien et al. 2006). The burden of RA also varied significantly across regions, which may be attributed to differences in lifestyle factors such as smoking and obesity, environmental exposures such as pollutants, and genetic predisposition (Zou et al. 2023). Analyses of health inequality and frontier performance indicate that certain low‐SDI countries, such as Somalia, appear to exhibit relatively narrow gaps from the estimated frontier, a pattern that may partially reflect limitations in healthcare infrastructure and disease surveillance capacity. In low‐ and middle‐income countries, structural barriers—including shortages of rheumatology specialists, limited access to diagnostic tools, cultural factors, and incomplete health records—collectively contribute to the underdiagnosis and underreporting of RA and related conditions (Maldonado et al. 2023). These findings highlight the need for such countries to strengthen primary healthcare systems, improve the training of medical personnel, and enhance disease monitoring frameworks. Projections indicate a continued increase in the global RA burden by 2050, underscoring the urgency of comprehensive RA control strategies worldwide. Adequate resource allocation for preventive and risk‐reduction programs targeting modifiable lifestyle factors, such as obesity, smoking, and sleep disorders, as well as dietary components, such as magnesium and salt intake, will be essential. Moreover, RA mitigation strategies should prioritize age‐ and gender‐related disparities by integrating personalized treatment approaches and optimizing long‐term disease management to achieve better population health outcomes.

Taken together, the integrated analysis combining MR, ML, and GBD approaches provides a complementary and hierarchically structured framework for evaluating RA risk and disease burden across causal, individual, and population levels. MR analyses, leveraging genetic instrumental variables, identified multiple potentially causal and modifiable risk factors—including smoking, obesity, salt intake, and sleep‐related traits—thereby offering relatively robust etiological evidence to inform primary prevention strategies for RA. Using nationally representative individual‐level data from NHANES, ML models further substantiated the predictive relevance of a subset of these factors, particularly smoking status and sleep duration, highlighting their practical utility for individual risk stratification and precision identification. At the population level, GBD analyses revealed a sustained global increase in RA burden, disproportionately affecting women and older adults, alongside pronounced regional disparities and health inequalities, underscoring the urgency of implementing targeted interventions in high‐risk populations and resource‐limited settings. By systematically linking causal inference, individual risk prediction, and population burden assessment, this multilevel integrative framework not only enhances consistency and interpretability across analytical scales but also substantially strengthens the translational relevance of the findings for RA prevention strategies and public health decision‐making.

This study has several limitations. First, the MR analysis was based on GWAS conducted in European populations, which may limit the generalizability of the findings due to population stratification and ethnic heterogeneity. Second, the cross‐sectional design is subject to inherent biases, including potential misclassification from self‐reported RA diagnoses and recall bias in dietary assessments. Third, the lack of external validation using independent, multicenter cohorts restricts the evaluation of the model's stability, interpretability, and applicability across diverse clinical settings and populations. Finally, estimates from GBD 2021 are derived from large‐scale modeling approaches; particularly in low‐ and middle‐income countries, regional disparities in healthcare accessibility and diagnostic accuracy may introduce systematic bias, leading to underdiagnosis and underreporting of RA. Future research should aim to enhance population diversity, adopt longitudinal designs, incorporate multicenter validation, and utilize multidimensional data sources to strengthen causal inference, improve the clinical utility of prediction models, and increase the precision of disease burden estimates. These efforts are essential to support evidence‐based strategies for the targeted prevention and management of RA, and to advance global health equity.

## 5 Conclusions

This study identified lifestyle and dietary factors causally associated with RA using MR analysis and developed an efficient individual‐level risk prediction model for RA. The global burden analysis revealed gender differences and health inequities in RA‐related disease burden. Future RA prevention and control strategies should focus on targeted lifestyle interventions and nutritional optimization among high‐risk populations, with an emphasis on BMI management, smoking cessation, sleep improvement, and healthy dietary practices. Moreover, integrating primary prevention of RA into chronic disease management and establishing a comprehensive “prevention–screening–control” framework may offer a practical pathway to reducing the global RA burden.

## Author Contributions

**Yan Gao:** conceptualization, methodology, software, data curation, formal analysis, visualization, writing – original draft, writing – review editing, supervision. **Guangxin Gu:** conceptualization, methodology, software, data curation, writing – original draft. **Ruiwen Wang:** conceptualization, methodology, software, data curation, formal analysis. **Wenfeng Han:** data curation, formal analysis. **Hailong Yu:** writing – review editing, supervision. **Chen Jia:** supervision, writing – review editing. **Yu Wang:** supervision, writing – review editing. Yan Gao, Guangxin Gu, and Ruiwen Wang contributed equally to this work. All the authors contributed to the article and approved the submitted version.

## Funding

This work was supported by the Liaoning Provincial Science and Technology Plan Joint Program (Applied Basic Research Project) [2023JH2/101700124] and the Liaoning Provincial Science and Technology Plan Joint Program (Technology Research and Development Project) [2024JH2/102600270].

## Ethics Statement

The authors have nothing to report.

## Conflicts of Interest

The authors declare no conflicts of interest.

## Supporting information


**Table S1:** Detailed information regarding studies and datasets used in the present study.
**Table S2:** Single nucleotide polymorphisms (SNPs) associated with lifestyle and dietary factors (used as instrumental variables in Mendelian randomization analysis).
**Table S3:** The causal relationship between lifestyle and dietary factors and rheumatoid arthritis.
**Table S4:** Baseline characteristics of participants from National Health and Nutrition Examination Survey (NHANES) 2007–2014.
**Table S5:** Complete performance results of machine learning models based on training–testing split and cross‐validation.
**Table S6:** Numbers of cases for prevalence of rheumatoid arthritis at the global and regional levels in 1990 and 2021, and their estimated annual percentage changes from 1990 to 2021.
**Table S7:** Numbers of cases for DALYs of rheumatoid arthritis at the global and regional levels in 1990 and 2021, and their estimated annual percentage changes from 1990 to 2021.
**Table S8:** Prevalence cases and age‐standardized rates of rheumatoid arthritis across 204 countries and territories in 1990 and 2021, and their estimated annual percentage changes from 1990 to 2021.
**Table S9:** DALYs and age‐standardized rate of rheumatoid arthritis across 204 countries and territories in 1990 and 2021, and their estimated annual percentage changes from 1990 to 2021.
**Table S10:** Age‐standardized DALYs rate of rheumatoid arthritis with frontier analysis across 204 countries and territories in 1990 and 2021.
**Table S11:** Projected number of prevalent cases and age‐standardized prevalence rates of rheumatoid arthritis worldwide from 2022 to 2050 based on the BAPC model, by sex.
**Table S12:** Projected number of DALYs and age‐standardized DALY rates of rheumatoid arthritis worldwide from 2022 to 2050 based on the BAPC model, by sex.
**Figure S1:** Flowchart of participant selection.
**Figure S2:** Feature selection for rheumatoid arthritis prediction using LASSO regression.
**Figure S3:** Calibration curves of different machine learning models.
**Figure S4:** Decision curve analysis (DCA) of different machine learning models.
**Figure S5:** Receiver operating characteristic (ROC) curves from cross‐validation.
**Figure S6:** Comparison of model performance on cross‐validation and test sets for overfitting analysis.
**Figure S7:** Confusion matrices of nine machine learning models on the test set.

## Data Availability

For the Mendelian randomization analysis, all summary‐level genetic data were obtained from the publicly available IEU Open GWAS database (https://opengwas.io/datasets/). The individual‐level data for this study were derived from the National Health and Nutrition Examination Survey (NHANES) (https://www.cdc.gov/nchs/nhanes/index.htm). Additionally, data from the Global Burden of Disease (GBD) studies were utilized, for which the Institutional Review Board of the University of Washington reviewed and approved a waiver of informed consent (https://www.healthdata.org/research‐analysis/gbd).
