# Supplementary material for: Causal Effects of Lifestyle and Dietary Factors on Rheumatoid Arthritis: An Integrated Analysis Combining Mendelian Randomization, Machine Learning, and Evaluation of Burden Dynamics and Health Inequality
Source: Food Sci Nutr. 2026 Feb 22;14(2):e71584. doi: 10.1002/fsn3.71584 (PMC12927935; doi:10.1002/fsn3.71584)
Supplement: Supplementary file 1 — Table S1: Detailed information regarding studies and datasets used in the present study. Table S2: Single nucleotide polymorphisms (SNPs) associated with lifestyle and dietary factors (used as instrumental variables in Mendelian randomization analysis). Table S3: The causal relationship between lifestyle and dietary factors and rheumatoid arthritis. Table S4: Baseline characteristics of participants from National Health and Nutrition Examination Survey (NHANES) 2007–2014. Table S5: Complete performance results of machine learning models based on training–testing split and cross‐validation. Table S6: Numbers of cases for prevalence of rheumatoid arthritis at the global and regional levels in 1990 and 2021, and their estimated annual percentage changes from 1990 to 2021. Table S7: Numbers of cases for DALYs of rheumatoid arthritis at the global and regional levels in 1990 and 2021, and their estimated annual percentage changes from 1990 to 2021. Table S8: Prevalence cases and age‐standardized rates of rheumatoid arthritis across 204 countries and territories in 1990 and 2021, and their estimated annual percentage changes from 1990 to 2021. Table S9: DALYs and age‐standardized rate of rheumatoid arthritis across 204 countries and territories in 1990 and 2021, and their estimated annual percentage changes from 1990 to 2021. Table S10: Age‐standardized DALYs rate of rheumatoid arthritis with frontier analysis across 204 countries and territories in 1990 and 2021. Table S11: Projected number of prevalent cases and age‐standardized prevalence rates of rheumatoid arthritis worldwide from 2022 to 2050 based on the BAPC model, by sex. Table S12: Projected number of DALYs and age‐standardized DALY rates of rheumatoid arthritis worldwide from 2022 to 2050 based on the BAPC model, by sex. Figure S1: Flowchart of participant selection. Figure S2: Feature selection for rheumatoid arthritis prediction using LASSO regression. Figure S3: Calibration curves of different machine learni [file FSN3-14-e71584-s001.doc]

**Additional file 1:**

**Causal effects of lifestyle and dietary factors on rheumatoid arthritis: an integrated analysis combining Mendelian randomization, Machine learning, and evaluation of burden dynamics and health in equality.**

Yan Gao ^1^ ^†^, Guangxin Gu ^2, 3^, ^†^, Ruiwen Wang ^2, 3^, ^†^, Hailong Yu ^4^, Wenfeng Han^4^, Chen Jia^1^ *, Yu Wang ^4^, *

^†^Yan Gao, Guangxin Gu and Ruiwen Wang contributed equally to this study.

^1^ Department of Disease Prevention and Control, General Hospital of Northern Theater Command, Shenyang, China

^2^ Key Laboratory of Environmental Stress and Chronic Disease Control & Prevention, China Medical University, Ministry of Education, Shenyang, China

^3^ Department of Epidemiology, School of Public Health, China Medical University, Shenyang, China

^4^ Department of Orthopedics, General Hospital of Northern Theater Command, Shenyang, China

***Corresponding authors**:

Dr. Chen Jia

Department of Disease Prevention and Control, General Hospital of Northern Theater Command

83 Wenhua Road, Shenyang, Liaoning, China

Tel: +8613840353576

Email: jc838703809@163.com

Dr. Yu Wang

Department of Orthopedics, General Hospital of Northern Theater Command

83 Wenhua Road, Shenyang, Liaoning, China

Tel: +8617602410872

Email: wangyu110016@163.com

**Contents**

[**Supplementary methods** 1](#__RefHeading___Toc205505377)

[**NHANES machine learning for RA** 1](#__RefHeading___Toc205505378)

[**Global trends and health inequality in RA burden** 3](#__RefHeading___Toc205505379)

[**Table S1. Detailed in information regarding studies and datasets used in the present study.** 1](#__RefHeading___Toc205505380)

[**Table S2. Single nucleotide polymorphisms (SNPs) associated with lifestyle and dietary factors (used as instrumental variables in Mendelian randomization analysis).** 3](#__RefHeading___Toc205505381)

[**Table S3. The causal relationship between lifestyle and dietary factors and rheumatoid arthritis.** 145](#__RefHeading___Toc205505382)

[**Table S4. Baseline characteristics of participants from National health and nutrition examination survey (NHANES) 2007–2014.** 149](#__RefHeading___Toc205505383)

[**Table S5. Numbers of cases for prevalence of rheumatoid arthritis at the global and regional levels in 1990 and 2021, and their estimated annual percentage changes from 1990 to 2021.** 152](#__RefHeading___Toc205505384)

[**Table S6. Numbers of cases for DALYs of rheumatoid arthritis at the global and regional levels in 1990 and 2021, and their estimated annual percentage changes from 1990 to 2021.** 155](#__RefHeading___Toc205505385)

[**Table S7. Prevalence cases and age-standardised rates of rheumatoid arthritis across 204 countries and territories in 1990 and 2021, and their estimated annual percentage changes from 1990 to 2021.** 158](#__RefHeading___Toc205505386)

[**Table S8. DALYs and age-standardised rate of rheumatoid arthritis across 204 countries and territories in 1990 and 2021, and their estimated annual percentage changes from 1990 to 2021.** 168](#__RefHeading___Toc205505387)

[**Table S9. Age-standardised DALYs rate of rheumatoid arthritis with frontier analysis across 204 countries and territories in 1990 and 2021.** 177](#__RefHeading___Toc205505388)

[**Table S10. Projected number of prevalent cases and age-standardised prevalence rates of rheumatoid arthritis worldwide from 2022 to 2050 based on the BAPC model, by sex.** 186](#__RefHeading___Toc205505389)

[**Table S11. Projected number of DALYs and age-standardised DALY rates of rheumatoid arthritis worldwide from 2022 to 2050 based on the BAPC model, by sex.** 189](#__RefHeading___Toc205505390)

[**Figure. S1. Flowchart of participant selection.** 192](#__RefHeading___Toc205505391)

[**Figure. S2. Feature selection for rheumatoid arthritis prediction using LASSO regression.** 193](#__RefHeading___Toc205505392)

[**Figure. S3.** **Receiver operating characteristic (ROC) curves from cross-validation.** 194](#__RefHeading___Toc205505393)

[**Figure. S4. Comparison of model performance on cross-validation and test sets for overfitting analysis.** 195](#__RefHeading___Toc205505394)

[**Figure. S5. Confusion matrices of nine machine learning models on the test set.** 196](#__RefHeading___Toc205505395)

**Supplementary methods**

**NHANES machine learning for RA**

**Data sources and study population**

The data used in this study were derived from the 2007–2014 National Health and Nutrition Examination Survey (NHANES), which is conducted by the National Center for Health Statistics (NCHS) and is widely regarded as an authoritative source for assessing the health and nutritional status of the U.S. population (<https://wwwn.cdc.gov/nchs/nhanes>) [1]. Across the four survey cycles from 2007 to 2014, the initial sample included 40,617 participants. Based on demographic baseline data and Mendelian randomization (MR)-related analytical variables, the covariates included in this study were: age, sex, race/ethnicity, education level, marital status, poverty-income ratio, body mass index (BMI), smoking, alcohol consumption, fish intake, sleep disorders, coffee intake, dietary copper, dietary magnesium, sodium intake, and intake of vitamins A, B6, B12, C, D, E, and folate. After excluding cases with missing data, the final analytical sample consisted of 8,446 participants.

Since the early 1970s, NHANES has collected various forms of dietary intake data, primarily through 24-hour dietary recalls (implemented since NHANES I), along with food frequency questionnaires (FFQs) that have varied slightly across different survey cycles. The 24-hour dietary recall is the most commonly used method for assessing dietary intake in large-scale population studies. NHANES has continued to use this method for many years, based on expert consensus reached in regular methodological workshops [2-4]. In this study, we applied the Random Forest (RF) [5] algorithm to impute missing values by leveraging information from other variables.

**Machine learning strategy**

We employed the Least Absolute Shrinkage and Selection Operator (LASSO) regression to perform variable selection. The variables selected by LASSO were subsequently incorporated into various machine learning models. All models underwent rigorous hyperparameter tuning and utilized ten-fold cross-validation to optimize predictive performance and enhance generalizability [6]. This process served a dual purpose: on the one hand, to assess the predictive capability and association strength of each variable in the progression of rheumatoid arthritis; on the other hand, to evaluate their contribution to improving overall model accuracy and performance. The final features included in the machine learning models were gender, age, smoking status, income level, body mass index (BMI), presence of sleep disorders, and dietary magnesium intake.

Data preprocessing steps included one-hot encoding for categorical variables and standardization of all features using StandardScaler. To address class imbalance in the dataset (with a case-to-control ratio of 1:9.26), we adopted a class weight balancing strategy by assigning higher weights to the minority class during model training. The dataset was stratified and split into training and testing sets in an 8:2 ratio. We evaluated nine machine learning algorithms: Random Forest (RF), Gradient Boosting (GB), XGBoost, Light Gradient Boosting Machine (LightGBM), Extremely Randomized Trees (ET), AdaBoost, K-Nearest Neighbors (KNN), Decision Tree (DT), and Naive Bayes (NB) [7-11]. All models were tuned using Bayesian optimization (BayesSearchCV) combined with five-fold stratified cross-validation. Considering the class imbalance, we searched for the optimal classification threshold for each model within the range of 0.1 to 0.9 through cross-validation, rather than using the default threshold of 0.5. Model performance was primarily evaluated using the area under the Receiver Operating Characteristic curve (AUROC), where a value closer to 1 indicates stronger discriminative ability.

To enhance model interpretability, we applied the SHAP (SHapley Additive exPlanations) framework to the best-performing model. SHAP, based on the concept of Shapley values from game theory [12, 13], quantifies the contribution of each feature for every prediction instance, providing both local and global interpretability. Through SHAP analysis, we were able not only to identify the most influential features affecting the prediction outcomes but also to uncover nonlinear relationships between features and model outputs. This enabled a more comprehensive understanding of the model's decision-making process and provided a consistent and fair explanation path.

**Global trends and health inequality in RA burden**

**Data source and disease definition**

The Global Burden of Disease (GBD) 2021 study systematically assessed the health burden caused by 371 diseases and injuries across 204 countries and territories and 811 subregions from 1990 to 2021. In this study, epidemiological data on rheumatoid arthritis (RA) were obtained via the visualization query tool available on the Global Health Data Exchange (GHDx) platform（https://vizhub.healthdata.org/gbd-results/）[14], with a focus on prevalence, disability-adjusted life years (DALYs), and the overall disease burden. All indicators were presented with 95% uncertainty intervals (UIs) to reflect the range of plausible estimates.

In the GBD 2021 study, RA cases were defined based on the classification criteria issued by the American College of Rheumatology (ACR) in 1987 [15]. Diagnoses were made according to the International Classification of Diseases, 10th Revision (ICD-10), specifically including codes M05–M05.9 and M08–M09.8.

**Estimated annual percentage change and percentage change**

This study utilized the age-standardized rate (ASR) to calculate the estimated annual percentage change (EAPC), in order to analyze temporal trends in the prevalence and DALY rates of rheumatoid arthritis from 1990 to 2021. EAPC provides a standardized quantitative approach to evaluate the temporal evolution of disease burden by quantifying the average annual rate of change in specific health metrics [16].

**Socio-demographic index (SDI)**

At the socioeconomic level, this study employed the Sociodemographic Index (SDI) to assess disparities in disease burden across countries and regions with varying levels of development. The SDI is a composite development indicator closely associated with health outcomes, calculated based on per capita income, average years of education, and total fertility rate. Its values range from 0 to 1, reflecting the overall level of socioeconomic development in each country [17].

**Cross-country inequality analysis**

To systematically assess health inequalities in the burden of rheumatoid arthritis, this study adopted the health inequality analysis framework recommended by the World Health Organization (WHO), analyzing disparities among different socioeconomic groups across multiple dimensions, including health status, disease burden, healthcare accessibility, and health outcomes [18, 19]. Specifically, the Slope Index of Inequality (SII) was used through regression analysis to assess the association between the burden of rheumatoid arthritis in each country and the Sociodemographic Index (SDI). The study first ranked countries by their SDI levels to determine their relative positions within the cumulative population distribution, then constructed Lorenz concentration curves to compare the cumulative distribution of disease burden with the cumulative distribution of the population. By integrating the values of this curve, the Concentration Index (CI) was calculated to quantitatively measure the degree of inequality in the distribution of disease burden among countries with different levels of social development [20, 21].

**Frontier analysis and decomposition analysis**

Furthermore, this study applied Frontier Analysis to construct a "survival potential frontier curve" in order to identify benchmark countries that achieved the lowest age-standardized prevalence and DALY rates at various SDI levels, thereby establishing best-practice reference points for disease prevention and control [22]. By comparing each country’s actual burden of disease against the frontier benchmarks, the study assessed their relative performance in rheumatoid arthritis prevention and control, thereby revealing the potential room for improvement in managing the disease burden across different countries.

**Decomposition analysis**

In addition, this study employed decomposition analysis to break down changes in the burden of rheumatoid arthritis into three contributing components: the population growth effect, the population aging effect, and the epidemiological change effect. This method helps to elucidate the primary drivers of disease burden changes across different regions and SDI groupings [23].

**BAPC model projection**

Finally, this study employed the Bayesian Age-Period-Cohort (BAPC) model to forecast the incidence of rheumatoid arthritis in different gender populations globally, aiming to depict its epidemiological characteristics and predict the trends in case numbers and age-standardized incidence rates (ASRs) through 2050. The advantage of this model lies in its ability to simultaneously capture and analyze the interactions between age effects, period effects, and birth cohort effects, thereby providing a more precise and comprehensive basis for the assessment of rheumatoid arthritis disease burden and its future development trends [24].

**References**

1. Wu L-D, Chu P, Kong C-H, Shi Y, Zhu M-H, Xia Y-Y, Li Z, Zhang J-X, Chen S-L: Estimated pulse wave velocity is associated with all-cause mortality and cardiovascular mortality among adults with diabetes. Frontiers in cardiovascular medicine 2023, 10:1157163.

2. Gibson RS: Principles of nutritional assessment. Oxford university press; 2005.

3. Archer DD: Edward Archer, PhD, MS Chief Science Officer EnduringFX PO Box 11695 Columbia, SC 29211 850-570-3162. 2018.

4. Wright JD, Ervin B, Briefel RR: Consensus workshop on dietary assessment: nutrition monitoring and tracking the year 2000 objectives. US Department of Health and Human Services, Public Health Service, Centers …; 1994.

5. Shah A, Bartlett J, Hemingway H, Nicholas O, Hingorani H: CALIBERrfimpute: Imputation in MICE using Random Forest. 2014.

6. Tibshirani R: Regression shrinkage and selection via the lasso. Journal of the Royal Statistical Society Series B: Statistical Methodology 1996, 58:267-288.

7. Bekele WT: Machine learning algorithms for predicting low birth weight in Ethiopia. BMC medical informatics and decision making 2022, 22:232.

8. Atalan A, Şahin H, Atalan YA: Integration of machine learning algorithms and discrete-event simulation for the cost of healthcare resources. In Healthcare. MDPI; 2022: 1920.

9. Zuo D, Yang L, Jin Y, Qi H, Liu Y, Ren L: Machine learning-based models for the prediction of breast cancer recurrence risk. BMC Medical Informatics and Decision Making 2023, 23:276.

10. Ahamed Fayaz S, Babu L, Paridayal L, Vasantha M, Paramasivam P, Sundarakumar K, Ponnuraja C: Machine learning algorithms to predict treatment success for patients with pulmonary tuberculosis. PLoS One 2024, 19:e0309151.

11. Lyu Y, Wu H-M, Yan H-X, Guo R, Xiong Y-J, Chen R, Huang W-Y, Hong J, Lyu R, Wang Y-Q: Classification of coronary artery disease using radial artery pulse wave analysis via machine learning. BMC Medical Informatics and Decision Making 2024, 24:256.

12. Lundberg SM, Lee S-I: A unified approach to interpreting model predictions. Advances in neural information processing systems 2017, 30.

13. Wolters S: Trustworthy machine learning: mitigating bias and promoting fairness in automated decision systems. ETSI_Informatica, 2023.

14. Naghavi M, Ong KL, Aali A, Ababneh HS, Abate YH, Abbafati C, Abbasgholizadeh R, Abbasian M, Abbasi-Kangevari M, Abbastabar H: Global burden of 288 causes of death and life expectancy decomposition in 204 countries and territories and 811 subnational locations, 1990–2021: a systematic analysis for the Global Burden of Disease Study 2021. The Lancet 2024, 403:2100-2132.

15. Arnett FC, Edworthy SM, Bloch DA, Mcshane DJ, Fries JF, Cooper NS, Healey LA, Kaplan SR, Liang MH, Luthra HS: The American Rheumatism Association 1987 revised criteria for the classification of rheumatoid arthritis. Arthritis & Rheumatism: Official Journal of the American College of Rheumatology 1988, 31:315-324.

16. Ding Q, Liu S, Yao Y, Liu H, Cai T, Han L: Global, regional, and national burden of ischemic stroke, 1990–2019. Neurology 2022, 98:e279-e290.

17. Cousin E, Duncan BB, Stein C, Ong KL, Vos T, Abbafati C, Abbasi-Kangevari M, Abdelmasseh M, Abdoli A, Abd-Rabu R: Diabetes mortality and trends before 25 years of age: an analysis of the Global Burden of Disease Study 2019. The Lancet diabetes & endocrinology 2022, 10:177-192.

18. Cao F, He Y-S, Wang Y, Zha C-K, Lu J-M, Tao L-M, Jiang Z-X, Pan H-F: Global burden and cross-country inequalities in autoimmune diseases from 1990 to 2019. Autoimmunity Reviews 2023, 22:103326.

19. Howe LD: Handbook on health inequality monitoring. Oxford University Press; 2014.

20. Organization WH: Handbook on health inequality monitoring: with a special focus on low-and middle-income countries. World Health Organization; 2013.

21. Xie Y, Bowe B, Mokdad AH, Xian H, Yan Y, Li T, Maddukuri G, Tsai C-Y, Floyd T, Al-Aly Z: Analysis of the Global Burden of Disease study highlights the global, regional, and national trends of chronic kidney disease epidemiology from 1990 to 2016. Kidney international 2018, 94:567-581.

22. GUPTA PD: Standardization and decomposition of rates from cross-classified data. Genus 1994:171-196.

23. Riebler A, Held L: Projecting the future burden of cancer: Bayesian age–period–cohort analysis with integrated nested Laplace approximations. Biometrical Journal 2017, 59:531-549.

24. Tu Y-K, Krämer N, Lee W-C: Addressing the identification problem in age-period-cohort analysis: a tutorial on the use of partial least squares and principal components analysis. Epidemiology 2012, 23:583-593.

**Table S1. Detailed in information regarding studies and datasets used in the present study.**

| **Classification** | **Exposures** | **Population** | **Sample size** | **GWAS ID** |
| --- | --- | --- | --- | --- |
| Lifestyle | Body mass index (BMI) | European | 454884 | ukb-b-2303 |
| Lifestyle | Obesity | European | 13848 | ebi-a-GCST001475 |
| Lifestyle | Obesity class 1 | European | 98697 | ieu-a-90 |
| Lifestyle | Obesity class 2 | European | 72546 | ieu-a-91 |
| Lifestyle | Obesity class 3 | European | 50364 | ieu-a-92 |
| Lifestyle | smoking initiation | European | 607291 | ieu-b-4877 |
| Lifestyle | Current tobacco smoking | European | 462434 | ukb-b-223 |
| Lifestyle | Smoking status: Never | European | 359706 | ukb-d-20116_0 |
| Lifestyle | Alcohol intake frequency | European | 336965 | ukb-a-25 |
| Lifestyle | Average weekly beer plus cider intake | European | 327634 | ukb-b-5174 |
| Lifestyle | Average weekly red wine intake | European | 327026 | ukb-b-5239 |
| Lifestyle | Coffee intake | European | 428860 | ukb-b-5237 |
| Lifestyle | Tea intake | European | 447485 | ukb-b-6066 |
| Lifestyle | Moderate to vigorous physical activity levels | European | 377234 | ebi-a-GCST006097 |
| Lifestyle | Active to sedentary transition probability | European | 88411 | ebi-a-GCST90061429 |
| Lifestyle | Sleeplessness / insomnia | European | 462341 | ukb-b-3957 |
| Dietary | Bacon intake | European | 64949 | ukb-b-4414 |
| Dietary | Beef intake | European | 461053 | ukb-b-2862 |
| Dietary | Lamb/mutton intake | European | 460006 | ukb-b-14179 |
| Dietary | Pork intake | European | 460162 | ukb-b-5640 |
| Dietary | Poultry intake | European | 461900 | ukb-b-8006 |
| Dietary | Processed meat intake | European | 461981 | ukb-b-6324 |
| Dietary | Oily fish intake | European | 460443 | ukb-b-2209 |
| Dietary | Non-oily fish intake | European | 460880 | ukb-b-17627 |
| Dietary | Fresh fruit intake | European | 446462 | ukb-b-3881 |
| Dietary | Dried fruit intake | European | 421764 | ukb-b-16576 |
| Dietary | Cooked vegetable intake | European | 448651 | ukb-b-8089 |
| Dietary | Salad / raw vegetable intake | European | 435435 | ukb-b-1996 |
| Dietary | Milk intake | European | 64943 | ukb-b-2966 |
| Dietary | Cheese intake | European | 451486 | ukb-b-1489 |
| Dietary | Cereal intake | European | 441640 | ukb-b-15926 |
| Dietary | Salt added to food | European | 462630 | ukb-b-8121 |
| Micronutrients | Copper | European | 2603 | ieu-a-1073 |
| Micronutrients | Magnesium | European | 64979 | ukb-b-7372 |
| Micronutrients | Potassium | European | 64979 | ukb-b-17881 |
| Micronutrients | Folate | European | 64979 | ukb-b-11349 |
| Micronutrients | Vitamin and mineral supplements: Vitamin A | European | 335591 | ukb-a-458 |
| Micronutrients | Vitamin B6 | European | 64979 | ukb-b-7864 |
| Micronutrients | Vitamin B12 | European | 64979 | ukb-b-19524 |
| Micronutrients | Vitamin C | European | 64979 | ukb-b-19390 |
| Micronutrients | Vitamin D | European | 64949 | ukb-b-18593 |
| Micronutrients | Vitamin E | European | 64979 | ukb-b-6888 |

**Abbreviation: GWAS, genome-wide association study.**

**Table S2. Single nucleotide polymorphisms (SNPs) associated with lifestyle and dietary factors (used as instrumental variables in Mendelian randomization analysis).**

| **Phenotype** | **SNP** | **Chr** | **Position** | **Effect allele** | **Other allele** | **EAF** | **Effect** | **SE** | **P-value** | **N** | **F-statistic** |
| --- | --- | --- | --- | --- | --- | --- | --- | --- | --- | --- | --- |
| Body mass index (BMI) | rs10063055 | 5 | 140990108 | T | C | 0.253 | 0.013 | 0.002 | 1.40001e-08 | 454884 | 32.183 |
| Body mass index (BMI) | rs10093572 | 8 | 137446430 | C | T | 0.206 | 0.012 | 0.002 | 1.6e-06 | 454884 | 22.988 |
| Body mass index (BMI) | rs1017529 | 17 | 27912415 | A | C | 0.175 | 0.015 | 0.003 | 1.2e-08 | 454884 | 32.483 |
| Body mass index (BMI) | rs10182416 | 2 | 104242992 | G | A | 0.512 | 0.013 | 0.002 | 1.80011e-11 | 454884 | 45.151 |
| Body mass index (BMI) | rs10184537 | 2 | 50742025 | T | C | 0.339 | -0.017 | 0.002 | 3.59998e-15 | 454884 | 61.932 |
| Body mass index (BMI) | rs10217047 | 8 | 74809210 | A | C | 0.096 | 0.019 | 0.003 | 1.5e-08 | 454884 | 32.066 |
| Body mass index (BMI) | rs10402950 | 19 | 33935675 | C | T | 0.289 | 0.014 | 0.002 | 6.4e-10 | 454884 | 38.192 |
| Body mass index (BMI) | rs10417386 | 19 | 1950042 | C | T | 0.696 | 0.012 | 0.002 | 1.29999e-08 | 454884 | 32.341 |
| Body mass index (BMI) | rs10438889 | 18 | 9287829 | T | C | 0.045 | -0.023 | 0.005 | 2e-06 | 454884 | 22.557 |
| Body mass index (BMI) | rs1048637 | 3 | 13358171 | G | T | 0.452 | 0.01 | 0.002 | 2.80001e-07 | 454884 | 26.415 |
| Body mass index (BMI) | rs1048932 | 11 | 115044850 | A | C | 0.413 | -0.015 | 0.002 | 2.09991e-14 | 454884 | 58.476 |
| Body mass index (BMI) | rs10505836 | 12 | 19288508 | C | A | 0.86 | 0.018 | 0.003 | 7.59994e-10 | 454884 | 37.848 |
| Body mass index (BMI) | rs10510025 | 10 | 118650996 | T | C | 0.247 | 0.018 | 0.002 | 5.00035e-15 | 454884 | 61.275 |
| Body mass index (BMI) | rs1064213 | 2 | 198950240 | A | G | 0.478 | 0.015 | 0.002 | 6.59933e-14 | 454884 | 56.18 |
| Body mass index (BMI) | rs10742752 | 11 | 45438374 | C | T | 0.612 | 0.012 | 0.002 | 1e-08 | 454884 | 32.756 |
| Body mass index (BMI) | rs10756792 | 9 | 16726119 | T | C | 0.743 | -0.019 | 0.002 | 2.39994e-17 | 454884 | 71.76 |
| Body mass index (BMI) | rs10760277 | 9 | 126093999 | T | C | 0.385 | 0.014 | 0.002 | 7.89951e-12 | 454884 | 46.781 |
| Body mass index (BMI) | rs10771041 | 12 | 24058135 | T | C | 0.116 | 0.021 | 0.003 | 1.69981e-11 | 454884 | 45.282 |
| Body mass index (BMI) | rs10795162 | 10 | 4445117 | C | A | 0.076 | 0.018 | 0.004 | 1.79999e-06 | 454884 | 22.842 |
| Body mass index (BMI) | rs10827380 | 10 | 34822258 | T | C | 0.314 | 0.011 | 0.002 | 5e-07 | 454884 | 25.248 |
| Body mass index (BMI) | rs10852008 | 15 | 38119294 | T | C | 0.424 | 0.01 | 0.002 | 1.29999e-06 | 454884 | 23.482 |
| Body mass index (BMI) | rs10891549 | 11 | 113278447 | C | T | 0.55 | -0.01 | 0.002 | 2.1e-07 | 454884 | 26.943 |
| Body mass index (BMI) | rs10903791 | 10 | 2569491 | A | G | 0.604 | 0.011 | 0.002 | 4.49997e-08 | 454884 | 29.931 |
| Body mass index (BMI) | rs10907231 | 1 | 16851440 | T | C | 0.368 | -0.01 | 0.002 | 1.2e-06 | 454884 | 23.603 |
| Body mass index (BMI) | rs10915821 | 1 | 225584968 | C | T | 0.777 | -0.013 | 0.002 | 1.2e-07 | 454884 | 27.998 |
| Body mass index (BMI) | rs10927006 | 1 | 243557659 | C | T | 0.144 | -0.017 | 0.003 | 1.6e-09 | 454884 | 36.356 |
| Body mass index (BMI) | rs10938398 | 4 | 45186139 | A | G | 0.433 | 0.029 | 0.002 | 4.00037e-48 | 454884 | 212.479 |
| Body mass index (BMI) | rs10960294 | 9 | 11828586 | T | G | 0.334 | -0.014 | 0.002 | 4.79954e-11 | 454884 | 43.243 |
| Body mass index (BMI) | rs10965698 | 9 | 23203619 | T | C | 0.37 | -0.011 | 0.002 | 5.49997e-08 | 454884 | 29.528 |
| Body mass index (BMI) | rs10982030 | 9 | 116775849 | C | T | 0.055 | -0.023 | 0.004 | 2.39999e-07 | 454884 | 26.707 |
| Body mass index (BMI) | rs11000993 | 10 | 76084111 | C | T | 0.124 | 0.021 | 0.003 | 1.80011e-12 | 454884 | 49.641 |
| Body mass index (BMI) | rs11004108 | 10 | 55921514 | C | T | 0.262 | 0.011 | 0.002 | 8.50002e-07 | 454884 | 24.238 |
| Body mass index (BMI) | rs11006477 | 10 | 52420041 | A | G | 0.208 | 0.011 | 0.002 | 3.69999e-06 | 454884 | 21.392 |
| Body mass index (BMI) | rs11009685 | 10 | 34511990 | T | C | 0.244 | -0.013 | 0.002 | 6.59994e-08 | 454884 | 29.172 |
| Body mass index (BMI) | rs11012732 | 10 | 21830104 | G | A | 0.332 | 0.021 | 0.002 | 3.29989e-24 | 454884 | 103.033 |
| Body mass index (BMI) | rs11024271 | 11 | 17395540 | C | T | 0.623 | 0.012 | 0.002 | 2e-08 | 454884 | 31.517 |
| Body mass index (BMI) | rs11057072 | 12 | 16933340 | G | A | 0.234 | 0.013 | 0.002 | 2.5e-08 | 454884 | 31.052 |
| Body mass index (BMI) | rs1106761 | 8 | 142619234 | A | G | 0.384 | 0.015 | 0.002 | 1.69981e-12 | 454884 | 49.778 |
| Body mass index (BMI) | rs11071646 | 15 | 62273994 | A | G | 0.022 | -0.04 | 0.007 | 4.79999e-09 | 454884 | 34.254 |
| Body mass index (BMI) | rs11079849 | 17 | 47090785 | T | C | 0.329 | -0.02 | 0.002 | 6.29941e-22 | 454884 | 92.641 |
| Body mass index (BMI) | rs11097236 | 4 | 77028450 | A | G | 0.202 | 0.013 | 0.002 | 1.29999e-07 | 454884 | 27.915 |
| Body mass index (BMI) | rs11099020 | 4 | 130724902 | T | C | 0.641 | -0.014 | 0.002 | 4.90004e-12 | 454884 | 47.745 |
| Body mass index (BMI) | rs11103654 | 9 | 137943713 | A | G | 0.153 | 0.013 | 0.003 | 1.09999e-06 | 454884 | 23.756 |
| Body mass index (BMI) | rs11105846 | 12 | 91257268 | T | G | 0.368 | -0.011 | 0.002 | 4.90004e-08 | 454884 | 29.774 |
| Body mass index (BMI) | rs11115160 | 12 | 82424100 | A | G | 0.238 | -0.013 | 0.002 | 2.39999e-08 | 454884 | 31.138 |
| Body mass index (BMI) | rs11118890 | 1 | 222079647 | T | C | 0.291 | -0.011 | 0.002 | 1.5e-06 | 454884 | 23.174 |
| Body mass index (BMI) | rs11122450 | 1 | 230301811 | G | T | 0.612 | -0.012 | 0.002 | 1.40001e-08 | 454884 | 32.144 |
| Body mass index (BMI) | rs11134679 | 5 | 170623391 | G | A | 0.685 | 0.019 | 0.002 | 5.50047e-18 | 454884 | 74.678 |
| Body mass index (BMI) | rs11150745 | 17 | 78757626 | G | A | 0.318 | -0.021 | 0.002 | 2.29985e-22 | 454884 | 94.634 |
| Body mass index (BMI) | rs11150911 | 18 | 73498528 | C | A | 0.722 | -0.011 | 0.002 | 1.09999e-06 | 454884 | 23.741 |
| Body mass index (BMI) | rs11164968 | 1 | 94192472 | A | G | 0.974 | 0.029 | 0.006 | 2.99999e-06 | 454884 | 21.791 |
| Body mass index (BMI) | rs11165643 | 1 | 96924097 | T | C | 0.59 | 0.02 | 0.002 | 7.89951e-23 | 454884 | 96.734 |
| Body mass index (BMI) | rs112765062 | 14 | 35766611 | T | C | 0.093 | -0.017 | 0.003 | 1.2e-06 | 454884 | 23.625 |
| Body mass index (BMI) | rs113079574 | 4 | 147354089 | T | C | 0.193 | -0.016 | 0.003 | 2.19999e-10 | 454884 | 40.275 |
| Body mass index (BMI) | rs113603865 | 1 | 39564930 | T | C | 0.212 | 0.019 | 0.002 | 2.49977e-14 | 454884 | 58.109 |
| Body mass index (BMI) | rs113624107 | 14 | 88326386 | A | G | 0.226 | 0.014 | 0.002 | 1.5e-09 | 454884 | 36.553 |
| Body mass index (BMI) | rs114508011 | 1 | 231114420 | C | A | 0.074 | 0.018 | 0.004 | 2.5e-06 | 454884 | 22.192 |
| Body mass index (BMI) | rs114875897 | 1 | 96468325 | A | C | 0.019 | 0.036 | 0.007 | 5.80003e-07 | 454884 | 24.981 |
| Body mass index (BMI) | rs11525873 | 7 | 138817193 | C | T | 0.098 | -0.024 | 0.003 | 8.49963e-13 | 454884 | 51.168 |
| Body mass index (BMI) | rs11586758 | 1 | 38101457 | C | T | 0.068 | 0.019 | 0.004 | 2e-06 | 454884 | 22.612 |
| Body mass index (BMI) | rs11607586 | 11 | 122543130 | T | C | 0.258 | 0.011 | 0.002 | 6.49995e-07 | 454884 | 24.758 |
| Body mass index (BMI) | rs11608710 | 12 | 23753927 | G | T | 0.062 | 0.025 | 0.004 | 1.09999e-09 | 454884 | 37.089 |
| Body mass index (BMI) | rs116269521 | 4 | 6114677 | A | G | 0.034 | 0.026 | 0.006 | 3.50002e-06 | 454884 | 21.528 |
| Body mass index (BMI) | rs11636611 | 15 | 36391965 | T | C | 0.503 | 0.009 | 0.002 | 3.50002e-06 | 454884 | 21.497 |
| Body mass index (BMI) | rs116374395 | 5 | 50723410 | A | G | 0.035 | 0.032 | 0.005 | 3.59998e-09 | 454884 | 34.82 |
| Body mass index (BMI) | rs116377258 | 1 | 110131521 | G | A | 0.026 | 0.067 | 0.006 | 1.59993e-26 | 454884 | 113.566 |
| Body mass index (BMI) | rs11642090 | 16 | 81730582 | C | T | 0.373 | 0.011 | 0.002 | 7.39997e-08 | 454884 | 28.945 |
| Body mass index (BMI) | rs11660167 | 18 | 23543998 | A | G | 0.553 | 0.01 | 0.002 | 1e-06 | 454884 | 23.877 |
| Body mass index (BMI) | rs1167311 | 1 | 49996959 | A | G | 0.681 | -0.019 | 0.002 | 1.59993e-19 | 454884 | 81.631 |
| Body mass index (BMI) | rs11691869 | 2 | 100805996 | A | C | 0.362 | -0.02 | 0.002 | 1.9002e-21 | 454884 | 90.483 |
| Body mass index (BMI) | rs116952199 | 12 | 38904810 | C | A | 0.038 | -0.027 | 0.005 | 7.19996e-07 | 454884 | 24.572 |
| Body mass index (BMI) | rs11699828 | 20 | 62157198 | A | G | 0.036 | -0.035 | 0.006 | 2.90001e-09 | 454884 | 35.238 |
| Body mass index (BMI) | rs11709402 | 3 | 131551027 | G | A | 0.279 | 0.023 | 0.002 | 9.49948e-25 | 454884 | 105.506 |
| Body mass index (BMI) | rs11714189 | 3 | 70584077 | A | G | 0.204 | 0.014 | 0.002 | 5e-08 | 454884 | 29.711 |
| Body mass index (BMI) | rs117143749 | 8 | 47447107 | T | G | 0.032 | -0.029 | 0.006 | 4.49997e-07 | 454884 | 25.458 |
| Body mass index (BMI) | rs117342986 | 16 | 54267868 | T | C | 0.026 | 0.037 | 0.007 | 1.79999e-08 | 454884 | 31.646 |
| Body mass index (BMI) | rs117435593 | 20 | 36562529 | A | G | 0.045 | -0.026 | 0.005 | 1.40001e-07 | 454884 | 27.703 |
| Body mass index (BMI) | rs11751591 | 6 | 33794215 | A | G | 0.151 | -0.018 | 0.003 | 1.5e-10 | 454884 | 41.031 |
| Body mass index (BMI) | rs11757278 | 6 | 13180454 | C | T | 0.304 | -0.015 | 0.002 | 1e-11 | 454884 | 46.247 |
| Body mass index (BMI) | rs11773362 | 7 | 147668180 | T | C | 0.336 | -0.011 | 0.002 | 2e-07 | 454884 | 27.014 |
| Body mass index (BMI) | rs11782341 | 8 | 4813459 | G | A | 0.189 | 0.013 | 0.003 | 1e-06 | 454884 | 23.923 |
| Body mass index (BMI) | rs118136827 | 17 | 2168104 | T | G | 0.281 | -0.014 | 0.002 | 1.09999e-09 | 454884 | 37.174 |
| Body mass index (BMI) | rs11914525 | 3 | 85880768 | G | A | 0.354 | -0.018 | 0.002 | 3.69999e-18 | 454884 | 75.471 |
| Body mass index (BMI) | rs1191600 | 14 | 30101641 | A | C | 0.594 | -0.012 | 0.002 | 3.29997e-09 | 454884 | 35.022 |
| Body mass index (BMI) | rs1196657 | 2 | 150324097 | G | A | 0.796 | 0.012 | 0.002 | 1.5e-06 | 454884 | 23.182 |
| Body mass index (BMI) | rs12001437 | 9 | 34074476 | C | T | 0.368 | 0.012 | 0.002 | 2.19999e-09 | 454884 | 35.779 |
| Body mass index (BMI) | rs1205593 | 1 | 11252716 | C | T | 0.759 | -0.013 | 0.002 | 2.99999e-08 | 454884 | 30.694 |
| Body mass index (BMI) | rs12072739 | 1 | 98315893 | G | A | 0.225 | 0.016 | 0.002 | 1.20005e-11 | 454884 | 46.042 |
| Body mass index (BMI) | rs12089815 | 1 | 91189933 | A | G | 0.549 | -0.013 | 0.002 | 1.89998e-10 | 454884 | 40.534 |
| Body mass index (BMI) | rs12096864 | 1 | 209439141 | C | T | 0.119 | 0.017 | 0.003 | 6.90001e-08 | 454884 | 29.106 |
| Body mass index (BMI) | rs12140153 | 1 | 62579891 | T | G | 0.094 | -0.034 | 0.003 | 5.70033e-22 | 454884 | 92.812 |
| Body mass index (BMI) | rs1214478 | 8 | 60921427 | C | T | 0.452 | -0.01 | 0.002 | 2e-06 | 454884 | 22.641 |
| Body mass index (BMI) | rs12149660 | 16 | 70309237 | A | G | 0.115 | -0.022 | 0.003 | 1.59993e-12 | 454884 | 49.98 |
| Body mass index (BMI) | rs1217540 | 8 | 4289976 | C | A | 0.457 | 0.01 | 0.002 | 7.49998e-07 | 454884 | 24.487 |
| Body mass index (BMI) | rs12259464 | 10 | 53680099 | A | G | 0.485 | 0.013 | 0.002 | 5.70033e-11 | 454884 | 42.919 |
| Body mass index (BMI) | rs1226954 | 2 | 230172176 | C | T | 0.928 | -0.018 | 0.004 | 3.59998e-06 | 454884 | 21.474 |
| Body mass index (BMI) | rs1229984 | 4 | 100239319 | C | T | 0.973 | 0.039 | 0.006 | 1.09999e-10 | 454884 | 41.555 |
| Body mass index (BMI) | rs12310752 | 12 | 122282117 | G | A | 0.5 | -0.009 | 0.002 | 3.50002e-06 | 454884 | 21.535 |
| Body mass index (BMI) | rs12340969 | 9 | 15645713 | T | C | 0.441 | -0.021 | 0.002 | 3.80014e-26 | 454884 | 111.874 |
| Body mass index (BMI) | rs12364470 | 11 | 134601012 | G | T | 0.165 | 0.019 | 0.003 | 1.10002e-12 | 454884 | 50.67 |
| Body mass index (BMI) | rs12427047 | 12 | 90213070 | T | C | 0.243 | -0.017 | 0.002 | 1.9002e-13 | 454884 | 54.137 |
| Body mass index (BMI) | rs12462975 | 19 | 30272202 | A | G | 0.33 | 0.019 | 0.002 | 8.49963e-20 | 454884 | 82.918 |
| Body mass index (BMI) | rs12478299 | 2 | 193811641 | C | T | 0.252 | -0.011 | 0.002 | 5.89997e-07 | 454884 | 24.95 |
| Body mass index (BMI) | rs12633841 | 3 | 11630252 | T | G | 0.178 | 0.014 | 0.003 | 2.19999e-07 | 454884 | 26.83 |
| Body mass index (BMI) | rs1263629 | 2 | 207936911 | G | A | 0.144 | 0.017 | 0.003 | 1.89998e-09 | 454884 | 36.061 |
| Body mass index (BMI) | rs12659834 | 5 | 152061427 | A | G | 0.116 | 0.015 | 0.003 | 1.7e-06 | 454884 | 22.858 |
| Body mass index (BMI) | rs1266874 | 6 | 51779638 | G | A | 0.35 | 0.014 | 0.002 | 1.20005e-11 | 454884 | 45.946 |
| Body mass index (BMI) | rs12681792 | 8 | 62054463 | A | C | 0.193 | 0.015 | 0.003 | 4.39997e-09 | 454884 | 34.424 |
| Body mass index (BMI) | rs12691079 | 18 | 44712257 | G | T | 0.394 | -0.011 | 0.002 | 8.09991e-08 | 454884 | 28.786 |
| Body mass index (BMI) | rs12692596 | 2 | 161265910 | T | C | 0.372 | 0.014 | 0.002 | 1.9002e-11 | 454884 | 45.096 |
| Body mass index (BMI) | rs12694457 | 2 | 220134010 | C | T | 0.883 | 0.015 | 0.003 | 6.69993e-07 | 454884 | 24.709 |
| Body mass index (BMI) | rs12762034 | 10 | 33969931 | C | T | 0.077 | 0.028 | 0.004 | 1.29987e-13 | 454884 | 54.846 |
| Body mass index (BMI) | rs12776809 | 10 | 114834215 | A | G | 0.035 | 0.028 | 0.006 | 5.39995e-07 | 454884 | 25.116 |
| Body mass index (BMI) | rs12881629 | 14 | 101146413 | G | A | 0.083 | 0.022 | 0.004 | 8.30004e-10 | 454884 | 37.687 |
| Body mass index (BMI) | rs12885458 | 14 | 47303114 | G | T | 0.508 | -0.016 | 0.002 | 1.80011e-16 | 454884 | 67.827 |
| Body mass index (BMI) | rs12921986 | 16 | 72312727 | G | A | 0.078 | 0.019 | 0.004 | 2.69998e-07 | 454884 | 26.443 |
| Body mass index (BMI) | rs12956148 | 18 | 63429049 | A | C | 0.278 | 0.014 | 0.002 | 7.90005e-10 | 454884 | 37.786 |
| Body mass index (BMI) | rs1296328 | 4 | 137083193 | C | A | 0.559 | -0.018 | 0.002 | 2.09991e-19 | 454884 | 81.163 |
| Body mass index (BMI) | rs12977787 | 19 | 1814025 | A | G | 0.541 | 0.013 | 0.002 | 1.69981e-11 | 454884 | 45.247 |
| Body mass index (BMI) | rs13012070 | 2 | 35447243 | A | G | 0.228 | -0.014 | 0.002 | 1.79999e-09 | 454884 | 36.141 |
| Body mass index (BMI) | rs13033310 | 2 | 133523605 | A | G | 0.253 | 0.012 | 0.002 | 7.49998e-08 | 454884 | 28.929 |
| Body mass index (BMI) | rs13039964 | 20 | 21438589 | T | G | 0.097 | 0.016 | 0.003 | 2.59998e-06 | 454884 | 22.092 |
| Body mass index (BMI) | rs13041173 | 20 | 32542814 | G | A | 0.342 | 0.01 | 0.002 | 1.2e-06 | 454884 | 23.509 |
| Body mass index (BMI) | rs13077503 | 3 | 1215083 | A | C | 0.315 | 0.011 | 0.002 | 8.19993e-07 | 454884 | 24.305 |
| Body mass index (BMI) | rs13107325 | 4 | 103188709 | T | C | 0.075 | 0.047 | 0.004 | 5.40008e-36 | 454884 | 156.906 |
| Body mass index (BMI) | rs13163306 | 5 | 136571959 | A | G | 0.466 | -0.01 | 0.002 | 1.7e-07 | 454884 | 27.314 |
| Body mass index (BMI) | rs13176412 | 5 | 92646195 | A | G | 0.706 | -0.01 | 0.002 | 4.90004e-06 | 454884 | 20.887 |
| Body mass index (BMI) | rs13176429 | 5 | 43152216 | C | T | 0.688 | 0.015 | 0.002 | 6.09958e-12 | 454884 | 47.291 |
| Body mass index (BMI) | rs1320251 | 17 | 21264396 | T | C | 0.455 | -0.018 | 0.002 | 1.50003e-18 | 454884 | 77.245 |
| Body mass index (BMI) | rs1322842 | 6 | 20488897 | G | A | 0.609 | -0.013 | 0.002 | 1.09999e-10 | 454884 | 41.681 |
| Body mass index (BMI) | rs13248187 | 8 | 14336834 | C | T | 0.268 | 0.016 | 0.002 | 1.50003e-12 | 454884 | 50.078 |
| Body mass index (BMI) | rs1327259 | 6 | 51177811 | G | A | 0.388 | -0.015 | 0.002 | 1.50003e-13 | 454884 | 54.535 |
| Body mass index (BMI) | rs13290403 | 9 | 104411771 | C | T | 0.295 | -0.011 | 0.002 | 1e-06 | 454884 | 23.863 |
| Body mass index (BMI) | rs1330199 | 9 | 27760946 | T | G | 0.483 | -0.012 | 0.002 | 2.80001e-09 | 454884 | 35.334 |
| Body mass index (BMI) | rs1330828 | 9 | 85134671 | G | A | 0.32 | -0.011 | 0.002 | 3.50002e-07 | 454884 | 25.945 |
| Body mass index (BMI) | rs13427822 | 2 | 213414265 | G | A | 0.271 | -0.018 | 0.002 | 4.70002e-16 | 454884 | 65.938 |
| Body mass index (BMI) | rs1346841 | 4 | 65651730 | A | G | 0.405 | -0.013 | 0.002 | 2.90001e-10 | 454884 | 39.728 |
| Body mass index (BMI) | rs1360201 | 9 | 73796450 | T | C | 0.482 | 0.013 | 0.002 | 4.00037e-11 | 454884 | 43.623 |
| Body mass index (BMI) | rs1401430 | 8 | 116274942 | G | A | 0.417 | -0.01 | 0.002 | 2.59998e-06 | 454884 | 22.112 |
| Body mass index (BMI) | rs140159717 | 15 | 73765586 | T | C | 0.082 | -0.025 | 0.004 | 3.90032e-11 | 454884 | 43.668 |
| Body mass index (BMI) | rs1402599 | 6 | 44799657 | G | A | 0.728 | -0.011 | 0.002 | 1.29999e-06 | 454884 | 23.359 |
| Body mass index (BMI) | rs140733155 | 21 | 48048773 | G | A | 0.011 | 0.052 | 0.01 | 5.69994e-08 | 454884 | 29.449 |
| Body mass index (BMI) | rs140734681 | 12 | 57964348 | G | A | 0.014 | 0.041 | 0.009 | 2.90001e-06 | 454884 | 21.862 |
| Body mass index (BMI) | rs1430332 | 10 | 77423623 | T | C | 0.773 | 0.012 | 0.002 | 3.29997e-07 | 454884 | 26.087 |
| Body mass index (BMI) | rs143121872 | 14 | 47893217 | C | T | 0.023 | -0.037 | 0.007 | 2.69998e-07 | 454884 | 26.463 |
| Body mass index (BMI) | rs1436348 | 3 | 104612668 | G | A | 0.583 | 0.016 | 0.002 | 6.4998e-15 | 454884 | 60.756 |
| Body mass index (BMI) | rs1441264 | 13 | 79580919 | A | G | 0.594 | 0.018 | 0.002 | 3.69999e-18 | 454884 | 75.5 |
| Body mass index (BMI) | rs1445652 | 2 | 155668460 | A | G | 0.183 | 0.013 | 0.003 | 4.70002e-07 | 454884 | 25.383 |
| Body mass index (BMI) | rs1446585 | 2 | 136407479 | G | A | 0.245 | -0.014 | 0.002 | 9.69996e-10 | 454884 | 37.386 |
| Body mass index (BMI) | rs144702715 | 8 | 106633269 | C | A | 0.08 | -0.018 | 0.004 | 1.7e-06 | 454884 | 22.939 |
| Body mass index (BMI) | rs1451963 | 14 | 41350367 | T | G | 0.082 | 0.023 | 0.004 | 3.09999e-10 | 454884 | 39.622 |
| Body mass index (BMI) | rs1458156 | 12 | 41887940 | T | C | 0.488 | 0.014 | 0.002 | 3.10027e-12 | 454884 | 48.643 |
| Body mass index (BMI) | rs1459190 | 5 | 64070962 | A | G | 0.519 | -0.014 | 0.002 | 6.29941e-13 | 454884 | 51.755 |
| Body mass index (BMI) | rs1471093 | 3 | 108031094 | A | G | 0.617 | 0.013 | 0.002 | 5.40008e-11 | 454884 | 43.043 |
| Body mass index (BMI) | rs1472370 | 4 | 173485263 | T | C | 0.54 | 0.01 | 0.002 | 2.19999e-07 | 454884 | 26.85 |
| Body mass index (BMI) | rs147568678 | 10 | 93061851 | C | T | 0.238 | -0.014 | 0.002 | 1.40001e-09 | 454884 | 36.607 |
| Body mass index (BMI) | rs1477290 | 5 | 87988934 | C | T | 0.137 | 0.034 | 0.003 | 4.19952e-31 | 454884 | 134.5 |
| Body mass index (BMI) | rs147730268 | 12 | 123024476 | T | G | 0.087 | -0.035 | 0.004 | 9.79941e-22 | 454884 | 91.765 |
| Body mass index (BMI) | rs1482419 | 21 | 22117491 | G | A | 0.77 | -0.012 | 0.002 | 3.69999e-07 | 454884 | 25.851 |
| Body mass index (BMI) | rs148603272 | 3 | 51652257 | G | T | 0.016 | -0.039 | 0.008 | 1e-06 | 454884 | 23.931 |
| Body mass index (BMI) | rs149050217 | 7 | 71624556 | A | G | 0.181 | -0.016 | 0.003 | 3.69999e-10 | 454884 | 39.242 |
| Body mass index (BMI) | rs149064961 | 6 | 166401184 | A | C | 0.028 | 0.03 | 0.006 | 1.2e-06 | 454884 | 23.638 |
| Body mass index (BMI) | rs1492914 | 12 | 89930426 | C | T | 0.754 | 0.011 | 0.002 | 2.39999e-06 | 454884 | 22.248 |
| Body mass index (BMI) | rs1503526 | 5 | 63020706 | C | T | 0.48 | 0.016 | 0.002 | 5.30029e-15 | 454884 | 61.139 |
| Body mass index (BMI) | rs1504369 | 6 | 63894206 | A | C | 0.701 | -0.012 | 0.002 | 8.30004e-08 | 454884 | 28.738 |
| Body mass index (BMI) | rs1523751 | 3 | 77658497 | A | G | 0.503 | -0.011 | 0.002 | 8.70001e-08 | 454884 | 28.648 |
| Body mass index (BMI) | rs1540719 | 5 | 165768035 | T | C | 0.43 | -0.009 | 0.002 | 2.99999e-06 | 454884 | 21.8 |
| Body mass index (BMI) | rs1554654 | 3 | 44044344 | T | C | 0.471 | -0.013 | 0.002 | 1.6e-10 | 454884 | 40.932 |
| Body mass index (BMI) | rs1582931 | 5 | 122657199 | A | G | 0.473 | -0.013 | 0.002 | 1.6e-10 | 454884 | 40.872 |
| Body mass index (BMI) | rs1609010 | 8 | 77227464 | G | A | 0.566 | 0.022 | 0.002 | 7.10068e-27 | 454884 | 115.193 |
| Body mass index (BMI) | rs16916303 | 9 | 30823761 | G | A | 0.12 | -0.02 | 0.003 | 3.09999e-10 | 454884 | 39.611 |
| Body mass index (BMI) | rs16966801 | 17 | 39573713 | G | A | 0.197 | 0.015 | 0.003 | 6.69993e-09 | 454884 | 33.614 |
| Body mass index (BMI) | rs17005677 | 4 | 120674786 | C | T | 0.305 | 0.011 | 0.002 | 1.6e-07 | 454884 | 27.513 |
| Body mass index (BMI) | rs17056301 | 5 | 158271680 | C | T | 0.256 | 0.013 | 0.002 | 8e-09 | 454884 | 33.271 |
| Body mass index (BMI) | rs17193211 | 21 | 38885506 | T | C | 0.067 | -0.025 | 0.004 | 1.09999e-09 | 454884 | 37.087 |
| Body mass index (BMI) | rs17289010 | 4 | 140774684 | G | A | 0.328 | -0.014 | 0.002 | 1.7e-10 | 454884 | 40.736 |
| Body mass index (BMI) | rs17399739 | 10 | 87490850 | G | A | 0.069 | 0.028 | 0.004 | 1.99986e-12 | 454884 | 49.496 |
| Body mass index (BMI) | rs17639546 | 3 | 61251635 | A | G | 0.148 | -0.023 | 0.003 | 6.70039e-17 | 454884 | 69.76 |
| Body mass index (BMI) | rs17741830 | 18 | 42862127 | C | T | 0.08 | 0.019 | 0.004 | 1.5e-07 | 454884 | 27.597 |
| Body mass index (BMI) | rs1778830 | 1 | 156489974 | A | G | 0.362 | 0.014 | 0.002 | 8.80035e-12 | 454884 | 46.57 |
| Body mass index (BMI) | rs17818405 | 15 | 54624740 | A | G | 0.663 | -0.01 | 0.002 | 1.7e-06 | 454884 | 22.912 |
| Body mass index (BMI) | rs17867755 | 7 | 126719882 | A | G | 0.203 | 0.012 | 0.002 | 9.20005e-07 | 454884 | 24.086 |
| Body mass index (BMI) | rs1818917 | 15 | 23941678 | T | C | 0.513 | -0.01 | 0.002 | 2.30001e-07 | 454884 | 26.739 |
| Body mass index (BMI) | rs1834144 | 18 | 40744790 | A | C | 0.373 | -0.014 | 0.002 | 8.10028e-12 | 454884 | 46.744 |
| Body mass index (BMI) | rs1842803 | 3 | 65169961 | C | T | 0.407 | -0.01 | 0.002 | 7.90005e-07 | 454884 | 24.376 |
| Body mass index (BMI) | rs1884897 | 20 | 6612832 | G | A | 0.627 | 0.02 | 0.002 | 1.69981e-21 | 454884 | 90.724 |
| Body mass index (BMI) | rs189252432 | 16 | 70735620 | T | G | 0.012 | -0.047 | 0.009 | 1.89998e-07 | 454884 | 27.182 |
| Body mass index (BMI) | rs1919243 | 5 | 88778861 | C | T | 0.488 | 0.012 | 0.002 | 7.10003e-09 | 454884 | 33.515 |
| Body mass index (BMI) | rs1935002 | 6 | 82328332 | T | C | 0.282 | -0.011 | 0.002 | 1e-06 | 454884 | 23.863 |
| Body mass index (BMI) | rs1952346 | 9 | 16568780 | A | G | 0.066 | -0.02 | 0.004 | 1.09999e-06 | 454884 | 23.756 |
| Body mass index (BMI) | rs1999433 | 9 | 81371441 | T | C | 0.447 | -0.012 | 0.002 | 3.69999e-09 | 454884 | 34.768 |
| Body mass index (BMI) | rs2006906 | 20 | 12412378 | A | C | 0.545 | -0.01 | 0.002 | 2.5e-07 | 454884 | 26.631 |
| Body mass index (BMI) | rs2035936 | 3 | 141298124 | T | G | 0.056 | 0.036 | 0.004 | 9.3994e-17 | 454884 | 69.081 |
| Body mass index (BMI) | rs2051559 | 4 | 3298800 | C | T | 0.133 | 0.021 | 0.003 | 2.70023e-12 | 454884 | 48.898 |
| Body mass index (BMI) | rs207466 | 21 | 25199084 | T | G | 0.645 | -0.01 | 0.002 | 5.80003e-07 | 454884 | 24.984 |
| Body mass index (BMI) | rs2076603 | 1 | 17319011 | A | G | 0.644 | -0.012 | 0.002 | 2.80001e-09 | 454884 | 35.289 |
| Body mass index (BMI) | rs2102278 | 4 | 52818664 | G | A | 0.323 | 0.012 | 0.002 | 2.69998e-08 | 454884 | 30.888 |
| Body mass index (BMI) | rs2114210 | 8 | 95595162 | A | G | 0.336 | 0.014 | 0.002 | 7.10068e-11 | 454884 | 42.499 |
| Body mass index (BMI) | rs213518 | 7 | 26941065 | C | T | 0.146 | 0.016 | 0.003 | 8.40001e-09 | 454884 | 33.174 |
| Body mass index (BMI) | rs2153740 | 20 | 2126089 | G | A | 0.48 | -0.011 | 0.002 | 1.2e-08 | 454884 | 32.522 |
| Body mass index (BMI) | rs215634 | 7 | 32369148 | G | A | 0.612 | -0.015 | 0.002 | 4.10015e-14 | 454884 | 57.097 |
| Body mass index (BMI) | rs2164300 | 4 | 67813017 | T | C | 0.519 | -0.012 | 0.002 | 8.60003e-10 | 454884 | 37.61 |
| Body mass index (BMI) | rs2172131 | 10 | 133978962 | C | T | 0.579 | -0.015 | 0.002 | 6.70039e-14 | 454884 | 56.157 |
| Body mass index (BMI) | rs2181350 | 6 | 5976859 | G | A | 0.713 | 0.011 | 0.002 | 7.39997e-07 | 454884 | 24.513 |
| Body mass index (BMI) | rs2190887 | 7 | 39378580 | T | C | 0.561 | -0.011 | 0.002 | 4.09996e-08 | 454884 | 30.118 |
| Body mass index (BMI) | rs2192158 | 4 | 55505360 | G | A | 0.553 | -0.015 | 0.002 | 1.39991e-14 | 454884 | 59.185 |
| Body mass index (BMI) | rs2216931 | 2 | 181599070 | A | C | 0.662 | 0.017 | 0.002 | 2.39994e-15 | 454884 | 62.732 |
| Body mass index (BMI) | rs2234458 | 11 | 65639374 | T | C | 0.64 | -0.021 | 0.002 | 1.10002e-23 | 454884 | 100.735 |
| Body mass index (BMI) | rs2250557 | 14 | 72803788 | A | G | 0.29 | 0.01 | 0.002 | 1.89998e-06 | 454884 | 22.696 |
| Body mass index (BMI) | rs2250982 | 19 | 4945914 | C | A | 0.556 | 0.01 | 0.002 | 1.09999e-06 | 454884 | 23.813 |
| Body mass index (BMI) | rs2271189 | 12 | 56494991 | A | G | 0.403 | -0.016 | 0.002 | 2.60016e-15 | 454884 | 62.58 |
| Body mass index (BMI) | rs2275444 | 1 | 193051685 | A | G | 0.726 | -0.012 | 0.002 | 4.60002e-08 | 454884 | 29.868 |
| Body mass index (BMI) | rs227638 | 20 | 24918863 | C | T | 0.856 | 0.015 | 0.003 | 3.40001e-07 | 454884 | 25.988 |
| Body mass index (BMI) | rs2289379 | 7 | 44804225 | T | C | 0.396 | -0.015 | 0.002 | 1.80011e-13 | 454884 | 54.166 |
| Body mass index (BMI) | rs2306593 | 17 | 34866546 | T | C | 0.488 | -0.017 | 0.002 | 1.69981e-17 | 454884 | 72.43 |
| Body mass index (BMI) | rs2307111 | 5 | 75003678 | C | T | 0.395 | -0.028 | 0.002 | 1.80011e-42 | 454884 | 186.543 |
| Body mass index (BMI) | rs2342892 | 16 | 24540806 | G | T | 0.516 | -0.013 | 0.002 | 1.7e-10 | 454884 | 40.818 |
| Body mass index (BMI) | rs2372604 | 2 | 36820390 | T | G | 0.38 | 0.011 | 0.002 | 5.99998e-08 | 454884 | 29.371 |
| Body mass index (BMI) | rs2374174 | 6 | 68247189 | T | C | 0.217 | -0.011 | 0.002 | 4.49997e-06 | 454884 | 21.038 |
| Body mass index (BMI) | rs2376481 | 15 | 27220713 | A | G | 0.469 | 0.01 | 0.002 | 1.09999e-06 | 454884 | 23.683 |
| Body mass index (BMI) | rs2381404 | 2 | 144035442 | C | T | 0.244 | 0.014 | 0.002 | 5.49997e-09 | 454884 | 34.012 |
| Body mass index (BMI) | rs2398861 | 9 | 96430747 | G | A | 0.259 | 0.018 | 0.002 | 1.59993e-15 | 454884 | 63.494 |
| Body mass index (BMI) | rs2425816 | 20 | 44895075 | A | G | 0.415 | 0.012 | 0.002 | 7.90005e-10 | 454884 | 37.795 |
| Body mass index (BMI) | rs2433733 | 2 | 230816703 | A | G | 0.678 | -0.018 | 0.002 | 1e-16 | 454884 | 68.91 |
| Body mass index (BMI) | rs2482356 | 9 | 94178371 | C | T | 0.429 | -0.011 | 0.002 | 2.80001e-08 | 454884 | 30.823 |
| Body mass index (BMI) | rs2512892 | 11 | 131451862 | C | T | 0.566 | 0.013 | 0.002 | 5.50047e-11 | 454884 | 42.978 |
| Body mass index (BMI) | rs2525481 | 20 | 51726357 | C | T | 0.1 | -0.016 | 0.003 | 1.7e-06 | 454884 | 22.875 |
| Body mass index (BMI) | rs252761 | 5 | 77380723 | T | G | 0.588 | -0.011 | 0.002 | 2.69998e-08 | 454884 | 30.876 |
| Body mass index (BMI) | rs2568958 | 1 | 72765116 | A | G | 0.604 | 0.022 | 0.002 | 1.50003e-28 | 454884 | 122.829 |
| Body mass index (BMI) | rs2569993 | 3 | 12926096 | C | T | 0.32 | 0.013 | 0.002 | 1.2e-09 | 454884 | 36.986 |
| Body mass index (BMI) | rs2585526 | 13 | 78624080 | G | A | 0.557 | 0.01 | 0.002 | 5.39995e-07 | 454884 | 25.109 |
| Body mass index (BMI) | rs2601062 | 2 | 169274248 | T | G | 0.931 | -0.019 | 0.004 | 6.29999e-07 | 454884 | 24.816 |
| Body mass index (BMI) | rs2606228 | 3 | 183537759 | C | A | 0.646 | -0.013 | 0.002 | 1.5e-10 | 454884 | 41.059 |
| Body mass index (BMI) | rs2616143 | 8 | 20632022 | A | G | 0.32 | -0.014 | 0.002 | 1.6e-10 | 454884 | 40.957 |
| Body mass index (BMI) | rs262136 | 7 | 158764915 | T | C | 0.046 | -0.022 | 0.005 | 4.09996e-06 | 454884 | 21.217 |
| Body mass index (BMI) | rs2646355 | 4 | 55702141 | C | T | 0.449 | 0.01 | 0.002 | 5e-07 | 454884 | 25.251 |
| Body mass index (BMI) | rs2678204 | 1 | 201800511 | G | T | 0.34 | 0.024 | 0.002 | 8.99912e-31 | 454884 | 133.018 |
| Body mass index (BMI) | rs2725371 | 8 | 30854033 | G | A | 0.696 | -0.016 | 0.002 | 3.50026e-13 | 454884 | 52.894 |
| Body mass index (BMI) | rs2781668 | 6 | 131897278 | T | C | 0.166 | 0.015 | 0.003 | 3.2e-08 | 454884 | 30.57 |
| Body mass index (BMI) | rs2785610 | 1 | 93508182 | A | G | 0.203 | 0.012 | 0.002 | 9.20005e-07 | 454884 | 24.096 |
| Body mass index (BMI) | rs2836961 | 21 | 40627020 | C | A | 0.376 | 0.012 | 0.002 | 2.39999e-09 | 454884 | 35.617 |
| Body mass index (BMI) | rs28404639 | 5 | 80874229 | T | C | 0.366 | -0.012 | 0.002 | 5.80003e-09 | 454884 | 33.886 |
| Body mass index (BMI) | rs28454448 | 4 | 115125392 | G | A | 0.718 | -0.011 | 0.002 | 6.69993e-07 | 454884 | 24.687 |
| Body mass index (BMI) | rs28489620 | 22 | 41804716 | A | G | 0.29 | -0.015 | 0.002 | 7.70016e-12 | 454884 | 46.831 |
| Body mass index (BMI) | rs2861685 | 2 | 67837553 | C | T | 0.412 | -0.017 | 0.002 | 4.10015e-17 | 454884 | 70.713 |
| Body mass index (BMI) | rs2864700 | 1 | 151028929 | T | C | 0.591 | -0.01 | 0.002 | 2.30001e-07 | 454884 | 26.731 |
| Body mass index (BMI) | rs28670671 | 9 | 140363045 | C | T | 0.286 | -0.013 | 0.002 | 4e-08 | 454884 | 30.136 |
| Body mass index (BMI) | rs28678108 | 4 | 94990661 | G | A | 0.448 | -0.01 | 0.002 | 4.49997e-07 | 454884 | 25.449 |
| Body mass index (BMI) | rs28732167 | 6 | 32031904 | A | G | 0.048 | 0.026 | 0.005 | 1.29999e-08 | 454884 | 32.273 |
| Body mass index (BMI) | rs2917705 | 16 | 64339094 | A | G | 0.141 | -0.014 | 0.003 | 1.40001e-06 | 454884 | 23.226 |
| Body mass index (BMI) | rs2919389 | 8 | 32588901 | T | C | 0.401 | 0.011 | 0.002 | 2.69998e-07 | 454884 | 26.488 |
| Body mass index (BMI) | rs2920503 | 3 | 12324230 | T | C | 0.285 | -0.014 | 0.002 | 1.40001e-10 | 454884 | 41.126 |
| Body mass index (BMI) | rs2962334 | 5 | 86879056 | T | G | 0.02 | 0.043 | 0.007 | 8.40001e-10 | 454884 | 37.654 |
| Body mass index (BMI) | rs2973564 | 5 | 73151033 | G | A | 0.3 | -0.011 | 0.002 | 3.59998e-07 | 454884 | 25.882 |
| Body mass index (BMI) | rs2975726 | 8 | 10094377 | G | A | 0.15 | 0.014 | 0.003 | 5.39995e-07 | 454884 | 25.127 |
| Body mass index (BMI) | rs308632 | 13 | 62742983 | T | C | 0.254 | -0.012 | 0.002 | 2.1e-07 | 454884 | 26.948 |
| Body mass index (BMI) | rs329118 | 5 | 133861663 | T | C | 0.419 | -0.017 | 0.002 | 1e-16 | 454884 | 68.913 |
| Body mass index (BMI) | rs329651 | 11 | 133767622 | T | G | 0.804 | 0.016 | 0.003 | 4.79999e-10 | 454884 | 38.748 |
| Body mass index (BMI) | rs34025316 | 7 | 137405295 | T | C | 0.338 | 0.012 | 0.002 | 2.59998e-08 | 454884 | 30.985 |
| Body mass index (BMI) | rs34045288 | 6 | 40369081 | T | C | 0.334 | 0.024 | 0.002 | 6.90081e-29 | 454884 | 124.408 |
| Body mass index (BMI) | rs34153025 | 15 | 41339697 | C | T | 0.022 | -0.038 | 0.007 | 1.79999e-08 | 454884 | 31.687 |
| Body mass index (BMI) | rs34231005 | 1 | 4594998 | G | T | 0.261 | -0.011 | 0.002 | 2.39999e-06 | 454884 | 22.207 |
| Body mass index (BMI) | rs34234296 | 2 | 175166636 | A | G | 0.392 | -0.015 | 0.002 | 2.80027e-13 | 454884 | 53.315 |
| Body mass index (BMI) | rs34481751 | 20 | 47501038 | A | C | 0.165 | -0.019 | 0.003 | 2.19989e-12 | 454884 | 49.332 |
| Body mass index (BMI) | rs34517439 | 1 | 78450517 | A | C | 0.122 | 0.038 | 0.003 | 1.29987e-35 | 454884 | 155.103 |
| Body mass index (BMI) | rs34573636 | 1 | 34302673 | C | A | 0.477 | -0.01 | 0.002 | 5.30005e-07 | 454884 | 25.139 |
| Body mass index (BMI) | rs34811474 | 4 | 25408838 | A | G | 0.231 | -0.028 | 0.002 | 3.29989e-33 | 454884 | 144.16 |
| Body mass index (BMI) | rs349071 | 11 | 84776849 | A | G | 0.5 | -0.013 | 0.002 | 9.20026e-11 | 454884 | 41.98 |
| Body mass index (BMI) | rs34921009 | 2 | 29099285 | C | T | 0.462 | -0.01 | 0.002 | 4.90004e-07 | 454884 | 25.302 |
| Body mass index (BMI) | rs35154326 | 16 | 24862414 | G | A | 0.274 | -0.013 | 0.002 | 2.80001e-09 | 454884 | 35.305 |
| Body mass index (BMI) | rs35364449 | 15 | 74278126 | T | C | 0.11 | 0.022 | 0.003 | 6.70039e-12 | 454884 | 47.12 |
| Body mass index (BMI) | rs35408866 | 4 | 187743245 | A | G | 0.136 | 0.014 | 0.003 | 9.20005e-07 | 454884 | 24.085 |
| Body mass index (BMI) | rs355393 | 15 | 46592396 | G | A | 0.47 | -0.012 | 0.002 | 8.50002e-09 | 454884 | 33.164 |
| Body mass index (BMI) | rs35746264 | 8 | 28062691 | G | A | 0.455 | -0.011 | 0.002 | 1.2e-07 | 454884 | 28.072 |
| Body mass index (BMI) | rs35809007 | 2 | 47019521 | A | G | 0.363 | -0.017 | 0.002 | 4.10015e-17 | 454884 | 70.74 |
| Body mass index (BMI) | rs35867081 | 17 | 79047278 | G | A | 0.512 | -0.015 | 0.002 | 1.99986e-13 | 454884 | 53.979 |
| Body mass index (BMI) | rs35957544 | 8 | 73440371 | T | G | 0.574 | -0.02 | 0.002 | 1.9002e-22 | 454884 | 94.988 |
| Body mass index (BMI) | rs35972789 | 10 | 75519691 | A | C | 0.037 | -0.029 | 0.005 | 2.90001e-08 | 454884 | 30.766 |
| Body mass index (BMI) | rs36007635 | 6 | 163009335 | A | G | 0.138 | -0.021 | 0.003 | 1.20005e-12 | 454884 | 50.431 |
| Body mass index (BMI) | rs36061954 | 8 | 38329650 | T | C | 0.399 | 0.013 | 0.002 | 2.5e-10 | 454884 | 40.052 |
| Body mass index (BMI) | rs3730071 | 12 | 49168798 | A | C | 0.03 | -0.029 | 0.006 | 8.40001e-07 | 454884 | 24.265 |
| Body mass index (BMI) | rs3751837 | 16 | 3583173 | T | C | 0.219 | 0.014 | 0.002 | 3.50002e-09 | 454884 | 34.88 |
| Body mass index (BMI) | rs3764625 | 19 | 49649051 | G | T | 0.588 | -0.011 | 0.002 | 1.5e-08 | 454884 | 32.069 |
| Body mass index (BMI) | rs3766823 | 1 | 32197257 | A | G | 0.172 | 0.016 | 0.003 | 7.59994e-10 | 454884 | 37.856 |
| Body mass index (BMI) | rs3784710 | 15 | 68072458 | C | T | 0.227 | -0.03 | 0.002 | 2.29985e-35 | 454884 | 153.993 |
| Body mass index (BMI) | rs3803286 | 14 | 103246470 | G | A | 0.667 | -0.018 | 0.002 | 3.10027e-18 | 454884 | 75.855 |
| Body mass index (BMI) | rs3807566 | 7 | 50564204 | T | G | 0.438 | -0.013 | 0.002 | 4.30002e-10 | 454884 | 38.98 |
| Body mass index (BMI) | rs3814883 | 16 | 29994922 | T | C | 0.482 | 0.024 | 0.002 | 2.49977e-33 | 454884 | 144.733 |
| Body mass index (BMI) | rs3848453 | 17 | 68362094 | C | T | 0.584 | -0.011 | 0.002 | 2e-07 | 454884 | 27.048 |
| Body mass index (BMI) | rs3866805 | 1 | 6657424 | A | C | 0.356 | 0.012 | 0.002 | 1.2e-08 | 454884 | 32.425 |
| Body mass index (BMI) | rs3897102 | 12 | 123492112 | T | C | 0.411 | 0.012 | 0.002 | 1.89998e-09 | 454884 | 36.082 |
| Body mass index (BMI) | rs3901286 | 7 | 99107727 | A | C | 0.152 | -0.023 | 0.003 | 3.69999e-16 | 454884 | 66.398 |
| Body mass index (BMI) | rs394608 | 21 | 46581798 | C | T | 0.538 | 0.019 | 0.002 | 2.29985e-20 | 454884 | 85.478 |
| Body mass index (BMI) | rs40071 | 5 | 107496102 | C | T | 0.179 | -0.026 | 0.003 | 1.29987e-23 | 454884 | 100.276 |
| Body mass index (BMI) | rs4055791 | 13 | 59266053 | T | C | 0.417 | -0.018 | 0.002 | 4.40048e-19 | 454884 | 79.695 |
| Body mass index (BMI) | rs40631 | 5 | 66222364 | T | G | 0.61 | 0.01 | 0.002 | 1.2e-06 | 454884 | 23.571 |
| Body mass index (BMI) | rs4148155 | 4 | 89054667 | G | A | 0.113 | -0.022 | 0.003 | 7.8001e-13 | 454884 | 51.327 |
| Body mass index (BMI) | rs4238749 | 16 | 52639234 | A | C | 0.412 | 0.01 | 0.002 | 2.19999e-07 | 454884 | 26.878 |
| Body mass index (BMI) | rs4261944 | 4 | 31003636 | G | T | 0.365 | 0.014 | 0.002 | 7.89951e-12 | 454884 | 46.791 |
| Body mass index (BMI) | rs4284600 | 15 | 31843528 | C | T | 0.467 | 0.012 | 0.002 | 1.29999e-09 | 454884 | 36.838 |
| Body mass index (BMI) | rs429343 | 2 | 147903382 | G | A | 0.577 | -0.017 | 0.002 | 8.4004e-18 | 454884 | 73.851 |
| Body mass index (BMI) | rs429358 | 19 | 45411941 | C | T | 0.154 | -0.027 | 0.003 | 5.90065e-22 | 454884 | 92.753 |
| Body mass index (BMI) | rs42999 | 7 | 90752135 | T | C | 0.826 | 0.013 | 0.003 | 2.39999e-06 | 454884 | 22.274 |
| Body mass index (BMI) | rs4307239 | 7 | 24354300 | G | A | 0.459 | 0.012 | 0.002 | 4.60002e-10 | 454884 | 38.819 |
| Body mass index (BMI) | rs4337209 | 14 | 27541848 | T | C | 0.642 | 0.01 | 0.002 | 1.79999e-06 | 454884 | 22.787 |
| Body mass index (BMI) | rs4388516 | 9 | 124631301 | G | A | 0.558 | -0.01 | 0.002 | 4.39997e-07 | 454884 | 25.518 |
| Body mass index (BMI) | rs4397962 | 13 | 28687084 | C | T | 0.221 | 0.013 | 0.002 | 1.5e-07 | 454884 | 27.546 |
| Body mass index (BMI) | rs4456769 | 20 | 25190777 | T | C | 0.333 | 0.015 | 0.002 | 3.80014e-12 | 454884 | 48.217 |
| Body mass index (BMI) | rs4477562 | 13 | 54104968 | T | C | 0.129 | 0.03 | 0.003 | 3.69999e-23 | 454884 | 98.253 |
| Body mass index (BMI) | rs4482463 | 2 | 205375909 | A | C | 0.923 | -0.031 | 0.004 | 1.39991e-16 | 454884 | 68.307 |
| Body mass index (BMI) | rs4524456 | 4 | 6492739 | A | G | 0.525 | -0.01 | 0.002 | 1.6e-07 | 454884 | 27.408 |
| Body mass index (BMI) | rs4562174 | 6 | 114213952 | A | G | 0.487 | 0.009 | 0.002 | 2.69998e-06 | 454884 | 22.018 |
| Body mass index (BMI) | rs4613074 | 16 | 19942527 | C | T | 0.185 | -0.024 | 0.003 | 3.69999e-20 | 454884 | 84.556 |
| Body mass index (BMI) | rs4625852 | 2 | 236762851 | A | G | 0.205 | -0.014 | 0.002 | 2e-08 | 454884 | 31.476 |
| Body mass index (BMI) | rs4625888 | 2 | 204101157 | T | C | 0.558 | 0.012 | 0.002 | 4e-09 | 454884 | 34.614 |
| Body mass index (BMI) | rs4648450 | 1 | 2723214 | A | C | 0.467 | -0.015 | 0.002 | 1.29987e-13 | 454884 | 54.838 |
| Body mass index (BMI) | rs4658403 | 1 | 243832560 | T | C | 0.834 | -0.019 | 0.003 | 2.70023e-12 | 454884 | 48.881 |
| Body mass index (BMI) | rs4672338 | 2 | 60217457 | T | C | 0.336 | 0.013 | 0.002 | 2.39999e-10 | 454884 | 40.095 |
| Body mass index (BMI) | rs4700780 | 5 | 178503640 | T | C | 0.299 | 0.011 | 0.002 | 2.80001e-07 | 454884 | 26.386 |
| Body mass index (BMI) | rs4718964 | 7 | 70038969 | T | G | 0.413 | 0.011 | 0.002 | 2.59998e-08 | 454884 | 30.998 |
| Body mass index (BMI) | rs4722398 | 7 | 3125220 | T | C | 0.136 | 0.019 | 0.003 | 1.40001e-10 | 454884 | 41.201 |
| Body mass index (BMI) | rs472502 | 3 | 62706298 | A | G | 0.213 | 0.013 | 0.002 | 1.09999e-07 | 454884 | 28.23 |
| Body mass index (BMI) | rs4749937 | 10 | 10255003 | G | T | 0.761 | -0.013 | 0.002 | 7.29995e-08 | 454884 | 28.977 |
| Body mass index (BMI) | rs4757144 | 11 | 13331226 | A | G | 0.59 | 0.016 | 0.002 | 1.50003e-15 | 454884 | 63.612 |
| Body mass index (BMI) | rs4764949 | 12 | 103658096 | G | A | 0.326 | -0.018 | 0.002 | 3.10027e-17 | 454884 | 71.292 |
| Body mass index (BMI) | rs4790292 | 17 | 1824305 | A | C | 0.154 | -0.026 | 0.003 | 6.90081e-21 | 454884 | 87.891 |
| Body mass index (BMI) | rs4800756 | 18 | 24247450 | A | G | 0.647 | -0.011 | 0.002 | 8e-08 | 454884 | 28.807 |
| Body mass index (BMI) | rs4832298 | 2 | 86764004 | T | C | 0.686 | -0.016 | 0.002 | 8.19974e-14 | 454884 | 55.75 |
| Body mass index (BMI) | rs484455 | 13 | 109835686 | A | G | 0.481 | -0.01 | 0.002 | 4.09996e-07 | 454884 | 25.65 |
| Body mass index (BMI) | rs4865796 | 5 | 53272664 | A | G | 0.693 | -0.01 | 0.002 | 2.99999e-06 | 454884 | 21.836 |
| Body mass index (BMI) | rs4876611 | 8 | 116671848 | G | A | 0.72 | 0.019 | 0.002 | 3.50026e-18 | 454884 | 75.564 |
| Body mass index (BMI) | rs4877148 | 9 | 80857307 | G | T | 0.548 | 0.01 | 0.002 | 1.2e-06 | 454884 | 23.522 |
| Body mass index (BMI) | rs4895799 | 6 | 126052729 | T | C | 0.585 | 0.012 | 0.002 | 1.09999e-08 | 454884 | 32.62 |
| Body mass index (BMI) | rs4900590 | 14 | 104146421 | T | C | 0.324 | 0.015 | 0.002 | 5.60015e-12 | 454884 | 47.482 |
| Body mass index (BMI) | rs4919197 | 10 | 100043147 | T | C | 0.474 | 0.011 | 0.002 | 4.79999e-08 | 454884 | 29.791 |
| Body mass index (BMI) | rs4929923 | 11 | 8639200 | C | T | 0.645 | 0.019 | 0.002 | 3.40017e-20 | 454884 | 84.728 |
| Body mass index (BMI) | rs4937729 | 11 | 132703585 | C | A | 0.488 | 0.01 | 0.002 | 1e-06 | 454884 | 23.902 |
| Body mass index (BMI) | rs4944769 | 11 | 87457254 | A | G | 0.282 | 0.011 | 0.002 | 1.29999e-06 | 454884 | 23.412 |
| Body mass index (BMI) | rs4947461 | 7 | 54410243 | C | T | 0.538 | -0.01 | 0.002 | 5.69994e-07 | 454884 | 25.005 |
| Body mass index (BMI) | rs4976553 | 5 | 167867953 | G | A | 0.193 | -0.014 | 0.003 | 4.39997e-08 | 454884 | 29.95 |
| Body mass index (BMI) | rs4984291 | 15 | 63777614 | G | A | 0.541 | 0.01 | 0.002 | 8.09991e-07 | 454884 | 24.338 |
| Body mass index (BMI) | rs512121 | 18 | 7548501 | C | T | 0.192 | -0.016 | 0.003 | 6.69993e-10 | 454884 | 38.1 |
| Body mass index (BMI) | rs529200 | 3 | 173114305 | G | A | 0.528 | 0.017 | 0.002 | 5.79963e-18 | 454884 | 74.579 |
| Body mass index (BMI) | rs539515 | 1 | 177889025 | C | A | 0.205 | 0.05 | 0.002 | 7.00003e-91 | 454884 | 408.699 |
| Body mass index (BMI) | rs55658481 | 2 | 219284215 | A | G | 0.339 | 0.013 | 0.002 | 1.79999e-10 | 454884 | 40.632 |
| Body mass index (BMI) | rs55707359 | 11 | 46159333 | G | T | 0.015 | 0.053 | 0.008 | 1.09999e-10 | 454884 | 41.717 |
| Body mass index (BMI) | rs55714539 | 19 | 18207397 | C | A | 0.344 | 0.018 | 0.002 | 3.50026e-17 | 454884 | 71.05 |
| Body mass index (BMI) | rs55726687 | 12 | 991306 | A | G | 0.21 | 0.025 | 0.002 | 2.70023e-24 | 454884 | 103.395 |
| Body mass index (BMI) | rs558882 | 3 | 118151652 | C | A | 0.217 | 0.013 | 0.002 | 1.2e-07 | 454884 | 28.074 |
| Body mass index (BMI) | rs558887 | 11 | 28712741 | G | A | 0.308 | -0.013 | 0.002 | 8e-10 | 454884 | 37.753 |
| Body mass index (BMI) | rs559231 | 18 | 39644247 | T | G | 0.393 | 0.013 | 0.002 | 3.2e-10 | 454884 | 39.529 |
| Body mass index (BMI) | rs56038322 | 3 | 69925128 | A | G | 0.311 | 0.014 | 0.002 | 6.79986e-11 | 454884 | 42.588 |
| Body mass index (BMI) | rs56094641 | 16 | 53806453 | G | A | 0.405 | 0.073 | 0.002 | 1e-200 | 454884 | 1306.491 |
| Body mass index (BMI) | rs56104747 | 2 | 12816195 | A | G | 0.193 | 0.013 | 0.003 | 5.39995e-07 | 454884 | 25.122 |
| Body mass index (BMI) | rs56133507 | 2 | 172818467 | G | T | 0.197 | 0.014 | 0.002 | 1.40001e-08 | 454884 | 32.252 |
| Body mass index (BMI) | rs56143236 | 3 | 157020444 | T | C | 0.256 | 0.012 | 0.002 | 2e-07 | 454884 | 27.009 |
| Body mass index (BMI) | rs56203622 | 9 | 131040874 | C | T | 0.146 | 0.018 | 0.003 | 2e-10 | 454884 | 40.461 |
| Body mass index (BMI) | rs56211164 | 7 | 158016764 | A | G | 0.24 | -0.013 | 0.002 | 6.80002e-08 | 454884 | 29.131 |
| Body mass index (BMI) | rs56295507 | 16 | 49731986 | A | G | 0.219 | -0.012 | 0.002 | 1.09999e-06 | 454884 | 23.8 |
| Body mass index (BMI) | rs56352336 | 19 | 19352155 | C | T | 0.155 | -0.017 | 0.003 | 1.79999e-09 | 454884 | 36.217 |
| Body mass index (BMI) | rs56356382 | 19 | 4064057 | C | T | 0.192 | -0.023 | 0.003 | 6.70039e-19 | 454884 | 78.86 |
| Body mass index (BMI) | rs56399737 | 13 | 33381721 | T | C | 0.449 | -0.016 | 0.002 | 3.59998e-15 | 454884 | 61.921 |
| Body mass index (BMI) | rs56803094 | 15 | 99222509 | G | A | 0.227 | -0.015 | 0.002 | 5e-10 | 454884 | 38.673 |
| Body mass index (BMI) | rs56858768 | 13 | 86511730 | A | G | 0.297 | 0.016 | 0.002 | 5.60015e-13 | 454884 | 51.995 |
| Body mass index (BMI) | rs56893062 | 8 | 25662655 | G | T | 0.303 | 0.013 | 0.002 | 2.39999e-09 | 454884 | 35.582 |
| Body mass index (BMI) | rs57263785 | 9 | 2197987 | G | A | 0.246 | -0.011 | 0.002 | 1.6e-06 | 454884 | 23.032 |
| Body mass index (BMI) | rs57488047 | 15 | 79403002 | C | T | 0.468 | -0.015 | 0.002 | 4.49987e-14 | 454884 | 56.948 |
| Body mass index (BMI) | rs57636386 | 18 | 58048295 | C | T | 0.084 | -0.041 | 0.004 | 1e-29 | 454884 | 128.189 |
| Body mass index (BMI) | rs577525 | 10 | 99769388 | C | T | 0.562 | 0.019 | 0.002 | 3.69999e-22 | 454884 | 93.658 |
| Body mass index (BMI) | rs57989773 | 6 | 100629078 | C | T | 0.245 | 0.013 | 0.002 | 1.6e-08 | 454884 | 31.945 |
| Body mass index (BMI) | rs587271 | 1 | 54743111 | T | C | 0.687 | 0.012 | 0.002 | 1e-07 | 454884 | 28.329 |
| Body mass index (BMI) | rs58862095 | 7 | 75081418 | T | C | 0.419 | -0.023 | 0.002 | 4.90004e-29 | 454884 | 125.07 |
| Body mass index (BMI) | rs59227842 | 11 | 43692423 | G | A | 0.311 | 0.023 | 0.002 | 1.9002e-26 | 454884 | 113.21 |
| Body mass index (BMI) | rs59237168 | 17 | 31479035 | C | T | 0.216 | -0.016 | 0.002 | 8.19974e-11 | 454884 | 42.215 |
| Body mass index (BMI) | rs5995843 | 22 | 40697377 | G | A | 0.346 | -0.018 | 0.002 | 5.00035e-17 | 454884 | 70.328 |
| Body mass index (BMI) | rs6000329 | 22 | 36959219 | A | G | 0.56 | -0.011 | 0.002 | 9.80009e-08 | 454884 | 28.414 |
| Body mass index (BMI) | rs6023655 | 20 | 53479658 | G | A | 0.766 | -0.014 | 0.002 | 1.7e-09 | 454884 | 36.299 |
| Body mass index (BMI) | rs60764613 | 18 | 1839911 | T | G | 0.145 | 0.02 | 0.003 | 1.59993e-12 | 454884 | 49.965 |
| Body mass index (BMI) | rs6092194 | 20 | 54423296 | G | T | 0.556 | 0.01 | 0.002 | 2e-07 | 454884 | 27.03 |
| Body mass index (BMI) | rs6134650 | 20 | 12708516 | G | A | 0.285 | 0.01 | 0.002 | 3.29997e-06 | 454884 | 21.643 |
| Body mass index (BMI) | rs6134916 | 20 | 1409757 | T | C | 0.493 | -0.011 | 0.002 | 4.60002e-08 | 454884 | 29.885 |
| Body mass index (BMI) | rs61813324 | 1 | 156049877 | T | C | 0.136 | 0.029 | 0.003 | 6.20012e-23 | 454884 | 97.22 |
| Body mass index (BMI) | rs61828088 | 1 | 174768522 | A | G | 0.11 | 0.022 | 0.003 | 2.90001e-12 | 454884 | 48.76 |
| Body mass index (BMI) | rs61901763 | 11 | 76942242 | G | A | 0.122 | -0.015 | 0.003 | 1.09999e-06 | 454884 | 23.796 |
| Body mass index (BMI) | rs61903695 | 11 | 89922417 | G | A | 0.255 | 0.017 | 0.002 | 2.70023e-13 | 454884 | 53.438 |
| Body mass index (BMI) | rs61992671 | 14 | 101531854 | G | A | 0.492 | -0.016 | 0.002 | 1.20005e-14 | 454884 | 59.475 |
| Body mass index (BMI) | rs62036155 | 16 | 4958961 | C | A | 0.031 | 0.027 | 0.006 | 2.99999e-06 | 454884 | 21.824 |
| Body mass index (BMI) | rs62058023 | 17 | 55336891 | C | T | 0.134 | 0.015 | 0.003 | 1.79999e-07 | 454884 | 27.276 |
| Body mass index (BMI) | rs62107261 | 2 | 422144 | C | T | 0.048 | -0.092 | 0.005 | 1.69981e-86 | 454884 | 388.512 |
| Body mass index (BMI) | rs62120278 | 2 | 10981461 | T | C | 0.139 | 0.015 | 0.003 | 1.09999e-07 | 454884 | 28.19 |
| Body mass index (BMI) | rs62131064 | 2 | 25568528 | A | C | 0.099 | -0.016 | 0.003 | 1.40001e-06 | 454884 | 23.298 |
| Body mass index (BMI) | rs62161461 | 2 | 137522814 | T | G | 0.47 | 0.009 | 0.002 | 2.5e-06 | 454884 | 22.191 |
| Body mass index (BMI) | rs62240473 | 22 | 18138246 | G | A | 0.039 | -0.026 | 0.005 | 5e-07 | 454884 | 25.253 |
| Body mass index (BMI) | rs62241847 | 3 | 20466465 | G | A | 0.315 | -0.012 | 0.002 | 5.80003e-09 | 454884 | 33.887 |
| Body mass index (BMI) | rs62246311 | 3 | 9498143 | A | G | 0.102 | 0.021 | 0.003 | 2.90001e-10 | 454884 | 39.735 |
| Body mass index (BMI) | rs62299452 | 4 | 55221578 | G | A | 0.506 | 0.01 | 0.002 | 1.2e-06 | 454884 | 23.503 |
| Body mass index (BMI) | rs62379271 | 5 | 105870033 | G | T | 0.578 | 0.012 | 0.002 | 7.29995e-09 | 454884 | 33.46 |
| Body mass index (BMI) | rs62509490 | 8 | 106004770 | G | A | 0.064 | -0.022 | 0.004 | 1e-07 | 454884 | 28.362 |
| Body mass index (BMI) | rs62568207 | 9 | 81885878 | C | T | 0.084 | -0.019 | 0.004 | 1.29999e-07 | 454884 | 27.816 |
| Body mass index (BMI) | rs6265 | 11 | 27679916 | T | C | 0.188 | -0.04 | 0.003 | 5.90065e-56 | 454884 | 248.355 |
| Body mass index (BMI) | rs6430068 | 2 | 145627927 | A | G | 0.109 | 0.019 | 0.003 | 5.60003e-09 | 454884 | 33.98 |
| Body mass index (BMI) | rs6444950 | 3 | 170602073 | A | G | 0.238 | 0.016 | 0.002 | 3.90032e-12 | 454884 | 48.177 |
| Body mass index (BMI) | rs6456288 | 6 | 20023589 | A | C | 0.39 | 0.01 | 0.002 | 2.90001e-06 | 454884 | 21.893 |
| Body mass index (BMI) | rs6474856 | 9 | 14768160 | T | C | 0.64 | -0.012 | 0.002 | 7.79992e-09 | 454884 | 33.313 |
| Body mass index (BMI) | rs6531639 | 4 | 38556399 | A | G | 0.248 | -0.014 | 0.002 | 2.99999e-09 | 454884 | 35.216 |
| Body mass index (BMI) | rs6544001 | 2 | 36494107 | A | C | 0.075 | 0.02 | 0.004 | 9.29994e-08 | 454884 | 28.512 |
| Body mass index (BMI) | rs6545714 | 2 | 59307725 | A | G | 0.601 | -0.021 | 0.002 | 2.49977e-24 | 454884 | 103.603 |
| Body mass index (BMI) | rs6551304 | 3 | 88306596 | G | A | 0.832 | 0.018 | 0.003 | 4.40048e-11 | 454884 | 43.414 |
| Body mass index (BMI) | rs6560906 | 12 | 133414054 | C | T | 0.692 | -0.012 | 0.002 | 1.7e-08 | 454884 | 31.769 |
| Body mass index (BMI) | rs6567160 | 18 | 57829135 | C | T | 0.233 | 0.054 | 0.002 | 2.50035e-114 | 454884 | 516.419 |
| Body mass index (BMI) | rs6574136 | 14 | 74145025 | T | C | 0.79 | 0.011 | 0.002 | 4.20001e-06 | 454884 | 21.189 |
| Body mass index (BMI) | rs6575340 | 14 | 94023972 | A | G | 0.636 | 0.021 | 0.002 | 9.09913e-24 | 454884 | 101.023 |
| Body mass index (BMI) | rs6606580 | 9 | 140278903 | G | A | 0.589 | -0.011 | 0.002 | 9.49992e-08 | 454884 | 28.473 |
| Body mass index (BMI) | rs66511537 | 2 | 107493008 | T | C | 0.362 | -0.01 | 0.002 | 3.29997e-06 | 454884 | 21.606 |
| Body mass index (BMI) | rs66679256 | 4 | 18351898 | T | C | 0.446 | 0.015 | 0.002 | 1.9002e-14 | 454884 | 58.595 |
| Body mass index (BMI) | rs6669189 | 1 | 75011358 | T | C | 0.402 | 0.018 | 0.002 | 2.80027e-18 | 454884 | 76.035 |
| Body mass index (BMI) | rs6669341 | 1 | 47678458 | G | A | 0.583 | -0.017 | 0.002 | 9.20026e-18 | 454884 | 73.678 |
| Body mass index (BMI) | rs6682438 | 1 | 33784146 | C | T | 0.673 | 0.013 | 0.002 | 1.79999e-09 | 454884 | 36.146 |
| Body mass index (BMI) | rs6688826 | 1 | 80812329 | C | T | 0.298 | 0.014 | 0.002 | 7.70016e-11 | 454884 | 42.324 |
| Body mass index (BMI) | rs66978877 | 19 | 18455657 | C | T | 0.268 | -0.018 | 0.002 | 7.89951e-15 | 454884 | 60.352 |
| Body mass index (BMI) | rs6707827 | 2 | 100123030 | G | A | 0.704 | 0.012 | 0.002 | 1.7e-08 | 454884 | 31.756 |
| Body mass index (BMI) | rs6744646 | 2 | 628504 | G | A | 0.828 | 0.055 | 0.003 | 1.69981e-98 | 454884 | 443.706 |
| Body mass index (BMI) | rs6752979 | 2 | 81741750 | A | G | 0.317 | 0.012 | 0.002 | 1.09999e-08 | 454884 | 32.586 |
| Body mass index (BMI) | rs67609008 | 10 | 126640936 | C | T | 0.284 | 0.018 | 0.002 | 2.80027e-15 | 454884 | 62.389 |
| Body mass index (BMI) | rs6777784 | 3 | 62376645 | T | G | 0.617 | 0.012 | 0.002 | 1.29999e-09 | 454884 | 36.763 |
| Body mass index (BMI) | rs680071 | 11 | 103088414 | C | T | 0.881 | 0.017 | 0.003 | 7.29995e-08 | 454884 | 28.992 |
| Body mass index (BMI) | rs6805758 | 3 | 159645671 | C | A | 0.331 | 0.01 | 0.002 | 1e-06 | 454884 | 23.863 |
| Body mass index (BMI) | rs6843852 | 4 | 162132758 | T | C | 0.508 | 0.013 | 0.002 | 1.69981e-11 | 454884 | 45.312 |
| Body mass index (BMI) | rs6849333 | 4 | 16635746 | A | C | 0.488 | 0.011 | 0.002 | 7.00003e-08 | 454884 | 29.066 |
| Body mass index (BMI) | rs6909685 | 6 | 97753952 | T | C | 0.327 | -0.015 | 0.002 | 6.20012e-12 | 454884 | 47.275 |
| Body mass index (BMI) | rs6922607 | 6 | 142703483 | G | A | 0.19 | 0.014 | 0.003 | 1.5e-08 | 454884 | 32.071 |
| Body mass index (BMI) | rs6935708 | 6 | 29416652 | C | T | 0.012 | 0.046 | 0.009 | 3.40001e-07 | 454884 | 26.03 |
| Body mass index (BMI) | rs6938973 | 6 | 98421721 | C | T | 0.602 | 0.018 | 0.002 | 1.9002e-19 | 454884 | 81.383 |
| Body mass index (BMI) | rs6950388 | 7 | 1270699 | A | G | 0.795 | 0.016 | 0.002 | 9.20026e-11 | 454884 | 41.99 |
| Body mass index (BMI) | rs6973656 | 7 | 77422583 | G | A | 0.397 | 0.011 | 0.002 | 1.89998e-07 | 454884 | 27.112 |
| Body mass index (BMI) | rs698147 | 5 | 3513485 | G | A | 0.543 | -0.013 | 0.002 | 2.90001e-11 | 454884 | 44.225 |
| Body mass index (BMI) | rs6998660 | 8 | 143373972 | G | A | 0.453 | 0.012 | 0.002 | 2.5e-09 | 454884 | 35.506 |
| Body mass index (BMI) | rs7005244 | 8 | 27168961 | A | G | 0.557 | -0.01 | 0.002 | 9.29994e-07 | 454884 | 24.057 |
| Body mass index (BMI) | rs7024334 | 9 | 109072075 | G | T | 0.779 | -0.014 | 0.002 | 1.7e-08 | 454884 | 31.793 |
| Body mass index (BMI) | rs7027304 | 9 | 129408290 | T | C | 0.653 | 0.015 | 0.002 | 1.69981e-12 | 454884 | 49.753 |
| Body mass index (BMI) | rs7034554 | 9 | 37081301 | G | A | 0.374 | -0.014 | 0.002 | 4.70002e-11 | 454884 | 43.294 |
| Body mass index (BMI) | rs7038943 | 9 | 120377178 | C | T | 0.339 | -0.014 | 0.002 | 1.50003e-11 | 454884 | 45.587 |
| Body mass index (BMI) | rs705145 | 10 | 125226178 | A | C | 0.345 | 0.014 | 0.002 | 1.59993e-11 | 454884 | 45.398 |
| Body mass index (BMI) | rs7070670 | 10 | 61842645 | T | C | 0.328 | -0.013 | 0.002 | 2.69998e-09 | 454884 | 35.415 |
| Body mass index (BMI) | rs7109581 | 11 | 93180531 | G | T | 0.42 | 0.01 | 0.002 | 7.29995e-07 | 454884 | 24.532 |
| Body mass index (BMI) | rs7124681 | 11 | 47529947 | A | C | 0.408 | 0.026 | 0.002 | 1.39991e-37 | 454884 | 164.136 |
| Body mass index (BMI) | rs7132908 | 12 | 50263148 | A | G | 0.384 | 0.029 | 0.002 | 1.10002e-46 | 454884 | 205.856 |
| Body mass index (BMI) | rs7169847 | 15 | 95272920 | T | G | 0.636 | -0.014 | 0.002 | 1.99986e-11 | 454884 | 44.93 |
| Body mass index (BMI) | rs7201895 | 16 | 407723 | A | G | 0.354 | -0.015 | 0.002 | 8.00018e-13 | 454884 | 51.284 |
| Body mass index (BMI) | rs7218014 | 17 | 65832016 | C | T | 0.197 | 0.019 | 0.003 | 7.8001e-14 | 454884 | 55.849 |
| Body mass index (BMI) | rs7232171 | 18 | 31251221 | T | G | 0.583 | 0.013 | 0.002 | 1.2e-10 | 454884 | 41.464 |
| Body mass index (BMI) | rs7235469 | 18 | 69243117 | G | A | 0.86 | 0.015 | 0.003 | 7.49998e-08 | 454884 | 28.925 |
| Body mass index (BMI) | rs723672 | 12 | 2161561 | T | C | 0.432 | 0.011 | 0.002 | 4e-08 | 454884 | 30.172 |
| Body mass index (BMI) | rs7237312 | 18 | 22460541 | T | C | 0.211 | -0.011 | 0.002 | 2.90001e-06 | 454884 | 21.891 |
| Body mass index (BMI) | rs7259070 | 19 | 47562509 | C | T | 0.596 | 0.022 | 0.002 | 3.80014e-26 | 454884 | 111.891 |
| Body mass index (BMI) | rs72634826 | 1 | 1601052 | A | G | 0.26 | -0.021 | 0.002 | 2.09991e-19 | 454884 | 81.147 |
| Body mass index (BMI) | rs72744924 | 15 | 56876088 | G | A | 0.089 | 0.018 | 0.004 | 3.69999e-07 | 454884 | 25.83 |
| Body mass index (BMI) | rs72892910 | 6 | 50816887 | T | G | 0.172 | 0.039 | 0.003 | 6.59933e-49 | 454884 | 216.04 |
| Body mass index (BMI) | rs72910629 | 6 | 69761994 | G | A | 0.136 | 0.016 | 0.003 | 2.5e-08 | 454884 | 31.026 |
| Body mass index (BMI) | rs72915955 | 11 | 56909311 | A | G | 0.161 | -0.014 | 0.003 | 1.2e-07 | 454884 | 28.093 |
| Body mass index (BMI) | rs72948836 | 6 | 73910108 | G | A | 0.059 | -0.024 | 0.004 | 7.00003e-09 | 454884 | 33.549 |
| Body mass index (BMI) | rs73052033 | 3 | 185828465 | C | T | 0.185 | -0.03 | 0.003 | 1e-31 | 454884 | 137.277 |
| Body mass index (BMI) | rs7306534 | 12 | 68107914 | A | G | 0.622 | -0.011 | 0.002 | 5.60003e-08 | 454884 | 29.483 |
| Body mass index (BMI) | rs7306544 | 12 | 132439381 | C | T | 0.105 | 0.016 | 0.003 | 6.69993e-07 | 454884 | 24.713 |
| Body mass index (BMI) | rs73142879 | 20 | 51195932 | T | C | 0.192 | -0.027 | 0.003 | 9.30037e-27 | 454884 | 114.673 |
| Body mass index (BMI) | rs73193736 | 12 | 108294381 | G | A | 0.244 | -0.018 | 0.002 | 3.59998e-15 | 454884 | 61.916 |
| Body mass index (BMI) | rs7331420 | 13 | 99236471 | A | G | 0.285 | -0.014 | 0.002 | 1.6e-10 | 454884 | 40.854 |
| Body mass index (BMI) | rs73529119 | 8 | 15126775 | T | C | 0.114 | 0.016 | 0.003 | 6.19998e-07 | 454884 | 24.854 |
| Body mass index (BMI) | rs7357754 | 9 | 92207308 | G | A | 0.5 | 0.014 | 0.002 | 5.79963e-13 | 454884 | 51.912 |
| Body mass index (BMI) | rs73601548 | 10 | 18549889 | T | C | 0.115 | 0.017 | 0.003 | 4.90004e-08 | 454884 | 29.763 |
| Body mass index (BMI) | rs74252325 | 17 | 2546340 | T | C | 0.212 | 0.011 | 0.002 | 3.09999e-06 | 454884 | 21.733 |
| Body mass index (BMI) | rs7442137 | 4 | 80718632 | T | C | 0.634 | -0.012 | 0.002 | 1.6e-09 | 454884 | 36.442 |
| Body mass index (BMI) | rs745249 | 2 | 105460333 | T | C | 0.282 | 0.018 | 0.002 | 3.29989e-16 | 454884 | 66.597 |
| Body mass index (BMI) | rs7498044 | 15 | 92573639 | A | G | 0.217 | -0.017 | 0.002 | 4.70002e-12 | 454884 | 47.807 |
| Body mass index (BMI) | rs7498665 | 16 | 28883241 | G | A | 0.4 | 0.027 | 0.002 | 9.30037e-39 | 454884 | 169.535 |
| Body mass index (BMI) | rs75035127 | 1 | 174973726 | G | A | 0.03 | -0.041 | 0.006 | 1.20005e-12 | 454884 | 50.434 |
| Body mass index (BMI) | rs7516554 | 1 | 210301331 | T | C | 0.4 | 0.012 | 0.002 | 1.5e-09 | 454884 | 36.499 |
| Body mass index (BMI) | rs7519259 | 1 | 66434743 | A | G | 0.528 | 0.014 | 0.002 | 5.79963e-12 | 454884 | 47.394 |
| Body mass index (BMI) | rs75499503 | 6 | 26145217 | T | C | 0.22 | -0.018 | 0.002 | 3.10027e-13 | 454884 | 53.16 |
| Body mass index (BMI) | rs75557510 | 3 | 84321469 | G | A | 0.061 | -0.031 | 0.004 | 1.59993e-13 | 454884 | 54.398 |
| Body mass index (BMI) | rs7571496 | 2 | 6169351 | G | A | 0.261 | -0.015 | 0.002 | 1.10002e-11 | 454884 | 46.223 |
| Body mass index (BMI) | rs760644 | 1 | 171509327 | A | G | 0.82 | -0.014 | 0.003 | 1.7e-07 | 454884 | 27.381 |
| Body mass index (BMI) | rs76183894 | 3 | 114371939 | C | T | 0.081 | -0.022 | 0.004 | 2.69998e-09 | 454884 | 35.397 |
| Body mass index (BMI) | rs76387394 | 8 | 85685773 | G | A | 0.053 | 0.027 | 0.004 | 1e-09 | 454884 | 37.284 |
| Body mass index (BMI) | rs76560824 | 11 | 370252 | C | T | 0.102 | -0.018 | 0.003 | 1.09999e-07 | 454884 | 28.274 |
| Body mass index (BMI) | rs76638898 | 10 | 21099584 | A | G | 0.024 | -0.032 | 0.007 | 2.30001e-06 | 454884 | 22.323 |
| Body mass index (BMI) | rs7693817 | 4 | 144051525 | C | T | 0.6 | -0.011 | 0.002 | 2e-07 | 454884 | 27.067 |
| Body mass index (BMI) | rs77188232 | 19 | 30523391 | A | G | 0.082 | 0.019 | 0.004 | 5.69994e-07 | 454884 | 25.023 |
| Body mass index (BMI) | rs7721385 | 5 | 176162581 | T | C | 0.458 | 0.011 | 0.002 | 6.59994e-08 | 454884 | 29.17 |
| Body mass index (BMI) | rs7742698 | 6 | 147354315 | G | T | 0.367 | 0.011 | 0.002 | 2.19999e-07 | 454884 | 26.841 |
| Body mass index (BMI) | rs7762794 | 6 | 153380228 | G | A | 0.286 | 0.015 | 0.002 | 1.99986e-11 | 454884 | 44.946 |
| Body mass index (BMI) | rs7768724 | 6 | 80279141 | T | G | 0.132 | 0.015 | 0.003 | 5.49997e-07 | 454884 | 25.063 |
| Body mass index (BMI) | rs7774 | 17 | 4801163 | A | C | 0.31 | 0.015 | 0.002 | 1.50003e-12 | 454884 | 50.068 |
| Body mass index (BMI) | rs7805441 | 7 | 78121458 | T | C | 0.502 | 0.013 | 0.002 | 2.60016e-11 | 454884 | 44.494 |
| Body mass index (BMI) | rs7828631 | 8 | 132875320 | T | C | 0.11 | 0.018 | 0.003 | 1.2e-08 | 454884 | 32.466 |
| Body mass index (BMI) | rs784257 | 18 | 53397199 | C | T | 0.813 | 0.018 | 0.003 | 2.39994e-12 | 454884 | 49.105 |
| Body mass index (BMI) | rs7852189 | 9 | 103121547 | G | A | 0.316 | 0.017 | 0.002 | 1.10002e-15 | 454884 | 64.252 |
| Body mass index (BMI) | rs78643454 | 12 | 47864423 | A | G | 0.08 | 0.018 | 0.004 | 2.1e-06 | 454884 | 22.517 |
| Body mass index (BMI) | rs7893571 | 10 | 16750129 | T | G | 0.666 | 0.014 | 0.002 | 9.3994e-12 | 454884 | 46.458 |
| Body mass index (BMI) | rs79027764 | 6 | 160891231 | C | T | 0.021 | -0.034 | 0.007 | 8e-07 | 454884 | 24.35 |
| Body mass index (BMI) | rs7916385 | 10 | 102470432 | T | C | 0.15 | -0.024 | 0.003 | 1.80011e-15 | 454884 | 63.245 |
| Body mass index (BMI) | rs79212998 | 15 | 53402758 | G | T | 0.069 | -0.023 | 0.004 | 2.80001e-09 | 454884 | 35.286 |
| Body mass index (BMI) | rs7924036 | 10 | 65191645 | T | G | 0.503 | -0.014 | 0.002 | 3.50026e-13 | 454884 | 52.889 |
| Body mass index (BMI) | rs7925100 | 11 | 118941596 | A | G | 0.396 | 0.014 | 0.002 | 1.10002e-12 | 454884 | 50.578 |
| Body mass index (BMI) | rs7925322 | 11 | 16406762 | G | A | 0.283 | 0.011 | 0.002 | 1.09999e-06 | 454884 | 23.715 |
| Body mass index (BMI) | rs7944782 | 11 | 130795698 | G | T | 0.51 | 0.016 | 0.002 | 1.39991e-15 | 454884 | 63.731 |
| Body mass index (BMI) | rs7947143 | 11 | 64090422 | A | G | 0.163 | -0.018 | 0.003 | 3.59998e-11 | 454884 | 43.794 |
| Body mass index (BMI) | rs7952102 | 11 | 892089 | C | T | 0.388 | -0.014 | 0.002 | 2.80027e-12 | 454884 | 48.813 |
| Body mass index (BMI) | rs7975187 | 12 | 60964108 | G | A | 0.214 | 0.015 | 0.002 | 1.79999e-10 | 454884 | 40.643 |
| Body mass index (BMI) | rs79780963 | 10 | 104952499 | T | C | 0.077 | 0.024 | 0.004 | 2.5e-10 | 454884 | 40.017 |
| Body mass index (BMI) | rs79906980 | 1 | 57887985 | T | C | 0.16 | 0.014 | 0.003 | 2.30001e-07 | 454884 | 26.783 |
| Body mass index (BMI) | rs79966207 | 22 | 50722408 | C | T | 0.176 | 0.014 | 0.003 | 2.39999e-07 | 454884 | 26.713 |
| Body mass index (BMI) | rs7996639 | 13 | 97019090 | A | G | 0.449 | 0.014 | 0.002 | 1.20005e-12 | 454884 | 50.536 |
| Body mass index (BMI) | rs8007832 | 14 | 56035579 | C | T | 0.364 | -0.011 | 0.002 | 3.2e-07 | 454884 | 26.103 |
| Body mass index (BMI) | rs8015400 | 14 | 25930988 | A | C | 0.677 | 0.021 | 0.002 | 9.60064e-24 | 454884 | 100.913 |
| Body mass index (BMI) | rs8076669 | 17 | 15888448 | C | T | 0.562 | 0.014 | 0.002 | 5.90065e-12 | 454884 | 47.368 |
| Body mass index (BMI) | rs8126575 | 21 | 46435610 | G | T | 0.139 | -0.016 | 0.003 | 1.2e-07 | 454884 | 27.959 |
| Body mass index (BMI) | rs8132491 | 21 | 40288577 | A | G | 0.313 | -0.016 | 0.002 | 2.09991e-12 | 454884 | 49.401 |
| Body mass index (BMI) | rs815163 | 1 | 190294726 | C | T | 0.563 | -0.017 | 0.002 | 4.60045e-17 | 454884 | 70.494 |
| Body mass index (BMI) | rs8176166 | 17 | 41240277 | C | T | 0.149 | -0.014 | 0.003 | 9.20005e-07 | 454884 | 24.094 |
| Body mass index (BMI) | rs862320 | 16 | 69651866 | T | C | 0.41 | -0.023 | 0.002 | 2.19989e-30 | 454884 | 131.226 |
| Body mass index (BMI) | rs876605 | 5 | 77801359 | G | A | 0.74 | -0.011 | 0.002 | 2e-06 | 454884 | 22.552 |
| Body mass index (BMI) | rs878627 | 18 | 76456503 | T | C | 0.379 | -0.01 | 0.002 | 2.30001e-06 | 454884 | 22.337 |
| Body mass index (BMI) | rs879620 | 16 | 4015729 | T | C | 0.613 | 0.024 | 0.002 | 5.70033e-32 | 454884 | 138.483 |
| Body mass index (BMI) | rs909892 | 20 | 41982698 | A | G | 0.135 | -0.018 | 0.003 | 8.60003e-10 | 454884 | 37.611 |
| Body mass index (BMI) | rs9290411 | 3 | 171108171 | G | T | 0.507 | -0.01 | 0.002 | 2.99999e-07 | 454884 | 26.219 |
| Body mass index (BMI) | rs9294260 | 6 | 83433228 | A | G | 0.477 | 0.015 | 0.002 | 1.99986e-13 | 454884 | 53.96 |
| Body mass index (BMI) | rs9309184 | 2 | 50891801 | G | A | 0.457 | -0.01 | 0.002 | 1.29999e-06 | 454884 | 23.373 |
| Body mass index (BMI) | rs935166 | 2 | 26949366 | A | G | 0.507 | -0.016 | 0.002 | 5.90065e-16 | 454884 | 65.475 |
| Body mass index (BMI) | rs9359658 | 6 | 86305769 | T | C | 0.594 | -0.009 | 0.002 | 2.90001e-06 | 454884 | 21.895 |
| Body mass index (BMI) | rs9366863 | 6 | 34688946 | C | T | 0.672 | -0.028 | 0.002 | 2.60016e-40 | 454884 | 176.684 |
| Body mass index (BMI) | rs9369370 | 6 | 42565741 | G | A | 0.414 | 0.011 | 0.002 | 1.7e-08 | 454884 | 31.845 |
| Body mass index (BMI) | rs9395885 | 6 | 53745477 | T | C | 0.084 | 0.017 | 0.004 | 2.19999e-06 | 454884 | 22.413 |
| Body mass index (BMI) | rs9400481 | 6 | 111850742 | T | C | 0.174 | 0.013 | 0.003 | 6.49995e-07 | 454884 | 24.754 |
| Body mass index (BMI) | rs9408878 | 9 | 118637741 | A | G | 0.497 | -0.01 | 0.002 | 8.30004e-07 | 454884 | 24.296 |
| Body mass index (BMI) | rs9463175 | 6 | 9510030 | T | C | 0.339 | -0.01 | 0.002 | 8.40001e-07 | 454884 | 24.256 |
| Body mass index (BMI) | rs947088 | 20 | 17171373 | T | G | 0.718 | 0.013 | 0.002 | 9.80009e-09 | 454884 | 32.882 |
| Body mass index (BMI) | rs9478496 | 6 | 154333183 | C | T | 0.164 | 0.018 | 0.003 | 6.59933e-11 | 454884 | 42.63 |
| Body mass index (BMI) | rs9514131 | 13 | 104090848 | T | G | 0.122 | -0.015 | 0.003 | 4.90004e-07 | 454884 | 25.307 |
| Body mass index (BMI) | rs9521287 | 13 | 89212569 | C | T | 0.886 | -0.014 | 0.003 | 4.39997e-06 | 454884 | 21.099 |
| Body mass index (BMI) | rs9571687 | 13 | 67472713 | A | C | 0.329 | -0.013 | 0.002 | 4.60002e-10 | 454884 | 38.857 |
| Body mass index (BMI) | rs958261 | 1 | 80014353 | T | C | 0.618 | -0.009 | 0.002 | 3.89996e-06 | 454884 | 21.326 |
| Body mass index (BMI) | rs9594704 | 13 | 42780398 | C | T | 0.28 | -0.01 | 0.002 | 4.49997e-06 | 454884 | 21.021 |
| Body mass index (BMI) | rs9638713 | 7 | 14645949 | G | A | 0.975 | -0.036 | 0.006 | 2.39999e-08 | 454884 | 31.151 |
| Body mass index (BMI) | rs9652638 | 16 | 6498714 | T | C | 0.504 | -0.009 | 0.002 | 2.90001e-06 | 454884 | 21.861 |
| Body mass index (BMI) | rs9673839 | 16 | 76895693 | G | A | 0.491 | 0.013 | 0.002 | 8.80035e-11 | 454884 | 42.068 |
| Body mass index (BMI) | rs973741 | 3 | 100953551 | A | G | 0.103 | -0.015 | 0.003 | 4.30002e-06 | 454884 | 21.114 |
| Body mass index (BMI) | rs9839081 | 3 | 123051230 | A | G | 0.325 | -0.012 | 0.002 | 7.39997e-08 | 454884 | 28.946 |
| Body mass index (BMI) | rs9843653 | 3 | 49920571 | C | T | 0.512 | 0.029 | 0.002 | 7.70016e-49 | 454884 | 215.732 |
| Body mass index (BMI) | rs9888533 | 13 | 107854612 | T | C | 0.538 | 0.012 | 0.002 | 2.19999e-09 | 454884 | 35.768 |
| Body mass index (BMI) | rs9951619 | 18 | 56882326 | G | T | 0.767 | 0.015 | 0.002 | 2.90001e-10 | 454884 | 39.746 |
| Body mass index (BMI) | rs9962691 | 18 | 38149766 | A | G | 0.152 | 0.013 | 0.003 | 2.30001e-06 | 454884 | 22.303 |
| Body mass index (BMI) | rs9991259 | 4 | 112686354 | A | G | 0.633 | 0.011 | 0.002 | 4.39997e-08 | 454884 | 29.974 |
| Obesity | rs10913469 | 1 | 177913519 | C | T | NA | 0.177 | 0.033 | 7.98693e-08 | 13848 | NA |
| Obesity | rs13130484 | 4 | 45175691 | T | C | NA | 0.143 | 0.027 | 1.30299e-07 | 13848 | NA |
| Obesity | rs17697518 | 18 | 38765659 | T | C | NA | 0.186 | 0.039 | 1.85302e-06 | 13848 | NA |
| Obesity | rs256335 | 19 | 34315896 | T | C | NA | 0.121 | 0.026 | 3.721e-06 | 13848 | NA |
| Obesity | rs28636 | 5 | 66149113 | T | C | NA | -0.147 | 0.032 | 3.07496e-06 | 13848 | NA |
| Obesity | rs4833407 | 4 | 113311790 | A | C | NA | 0.123 | 0.026 | 3.879e-06 | 13848 | NA |
| Obesity | rs4854344 | 2 | 638144 | T | G | NA | 0.244 | 0.035 | 3.22329e-12 | 13848 | NA |
| Obesity | rs4864201 | 4 | 130731284 | C | T | NA | -0.136 | 0.028 | 1.41299e-06 | 13848 | NA |
| Obesity | rs571312 | 18 | 57839769 | A | C | NA | 0.199 | 0.031 | 1.25e-10 | 13848 | NA |
| Obesity | rs6752378 | 2 | 25150116 | A | C | NA | 0.17 | 0.026 | 1.05099e-10 | 13848 | NA |
| Obesity | rs7138803 | 12 | 50247468 | A | G | NA | 0.167 | 0.027 | 6.49501e-10 | 13848 | NA |
| Obesity | rs9299 | 17 | 46669430 | T | C | NA | 0.134 | 0.028 | 1.907e-06 | 13848 | NA |
| Obesity | rs9568856 | 13 | 54064981 | A | G | NA | 0.191 | 0.04 | 1.359e-06 | 13848 | NA |
| Obesity | rs9941349 | 16 | 53825488 | T | C | NA | 0.198 | 0.027 | 1.15904e-13 | 13848 | NA |
| Obesity class 1 | rs10182181 | 2 | 25150296 | G | A | 0.5 | 0.073 | 0.012 | 3.29997e-09 | 98635 | 37.006 |
| Obesity class 1 | rs10733682 | 9 | 129460914 | G | A | 0.568 | -0.059 | 0.013 | 3.59998e-06 | 98658 | 20.597 |
| Obesity class 1 | rs10968576 | 9 | 28414339 | G | A | 0.292 | 0.068 | 0.013 | 3.89996e-07 | 95893 | 27.36 |
| Obesity class 1 | rs11073381 | 15 | 95265012 | G | T | 0.492 | -0.058 | 0.012 | 2.90001e-06 | 98667 | 23.361 |
| Obesity class 1 | rs11075989 | 16 | 53819877 | T | C | 0.407 | 0.21 | 0.012 | 5.00035e-67 | 98684 | 306.244 |
| Obesity class 1 | rs11672660 | 19 | 46180184 | T | C | 0.149 | -0.084 | 0.017 | 1e-06 | 85585 | 24.415 |
| Obesity class 1 | rs11847697 | 14 | 30515112 | T | C | 0.042 | 0.2 | 0.039 | 4.49997e-07 | 59572 | 26.298 |
| Obesity class 1 | rs12141962 | 1 | 49484873 | T | G | 0.237 | 0.07 | 0.015 | 3.59998e-06 | 96473 | 21.777 |
| Obesity class 1 | rs13130484 | 4 | 45175691 | T | C | 0.424 | 0.11 | 0.013 | 3.40017e-16 | 98425 | 71.596 |
| Obesity class 1 | rs13393304 | 2 | 637830 | G | A | 0.89 | 0.18 | 0.017 | 9.49948e-27 | 93304 | 112.108 |
| Obesity class 1 | rs17024258 | 1 | 110147321 | T | C | 0.042 | 0.21 | 0.044 | 1.40001e-06 | 57088 | 22.778 |
| Obesity class 1 | rs17318744 | 1 | 210244470 | G | A | 0.25 | -0.066 | 0.014 | 2.59998e-06 | 96815 | 22.224 |
| Obesity class 1 | rs180829 | 18 | 40063124 | A | C | 0.725 | -0.065 | 0.013 | 6.80002e-07 | 97269 | 24.999 |
| Obesity class 1 | rs2030323 | 11 | 27728539 | C | A | 0.783 | 0.11 | 0.015 | 2.49977e-12 | 96889 | 53.777 |
| Obesity class 1 | rs2196618 | 8 | 85089437 | G | A | 0.767 | 0.079 | 0.015 | 9.59997e-08 | 96941 | 27.737 |
| Obesity class 1 | rs2307111 | 5 | 75003678 | C | T | 0.425 | -0.069 | 0.013 | 3.29997e-08 | 98488 | 28.171 |
| Obesity class 1 | rs2531995 | 16 | 4013467 | T | C | 0.592 | 0.063 | 0.013 | 3.2e-06 | 97975 | 23.485 |
| Obesity class 1 | rs2881156 | 5 | 135148662 | G | A | 0.396 | 0.073 | 0.015 | 8.09991e-07 | 97122 | 23.684 |
| Obesity class 1 | rs2984433 | 6 | 39973445 | G | T | 0.642 | 0.062 | 0.013 | 4.39997e-06 | 97891 | 22.745 |
| Obesity class 1 | rs29939 | 19 | 34310800 | G | A | 0.667 | 0.072 | 0.013 | 4.20001e-08 | 96557 | 30.674 |
| Obesity class 1 | rs4498364 | 6 | 97613773 | T | C | 0.7 | -0.065 | 0.013 | 1e-06 | 97799 | 24.999 |
| Obesity class 1 | rs4735692 | 8 | 76615663 | G | A | 0.442 | -0.068 | 0.013 | 5e-08 | 98372 | 27.36 |
| Obesity class 1 | rs4788099 | 16 | 28855727 | G | A | 0.362 | 0.068 | 0.013 | 5.99998e-08 | 98695 | 27.36 |
| Obesity class 1 | rs4929923 | 11 | 8639200 | C | T | 0.729 | 0.075 | 0.013 | 8e-09 | 98328 | 33.283 |
| Obesity class 1 | rs527248 | 1 | 177875514 | G | A | 0.263 | 0.11 | 0.016 | 6.29941e-13 | 96419 | 47.265 |
| Obesity class 1 | rs7023913 | 9 | 131017962 | G | A | 0.271 | 0.075 | 0.015 | 7.29995e-07 | 97010 | 24.999 |
| Obesity class 1 | rs7138803 | 12 | 50247468 | A | G | 0.442 | 0.084 | 0.013 | 2.60016e-11 | 98653 | 41.751 |
| Obesity class 1 | rs7141420 | 14 | 79899454 | T | C | 0.621 | 0.079 | 0.012 | 2.30001e-10 | 98622 | 43.339 |
| Obesity class 1 | rs7164558 | 15 | 78146382 | G | A | 0.604 | -0.064 | 0.013 | 5.60003e-07 | 97962 | 24.236 |
| Obesity class 1 | rs7531118 | 1 | 72837239 | C | T | 0.608 | 0.08 | 0.013 | 4.60002e-10 | 98523 | 37.869 |
| Obesity class 1 | rs7604277 | 2 | 58823770 | C | T | 0.325 | -0.06 | 0.013 | 2.90001e-06 | 98650 | 21.301 |
| Obesity class 1 | rs7607584 | 2 | 142999043 | C | T | 0.092 | 0.091 | 0.02 | 3.89996e-06 | 94179 | 20.702 |
| Obesity class 1 | rs8097783 | 18 | 58051294 | A | G | 0.117 | -0.12 | 0.026 | 4.60002e-06 | 78610 | 21.301 |
| Obesity class 1 | rs815611 | 5 | 153518766 | A | G | 0.533 | -0.058 | 0.012 | 3.40001e-06 | 98678 | 23.361 |
| Obesity class 1 | rs887912 | 2 | 59302877 | C | T | 0.681 | -0.082 | 0.014 | 2.30001e-09 | 97424 | 34.305 |
| Obesity class 1 | rs987237 | 6 | 50803050 | G | A | 0.098 | 0.13 | 0.016 | 1e-16 | 95109 | 66.014 |
| Obesity class 2 | rs1000232 | 17 | 45356561 | C | T | 0.337 | 0.1 | 0.02 | 6.90001e-07 | 71289 | 24.999 |
| Obesity class 2 | rs1016287 | 2 | 59305625 | C | T | 0.689 | -0.1 | 0.021 | 5.80003e-07 | 70866 | 22.675 |
| Obesity class 2 | rs10840063 | 11 | 8522182 | C | T | 0.424 | -0.091 | 0.019 | 1.79999e-06 | 72289 | 22.938 |
| Obesity class 2 | rs10847689 | 12 | 122613000 | C | T | 0.225 | -0.12 | 0.025 | 1.09999e-06 | 66891 | 23.039 |
| Obesity class 2 | rs10871777 | 18 | 57851763 | G | A | 0.283 | 0.17 | 0.023 | 5.30029e-14 | 68784 | 54.63 |
| Obesity class 2 | rs11900505 | 2 | 25131986 | C | A | 0.44 | 0.093 | 0.019 | 1.09999e-06 | 71902 | 23.958 |
| Obesity class 2 | rs12444979 | 16 | 19933600 | T | C | 0.06 | -0.17 | 0.03 | 6.80002e-09 | 62287 | 32.11 |
| Obesity class 2 | rs12914773 | 15 | 68019958 | A | G | 0.562 | 0.099 | 0.019 | 2.39999e-07 | 71919 | 27.149 |
| Obesity class 2 | rs13130484 | 4 | 45175691 | T | C | 0.424 | 0.12 | 0.02 | 1.6e-09 | 71761 | 35.999 |
| Obesity class 2 | rs13401686 | 2 | 650519 | G | A | 0.125 | -0.24 | 0.027 | 1.29987e-19 | 65303 | 79.01 |
| Obesity class 2 | rs1415991 | 1 | 219798632 | G | A | 0.217 | 0.11 | 0.023 | 2.39999e-06 | 70145 | 22.873 |
| Obesity class 2 | rs1516725 | 3 | 185824004 | C | T | 0.908 | 0.19 | 0.03 | 1.2e-10 | 61014 | 40.11 |
| Obesity class 2 | rs2030323 | 11 | 27728539 | C | A | 0.783 | 0.13 | 0.024 | 5.19996e-08 | 66556 | 29.339 |
| Obesity class 2 | rs2112347 | 5 | 75015242 | G | T | 0.381 | -0.1 | 0.02 | 3.69999e-07 | 71951 | 24.999 |
| Obesity class 2 | rs2207139 | 6 | 50845490 | G | A | 0.102 | 0.17 | 0.025 | 8.19974e-12 | 62999 | 46.239 |
| Obesity class 2 | rs2815752 | 1 | 72812440 | A | G | 0.65 | 0.11 | 0.02 | 4.79999e-09 | 71717 | 30.249 |
| Obesity class 2 | rs29938 | 19 | 34311481 | C | T | 0.653 | 0.096 | 0.02 | 2.1e-06 | 71108 | 23.039 |
| Obesity class 2 | rs3732138 | 2 | 58685318 | A | C | 0.15 | 0.16 | 0.031 | 1.79999e-07 | 59288 | 26.638 |
| Obesity class 2 | rs620985 | 9 | 79318921 | G | A | 0.627 | 0.089 | 0.019 | 3.59998e-06 | 71978 | 21.941 |
| Obesity class 2 | rs633715 | 1 | 177852580 | C | T | 0.267 | 0.16 | 0.024 | 2.60016e-11 | 65464 | 44.443 |
| Obesity class 2 | rs7138803 | 12 | 50247468 | A | G | 0.442 | 0.13 | 0.019 | 1.99986e-11 | 71541 | 46.813 |
| Obesity class 2 | rs7176675 | 15 | 95257956 | C | T | 0.583 | -0.092 | 0.019 | 2e-06 | 71943 | 23.445 |
| Obesity class 2 | rs7184597 | 16 | 28921809 | C | T | 0.729 | -0.11 | 0.022 | 5.1e-07 | 70459 | 24.999 |
| Obesity class 2 | rs7527364 | 1 | 49566557 | G | A | 0.658 | -0.1 | 0.02 | 4e-07 | 71251 | 24.999 |
| Obesity class 2 | rs7989336 | 13 | 97017548 | A | G | 0.425 | 0.11 | 0.019 | 5.89997e-09 | 71938 | 33.517 |
| Obesity class 2 | rs8051591 | 16 | 53816752 | G | A | 0.448 | 0.29 | 0.019 | 3.80014e-51 | 71976 | 232.958 |
| Obesity class 2 | rs9512893 | 13 | 28416179 | A | G | 0.025 | 0.21 | 0.046 | 2.99999e-06 | 47813 | 20.84 |
| Obesity class 2 | rs9829032 | 3 | 85683600 | G | A | 0.432 | 0.091 | 0.02 | 3.79997e-06 | 71072 | 20.702 |
| Obesity class 3 | rs13104545 | 4 | 45184907 | A | G | 0.194 | 0.29 | 0.055 | 1.2e-07 | 35767 | 27.8 |
| Obesity class 3 | rs1317005 | 2 | 656312 | A | C | 0.278 | 0.2 | 0.04 | 1.09999e-06 | 40632 | 24.999 |
| Obesity class 3 | rs1421085 | 16 | 53800954 | C | T | 0.448 | 0.37 | 0.035 | 3.90032e-26 | 47527 | 111.75 |
| Obesity class 3 | rs2206277 | 6 | 50798526 | T | C | 0.096 | 0.27 | 0.052 | 2e-07 | 31686 | 26.958 |
| Obesity class 3 | rs2904669 | 8 | 17053322 | A | G | 0.492 | 0.16 | 0.035 | 3.59998e-06 | 46657 | 20.897 |
| Obesity class 3 | rs4074720 | 10 | 114748497 | T | C | 0.417 | -0.16 | 0.034 | 1.2e-06 | 48876 | 22.144 |
| Obesity class 3 | rs633715 | 1 | 177852580 | C | T | 0.267 | 0.22 | 0.047 | 2e-06 | 33148 | 21.909 |
| Obesity class 3 | rs6703138 | 1 | 202847719 | G | A | 0.225 | 0.23 | 0.047 | 7.29995e-07 | 32467 | 23.946 |
| Obesity class 3 | rs782236 | 1 | 72934826 | G | A | 0.317 | -0.17 | 0.036 | 2.30001e-06 | 45377 | 22.298 |
| Obesity class 3 | rs7973894 | 12 | 50233256 | A | G | 0.492 | 0.19 | 0.034 | 2.30001e-08 | 48807 | 31.227 |
| smoking initiation | rs10001365 | 4 | 147797214 | A | G | 0.405 | -0.025 | 0.004 | 6.65e-12 | 632802 | 47.1 |
| smoking initiation | rs1004787 | 2 | 45159091 | A | G | 0.581 | 0.03 | 0.004 | 5.27e-17 | 632802 | 70.2 |
| smoking initiation | rs10062607 | 5 | 79290634 | A | C | 0.615 | 0.018 | 0.004 | 1.61e-06 | 632802 | 23 |
| smoking initiation | rs10119117 | 9 | 29740028 | T | C | 0.51 | 0.017 | 0.004 | 1.72e-06 | 632802 | 22.9 |
| smoking initiation | rs10130576 | 14 | 28291917 | A | G | 0.389 | 0.019 | 0.004 | 3.16e-07 | 632802 | 26.2 |
| smoking initiation | rs10179773 | 2 | 49849268 | A | G | 0.217 | -0.019 | 0.004 | 3.89e-06 | 632802 | 21.3 |
| smoking initiation | rs10212466 | 3 | 43994607 | T | G | 0.398 | -0.017 | 0.004 | 2.29e-06 | 632802 | 22.3 |
| smoking initiation | rs10233018 | 7 | 117523709 | G | A | 0.503 | 0.027 | 0.004 | 2.75e-14 | 632802 | 57.9 |
| smoking initiation | rs10260968 | 7 | 1889773 | A | G | 0.597 | -0.02 | 0.004 | 1.75e-08 | 632802 | 31.7 |
| smoking initiation | rs10279261 | 7 | 133589846 | A | G | 0.619 | -0.021 | 0.004 | 5e-09 | 632802 | 34.2 |
| smoking initiation | rs10446671 | 4 | 35483932 | T | C | 0.482 | 0.017 | 0.004 | 3.28e-06 | 632802 | 21.6 |
| smoking initiation | rs10498846 | 6 | 67405337 | T | C | 0.473 | 0.021 | 0.004 | 6.62e-09 | 632802 | 33.6 |
| smoking initiation | rs10507911 | 13 | 81189301 | T | G | 0.229 | -0.021 | 0.004 | 5.53e-07 | 632802 | 25.1 |
| smoking initiation | rs1050847 | 16 | 87443734 | T | C | 0.505 | -0.022 | 0.004 | 1.67e-09 | 632802 | 36.3 |
| smoking initiation | rs10698713 | 6 | 158882320 | A | G | 0.054 | -0.043 | 0.008 | 8.29e-08 | 632802 | 28.7 |
| smoking initiation | rs10819050 | 9 | 128237178 | T | G | 0.423 | 0.02 | 0.004 | 1.64e-07 | 632802 | 27.4 |
| smoking initiation | rs10873871 | 1 | 76689019 | G | A | 0.191 | 0.021 | 0.004 | 1.9e-06 | 632802 | 22.7 |
| smoking initiation | rs10905461 | 10 | 8803551 | C | T | 0.718 | -0.024 | 0.004 | 7.35e-09 | 632802 | 33.4 |
| smoking initiation | rs10934272 | 3 | 114151511 | T | C | 0.736 | 0.019 | 0.004 | 3.78e-06 | 632802 | 21.4 |
| smoking initiation | rs10953957 | 7 | 121954709 | A | G | 0.392 | 0.018 | 0.004 | 4.72e-07 | 632802 | 25.4 |
| smoking initiation | rs10968592 | 9 | 28429226 | C | T | 0.077 | 0.031 | 0.007 | 3.34e-06 | 632802 | 21.6 |
| smoking initiation | rs10997380 | 10 | 68542366 | T | C | 0.798 | -0.02 | 0.004 | 4.59e-06 | 632802 | 21 |
| smoking initiation | rs11057005 | 12 | 16748721 | G | A | 0.43 | -0.021 | 0.004 | 4.85e-09 | 632802 | 34.2 |
| smoking initiation | rs11076967 | 16 | 5834006 | T | G | 0.289 | 0.021 | 0.004 | 7.34e-07 | 632802 | 24.5 |
| smoking initiation | rs11078713 | 17 | 7795972 | G | A | 0.454 | -0.02 | 0.004 | 2.23e-08 | 632802 | 31.3 |
| smoking initiation | rs11256434 | 10 | 10044905 | C | T | 0.403 | -0.017 | 0.004 | 1.85e-06 | 632802 | 22.7 |
| smoking initiation | rs1126757 | 19 | 55879872 | T | C | 0.469 | 0.019 | 0.004 | 1.53e-07 | 632802 | 27.6 |
| smoking initiation | rs113230003 | 19 | 18460956 | A | G | 0.249 | -0.021 | 0.004 | 5.19e-07 | 632802 | 25.2 |
| smoking initiation | rs113746525 | 2 | 60977295 | T | G | 0.026 | -0.057 | 0.011 | 5.65e-07 | 632802 | 25 |
| smoking initiation | rs1154693 | 3 | 117804154 | G | A | 0.856 | 0.033 | 0.005 | 3.12e-11 | 632802 | 44.1 |
| smoking initiation | rs11610032 | 12 | 108397264 | C | T | 0.205 | -0.022 | 0.004 | 3.08e-07 | 632802 | 26.2 |
| smoking initiation | rs11611651 | 12 | 133380790 | A | G | 0.078 | 0.034 | 0.006 | 8.02e-08 | 632802 | 28.8 |
| smoking initiation | rs116531886 | 1 | 108452826 | G | A | 0.053 | -0.036 | 0.008 | 3.47e-06 | 632802 | 21.5 |
| smoking initiation | rs11658881 | 17 | 2072949 | G | A | 0.418 | 0.02 | 0.004 | 2.43e-08 | 632802 | 31.1 |
| smoking initiation | rs11692232 | 2 | 228464165 | A | C | 0.099 | 0.026 | 0.006 | 4.38e-06 | 632802 | 21.1 |
| smoking initiation | rs11692435 | 2 | 98275354 | A | G | 0.086 | 0.03 | 0.006 | 8.71e-07 | 632802 | 24.2 |
| smoking initiation | rs11712680 | 3 | 75009019 | C | A | 0.174 | -0.027 | 0.005 | 3.51e-09 | 632802 | 34.9 |
| smoking initiation | rs117143374 | 21 | 40555561 | C | T | 0.12 | 0.029 | 0.005 | 2.76e-08 | 632802 | 30.9 |
| smoking initiation | rs11716705 | 3 | 34728753 | G | A | 0.253 | 0.02 | 0.004 | 1.45e-06 | 632802 | 23.2 |
| smoking initiation | rs11872397 | 18 | 72535282 | A | G | 0.252 | -0.025 | 0.004 | 1.43e-09 | 632802 | 36.6 |
| smoking initiation | rs11926232 | 3 | 55936417 | G | A | 0.048 | 0.041 | 0.008 | 2.21e-07 | 632802 | 26.8 |
| smoking initiation | rs11941714 | 4 | 31202669 | A | G | 0.337 | -0.019 | 0.004 | 3.64e-07 | 632802 | 25.9 |
| smoking initiation | rs12025237 | 1 | 154205120 | C | A | 0.124 | -0.033 | 0.005 | 6.52e-10 | 632802 | 38.2 |
| smoking initiation | rs12042107 | 1 | 91196176 | C | T | 0.527 | -0.022 | 0.004 | 4.22e-10 | 632802 | 39 |
| smoking initiation | rs12047884 | 1 | 112142413 | G | T | 0.515 | -0.018 | 0.004 | 7.53e-07 | 632802 | 24.5 |
| smoking initiation | rs12112638 | 7 | 69735251 | G | A | 0.275 | -0.025 | 0.004 | 1.34e-09 | 632802 | 36.8 |
| smoking initiation | rs12151152 | 19 | 47563532 | G | A | 0.581 | 0.018 | 0.004 | 4.31e-07 | 632802 | 25.6 |
| smoking initiation | rs12186738 | 5 | 103816655 | T | G | 0.154 | -0.033 | 0.005 | 3.42e-11 | 632802 | 43.9 |
| smoking initiation | rs12333760 | 7 | 99185406 | C | T | 0.204 | -0.029 | 0.005 | 1.44e-09 | 632802 | 36.6 |
| smoking initiation | rs12441907 | 15 | 83922387 | A | C | 0.186 | -0.029 | 0.005 | 1.06e-10 | 632802 | 41.7 |
| smoking initiation | rs12474587 | 2 | 162802993 | T | G | 0.404 | 0.028 | 0.004 | 1.25e-14 | 632802 | 59.5 |
| smoking initiation | rs12479064 | 2 | 100046038 | T | C | 0.218 | 0.021 | 0.004 | 2.46e-06 | 632802 | 22.2 |
| smoking initiation | rs12545053 | 8 | 65073605 | G | A | 0.397 | 0.02 | 0.004 | 2.43e-08 | 632802 | 31.1 |
| smoking initiation | rs12632110 | 3 | 50224225 | G | A | 0.647 | -0.023 | 0.004 | 4.78e-10 | 632802 | 38.8 |
| smoking initiation | rs12714017 | 2 | 80999398 | C | T | 0.5 | 0.019 | 0.004 | 1.89e-07 | 632802 | 27.1 |
| smoking initiation | rs13007361 | 2 | 166250244 | A | G | 0.199 | 0.022 | 0.004 | 5.45e-07 | 632802 | 25.1 |
| smoking initiation | rs13013954 | 2 | 184439743 | C | A | 0.2 | -0.02 | 0.004 | 3.78e-06 | 632802 | 21.4 |
| smoking initiation | rs13030994 | 2 | 146143090 | A | G | 0.485 | 0.036 | 0.004 | 3.56e-24 | 632802 | 103 |
| smoking initiation | rs13250170 | 8 | 93035331 | G | A | 0.231 | -0.022 | 0.004 | 3.5e-07 | 632802 | 26 |
| smoking initiation | rs13261666 | 8 | 59814666 | T | G | 0.522 | -0.027 | 0.004 | 3.9e-14 | 632802 | 57.2 |
| smoking initiation | rs13391128 | 2 | 213117204 | G | A | 0.122 | 0.028 | 0.005 | 1.7e-07 | 632802 | 27.4 |
| smoking initiation | rs134529 | 22 | 28781758 | C | T | 0.349 | -0.02 | 0.004 | 4.85e-08 | 632802 | 29.8 |
| smoking initiation | rs1385108 | 5 | 154839646 | T | C | 0.239 | 0.025 | 0.004 | 3e-09 | 632802 | 35.2 |
| smoking initiation | rs1392510 | 9 | 8296740 | C | T | 0.148 | 0.028 | 0.005 | 6.84e-08 | 632802 | 29.1 |
| smoking initiation | rs1393450 | 3 | 173402170 | G | T | 0.221 | 0.02 | 0.004 | 2.71e-06 | 632802 | 22 |
| smoking initiation | rs1414909 | 10 | 82815674 | A | G | 0.378 | 0.018 | 0.004 | 1.19e-06 | 632802 | 23.6 |
| smoking initiation | rs1435741 | 15 | 47935843 | A | G | 0.425 | 0.029 | 0.004 | 2.64e-16 | 632802 | 67.1 |
| smoking initiation | rs1445649 | 2 | 155682556 | C | T | 0.525 | 0.024 | 0.004 | 1.68e-11 | 632802 | 45.3 |
| smoking initiation | rs145151226 | 3 | 109195284 | A | C | 0.028 | 0.048 | 0.01 | 2.92e-06 | 632802 | 21.9 |
| smoking initiation | rs1471093 | 3 | 108031094 | A | G | 0.597 | 0.018 | 0.004 | 1.1e-06 | 632802 | 23.7 |
| smoking initiation | rs147662818 | 10 | 126634762 | T | C | 0.009 | 0.102 | 0.019 | 7.25e-08 | 632802 | 29 |
| smoking initiation | rs1485272 | 3 | 3727589 | C | T | 0.355 | -0.018 | 0.004 | 1.48e-06 | 632802 | 23.2 |
| smoking initiation | rs1533772 | 1 | 237844948 | G | A | 0.358 | -0.021 | 0.004 | 1.11e-07 | 632802 | 28.2 |
| smoking initiation | rs1569916 | 20 | 54392436 | T | C | 0.561 | -0.02 | 0.004 | 5.13e-08 | 632802 | 29.7 |
| smoking initiation | rs16896316 | 6 | 65832814 | G | A | 0.192 | 0.022 | 0.005 | 1.24e-06 | 632802 | 23.5 |
| smoking initiation | rs16975171 | 18 | 39269650 | A | C | 0.082 | -0.032 | 0.007 | 2.61e-06 | 632802 | 22.1 |
| smoking initiation | rs17016894 | 3 | 25672778 | T | C | 0.298 | -0.018 | 0.004 | 4.76e-06 | 632802 | 20.9 |
| smoking initiation | rs1714521 | 3 | 158284861 | C | A | 0.402 | -0.02 | 0.004 | 6.33e-08 | 632802 | 29.3 |
| smoking initiation | rs17165769 | 5 | 107365642 | G | A | 0.389 | 0.017 | 0.004 | 3.08e-06 | 632802 | 21.8 |
| smoking initiation | rs17692129 | 17 | 44793283 | T | C | 0.321 | 0.02 | 0.004 | 1.32e-07 | 632802 | 27.8 |
| smoking initiation | rs1776631 | 10 | 31422577 | C | T | 0.796 | -0.021 | 0.004 | 1.78e-06 | 632802 | 22.8 |
| smoking initiation | rs1834306 | 11 | 122023187 | G | A | 0.576 | -0.016 | 0.004 | 4.83e-06 | 632802 | 20.9 |
| smoking initiation | rs1869243 | 3 | 5724536 | C | T | 0.481 | 0.02 | 0.004 | 2.97e-08 | 632802 | 30.7 |
| smoking initiation | rs1899896 | 8 | 93201036 | T | C | 0.286 | 0.026 | 0.004 | 1.04e-11 | 632802 | 46.3 |
| smoking initiation | rs1921087 | 5 | 30839607 | C | A | 0.502 | 0.019 | 0.004 | 5.62e-08 | 632802 | 29.5 |
| smoking initiation | rs1971318 | 12 | 121389500 | T | C | 0.141 | 0.029 | 0.005 | 7.06e-09 | 632802 | 33.5 |
| smoking initiation | rs2010921 | 11 | 132098205 | A | G | 0.305 | 0.019 | 0.004 | 1.07e-06 | 632802 | 23.8 |
| smoking initiation | rs2046850 | 1 | 210304319 | T | C | 0.187 | -0.025 | 0.004 | 3.03e-08 | 632802 | 30.7 |
| smoking initiation | rs2072155 | 7 | 77762457 | C | T | 0.697 | 0.019 | 0.004 | 5.47e-07 | 632802 | 25.1 |
| smoking initiation | rs2074118 | 7 | 111395987 | G | A | 0.7 | -0.02 | 0.004 | 6.22e-07 | 632802 | 24.8 |
| smoking initiation | rs2286664 | 15 | 67701495 | A | G | 0.728 | 0.019 | 0.004 | 1.92e-06 | 632802 | 22.7 |
| smoking initiation | rs2378662 | 9 | 86707289 | A | G | 0.556 | 0.021 | 0.004 | 4.16e-09 | 632802 | 34.5 |
| smoking initiation | rs240963 | 6 | 111644332 | C | T | 0.836 | -0.041 | 0.005 | 2.16e-17 | 632802 | 72 |
| smoking initiation | rs2547030 | 16 | 24853918 | A | G | 0.639 | 0.017 | 0.004 | 4.71e-06 | 632802 | 21 |
| smoking initiation | rs2551456 | 18 | 53702160 | A | C | 0.451 | 0.018 | 0.004 | 5.5e-07 | 632802 | 25.1 |
| smoking initiation | rs2595255 | 3 | 159035620 | T | G | 0.395 | -0.017 | 0.004 | 1.4e-06 | 632802 | 23.3 |
| smoking initiation | rs2622167 | 7 | 153486704 | A | G | 0.416 | 0.017 | 0.004 | 2.15e-06 | 632802 | 22.5 |
| smoking initiation | rs2631024 | 8 | 91995577 | G | A | 0.737 | -0.023 | 0.004 | 1.18e-08 | 632802 | 32.5 |
| smoking initiation | rs266047 | 2 | 104088751 | A | G | 0.529 | -0.031 | 0.004 | 3.36e-16 | 632802 | 66.599 |
| smoking initiation | rs2678897 | 2 | 58169418 | A | G | 0.625 | 0.02 | 0.004 | 3.51e-08 | 632802 | 30.4 |
| smoking initiation | rs2710634 | 2 | 32808804 | C | T | 0.505 | -0.018 | 0.004 | 3.07e-07 | 632802 | 26.2 |
| smoking initiation | rs2958853 | 11 | 41435765 | A | G | 0.475 | -0.018 | 0.004 | 6.4e-07 | 632802 | 24.8 |
| smoking initiation | rs2960029 | 18 | 27796551 | A | G | 0.238 | 0.02 | 0.004 | 1.49e-06 | 632802 | 23.2 |
| smoking initiation | rs3001723 | 1 | 44037685 | A | G | 0.321 | 0.034 | 0.004 | 8.12e-18 | 632802 | 73.9 |
| smoking initiation | rs301805 | 1 | 8481016 | G | T | 0.559 | 0.021 | 0.004 | 2.8e-09 | 632802 | 35.3 |
| smoking initiation | rs3213876 | 18 | 73183978 | C | T | 0.301 | 0.019 | 0.004 | 2.24e-07 | 632802 | 26.8 |
| smoking initiation | rs324768 | 12 | 83969100 | G | A | 0.363 | -0.018 | 0.004 | 6.92e-07 | 632802 | 24.6 |
| smoking initiation | rs34940743 | 14 | 80102233 | G | A | 0.336 | 0.019 | 0.004 | 2.15e-07 | 632802 | 26.9 |
| smoking initiation | rs35702515 | 2 | 137542847 | T | G | 0.162 | 0.025 | 0.004 | 2.43e-09 | 632802 | 35.6 |
| smoking initiation | rs35887108 | 3 | 143780632 | T | C | 0.261 | -0.021 | 0.004 | 9.74e-07 | 632802 | 24 |
| smoking initiation | rs3800227 | 6 | 108994161 | G | A | 0.701 | 0.023 | 0.004 | 1.93e-08 | 632802 | 31.6 |
| smoking initiation | rs3801289 | 7 | 96638267 | C | A | 0.351 | -0.022 | 0.004 | 3.74e-09 | 632802 | 34.8 |
| smoking initiation | rs3811038 | 2 | 113240183 | C | T | 0.272 | 0.019 | 0.004 | 1.58e-06 | 632802 | 23.1 |
| smoking initiation | rs3904512 | 13 | 38357471 | A | G | 0.429 | -0.021 | 0.004 | 3.23e-09 | 632802 | 35 |
| smoking initiation | rs3934797 | 4 | 112467612 | A | G | 0.17 | -0.024 | 0.005 | 1.53e-07 | 632802 | 27.6 |
| smoking initiation | rs4044321 | 5 | 166989513 | G | A | 0.642 | -0.028 | 0.004 | 6.08e-14 | 632802 | 56.3 |
| smoking initiation | rs41264285 | 1 | 155033918 | T | C | 0.195 | 0.023 | 0.004 | 6.76e-08 | 632802 | 29.1 |
| smoking initiation | rs41274959 | 9 | 103068966 | A | G | 0.033 | -0.048 | 0.009 | 1.93e-07 | 632802 | 27.1 |
| smoking initiation | rs4236259 | 7 | 1708080 | G | T | 0.499 | -0.025 | 0.004 | 3.35e-12 | 632802 | 48.5 |
| smoking initiation | rs4352629 | 5 | 87756821 | T | C | 0.492 | -0.028 | 0.004 | 1.22e-14 | 632802 | 59.5 |
| smoking initiation | rs4433590 | 11 | 73310668 | A | G | 0.831 | -0.023 | 0.005 | 1.34e-06 | 632802 | 23.4 |
| smoking initiation | rs4518351 | 5 | 22219503 | G | A | 0.428 | 0.017 | 0.004 | 1.12e-06 | 632802 | 23.7 |
| smoking initiation | rs4523689 | 11 | 7950797 | G | A | 0.408 | -0.021 | 0.004 | 1.55e-08 | 632802 | 32 |
| smoking initiation | rs4543592 | 9 | 3014254 | C | T | 0.468 | 0.022 | 0.004 | 7.46e-10 | 632802 | 37.9 |
| smoking initiation | rs4674993 | 2 | 226332033 | G | A | 0.207 | -0.025 | 0.004 | 1.32e-08 | 632802 | 32.3 |
| smoking initiation | rs4713121 | 6 | 27722064 | C | T | 0.231 | -0.022 | 0.004 | 1.17e-07 | 632802 | 28.1 |
| smoking initiation | rs4714071 | 6 | 37474393 | T | C | 0.511 | -0.018 | 0.004 | 6.95e-07 | 632802 | 24.6 |
| smoking initiation | rs4781977 | 16 | 17572674 | C | T | 0.205 | -0.024 | 0.004 | 4.54e-08 | 632802 | 29.9 |
| smoking initiation | rs4785187 | 16 | 49766772 | A | G | 0.229 | 0.02 | 0.004 | 2.06e-06 | 632802 | 22.5 |
| smoking initiation | rs4785836 | 16 | 65604652 | C | T | 0.398 | -0.02 | 0.004 | 2.26e-08 | 632802 | 31.3 |
| smoking initiation | rs480939 | 11 | 92282413 | A | G | 0.561 | -0.019 | 0.004 | 1.48e-07 | 632802 | 27.6 |
| smoking initiation | rs4822102 | 22 | 42698430 | T | C | 0.613 | -0.018 | 0.004 | 3.97e-07 | 632802 | 25.7 |
| smoking initiation | rs4837631 | 9 | 122061948 | T | C | 0.436 | -0.018 | 0.004 | 4.1e-07 | 632802 | 25.6 |
| smoking initiation | rs4841484 | 8 | 10909202 | A | G | 0.286 | -0.02 | 0.004 | 2.31e-07 | 632802 | 26.8 |
| smoking initiation | rs4949463 | 1 | 32169415 | T | C | 0.141 | 0.027 | 0.005 | 1.13e-06 | 632802 | 23.7 |
| smoking initiation | rs499606 | 6 | 32194308 | T | C | 0.107 | 0.034 | 0.008 | 4.55e-06 | 632802 | 21 |
| smoking initiation | rs501796 | 18 | 7466110 | A | G | 0.517 | 0.017 | 0.004 | 3.13e-06 | 632802 | 21.7 |
| smoking initiation | rs55958435 | 15 | 96852638 | G | A | 0.24 | -0.021 | 0.004 | 1.92e-07 | 632802 | 27.1 |
| smoking initiation | rs55968130 | 16 | 49618174 | A | C | 0.09 | 0.03 | 0.006 | 3.88e-07 | 632802 | 25.8 |
| smoking initiation | rs56169608 | 1 | 18439991 | A | G | 0.45 | 0.017 | 0.004 | 2.82e-06 | 632802 | 21.9 |
| smoking initiation | rs61533748 | 2 | 22582968 | C | T | 0.375 | 0.019 | 0.004 | 1.85e-07 | 632802 | 27.2 |
| smoking initiation | rs61959481 | 13 | 55834929 | A | G | 0.207 | -0.023 | 0.004 | 1.91e-07 | 632802 | 27.1 |
| smoking initiation | rs62074192 | 17 | 16245127 | A | G | 0.511 | -0.016 | 0.004 | 4.95e-06 | 632802 | 20.9 |
| smoking initiation | rs6265 | 11 | 27679916 | T | C | 0.203 | -0.032 | 0.005 | 3.77e-12 | 632802 | 48.2 |
| smoking initiation | rs628891 | 18 | 8381073 | T | C | 0.302 | -0.019 | 0.004 | 3.92e-07 | 632802 | 25.7 |
| smoking initiation | rs6433897 | 2 | 182034448 | C | T | 0.754 | 0.022 | 0.004 | 3.16e-08 | 632802 | 30.6 |
| smoking initiation | rs644400 | 6 | 94174210 | A | G | 0.433 | 0.016 | 0.004 | 4.51e-06 | 632802 | 21 |
| smoking initiation | rs66680800 | 3 | 85985324 | T | G | 0.397 | -0.02 | 0.004 | 2.83e-08 | 632802 | 30.8 |
| smoking initiation | rs6669839 | 1 | 50625979 | T | C | 0.204 | 0.026 | 0.004 | 3.36e-09 | 632802 | 35 |
| smoking initiation | rs6691053 | 1 | 173868955 | T | C | 0.226 | -0.022 | 0.004 | 5.38e-07 | 632802 | 25.1 |
| smoking initiation | rs6728726 | 2 | 623976 | C | T | 0.829 | 0.035 | 0.005 | 6.73e-14 | 632802 | 56.1 |
| smoking initiation | rs6800583 | 3 | 16851755 | A | G | 0.363 | 0.02 | 0.004 | 6.73e-08 | 632802 | 29.1 |
| smoking initiation | rs6893752 | 5 | 60374912 | G | A | 0.766 | -0.024 | 0.004 | 3.25e-09 | 632802 | 35 |
| smoking initiation | rs6914489 | 6 | 97696564 | T | C | 0.74 | 0.02 | 0.004 | 6.5e-07 | 632802 | 24.8 |
| smoking initiation | rs694739 | 11 | 64097233 | G | A | 0.363 | -0.019 | 0.004 | 3.27e-07 | 632802 | 26.1 |
| smoking initiation | rs6956116 | 7 | 122246338 | A | G | 0.338 | 0.017 | 0.004 | 4.4e-06 | 632802 | 21.1 |
| smoking initiation | rs6974377 | 7 | 21397405 | C | T | 0.316 | 0.02 | 0.004 | 1.91e-07 | 632802 | 27.1 |
| smoking initiation | rs7026534 | 9 | 134907263 | G | T | 0.689 | -0.02 | 0.004 | 1.99e-07 | 632802 | 27 |
| smoking initiation | rs71627577 | 5 | 43125795 | G | A | 0.102 | -0.03 | 0.006 | 8.59e-08 | 632802 | 28.7 |
| smoking initiation | rs7197072 | 16 | 717085 | T | C | 0.238 | -0.025 | 0.004 | 2.77e-09 | 632802 | 35.3 |
| smoking initiation | rs7224742 | 17 | 30657058 | T | C | 0.595 | -0.021 | 0.004 | 1.43e-08 | 632802 | 32.1 |
| smoking initiation | rs72730742 | 15 | 78097213 | C | A | 0.09 | -0.031 | 0.006 | 3.89e-07 | 632802 | 25.7 |
| smoking initiation | rs72789632 | 5 | 106834363 | T | C | 0.12 | -0.033 | 0.005 | 5.02e-10 | 632802 | 38.7 |
| smoking initiation | rs72802545 | 10 | 72724102 | C | A | 0.174 | -0.022 | 0.005 | 2.35e-06 | 632802 | 22.3 |
| smoking initiation | rs72976160 | 18 | 77497695 | A | G | 0.166 | 0.023 | 0.004 | 3.87e-07 | 632802 | 25.8 |
| smoking initiation | rs745570 | 17 | 77781725 | G | A | 0.511 | -0.018 | 0.004 | 5.99e-07 | 632802 | 24.9 |
| smoking initiation | rs74876138 | 3 | 171518379 | C | T | 0.234 | 0.02 | 0.004 | 7.42e-07 | 632802 | 24.5 |
| smoking initiation | rs7505855 | 18 | 31696075 | T | C | 0.596 | -0.019 | 0.004 | 1.45e-07 | 632802 | 27.6 |
| smoking initiation | rs7555507 | 1 | 73766037 | T | C | 0.496 | -0.024 | 0.004 | 1.14e-11 | 632802 | 46.1 |
| smoking initiation | rs76214862 | 14 | 29500130 | C | A | 0.202 | -0.025 | 0.005 | 3.99e-08 | 632802 | 30.2 |
| smoking initiation | rs7631379 | 3 | 181409057 | C | T | 0.207 | 0.022 | 0.004 | 6.58e-07 | 632802 | 24.7 |
| smoking initiation | rs76608582 | 19 | 4474725 | A | C | 0.039 | -0.05 | 0.008 | 1.94e-09 | 632802 | 36 |
| smoking initiation | rs77075313 | 15 | 63901776 | G | A | 0.045 | -0.043 | 0.009 | 8.15e-07 | 632802 | 24.3 |
| smoking initiation | rs7717387 | 5 | 91255566 | T | C | 0.953 | -0.045 | 0.009 | 6.97e-07 | 632802 | 24.6 |
| smoking initiation | rs77803288 | 14 | 90057852 | T | C | 0.046 | -0.038 | 0.008 | 2.24e-06 | 632802 | 22.4 |
| smoking initiation | rs78648167 | 11 | 87073001 | G | A | 0.083 | -0.029 | 0.006 | 3.65e-06 | 632802 | 21.4 |
| smoking initiation | rs7902527 | 10 | 118715399 | A | G | 0.227 | 0.021 | 0.004 | 7.86e-07 | 632802 | 24.4 |
| smoking initiation | rs79222572 | 6 | 165108555 | G | T | 0.251 | 0.019 | 0.004 | 2.04e-06 | 632802 | 22.6 |
| smoking initiation | rs7929518 | 11 | 85980958 | G | A | 0.765 | 0.024 | 0.004 | 1.56e-08 | 632802 | 32 |
| smoking initiation | rs7938812 | 11 | 112911004 | G | T | 0.424 | 0.044 | 0.004 | 2.71e-33 | 632802 | 144.999 |
| smoking initiation | rs7969559 | 12 | 69655167 | G | A | 0.688 | -0.024 | 0.004 | 7.31e-10 | 632802 | 37.9 |
| smoking initiation | rs7999164 | 13 | 101254933 | A | G | 0.549 | 0.017 | 0.004 | 9.5e-07 | 632802 | 24 |
| smoking initiation | rs8010469 | 14 | 58643368 | G | A | 0.54 | -0.018 | 0.004 | 7.56e-07 | 632802 | 24.5 |
| smoking initiation | rs8018617 | 14 | 98594361 | C | A | 0.384 | 0.02 | 0.004 | 6.58e-08 | 632802 | 29.2 |
| smoking initiation | rs8027457 | 15 | 99204101 | C | T | 0.524 | 0.019 | 0.004 | 1.2e-07 | 632802 | 28 |
| smoking initiation | rs888817 | 5 | 80257798 | A | G | 0.63 | 0.018 | 0.004 | 6.96e-07 | 632802 | 24.6 |
| smoking initiation | rs9388686 | 6 | 129425564 | C | A | 0.815 | 0.023 | 0.005 | 6.14e-07 | 632802 | 24.9 |
| smoking initiation | rs9401770 | 6 | 98748008 | A | G | 0.273 | 0.028 | 0.004 | 3.47e-12 | 632802 | 48.4 |
| smoking initiation | rs9471935 | 6 | 42840318 | A | G | 0.232 | 0.02 | 0.004 | 1.91e-06 | 632802 | 22.7 |
| smoking initiation | rs9603051 | 13 | 37093427 | G | A | 0.513 | 0.017 | 0.004 | 1.78e-06 | 632802 | 22.8 |
| smoking initiation | rs962625 | 4 | 28473524 | G | A | 0.24 | 0.024 | 0.004 | 4.37e-09 | 632802 | 34.5 |
| smoking initiation | rs993700 | 4 | 67825894 | C | T | 0.766 | -0.026 | 0.004 | 1.53e-09 | 632802 | 36.5 |
| Current tobacco smoking | rs10042227 | 5 | 125432585 | C | A | 0.839 | -0.007 | 0.002 | 3.69999e-06 | 462434 | 21.396 |
| Current tobacco smoking | rs1009181 | 6 | 26158993 | C | T | 0.378 | -0.006 | 0.001 | 5.19996e-07 | 462434 | 25.187 |
| Current tobacco smoking | rs10134246 | 14 | 80356531 | A | G | 0.909 | 0.012 | 0.002 | 7.29995e-09 | 462434 | 33.458 |
| Current tobacco smoking | rs10149845 | 14 | 30177079 | T | C | 0.418 | 0.006 | 0.001 | 6.29999e-08 | 462434 | 29.26 |
| Current tobacco smoking | rs10458563 | 1 | 50612250 | G | A | 0.209 | 0.007 | 0.001 | 1.79999e-06 | 462434 | 22.808 |
| Current tobacco smoking | rs10786717 | 10 | 104625237 | G | A | 0.329 | 0.008 | 0.001 | 1.89998e-10 | 462434 | 40.564 |
| Current tobacco smoking | rs10822059 | 10 | 64527697 | G | T | 0.382 | -0.006 | 0.001 | 2.59998e-06 | 462434 | 22.121 |
| Current tobacco smoking | rs10891481 | 11 | 112830562 | G | A | 0.384 | 0.008 | 0.001 | 5.90065e-13 | 462434 | 51.874 |
| Current tobacco smoking | rs10920259 | 1 | 201797696 | C | T | 0.504 | -0.005 | 0.001 | 1.79999e-06 | 462434 | 22.806 |
| Current tobacco smoking | rs11030382 | 11 | 28604477 | T | C | 0.381 | -0.006 | 0.001 | 4.70002e-07 | 462434 | 25.393 |
| Current tobacco smoking | rs11057005 | 12 | 16748721 | G | A | 0.437 | -0.006 | 0.001 | 4.79999e-07 | 462434 | 25.353 |
| Current tobacco smoking | rs1108059 | 15 | 75338890 | C | A | 0.299 | 0.006 | 0.001 | 2.1e-06 | 462434 | 22.533 |
| Current tobacco smoking | rs11096777 | 4 | 35568903 | C | T | 0.18 | 0.009 | 0.001 | 1.7e-10 | 462434 | 40.735 |
| Current tobacco smoking | rs11138389 | 9 | 82478500 | C | T | 0.454 | -0.005 | 0.001 | 4.20001e-06 | 462434 | 21.168 |
| Current tobacco smoking | rs11210229 | 1 | 73860028 | G | A | 0.616 | -0.006 | 0.001 | 2.90001e-07 | 462434 | 26.313 |
| Current tobacco smoking | rs11255908 | 10 | 8802912 | G | T | 0.257 | 0.007 | 0.001 | 2.39999e-07 | 462434 | 26.665 |
| Current tobacco smoking | rs112704858 | 11 | 115459742 | T | C | 0.151 | 0.007 | 0.002 | 4.49997e-06 | 462434 | 21.056 |
| Current tobacco smoking | rs114895876 | 3 | 127352970 | G | A | 0.013 | 0.027 | 0.005 | 3.29997e-07 | 462434 | 26.089 |
| Current tobacco smoking | rs1149331 | 1 | 7528669 | A | G | 0.523 | 0.006 | 0.001 | 4.49997e-07 | 462434 | 25.48 |
| Current tobacco smoking | rs115302983 | 1 | 227376624 | T | G | 0.064 | 0.011 | 0.002 | 1.79999e-06 | 462434 | 22.823 |
| Current tobacco smoking | rs11584725 | 1 | 71754192 | A | G | 0.465 | -0.005 | 0.001 | 2.19999e-06 | 462434 | 22.395 |
| Current tobacco smoking | rs11596214 | 10 | 106453832 | A | G | 0.407 | -0.006 | 0.001 | 4.09996e-08 | 462434 | 30.105 |
| Current tobacco smoking | rs11693885 | 2 | 60122046 | A | G | 0.447 | 0.005 | 0.001 | 4.60002e-06 | 462434 | 21.005 |
| Current tobacco smoking | rs117382215 | 18 | 63675508 | C | T | 0.015 | 0.022 | 0.005 | 3.40001e-06 | 462434 | 21.56 |
| Current tobacco smoking | rs11928368 | 3 | 85661265 | T | G | 0.328 | -0.006 | 0.001 | 1e-06 | 462434 | 23.842 |
| Current tobacco smoking | rs12081925 | 1 | 214472184 | G | A | 0.582 | 0.006 | 0.001 | 1.6e-06 | 462434 | 23.051 |
| Current tobacco smoking | rs12469094 | 2 | 22967228 | C | A | 0.682 | -0.007 | 0.001 | 2.19999e-08 | 462434 | 31.343 |
| Current tobacco smoking | rs12532196 | 7 | 80241570 | C | T | 0.355 | -0.006 | 0.001 | 1.2e-06 | 462434 | 23.567 |
| Current tobacco smoking | rs12666306 | 7 | 115082406 | G | A | 0.499 | -0.009 | 0.001 | 8.80035e-16 | 462434 | 64.68 |
| Current tobacco smoking | rs12693975 | 2 | 203720745 | A | G | 0.814 | 0.008 | 0.001 | 5e-08 | 462434 | 29.73 |
| Current tobacco smoking | rs13177716 | 5 | 148034502 | A | G | 0.237 | -0.006 | 0.001 | 1.5e-06 | 462434 | 23.208 |
| Current tobacco smoking | rs13179596 | 5 | 154779548 | T | C | 0.249 | 0.006 | 0.001 | 2.69998e-06 | 462434 | 22.006 |
| Current tobacco smoking | rs138938221 | 1 | 46683480 | A | G | 0.062 | -0.011 | 0.002 | 3.79997e-06 | 462434 | 21.35 |
| Current tobacco smoking | rs139915 | 22 | 40720963 | T | C | 0.579 | 0.005 | 0.001 | 3.09999e-06 | 462434 | 21.784 |
| Current tobacco smoking | rs1402940 | 8 | 114116686 | T | G | 0.307 | 0.006 | 0.001 | 3.29997e-06 | 462434 | 21.627 |
| Current tobacco smoking | rs144799604 | 2 | 160674308 | T | C | 0.142 | -0.008 | 0.002 | 9.40005e-07 | 462434 | 24.057 |
| Current tobacco smoking | rs1452787 | 18 | 53207207 | G | A | 0.276 | 0.007 | 0.001 | 2.80001e-08 | 462434 | 30.808 |
| Current tobacco smoking | rs1505864 | 4 | 139731065 | C | T | 0.709 | -0.006 | 0.001 | 4.09996e-06 | 462434 | 21.217 |
| Current tobacco smoking | rs1549212 | 5 | 166996722 | T | C | 0.626 | -0.008 | 0.001 | 1.7e-10 | 462434 | 40.735 |
| Current tobacco smoking | rs1577792 | 6 | 79886077 | G | A | 0.596 | 0.005 | 0.001 | 3.79997e-06 | 462434 | 21.343 |
| Current tobacco smoking | rs159032 | 5 | 94206202 | T | C | 0.246 | 0.007 | 0.001 | 2.19999e-07 | 462434 | 26.884 |
| Current tobacco smoking | rs1603572 | 12 | 84103615 | A | G | 0.596 | 0.005 | 0.001 | 3.2e-06 | 462434 | 21.701 |
| Current tobacco smoking | rs17028559 | 2 | 103900940 | G | A | 0.28 | 0.006 | 0.001 | 2.80001e-06 | 462434 | 21.959 |
| Current tobacco smoking | rs17730481 | 8 | 9276163 | A | G | 0.321 | 0.006 | 0.001 | 1.89998e-06 | 462434 | 22.697 |
| Current tobacco smoking | rs177915 | 1 | 99455856 | C | T | 0.359 | 0.006 | 0.001 | 1.2e-06 | 462434 | 23.579 |
| Current tobacco smoking | rs1931263 | 1 | 96175101 | T | G | 0.49 | 0.006 | 0.001 | 8.09991e-07 | 462434 | 24.327 |
| Current tobacco smoking | rs2035299 | 12 | 98650784 | C | T | 0.432 | 0.006 | 0.001 | 1.2e-06 | 462434 | 23.553 |
| Current tobacco smoking | rs2047502 | 9 | 128178894 | A | C | 0.411 | 0.007 | 0.001 | 7.79992e-10 | 462434 | 37.811 |
| Current tobacco smoking | rs214904 | 11 | 17225436 | T | C | 0.398 | -0.007 | 0.001 | 1.6e-08 | 462434 | 31.948 |
| Current tobacco smoking | rs2153531 | 14 | 69762934 | C | T | 0.621 | 0.006 | 0.001 | 2.99999e-06 | 462434 | 21.807 |
| Current tobacco smoking | rs2463126 | 10 | 122195629 | G | A | 0.939 | -0.013 | 0.002 | 1.5e-07 | 462434 | 27.548 |
| Current tobacco smoking | rs2469756 | 8 | 22360423 | A | G | 0.45 | 0.006 | 0.001 | 7.39997e-07 | 462434 | 24.503 |
| Current tobacco smoking | rs2588978 | 10 | 63559609 | C | T | 0.519 | -0.007 | 0.001 | 1e-08 | 462434 | 32.765 |
| Current tobacco smoking | rs2740776 | 8 | 92003130 | T | C | 0.727 | -0.007 | 0.001 | 1.89998e-08 | 462434 | 31.576 |
| Current tobacco smoking | rs28481902 | 9 | 108904944 | C | T | 0.049 | 0.014 | 0.003 | 1.79999e-07 | 462434 | 27.243 |
| Current tobacco smoking | rs28545614 | 2 | 105994827 | T | C | 0.157 | 0.009 | 0.002 | 1.29999e-08 | 462434 | 32.294 |
| Current tobacco smoking | rs2939755 | 11 | 41443472 | C | T | 0.581 | -0.006 | 0.001 | 1e-07 | 462434 | 28.283 |
| Current tobacco smoking | rs3001723 | 1 | 44037685 | A | G | 0.301 | 0.007 | 0.001 | 5.69994e-09 | 462434 | 33.927 |
| Current tobacco smoking | rs3025316 | 9 | 136459543 | C | T | 0.114 | 0.02 | 0.002 | 5.00035e-28 | 462434 | 120.466 |
| Current tobacco smoking | rs30594 | 7 | 32106805 | T | C | 0.578 | 0.006 | 0.001 | 4.90004e-07 | 462434 | 25.3 |
| Current tobacco smoking | rs3087898 | 2 | 61765074 | A | G | 0.425 | -0.006 | 0.001 | 2.59998e-08 | 462434 | 31.002 |
| Current tobacco smoking | rs329120 | 5 | 133861756 | T | C | 0.419 | -0.007 | 0.001 | 7.69999e-09 | 462434 | 33.359 |
| Current tobacco smoking | rs34466545 | 20 | 42079587 | A | G | 0.341 | -0.006 | 0.001 | 1.40001e-06 | 462434 | 23.296 |
| Current tobacco smoking | rs34488670 | 15 | 47684936 | C | T | 0.211 | 0.01 | 0.001 | 2.70023e-12 | 462434 | 48.905 |
| Current tobacco smoking | rs34853150 | 10 | 87339122 | A | G | 0.159 | 0.007 | 0.002 | 1.5e-06 | 462434 | 23.165 |
| Current tobacco smoking | rs35120974 | 5 | 50977800 | G | A | 0.417 | 0.006 | 0.001 | 5.49997e-08 | 462434 | 29.523 |
| Current tobacco smoking | rs35248 | 5 | 68060579 | C | T | 0.136 | 0.008 | 0.002 | 3.2e-06 | 462434 | 21.673 |
| Current tobacco smoking | rs35924491 | 7 | 96712514 | C | T | 0.104 | -0.009 | 0.002 | 2.30001e-06 | 462434 | 22.365 |
| Current tobacco smoking | rs3742365 | 14 | 104198251 | C | T | 0.405 | 0.007 | 0.001 | 9.49992e-09 | 462434 | 32.932 |
| Current tobacco smoking | rs3773814 | 3 | 130715748 | C | A | 0.152 | 0.008 | 0.002 | 5.80003e-07 | 462434 | 24.979 |
| Current tobacco smoking | rs385076 | 2 | 32489851 | C | T | 0.644 | -0.006 | 0.001 | 7.19996e-08 | 462434 | 29 |
| Current tobacco smoking | rs4321249 | 17 | 7795529 | A | G | 0.42 | -0.006 | 0.001 | 1.29999e-06 | 462434 | 23.475 |
| Current tobacco smoking | rs4385321 | 6 | 153394728 | A | G | 0.342 | 0.006 | 0.001 | 2.59998e-06 | 462434 | 22.088 |
| Current tobacco smoking | rs453353 | 3 | 136920208 | T | C | 0.727 | 0.006 | 0.001 | 4.39997e-06 | 462434 | 21.086 |
| Current tobacco smoking | rs4543592 | 9 | 3014254 | C | T | 0.48 | 0.006 | 0.001 | 3.40001e-08 | 462434 | 30.469 |
| Current tobacco smoking | rs4832280 | 2 | 86595367 | T | C | 0.395 | 0.006 | 0.001 | 6.19998e-08 | 462434 | 29.3 |
| Current tobacco smoking | rs4955411 | 3 | 49145304 | G | A | 0.779 | -0.008 | 0.001 | 4.09996e-08 | 462434 | 30.107 |
| Current tobacco smoking | rs4969249 | 17 | 79085094 | A | G | 0.045 | -0.013 | 0.003 | 1.5e-06 | 462434 | 23.179 |
| Current tobacco smoking | rs4976076 | 5 | 68321092 | T | C | 0.428 | -0.005 | 0.001 | 1.89998e-06 | 462434 | 22.702 |
| Current tobacco smoking | rs55649572 | 7 | 150575940 | T | C | 0.463 | -0.005 | 0.001 | 3.69999e-06 | 462434 | 21.401 |
| Current tobacco smoking | rs55655049 | 15 | 78042708 | T | C | 0.235 | -0.007 | 0.001 | 8.09991e-07 | 462434 | 24.331 |
| Current tobacco smoking | rs55748187 | 22 | 31265236 | G | A | 0.079 | 0.011 | 0.002 | 2.30001e-07 | 462434 | 26.722 |
| Current tobacco smoking | rs56113850 | 19 | 41353107 | C | T | 0.578 | -0.013 | 0.001 | 4.19952e-28 | 462434 | 120.822 |
| Current tobacco smoking | rs56133648 | 20 | 36195582 | T | C | 0.234 | 0.007 | 0.001 | 1.6e-06 | 462434 | 23.063 |
| Current tobacco smoking | rs6050215 | 20 | 24948737 | T | C | 0.969 | 0.017 | 0.003 | 3.89996e-07 | 462434 | 25.744 |
| Current tobacco smoking | rs60616188 | 17 | 27413115 | T | C | 0.153 | 0.008 | 0.002 | 3.2e-07 | 462434 | 26.109 |
| Current tobacco smoking | rs62107261 | 2 | 422144 | C | T | 0.048 | -0.013 | 0.003 | 1.29999e-06 | 462434 | 23.494 |
| Current tobacco smoking | rs621125 | 11 | 82908161 | T | G | 0.668 | 0.006 | 0.001 | 4.70002e-07 | 462434 | 25.378 |
| Current tobacco smoking | rs62175972 | 2 | 161362830 | C | T | 0.034 | -0.016 | 0.003 | 5.19996e-07 | 462434 | 25.191 |
| Current tobacco smoking | rs627550 | 1 | 57746538 | C | T | 0.452 | -0.005 | 0.001 | 1.6e-06 | 462434 | 22.984 |
| Current tobacco smoking | rs6466488 | 7 | 114145525 | A | G | 0.594 | 0.006 | 0.001 | 8.9e-08 | 462434 | 28.6 |
| Current tobacco smoking | rs6534338 | 4 | 123026869 | C | T | 0.704 | 0.007 | 0.001 | 5.39995e-08 | 462434 | 29.583 |
| Current tobacco smoking | rs6727997 | 2 | 146346285 | G | A | 0.654 | 0.007 | 0.001 | 8.99995e-10 | 462434 | 37.521 |
| Current tobacco smoking | rs6813082 | 4 | 36531464 | C | T | 0.415 | -0.005 | 0.001 | 3.59998e-06 | 462434 | 21.472 |
| Current tobacco smoking | rs6821949 | 4 | 60424834 | A | G | 0.191 | 0.007 | 0.001 | 1.5e-06 | 462434 | 23.105 |
| Current tobacco smoking | rs6894833 | 5 | 137692821 | G | A | 0.363 | -0.006 | 0.001 | 3.2e-06 | 462434 | 21.688 |
| Current tobacco smoking | rs6962665 | 7 | 110949566 | A | C | 0.295 | 0.006 | 0.001 | 1.5e-06 | 462434 | 23.176 |
| Current tobacco smoking | rs6963853 | 7 | 1858725 | A | G | 0.429 | 0.005 | 0.001 | 2.1e-06 | 462434 | 22.508 |
| Current tobacco smoking | rs701836 | 10 | 102767117 | A | C | 0.24 | -0.006 | 0.001 | 4.90004e-06 | 462434 | 20.866 |
| Current tobacco smoking | rs705308 | 7 | 97695363 | A | C | 0.473 | 0.006 | 0.001 | 1.40001e-07 | 462434 | 27.662 |
| Current tobacco smoking | rs71367544 | 18 | 77574374 | T | C | 0.21 | 0.006 | 0.001 | 4.70002e-06 | 462434 | 20.959 |
| Current tobacco smoking | rs7155595 | 14 | 77502546 | C | A | 0.326 | 0.007 | 0.001 | 1.2e-08 | 462434 | 32.482 |
| Current tobacco smoking | rs7168275 | 15 | 59033972 | G | A | 0.158 | -0.008 | 0.002 | 1.6e-06 | 462434 | 23.06 |
| Current tobacco smoking | rs7206524 | 16 | 69975896 | T | G | 0.314 | 0.006 | 0.001 | 2.69998e-06 | 462434 | 22.031 |
| Current tobacco smoking | rs72635666 | 8 | 49167054 | T | C | 0.061 | 0.011 | 0.002 | 2.39999e-06 | 462434 | 22.245 |
| Current tobacco smoking | rs72757016 | 1 | 226188551 | G | A | 0.124 | -0.008 | 0.002 | 2.5e-06 | 462434 | 22.173 |
| Current tobacco smoking | rs73028396 | 12 | 1579408 | T | C | 0.029 | 0.016 | 0.003 | 4.20001e-06 | 462434 | 21.173 |
| Current tobacco smoking | rs74963256 | 20 | 51588847 | C | T | 0.094 | -0.01 | 0.002 | 4.79999e-07 | 462434 | 25.35 |
| Current tobacco smoking | rs752894 | 16 | 49769636 | G | A | 0.325 | 0.006 | 0.001 | 1e-07 | 462434 | 28.315 |
| Current tobacco smoking | rs7569203 | 2 | 45154418 | C | A | 0.311 | 0.007 | 0.001 | 2e-08 | 462434 | 31.472 |
| Current tobacco smoking | rs76239555 | 4 | 32300756 | G | A | 0.024 | 0.021 | 0.004 | 2.90001e-07 | 462434 | 26.326 |
| Current tobacco smoking | rs7689379 | 4 | 93481256 | G | T | 0.36 | 0.006 | 0.001 | 2.39999e-07 | 462434 | 26.696 |
| Current tobacco smoking | rs7689452 | 4 | 147945733 | G | A | 0.314 | -0.007 | 0.001 | 1.6e-09 | 462434 | 36.456 |
| Current tobacco smoking | rs77463368 | 7 | 133939461 | C | T | 0.056 | 0.013 | 0.002 | 2.1e-07 | 462434 | 26.941 |
| Current tobacco smoking | rs7778443 | 7 | 32314690 | C | T | 0.613 | -0.006 | 0.001 | 1.89998e-06 | 462434 | 22.724 |
| Current tobacco smoking | rs7807019 | 7 | 117543063 | G | A | 0.46 | 0.007 | 0.001 | 6.89922e-11 | 462434 | 42.549 |
| Current tobacco smoking | rs7849527 | 9 | 78014740 | G | A | 0.529 | -0.005 | 0.001 | 3.59998e-06 | 462434 | 21.446 |
| Current tobacco smoking | rs8031550 | 15 | 80956407 | A | G | 0.24 | -0.008 | 0.001 | 2.1e-09 | 462434 | 35.86 |
| Current tobacco smoking | rs928023 | 13 | 70668843 | G | A | 0.155 | -0.007 | 0.002 | 4.09996e-06 | 462434 | 21.203 |
| Current tobacco smoking | rs9607805 | 22 | 41854446 | T | C | 0.726 | 0.008 | 0.001 | 1.2e-10 | 462434 | 41.437 |
| Current tobacco smoking | rs966509 | 18 | 75304495 | C | T | 0.738 | 0.006 | 0.001 | 4.60002e-06 | 462434 | 21.012 |
| Smoking status: Never | rs1004787 | 2 | 45159091 | A | G | 0.53 | -0.009 | 0.001 | 1.58416e-13 | 359706 | 54.467 |
| Smoking status: Never | rs10193706 | 2 | 146316319 | C | A | 0.525 | -0.012 | 0.001 | 8.25468e-24 | 359706 | 101.228 |
| Smoking status: Never | rs10233018 | 7 | 117523709 | G | A | 0.504 | -0.008 | 0.001 | 4.5941e-11 | 359706 | 43.345 |
| Smoking status: Never | rs1029986 | 2 | 60526817 | T | C | 0.585 | -0.007 | 0.001 | 4.26521e-09 | 359706 | 34.5 |
| Smoking status: Never | rs10458563 | 1 | 50612250 | G | A | 0.21 | -0.008 | 0.001 | 1.4668e-07 | 359706 | 27.633 |
| Smoking status: Never | rs10515748 | 5 | 156805286 | G | A | 0.141 | 0.008 | 0.002 | 3.81294e-06 | 359706 | 21.357 |
| Smoking status: Never | rs10774625 | 12 | 111910219 | G | A | 0.504 | 0.007 | 0.001 | 1.12463e-10 | 359706 | 41.594 |
| Smoking status: Never | rs10813628 | 9 | 3176796 | C | T | 0.515 | -0.007 | 0.001 | 3.99991e-09 | 359706 | 34.625 |
| Smoking status: Never | rs10868174 | 9 | 87129942 | A | C | 0.062 | -0.012 | 0.002 | 9.64806e-07 | 359706 | 23.998 |
| Smoking status: Never | rs10897561 | 11 | 79898660 | C | T | 0.356 | -0.007 | 0.001 | 4.49738e-08 | 359706 | 29.923 |
| Smoking status: Never | rs10905461 | 10 | 8803551 | C | T | 0.744 | 0.007 | 0.001 | 4.76354e-08 | 359706 | 29.812 |
| Smoking status: Never | rs10914684 | 1 | 33795572 | A | G | 0.324 | 0.008 | 0.001 | 4.57236e-10 | 359706 | 38.854 |
| Smoking status: Never | rs10956808 | 8 | 92775372 | G | T | 0.422 | 0.008 | 0.001 | 1.03729e-10 | 359706 | 41.752 |
| Smoking status: Never | rs10959442 | 9 | 10993737 | G | T | 0.467 | -0.006 | 0.001 | 7.07848e-07 | 359706 | 24.594 |
| Smoking status: Never | rs10984475 | 9 | 122072014 | A | C | 0.452 | 0.006 | 0.001 | 2.12902e-06 | 359706 | 22.476 |
| Smoking status: Never | rs11048375 | 12 | 26159274 | C | T | 0.645 | 0.006 | 0.001 | 2.72471e-06 | 359706 | 22.002 |
| Smoking status: Never | rs1109480 | 12 | 121083279 | A | G | 0.39 | 0.007 | 0.001 | 5.09038e-08 | 359706 | 29.683 |
| Smoking status: Never | rs11103667 | 9 | 137978360 | T | C | 0.192 | -0.009 | 0.001 | 7.02571e-09 | 359706 | 33.529 |
| Smoking status: Never | rs11123422 | 2 | 118238488 | G | A | 0.503 | -0.005 | 0.001 | 3.40738e-06 | 359706 | 21.573 |
| Smoking status: Never | rs11127913 | 3 | 85987108 | C | T | 0.39 | 0.008 | 0.001 | 6.18443e-12 | 359706 | 47.273 |
| Smoking status: Never | rs111607094 | 4 | 20109368 | T | C | 0.055 | -0.012 | 0.003 | 1.56138e-06 | 359706 | 23.071 |
| Smoking status: Never | rs11172256 | 12 | 57976118 | G | A | 0.257 | -0.007 | 0.001 | 3.14811e-07 | 359706 | 26.158 |
| Smoking status: Never | rs11185172 | 1 | 108286632 | C | T | 0.443 | 0.005 | 0.001 | 4.73424e-06 | 359706 | 20.942 |
| Smoking status: Never | rs11264339 | 1 | 155140648 | T | C | 0.488 | -0.005 | 0.001 | 4.15336e-06 | 359706 | 21.193 |
| Smoking status: Never | rs113924927 | 3 | 82582167 | T | C | 0.051 | 0.014 | 0.003 | 1.17923e-07 | 359706 | 28.056 |
| Smoking status: Never | rs11513957 | 12 | 86435551 | T | C | 0.409 | -0.006 | 0.001 | 8.75588e-07 | 359706 | 24.185 |
| Smoking status: Never | rs11611651 | 12 | 133380790 | A | G | 0.088 | -0.012 | 0.002 | 5.1663e-09 | 359706 | 34.127 |
| Smoking status: Never | rs11617401 | 13 | 111198459 | T | C | 0.112 | -0.009 | 0.002 | 3.09393e-06 | 359706 | 21.758 |
| Smoking status: Never | rs11631530 | 15 | 52100215 | T | C | 0.118 | -0.01 | 0.002 | 2.76701e-08 | 359706 | 30.865 |
| Smoking status: Never | rs11646575 | 16 | 69567781 | A | G | 0.439 | -0.008 | 0.001 | 2.0017e-12 | 359706 | 49.485 |
| Smoking status: Never | rs117009418 | 18 | 63899479 | G | A | 0.031 | 0.016 | 0.003 | 2.01164e-06 | 359706 | 22.585 |
| Smoking status: Never | rs11721059 | 3 | 5725560 | T | C | 0.469 | -0.006 | 0.001 | 5.86098e-07 | 359706 | 24.958 |
| Smoking status: Never | rs1174864 | 7 | 53127559 | A | G | 0.551 | -0.006 | 0.001 | 2.54162e-07 | 359706 | 26.571 |
| Smoking status: Never | rs117991307 | 6 | 158947672 | C | T | 0.056 | 0.012 | 0.003 | 8.59053e-07 | 359706 | 24.221 |
| Smoking status: Never | rs118015114 | 11 | 91818105 | A | G | 0.02 | 0.019 | 0.004 | 3.95776e-06 | 359706 | 21.286 |
| Smoking status: Never | rs11872397 | 18 | 72535282 | A | G | 0.26 | 0.007 | 0.001 | 1.86067e-07 | 359706 | 27.174 |
| Smoking status: Never | rs1190234 | 14 | 103398706 | A | G | 0.149 | -0.008 | 0.002 | 3.7608e-06 | 359706 | 21.384 |
| Smoking status: Never | rs11943397 | 4 | 143617304 | C | T | 0.628 | -0.006 | 0.001 | 4.48229e-07 | 359706 | 25.475 |
| Smoking status: Never | rs12450028 | 17 | 2207425 | T | C | 0.345 | -0.007 | 0.001 | 7.32437e-09 | 359706 | 33.448 |
| Smoking status: Never | rs12479064 | 2 | 100046038 | T | C | 0.208 | -0.008 | 0.001 | 4.36034e-08 | 359706 | 29.983 |
| Smoking status: Never | rs12487411 | 3 | 34422170 | A | G | 0.472 | 0.008 | 0.001 | 1.18623e-10 | 359706 | 41.49 |
| Smoking status: Never | rs12517438 | 5 | 30842054 | G | T | 0.543 | -0.006 | 0.001 | 3.75889e-07 | 359706 | 25.815 |
| Smoking status: Never | rs12596316 | 16 | 82646152 | G | A | 0.448 | 0.006 | 0.001 | 1.00187e-06 | 359706 | 23.925 |
| Smoking status: Never | rs12608052 | 18 | 49803160 | T | C | 0.52 | 0.007 | 0.001 | 6.47635e-09 | 359706 | 33.687 |
| Smoking status: Never | rs12693900 | 2 | 200616368 | T | C | 0.904 | -0.009 | 0.002 | 1.66039e-06 | 359706 | 22.953 |
| Smoking status: Never | rs1271272 | 2 | 207955477 | A | G | 0.312 | 0.007 | 0.001 | 1.75194e-07 | 359706 | 27.29 |
| Smoking status: Never | rs12737625 | 1 | 190887818 | G | A | 0.278 | -0.006 | 0.001 | 2.91212e-06 | 359706 | 21.874 |
| Smoking status: Never | rs12770479 | 10 | 13537204 | A | G | 0.406 | 0.006 | 0.001 | 1.45415e-07 | 359706 | 27.65 |
| Smoking status: Never | rs12895462 | 14 | 77615441 | C | T | 0.192 | 0.007 | 0.001 | 7.79076e-07 | 359706 | 24.41 |
| Smoking status: Never | rs12906448 | 15 | 87391845 | G | T | 0.177 | -0.008 | 0.002 | 8.89263e-07 | 359706 | 24.155 |
| Smoking status: Never | rs12910916 | 15 | 47675655 | T | C | 0.212 | -0.009 | 0.001 | 2.17495e-10 | 359706 | 40.305 |
| Smoking status: Never | rs12930834 | 16 | 72951627 | C | A | 0.236 | 0.007 | 0.001 | 1.50796e-07 | 359706 | 27.58 |
| Smoking status: Never | rs13009008 | 2 | 174043233 | G | A | 0.674 | 0.006 | 0.001 | 7.66055e-07 | 359706 | 24.442 |
| Smoking status: Never | rs13035244 | 2 | 25134009 | C | T | 0.452 | -0.006 | 0.001 | 1.7447e-06 | 359706 | 22.858 |
| Smoking status: Never | rs13357015 | 5 | 80263403 | A | G | 0.636 | -0.007 | 0.001 | 7.89333e-08 | 359706 | 28.833 |
| Smoking status: Never | rs1381274 | 14 | 98655131 | T | C | 0.463 | -0.006 | 0.001 | 4.52064e-07 | 359706 | 25.459 |
| Smoking status: Never | rs139896 | 22 | 38397797 | C | T | 0.649 | -0.006 | 0.001 | 5.87963e-07 | 359706 | 24.952 |
| Smoking status: Never | rs144241237 | 15 | 74090143 | A | G | 0.015 | 0.024 | 0.005 | 2.00932e-06 | 359706 | 22.587 |
| Smoking status: Never | rs147052174 | 1 | 179783167 | T | G | 0.019 | -0.02 | 0.004 | 1.89942e-06 | 359706 | 22.695 |
| Smoking status: Never | rs1499982 | 3 | 117820386 | T | C | 0.851 | -0.011 | 0.002 | 3.00193e-12 | 359706 | 48.691 |
| Smoking status: Never | rs151181409 | 8 | 82623602 | A | G | 0.018 | -0.022 | 0.005 | 1.32407e-06 | 359706 | 23.389 |
| Smoking status: Never | rs1551850 | 3 | 178197021 | A | G | 0.71 | 0.006 | 0.001 | 4.57193e-06 | 359706 | 21.009 |
| Smoking status: Never | rs16835705 | 1 | 237908911 | A | G | 0.286 | 0.007 | 0.001 | 3.23869e-07 | 359706 | 26.103 |
| Smoking status: Never | rs16951001 | 15 | 67854241 | T | G | 0.419 | -0.007 | 0.001 | 2.15581e-08 | 359706 | 31.35 |
| Smoking status: Never | rs16975171 | 18 | 39269650 | A | C | 0.08 | 0.01 | 0.002 | 2.25819e-06 | 359706 | 22.363 |
| Smoking status: Never | rs17003752 | 4 | 80322959 | G | A | 0.139 | 0.01 | 0.002 | 6.58021e-09 | 359706 | 33.657 |
| Smoking status: Never | rs17055603 | 13 | 59454168 | A | G | 0.315 | 0.007 | 0.001 | 1.60247e-07 | 359706 | 27.462 |
| Smoking status: Never | rs17151637 | 8 | 10153082 | T | C | 0.281 | 0.008 | 0.001 | 6.11604e-09 | 359706 | 33.799 |
| Smoking status: Never | rs17207435 | 10 | 107099617 | G | A | 0.058 | -0.012 | 0.002 | 1.74261e-06 | 359706 | 22.86 |
| Smoking status: Never | rs17284251 | 18 | 73207099 | G | A | 0.351 | -0.006 | 0.001 | 4.95268e-06 | 359706 | 20.856 |
| Smoking status: Never | rs17386750 | 3 | 104729457 | A | G | 0.03 | -0.017 | 0.004 | 8.18276e-07 | 359706 | 24.315 |
| Smoking status: Never | rs17723371 | 4 | 149839527 | C | T | 0.089 | -0.01 | 0.002 | 1.84086e-06 | 359706 | 22.755 |
| Smoking status: Never | rs1867368 | 13 | 53903511 | C | T | 0.309 | -0.006 | 0.001 | 1.2738e-06 | 359706 | 23.463 |
| Smoking status: Never | rs187510156 | 11 | 98006982 | C | T | 0.01 | -0.028 | 0.006 | 1.73584e-06 | 359706 | 22.868 |
| Smoking status: Never | rs1899896 | 8 | 93201036 | T | C | 0.298 | -0.008 | 0.001 | 3.55566e-10 | 359706 | 39.345 |
| Smoking status: Never | rs1900861 | 3 | 158925636 | T | C | 0.506 | -0.006 | 0.001 | 2.32001e-06 | 359706 | 22.311 |
| Smoking status: Never | rs2017500 | 15 | 99196112 | A | G | 0.514 | -0.006 | 0.001 | 1.91611e-07 | 359706 | 27.117 |
| Smoking status: Never | rs2092601 | 6 | 131718009 | C | A | 0.532 | -0.005 | 0.001 | 3.27627e-06 | 359706 | 21.648 |
| Smoking status: Never | rs2372435 | 3 | 43505963 | A | G | 0.104 | -0.009 | 0.002 | 4.49904e-06 | 359706 | 21.04 |
| Smoking status: Never | rs2467957 | 8 | 133786056 | G | T | 0.444 | 0.006 | 0.001 | 1.73804e-06 | 359706 | 22.865 |
| Smoking status: Never | rs2471739 | 17 | 44080354 | T | C | 0.806 | 0.007 | 0.001 | 1.12567e-06 | 359706 | 23.701 |
| Smoking status: Never | rs2675609 | 10 | 63636531 | C | T | 0.627 | 0.008 | 0.001 | 1.36144e-11 | 359706 | 45.727 |
| Smoking status: Never | rs2783130 | 13 | 80170160 | G | A | 0.476 | 0.006 | 0.001 | 6.58112e-08 | 359706 | 29.185 |
| Smoking status: Never | rs2797116 | 1 | 72997261 | C | T | 0.267 | 0.008 | 0.001 | 1.65951e-09 | 359706 | 36.339 |
| Smoking status: Never | rs28666004 | 4 | 96310659 | T | G | 0.539 | -0.005 | 0.001 | 4.56541e-06 | 359706 | 21.012 |
| Smoking status: Never | rs2867749 | 11 | 59233233 | A | C | 0.319 | 0.007 | 0.001 | 2.57632e-08 | 359706 | 31.004 |
| Smoking status: Never | rs28760000 | 5 | 46405411 | G | A | 0.673 | 0.006 | 0.001 | 2.15705e-06 | 359706 | 22.451 |
| Smoking status: Never | rs2958853 | 11 | 41435765 | A | G | 0.479 | 0.006 | 0.001 | 2.89188e-07 | 359706 | 26.322 |
| Smoking status: Never | rs299688 | 1 | 8352039 | T | G | 0.716 | -0.007 | 0.001 | 2.94598e-08 | 359706 | 30.744 |
| Smoking status: Never | rs303948 | 13 | 72366052 | G | A | 0.091 | -0.01 | 0.002 | 1.42348e-06 | 359706 | 23.249 |
| Smoking status: Never | rs326341 | 3 | 107811142 | A | G | 0.476 | 0.007 | 0.001 | 1.7408e-08 | 359706 | 31.766 |
| Smoking status: Never | rs34173129 | 8 | 64460809 | G | T | 0.107 | -0.01 | 0.002 | 2.9893e-07 | 359706 | 26.258 |
| Smoking status: Never | rs34260955 | 6 | 33453111 | T | C | 0.238 | -0.007 | 0.001 | 1.89972e-07 | 359706 | 27.134 |
| Smoking status: Never | rs34638471 | 3 | 146367076 | A | G | 0.348 | 0.006 | 0.001 | 2.02344e-06 | 359706 | 22.573 |
| Smoking status: Never | rs35054527 | 7 | 132302866 | C | T | 0.151 | 0.008 | 0.002 | 5.81835e-07 | 359706 | 24.972 |
| Smoking status: Never | rs35891966 | 11 | 20129311 | A | G | 0.072 | 0.015 | 0.002 | 4.55617e-11 | 359706 | 43.361 |
| Smoking status: Never | rs35892365 | 8 | 26280685 | T | C | 0.246 | 0.007 | 0.001 | 4.87113e-07 | 359706 | 25.315 |
| Smoking status: Never | rs379525 | 12 | 125788119 | T | C | 0.481 | -0.006 | 0.001 | 4.96169e-08 | 359706 | 29.733 |
| Smoking status: Never | rs3796220 | 3 | 191048852 | T | C | 0.207 | -0.007 | 0.001 | 4.07681e-07 | 359706 | 25.658 |
| Smoking status: Never | rs3800227 | 6 | 108994161 | G | A | 0.744 | -0.006 | 0.001 | 1.56556e-06 | 359706 | 23.066 |
| Smoking status: Never | rs3811038 | 2 | 113240183 | C | T | 0.277 | -0.006 | 0.001 | 6.71815e-07 | 359706 | 24.695 |
| Smoking status: Never | rs3896224 | 10 | 106467853 | G | A | 0.412 | 0.006 | 0.001 | 7.02716e-08 | 359706 | 29.058 |
| Smoking status: Never | rs42417 | 5 | 94198290 | T | C | 0.69 | -0.007 | 0.001 | 2.54367e-08 | 359706 | 31.029 |
| Smoking status: Never | rs4310854 | 16 | 65597729 | A | G | 0.396 | 0.006 | 0.001 | 1.75647e-06 | 359706 | 22.845 |
| Smoking status: Never | rs4386836 | 6 | 157744058 | G | A | 0.576 | 0.006 | 0.001 | 8.52511e-08 | 359706 | 28.684 |
| Smoking status: Never | rs4410606 | 5 | 59544262 | C | T | 0.384 | -0.006 | 0.001 | 8.63674e-08 | 359706 | 28.659 |
| Smoking status: Never | rs4499947 | 6 | 144831029 | A | G | 0.398 | -0.006 | 0.001 | 3.83266e-07 | 359706 | 25.777 |
| Smoking status: Never | rs454440 | 20 | 14843670 | A | G | 0.124 | -0.008 | 0.002 | 3.78573e-06 | 359706 | 21.371 |
| Smoking status: Never | rs4566215 | 17 | 50209317 | C | A | 0.533 | 0.007 | 0.001 | 1.62028e-08 | 359706 | 31.905 |
| Smoking status: Never | rs4598829 | 14 | 86881373 | G | A | 0.077 | -0.012 | 0.002 | 8.58994e-08 | 359706 | 28.669 |
| Smoking status: Never | rs4677381 | 3 | 71063373 | G | A | 0.542 | -0.006 | 0.001 | 1.68795e-06 | 359706 | 22.922 |
| Smoking status: Never | rs4679531 | 3 | 60299260 | G | T | 0.382 | -0.006 | 0.001 | 2.38177e-06 | 359706 | 22.26 |
| Smoking status: Never | rs4680392 | 3 | 157404329 | C | T | 0.672 | -0.007 | 0.001 | 7.36461e-08 | 359706 | 28.968 |
| Smoking status: Never | rs4687552 | 3 | 52838402 | C | T | 0.34 | -0.006 | 0.001 | 2.42438e-06 | 359706 | 22.226 |
| Smoking status: Never | rs4737525 | 8 | 59799781 | A | G | 0.507 | 0.006 | 0.001 | 1.8321e-06 | 359706 | 22.764 |
| Smoking status: Never | rs4797080 | 18 | 279300 | C | T | 0.266 | -0.007 | 0.001 | 7.37293e-08 | 359706 | 28.965 |
| Smoking status: Never | rs4812994 | 20 | 44729089 | A | C | 0.53 | 0.006 | 0.001 | 1.02056e-06 | 359706 | 23.89 |
| Smoking status: Never | rs4910656 | 11 | 4662087 | C | T | 0.342 | 0.007 | 0.001 | 2.47474e-08 | 359706 | 31.082 |
| Smoking status: Never | rs4943384 | 13 | 37096833 | C | T | 0.501 | -0.006 | 0.001 | 1.085e-07 | 359706 | 28.217 |
| Smoking status: Never | rs4957528 | 5 | 106420589 | C | A | 0.792 | -0.008 | 0.001 | 4.57889e-09 | 359706 | 34.362 |
| Smoking status: Never | rs522180 | 18 | 77458644 | T | C | 0.353 | -0.006 | 0.001 | 1.55367e-06 | 359706 | 23.081 |
| Smoking status: Never | rs523528 | 11 | 132212816 | T | C | 0.586 | 0.008 | 0.001 | 1.29509e-11 | 359706 | 45.825 |
| Smoking status: Never | rs55745089 | 1 | 88236488 | G | A | 0.094 | -0.01 | 0.002 | 4.53576e-06 | 359706 | 21.024 |
| Smoking status: Never | rs55921136 | 1 | 210359333 | C | T | 0.203 | 0.009 | 0.001 | 2.92981e-09 | 359706 | 35.232 |
| Smoking status: Never | rs56193625 | 1 | 108507209 | A | G | 0.056 | 0.014 | 0.003 | 1.20235e-07 | 359706 | 28.018 |
| Smoking status: Never | rs59357769 | 2 | 135308431 | G | A | 0.179 | -0.008 | 0.002 | 1.02106e-07 | 359706 | 28.335 |
| Smoking status: Never | rs6022263 | 20 | 51681731 | C | T | 0.51 | -0.006 | 0.001 | 2.51229e-06 | 359706 | 22.157 |
| Smoking status: Never | rs6120675 | 20 | 33138870 | C | T | 0.486 | 0.006 | 0.001 | 1.70463e-07 | 359706 | 27.343 |
| Smoking status: Never | rs6141314 | 20 | 31093514 | A | G | 0.242 | -0.008 | 0.001 | 3.39727e-09 | 359706 | 34.943 |
| Smoking status: Never | rs62022630 | 15 | 89932790 | T | C | 0.077 | 0.011 | 0.002 | 1.34837e-06 | 359706 | 23.354 |
| Smoking status: Never | rs62099204 | 18 | 37722425 | T | G | 0.038 | 0.015 | 0.003 | 5.41589e-07 | 359706 | 25.111 |
| Smoking status: Never | rs62180323 | 2 | 63412289 | C | T | 0.234 | 0.006 | 0.001 | 3.46099e-06 | 359706 | 21.543 |
| Smoking status: Never | rs6265 | 11 | 27679916 | T | C | 0.189 | 0.01 | 0.001 | 7.61027e-12 | 359706 | 46.867 |
| Smoking status: Never | rs6433897 | 2 | 182034448 | C | T | 0.737 | -0.007 | 0.001 | 4.2458e-08 | 359706 | 30.035 |
| Smoking status: Never | rs6464024 | 7 | 1688369 | T | C | 0.429 | 0.006 | 0.001 | 7.69042e-08 | 359706 | 28.883 |
| Smoking status: Never | rs6477527 | 9 | 109323457 | A | G | 0.366 | 0.006 | 0.001 | 2.47281e-06 | 359706 | 22.188 |
| Smoking status: Never | rs653953 | 1 | 44083015 | A | G | 0.676 | 0.008 | 0.001 | 5.22516e-11 | 359706 | 43.093 |
| Smoking status: Never | rs6676022 | 1 | 154162503 | T | C | 0.122 | 0.012 | 0.002 | 7.18787e-11 | 359706 | 42.47 |
| Smoking status: Never | rs6689480 | 1 | 32175918 | G | A | 0.12 | -0.009 | 0.002 | 2.09788e-07 | 359706 | 26.942 |
| Smoking status: Never | rs6690680 | 1 | 5701154 | C | T | 0.158 | 0.009 | 0.002 | 3.07872e-08 | 359706 | 30.658 |
| Smoking status: Never | rs6690871 | 1 | 74977277 | G | A | 0.407 | -0.006 | 0.001 | 1.4844e-07 | 359706 | 27.611 |
| Smoking status: Never | rs6716891 | 2 | 37206683 | G | A | 0.331 | -0.007 | 0.001 | 8.07161e-08 | 359706 | 28.79 |
| Smoking status: Never | rs67402191 | 4 | 183528760 | A | G | 0.515 | 0.006 | 0.001 | 6.58173e-07 | 359706 | 24.735 |
| Smoking status: Never | rs7130826 | 11 | 17037793 | G | T | 0.272 | 0.007 | 0.001 | 2.07281e-07 | 359706 | 26.965 |
| Smoking status: Never | rs71491831 | 11 | 124605783 | A | G | 0.076 | 0.011 | 0.002 | 5.52599e-07 | 359706 | 25.072 |
| Smoking status: Never | rs71627577 | 5 | 43125795 | G | A | 0.112 | 0.01 | 0.002 | 8.87646e-08 | 359706 | 28.606 |
| Smoking status: Never | rs72678864 | 4 | 112422145 | A | G | 0.174 | 0.01 | 0.002 | 1.96911e-10 | 359706 | 40.499 |
| Smoking status: Never | rs72686156 | 14 | 49210983 | G | A | 0.016 | -0.023 | 0.005 | 1.6897e-06 | 359706 | 22.92 |
| Smoking status: Never | rs73009376 | 2 | 145864826 | T | C | 0.026 | -0.018 | 0.004 | 3.94657e-07 | 359706 | 25.721 |
| Smoking status: Never | rs7316843 | 12 | 71449811 | T | C | 0.751 | 0.006 | 0.001 | 2.42198e-06 | 359706 | 22.228 |
| Smoking status: Never | rs7333559 | 13 | 100546450 | A | G | 0.79 | 0.008 | 0.001 | 2.05329e-08 | 359706 | 31.445 |
| Smoking status: Never | rs745570 | 17 | 77781725 | G | A | 0.515 | 0.006 | 0.001 | 2.13182e-06 | 359706 | 22.473 |
| Smoking status: Never | rs748828 | 4 | 147879993 | T | C | 0.283 | 0.009 | 0.001 | 2.25684e-11 | 359706 | 44.737 |
| Smoking status: Never | rs7517629 | 1 | 91196099 | G | A | 0.545 | 0.007 | 0.001 | 8.01364e-10 | 359706 | 37.758 |
| Smoking status: Never | rs7528604 | 1 | 66407352 | A | G | 0.433 | 0.007 | 0.001 | 5.37391e-09 | 359706 | 34.05 |
| Smoking status: Never | rs75495938 | 4 | 70511625 | C | T | 0.186 | 0.007 | 0.001 | 7.31021e-07 | 359706 | 24.532 |
| Smoking status: Never | rs7562367 | 2 | 48032613 | A | G | 0.671 | 0.006 | 0.001 | 1.77873e-06 | 359706 | 22.821 |
| Smoking status: Never | rs75641162 | 14 | 30533024 | C | A | 0.046 | -0.013 | 0.003 | 3.80838e-06 | 359706 | 21.359 |
| Smoking status: Never | rs7564248 | 2 | 51379098 | G | A | 0.405 | -0.007 | 0.001 | 1.46083e-08 | 359706 | 32.106 |
| Smoking status: Never | rs7567570 | 2 | 615140 | C | T | 0.827 | -0.009 | 0.002 | 2.58232e-09 | 359706 | 35.477 |
| Smoking status: Never | rs762995 | 22 | 42672124 | G | A | 0.535 | 0.006 | 0.001 | 1.37461e-07 | 359706 | 27.759 |
| Smoking status: Never | rs7630111 | 3 | 114165901 | A | C | 0.753 | -0.006 | 0.001 | 2.71888e-06 | 359706 | 22.006 |
| Smoking status: Never | rs763053 | 16 | 735921 | C | T | 0.225 | 0.008 | 0.001 | 7.91097e-09 | 359706 | 33.298 |
| Smoking status: Never | rs76608582 | 19 | 4474725 | A | C | 0.048 | 0.018 | 0.003 | 1.61154e-10 | 359706 | 40.891 |
| Smoking status: Never | rs7667302 | 4 | 57749811 | T | C | 0.388 | 0.006 | 0.001 | 2.78779e-06 | 359706 | 21.958 |
| Smoking status: Never | rs7679162 | 4 | 31183330 | G | T | 0.339 | 0.006 | 0.001 | 4.74187e-07 | 359706 | 25.367 |
| Smoking status: Never | rs7703284 | 5 | 103782048 | G | A | 0.167 | 0.008 | 0.002 | 1.66752e-07 | 359706 | 27.385 |
| Smoking status: Never | rs77068442 | 1 | 158845316 | G | A | 0.108 | -0.009 | 0.002 | 1.17815e-06 | 359706 | 23.613 |
| Smoking status: Never | rs77217252 | 21 | 40663253 | T | G | 0.127 | -0.009 | 0.002 | 3.31329e-07 | 359706 | 26.059 |
| Smoking status: Never | rs772921 | 12 | 56403577 | T | C | 0.343 | 0.007 | 0.001 | 2.74075e-09 | 359706 | 35.361 |
| Smoking status: Never | rs7733044 | 5 | 107573532 | C | T | 0.369 | 0.006 | 0.001 | 1.30419e-06 | 359706 | 23.418 |
| Smoking status: Never | rs77608903 | 3 | 131142418 | G | T | 0.046 | -0.013 | 0.003 | 4.8568e-06 | 359706 | 20.893 |
| Smoking status: Never | rs78515650 | 6 | 33800340 | C | T | 0.622 | 0.006 | 0.001 | 1.66606e-06 | 359706 | 22.947 |
| Smoking status: Never | rs7870475 | 9 | 128134034 | C | T | 0.474 | -0.007 | 0.001 | 6.33228e-10 | 359706 | 38.218 |
| Smoking status: Never | rs79305858 | 11 | 8527666 | G | A | 0.075 | -0.011 | 0.002 | 5.73416e-07 | 359706 | 25 |
| Smoking status: Never | rs79399187 | 3 | 68932660 | G | A | 0.063 | 0.011 | 0.002 | 4.98701e-06 | 359706 | 20.843 |
| Smoking status: Never | rs7947529 | 11 | 85935945 | T | C | 0.785 | -0.007 | 0.001 | 1.83671e-06 | 359706 | 22.759 |
| Smoking status: Never | rs7948789 | 11 | 112839532 | G | A | 0.386 | -0.016 | 0.001 | 1.33045e-41 | 359706 | 182.616 |
| Smoking status: Never | rs80233585 | 8 | 145688534 | A | G | 0.464 | 0.006 | 0.001 | 1.82155e-07 | 359706 | 27.215 |
| Smoking status: Never | rs8063842 | 16 | 49561498 | A | G | 0.08 | -0.011 | 0.002 | 2.58446e-07 | 359706 | 26.538 |
| Smoking status: Never | rs883403 | 7 | 99047978 | C | T | 0.154 | 0.009 | 0.002 | 4.55187e-09 | 359706 | 34.374 |
| Smoking status: Never | rs9323328 | 14 | 58653514 | G | A | 0.543 | 0.006 | 0.001 | 8.21183e-07 | 359706 | 24.308 |
| Smoking status: Never | rs9375371 | 6 | 98751680 | A | G | 0.269 | -0.007 | 0.001 | 1.7382e-08 | 359706 | 31.768 |
| Smoking status: Never | rs9381917 | 6 | 50911334 | A | G | 0.102 | 0.011 | 0.002 | 4.4727e-09 | 359706 | 34.408 |
| Smoking status: Never | rs9388686 | 6 | 129425564 | C | A | 0.826 | -0.008 | 0.002 | 1.30068e-07 | 359706 | 27.866 |
| Smoking status: Never | rs9487626 | 6 | 111716868 | T | C | 0.818 | 0.013 | 0.002 | 3.06902e-18 | 359706 | 75.852 |
| Smoking status: Never | rs9538536 | 13 | 60536321 | G | T | 0.701 | 0.006 | 0.001 | 4.75905e-06 | 359706 | 20.932 |
| Smoking status: Never | rs9849597 | 3 | 147142757 | G | A | 0.079 | 0.012 | 0.002 | 5.01037e-08 | 359706 | 29.714 |
| Smoking status: Never | rs986969 | 13 | 97103328 | T | G | 0.32 | -0.007 | 0.001 | 8.24423e-08 | 359706 | 28.749 |
| Alcohol intake frequency | rs1022666 | 14 | 29593934 | T | G | 0.864 | -0.026 | 0.005 | 6.60998e-07 | 336965 | 24.726 |
| Alcohol intake frequency | rs10229027 | 7 | 69799863 | C | T | 0.246 | 0.019 | 0.004 | 2.46786e-06 | 336965 | 22.192 |
| Alcohol intake frequency | rs10259230 | 7 | 89635741 | C | T | 0.36 | 0.018 | 0.004 | 1.0616e-06 | 336965 | 23.814 |
| Alcohol intake frequency | rs10454798 | 4 | 67980830 | T | G | 0.247 | 0.022 | 0.004 | 1.70939e-07 | 336965 | 27.338 |
| Alcohol intake frequency | rs11039429 | 11 | 47867059 | T | C | 0.454 | -0.025 | 0.004 | 1.66571e-12 | 336965 | 49.846 |
| Alcohol intake frequency | rs111648099 | 6 | 17112775 | A | G | 0.167 | 0.023 | 0.005 | 1.59698e-06 | 336965 | 23.028 |
| Alcohol intake frequency | rs11221547 | 11 | 99701653 | C | T | 0.177 | -0.023 | 0.005 | 1.02077e-06 | 336965 | 23.889 |
| Alcohol intake frequency | rs11223617 | 11 | 133780757 | A | G | 0.206 | 0.023 | 0.004 | 1.26625e-07 | 336965 | 27.918 |
| Alcohol intake frequency | rs113377562 | 12 | 1898300 | T | G | 0.127 | 0.025 | 0.005 | 3.65183e-06 | 336965 | 21.44 |
| Alcohol intake frequency | rs113929582 | 11 | 65115761 | T | G | 0.051 | -0.047 | 0.008 | 6.92915e-09 | 336965 | 33.556 |
| Alcohol intake frequency | rs116212148 | 3 | 181233686 | G | A | 0.164 | -0.024 | 0.005 | 9.83309e-07 | 336965 | 23.961 |
| Alcohol intake frequency | rs11635606 | 15 | 77819591 | G | A | 0.333 | 0.02 | 0.004 | 1.30005e-07 | 336965 | 27.867 |
| Alcohol intake frequency | rs11646721 | 16 | 9570367 | T | C | 0.076 | 0.031 | 0.007 | 3.87588e-06 | 336965 | 21.326 |
| Alcohol intake frequency | rs11775 | 19 | 13254875 | A | G | 0.681 | 0.018 | 0.004 | 1.91527e-06 | 336965 | 22.679 |
| Alcohol intake frequency | rs11787216 | 8 | 142615222 | T | C | 0.372 | 0.025 | 0.004 | 1.70255e-11 | 336965 | 45.289 |
| Alcohol intake frequency | rs11886864 | 2 | 220156531 | G | A | 0.167 | -0.025 | 0.005 | 1.57605e-07 | 336965 | 27.495 |
| Alcohol intake frequency | rs11940694 | 4 | 39414993 | G | A | 0.605 | -0.043 | 0.004 | 1.75186e-32 | 336965 | 140.86 |
| Alcohol intake frequency | rs12139282 | 1 | 243740714 | A | G | 0.086 | 0.032 | 0.006 | 5.87449e-07 | 336965 | 24.954 |
| Alcohol intake frequency | rs12259464 | 10 | 53680099 | A | G | 0.488 | 0.017 | 0.004 | 2.0296e-06 | 336965 | 22.568 |
| Alcohol intake frequency | rs1228589 | 3 | 131634826 | A | G | 0.246 | 0.02 | 0.004 | 1.44092e-06 | 336965 | 23.226 |
| Alcohol intake frequency | rs1229984 | 4 | 100239319 | C | T | 0.978 | -0.282 | 0.012 | 3.56451e-122 | 336965 | 552.967 |
| Alcohol intake frequency | rs1260326 | 2 | 27730940 | C | T | 0.607 | -0.048 | 0.004 | 7.59976e-40 | 336965 | 174.57 |
| Alcohol intake frequency | rs12613680 | 2 | 186067246 | G | A | 0.367 | 0.018 | 0.004 | 1.69887e-06 | 336965 | 22.909 |
| Alcohol intake frequency | rs12619354 | 2 | 194002782 | C | T | 0.368 | 0.019 | 0.004 | 2.65051e-07 | 336965 | 26.49 |
| Alcohol intake frequency | rs12755107 | 1 | 171420393 | T | C | 0.095 | 0.03 | 0.006 | 5.78496e-07 | 336965 | 24.983 |
| Alcohol intake frequency | rs12770757 | 10 | 125055242 | A | C | 0.15 | 0.026 | 0.005 | 1.6573e-07 | 336965 | 27.397 |
| Alcohol intake frequency | rs12897149 | 14 | 91467876 | G | A | 0.36 | 0.018 | 0.004 | 1.40546e-06 | 336965 | 23.274 |
| Alcohol intake frequency | rs13020444 | 2 | 161333557 | C | T | 0.266 | 0.018 | 0.004 | 4.78619e-06 | 336965 | 20.922 |
| Alcohol intake frequency | rs13031721 | 2 | 61106471 | G | A | 0.372 | -0.018 | 0.004 | 8.85156e-07 | 336965 | 24.164 |
| Alcohol intake frequency | rs13048685 | 21 | 46573848 | C | T | 0.069 | 0.033 | 0.007 | 3.32476e-06 | 336965 | 21.62 |
| Alcohol intake frequency | rs13099797 | 3 | 150664390 | G | A | 0.567 | 0.018 | 0.004 | 7.10035e-07 | 336965 | 24.588 |
| Alcohol intake frequency | rs13102973 | 4 | 135900688 | C | T | 0.622 | -0.021 | 0.004 | 1.59412e-08 | 336965 | 31.937 |
| Alcohol intake frequency | rs13108218 | 4 | 3443931 | G | A | 0.618 | -0.019 | 0.004 | 1.57641e-07 | 336965 | 27.494 |
| Alcohol intake frequency | rs13135092 | 4 | 103198082 | G | A | 0.083 | 0.05 | 0.006 | 1.47877e-14 | 336965 | 59.131 |
| Alcohol intake frequency | rs13231886 | 7 | 44814172 | A | G | 0.402 | -0.02 | 0.004 | 4.9221e-08 | 336965 | 29.748 |
| Alcohol intake frequency | rs13390019 | 2 | 97797680 | C | T | 0.132 | 0.029 | 0.005 | 3.3315e-08 | 336965 | 30.505 |
| Alcohol intake frequency | rs13433863 | 3 | 61699331 | A | G | 0.041 | -0.042 | 0.009 | 3.53102e-06 | 336965 | 21.505 |
| Alcohol intake frequency | rs138993217 | 16 | 69770032 | A | G | 0.169 | 0.025 | 0.005 | 1.65848e-07 | 336965 | 27.396 |
| Alcohol intake frequency | rs139316718 | 12 | 126328572 | T | C | 0.018 | 0.071 | 0.014 | 1.77959e-07 | 336965 | 27.26 |
| Alcohol intake frequency | rs1421085 | 16 | 53800954 | C | T | 0.402 | 0.022 | 0.004 | 1.07169e-09 | 336965 | 37.192 |
| Alcohol intake frequency | rs1490492 | 4 | 172556727 | T | C | 0.664 | 0.02 | 0.004 | 2.08987e-07 | 336965 | 26.949 |
| Alcohol intake frequency | rs1491754 | 3 | 68369396 | A | G | 0.523 | 0.021 | 0.004 | 5.6867e-09 | 336965 | 33.941 |
| Alcohol intake frequency | rs149726000 | 2 | 128874049 | G | A | 0.017 | 0.067 | 0.014 | 1.51259e-06 | 336965 | 23.133 |
| Alcohol intake frequency | rs1521754 | 2 | 139415944 | C | A | 0.693 | 0.019 | 0.004 | 1.67367e-06 | 336965 | 22.938 |
| Alcohol intake frequency | rs1678849 | 19 | 5836964 | A | G | 0.701 | 0.018 | 0.004 | 4.44232e-06 | 336965 | 21.064 |
| Alcohol intake frequency | rs17097556 | 1 | 71582197 | G | A | 0.179 | -0.025 | 0.005 | 4.19141e-08 | 336965 | 30.06 |
| Alcohol intake frequency | rs1727332 | 12 | 123718301 | T | C | 0.756 | 0.02 | 0.004 | 1.3059e-06 | 336965 | 23.415 |
| Alcohol intake frequency | rs17391694 | 1 | 78623626 | T | C | 0.14 | 0.024 | 0.005 | 3.60869e-06 | 336965 | 21.463 |
| Alcohol intake frequency | rs17690703 | 17 | 43925297 | T | C | 0.265 | 0.027 | 0.004 | 3.6425e-11 | 336965 | 43.799 |
| Alcohol intake frequency | rs177393 | 14 | 73716774 | A | G | 0.308 | 0.018 | 0.004 | 4.91157e-06 | 336965 | 20.872 |
| Alcohol intake frequency | rs17796864 | 5 | 145459262 | T | C | 0.296 | -0.019 | 0.004 | 7.33432e-07 | 336965 | 24.526 |
| Alcohol intake frequency | rs1788030 | 18 | 53045198 | T | C | 0.458 | 0.02 | 0.004 | 1.2933e-08 | 336965 | 32.343 |
| Alcohol intake frequency | rs1809931 | 5 | 104026201 | A | G | 0.167 | -0.022 | 0.005 | 4.65661e-06 | 336965 | 20.974 |
| Alcohol intake frequency | rs1870998 | 17 | 72169114 | T | C | 0.161 | 0.022 | 0.005 | 3.95085e-06 | 336965 | 21.289 |
| Alcohol intake frequency | rs1905239 | 12 | 33656636 | A | G | 0.112 | 0.028 | 0.006 | 2.61788e-06 | 336965 | 22.079 |
| Alcohol intake frequency | rs194868 | 7 | 103790469 | C | T | 0.458 | -0.017 | 0.004 | 1.51321e-06 | 336965 | 23.132 |
| Alcohol intake frequency | rs1955058 | 6 | 162360221 | A | G | 0.406 | -0.017 | 0.004 | 4.18524e-06 | 336965 | 21.179 |
| Alcohol intake frequency | rs201440 | 7 | 101735599 | T | C | 0.578 | 0.019 | 0.004 | 1.11946e-07 | 336965 | 28.157 |
| Alcohol intake frequency | rs2049285 | 3 | 182591147 | G | T | 0.672 | 0.018 | 0.004 | 2.51884e-06 | 336965 | 22.153 |
| Alcohol intake frequency | rs2111861 | 8 | 30840591 | A | G | 0.606 | -0.019 | 0.004 | 1.11566e-07 | 336965 | 28.163 |
| Alcohol intake frequency | rs2159935 | 4 | 55521017 | A | G | 0.492 | -0.021 | 0.004 | 5.73548e-09 | 336965 | 33.924 |
| Alcohol intake frequency | rs2181328 | 13 | 49986118 | G | A | 0.712 | -0.023 | 0.004 | 7.70957e-09 | 336965 | 33.348 |
| Alcohol intake frequency | rs248558 | 5 | 144175343 | G | T | 0.504 | -0.022 | 0.004 | 4.77331e-10 | 336965 | 38.77 |
| Alcohol intake frequency | rs2487908 | 1 | 210373952 | G | A | 0.634 | 0.019 | 0.004 | 1.31504e-07 | 336965 | 27.845 |
| Alcohol intake frequency | rs2622167 | 7 | 153486704 | A | G | 0.427 | -0.018 | 0.004 | 1.04561e-06 | 336965 | 23.843 |
| Alcohol intake frequency | rs2717063 | 2 | 58110969 | A | C | 0.584 | -0.019 | 0.004 | 2.27237e-07 | 336965 | 26.787 |
| Alcohol intake frequency | rs2746025 | 17 | 18151611 | A | C | 0.338 | -0.023 | 0.004 | 8.0536e-10 | 336965 | 37.749 |
| Alcohol intake frequency | rs2834003 | 21 | 34287800 | A | C | 0.314 | 0.019 | 0.004 | 1.11512e-06 | 336965 | 23.719 |
| Alcohol intake frequency | rs28525613 | 14 | 32369603 | A | C | 0.152 | 0.025 | 0.005 | 4.79943e-07 | 336965 | 25.344 |
| Alcohol intake frequency | rs28564580 | 17 | 57692689 | G | A | 0.209 | 0.023 | 0.004 | 1.53494e-07 | 336965 | 27.546 |
| Alcohol intake frequency | rs3001426 | 12 | 57509055 | C | T | 0.455 | -0.017 | 0.004 | 1.58023e-06 | 336965 | 23.048 |
| Alcohol intake frequency | rs34638029 | 18 | 27481300 | A | G | 0.033 | 0.051 | 0.01 | 3.71048e-07 | 336965 | 25.84 |
| Alcohol intake frequency | rs34805485 | 9 | 71182471 | A | G | 0.014 | -0.083 | 0.015 | 2.72226e-08 | 336965 | 30.897 |
| Alcohol intake frequency | rs34997 | 5 | 80273341 | G | A | 0.895 | -0.031 | 0.006 | 6.82794e-08 | 336965 | 29.114 |
| Alcohol intake frequency | rs35005436 | 7 | 74134911 | C | T | 0.159 | 0.024 | 0.005 | 6.22401e-07 | 336965 | 24.842 |
| Alcohol intake frequency | rs35105141 | 16 | 30057148 | T | C | 0.402 | 0.025 | 0.004 | 7.20775e-12 | 336965 | 46.973 |
| Alcohol intake frequency | rs35126293 | 1 | 94089946 | C | T | 0.14 | 0.026 | 0.005 | 2.90576e-07 | 336965 | 26.312 |
| Alcohol intake frequency | rs363096 | 4 | 3180021 | C | T | 0.575 | -0.023 | 0.004 | 8.57828e-11 | 336965 | 42.124 |
| Alcohol intake frequency | rs3752252 | 20 | 59829847 | T | C | 0.641 | -0.019 | 0.004 | 5.28762e-07 | 336965 | 25.157 |
| Alcohol intake frequency | rs4057919 | 11 | 124198828 | T | C | 0.287 | 0.018 | 0.004 | 2.683e-06 | 336965 | 22.032 |
| Alcohol intake frequency | rs41277317 | 22 | 24981983 | A | G | 0.013 | -0.077 | 0.016 | 1.76003e-06 | 336965 | 22.841 |
| Alcohol intake frequency | rs4135294 | 3 | 12466715 | A | G | 0.151 | -0.023 | 0.005 | 3.75483e-06 | 336965 | 21.387 |
| Alcohol intake frequency | rs4241258 | 2 | 74226102 | T | C | 0.139 | 0.027 | 0.005 | 2.08684e-07 | 336965 | 26.952 |
| Alcohol intake frequency | rs4242715 | 10 | 133986135 | A | G | 0.683 | -0.019 | 0.004 | 3.42255e-07 | 336965 | 25.996 |
| Alcohol intake frequency | rs4291980 | 18 | 50617175 | G | T | 0.433 | 0.017 | 0.004 | 3.13357e-06 | 336965 | 21.734 |
| Alcohol intake frequency | rs4417025 | 1 | 35363679 | A | G | 0.363 | -0.019 | 0.004 | 2.95189e-07 | 336965 | 26.282 |
| Alcohol intake frequency | rs4500930 | 2 | 228985505 | T | C | 0.342 | 0.02 | 0.004 | 1.6038e-07 | 336965 | 27.461 |
| Alcohol intake frequency | rs4726481 | 7 | 141668403 | T | G | 0.399 | 0.022 | 0.004 | 7.88969e-10 | 336965 | 37.789 |
| Alcohol intake frequency | rs4739105 | 8 | 64496159 | C | T | 0.786 | -0.022 | 0.004 | 4.08733e-07 | 336965 | 25.653 |
| Alcohol intake frequency | rs4739279 | 8 | 21000968 | C | A | 0.304 | 0.019 | 0.004 | 4.99563e-07 | 336965 | 25.266 |
| Alcohol intake frequency | rs4800487 | 18 | 21083275 | G | A | 0.461 | -0.03 | 0.004 | 4.01236e-17 | 336965 | 70.778 |
| Alcohol intake frequency | rs4801850 | 19 | 51210493 | G | A | 0.706 | 0.019 | 0.004 | 2.66944e-06 | 336965 | 22.041 |
| Alcohol intake frequency | rs4815364 | 20 | 25035711 | A | G | 0.606 | -0.017 | 0.004 | 1.73488e-06 | 336965 | 22.869 |
| Alcohol intake frequency | rs4887069 | 15 | 78909070 | G | A | 0.233 | -0.021 | 0.004 | 7.8986e-07 | 336965 | 24.383 |
| Alcohol intake frequency | rs489062 | 10 | 99715744 | A | G | 0.44 | 0.019 | 0.004 | 1.81958e-07 | 336965 | 27.217 |
| Alcohol intake frequency | rs4912532 | 3 | 184000619 | T | G | 0.491 | -0.017 | 0.004 | 9.78363e-07 | 336965 | 23.971 |
| Alcohol intake frequency | rs4970394 | 1 | 962891 | T | C | 0.579 | 0.02 | 0.004 | 5.39088e-08 | 336965 | 29.572 |
| Alcohol intake frequency | rs4982052 | 14 | 33588166 | G | A | 0.34 | -0.018 | 0.004 | 1.11946e-06 | 336965 | 23.712 |
| Alcohol intake frequency | rs533143 | 15 | 44188854 | C | T | 0.286 | -0.019 | 0.004 | 1.7574e-06 | 336965 | 22.844 |
| Alcohol intake frequency | rs540606 | 2 | 45138507 | G | A | 0.552 | -0.023 | 0.004 | 2.14274e-10 | 336965 | 40.334 |
| Alcohol intake frequency | rs560650 | 18 | 24404869 | G | T | 0.767 | -0.02 | 0.004 | 1.60517e-06 | 336965 | 23.018 |
| Alcohol intake frequency | rs56189237 | 6 | 31328518 | A | G | 0.129 | 0.028 | 0.005 | 1.28591e-07 | 336965 | 27.888 |
| Alcohol intake frequency | rs56228311 | 6 | 76179197 | T | G | 0.345 | -0.02 | 0.004 | 1.39053e-07 | 336965 | 27.737 |
| Alcohol intake frequency | rs56242580 | 14 | 56848182 | A | C | 0.247 | -0.021 | 0.004 | 2.27877e-07 | 336965 | 26.782 |
| Alcohol intake frequency | rs56247556 | 3 | 117743147 | C | T | 0.292 | 0.019 | 0.004 | 2.28528e-06 | 336965 | 22.34 |
| Alcohol intake frequency | rs571312 | 18 | 57839769 | A | C | 0.234 | 0.027 | 0.004 | 1.78443e-10 | 336965 | 40.692 |
| Alcohol intake frequency | rs5750673 | 22 | 39110124 | A | G | 0.278 | 0.019 | 0.004 | 1.35563e-06 | 336965 | 23.343 |
| Alcohol intake frequency | rs5758969 | 22 | 43228969 | C | A | 0.492 | 0.017 | 0.004 | 9.482e-07 | 336965 | 24.031 |
| Alcohol intake frequency | rs58740162 | 2 | 86620295 | A | G | 0.148 | -0.024 | 0.005 | 1.25603e-06 | 336965 | 23.49 |
| Alcohol intake frequency | rs58905411 | 12 | 54623132 | A | G | 0.412 | -0.03 | 0.004 | 9.31751e-17 | 336965 | 69.116 |
| Alcohol intake frequency | rs6016781 | 20 | 35555921 | T | C | 0.392 | -0.022 | 0.004 | 1.76795e-09 | 336965 | 36.216 |
| Alcohol intake frequency | rs60391689 | 17 | 27732587 | G | A | 0.181 | 0.024 | 0.005 | 1.73972e-07 | 336965 | 27.304 |
| Alcohol intake frequency | rs6078366 | 20 | 11848828 | T | G | 0.406 | 0.018 | 0.004 | 9.03899e-07 | 336965 | 24.123 |
| Alcohol intake frequency | rs61873510 | 10 | 102626510 | T | G | 0.329 | 0.023 | 0.004 | 2.0147e-09 | 336965 | 35.961 |
| Alcohol intake frequency | rs62159866 | 2 | 119607092 | G | A | 0.252 | 0.019 | 0.004 | 2.70097e-06 | 336965 | 22.019 |
| Alcohol intake frequency | rs62290364 | 3 | 132621814 | A | G | 0.145 | -0.025 | 0.005 | 5.00622e-07 | 336965 | 25.262 |
| Alcohol intake frequency | rs62466318 | 7 | 73042085 | T | C | 0.204 | -0.024 | 0.004 | 3.3759e-08 | 336965 | 30.48 |
| Alcohol intake frequency | rs6452788 | 5 | 87712913 | A | G | 0.236 | -0.031 | 0.004 | 1.69161e-13 | 336965 | 54.338 |
| Alcohol intake frequency | rs650558 | 17 | 40721042 | T | C | 0.25 | 0.023 | 0.004 | 1.04419e-08 | 336965 | 32.759 |
| Alcohol intake frequency | rs66523860 | 2 | 23880823 | G | A | 0.211 | 0.029 | 0.004 | 3.79228e-11 | 336965 | 43.721 |
| Alcohol intake frequency | rs6750325 | 2 | 211501333 | T | G | 0.446 | -0.017 | 0.004 | 2.89714e-06 | 336965 | 21.884 |
| Alcohol intake frequency | rs6776114 | 3 | 85426840 | A | G | 0.717 | 0.024 | 0.004 | 2.14714e-09 | 336965 | 35.837 |
| Alcohol intake frequency | rs6779539 | 3 | 28931568 | A | G | 0.377 | 0.017 | 0.004 | 3.41743e-06 | 336965 | 21.567 |
| Alcohol intake frequency | rs68024891 | 5 | 52748426 | C | T | 0.232 | -0.02 | 0.004 | 1.46974e-06 | 336965 | 23.188 |
| Alcohol intake frequency | rs7000542 | 8 | 9881135 | C | T | 0.284 | -0.018 | 0.004 | 3.54022e-06 | 336965 | 21.5 |
| Alcohol intake frequency | rs7143137 | 14 | 79952615 | C | T | 0.093 | -0.03 | 0.006 | 1.47096e-06 | 336965 | 23.186 |
| Alcohol intake frequency | rs7192193 | 16 | 26171345 | G | A | 0.562 | 0.017 | 0.004 | 1.38328e-06 | 336965 | 23.304 |
| Alcohol intake frequency | rs728538 | 16 | 51205819 | G | T | 0.167 | 0.023 | 0.005 | 1.10123e-06 | 336965 | 23.743 |
| Alcohol intake frequency | rs72946169 | 2 | 178151871 | C | A | 0.208 | 0.02 | 0.004 | 3.18559e-06 | 336965 | 21.702 |
| Alcohol intake frequency | rs7302200 | 12 | 56449435 | A | G | 0.343 | -0.018 | 0.004 | 1.7764e-06 | 336965 | 22.824 |
| Alcohol intake frequency | rs73050128 | 7 | 1961882 | A | C | 0.162 | -0.025 | 0.005 | 1.56153e-07 | 336965 | 27.513 |
| Alcohol intake frequency | rs7306710 | 12 | 66376091 | C | T | 0.518 | 0.018 | 0.004 | 3.49623e-07 | 336965 | 25.955 |
| Alcohol intake frequency | rs7323523 | 13 | 92912119 | C | T | 0.256 | -0.019 | 0.004 | 2.47902e-06 | 336965 | 22.183 |
| Alcohol intake frequency | rs73543300 | 19 | 5236519 | C | T | 0.085 | -0.032 | 0.006 | 6.29187e-07 | 336965 | 24.821 |
| Alcohol intake frequency | rs7428430 | 3 | 50174184 | T | C | 0.486 | -0.023 | 0.004 | 7.93963e-11 | 336965 | 42.275 |
| Alcohol intake frequency | rs74424378 | 9 | 109331094 | G | T | 0.236 | 0.02 | 0.004 | 1.32181e-06 | 336965 | 23.392 |
| Alcohol intake frequency | rs7460106 | 8 | 143534777 | C | T | 0.233 | -0.025 | 0.004 | 3.86919e-09 | 336965 | 34.69 |
| Alcohol intake frequency | rs74615678 | 10 | 22324266 | T | C | 0.013 | 0.077 | 0.016 | 1.42712e-06 | 336965 | 23.244 |
| Alcohol intake frequency | rs74688968 | 6 | 103000830 | G | A | 0.086 | -0.029 | 0.006 | 3.29686e-06 | 336965 | 21.636 |
| Alcohol intake frequency | rs74712803 | 19 | 39925056 | G | A | 0.153 | -0.023 | 0.005 | 1.8933e-06 | 336965 | 22.701 |
| Alcohol intake frequency | rs76225816 | 8 | 93038019 | G | A | 0.011 | 0.093 | 0.018 | 2.39398e-07 | 336965 | 26.687 |
| Alcohol intake frequency | rs76749787 | 7 | 12172960 | T | C | 0.121 | -0.026 | 0.005 | 1.74426e-06 | 336965 | 22.859 |
| Alcohol intake frequency | rs78910298 | 7 | 114109636 | C | A | 0.019 | 0.064 | 0.013 | 1.80551e-06 | 336965 | 22.792 |
| Alcohol intake frequency | rs7919624 | 10 | 2269933 | T | C | 0.383 | 0.019 | 0.004 | 4.06135e-07 | 336965 | 25.666 |
| Alcohol intake frequency | rs7948028 | 11 | 113328681 | C | A | 0.378 | 0.019 | 0.004 | 3.09642e-07 | 336965 | 26.189 |
| Alcohol intake frequency | rs80101850 | 9 | 82018969 | C | T | 0.039 | 0.045 | 0.009 | 1.3881e-06 | 336965 | 23.298 |
| Alcohol intake frequency | rs801733 | 11 | 65934549 | C | A | 0.359 | -0.02 | 0.004 | 8.77991e-08 | 336965 | 28.627 |
| Alcohol intake frequency | rs8030809 | 15 | 23886293 | A | G | 0.55 | 0.017 | 0.004 | 3.62418e-06 | 336965 | 21.455 |
| Alcohol intake frequency | rs838145 | 19 | 49248730 | A | G | 0.542 | 0.024 | 0.004 | 2.38726e-11 | 336965 | 44.627 |
| Alcohol intake frequency | rs874296 | 19 | 34107641 | C | T | 0.385 | 0.019 | 0.004 | 1.4771e-07 | 336965 | 27.62 |
| Alcohol intake frequency | rs9349379 | 6 | 12903957 | G | A | 0.405 | -0.019 | 0.004 | 2.19286e-07 | 336965 | 26.856 |
| Alcohol intake frequency | rs9372625 | 6 | 98344031 | A | G | 0.382 | -0.028 | 0.004 | 4.32514e-14 | 336965 | 57.019 |
| Alcohol intake frequency | rs9389888 | 6 | 141532281 | G | A | 0.366 | 0.017 | 0.004 | 2.96633e-06 | 336965 | 21.839 |
| Alcohol intake frequency | rs9690021 | 7 | 15444537 | T | G | 0.49 | 0.018 | 0.004 | 7.06529e-07 | 336965 | 24.598 |
| Alcohol intake frequency | rs9829192 | 3 | 38569463 | T | G | 0.433 | 0.019 | 0.004 | 1.35204e-07 | 336965 | 27.791 |
| Alcohol intake frequency | rs9842406 | 3 | 71553132 | G | T | 0.433 | -0.025 | 0.004 | 3.35815e-12 | 336965 | 48.471 |
| Alcohol intake frequency | rs9907011 | 17 | 7615286 | T | C | 0.164 | 0.026 | 0.005 | 8.53041e-08 | 336965 | 28.683 |
| Alcohol intake frequency | rs9923768 | 16 | 6163838 | A | G | 0.6 | -0.021 | 0.004 | 7.1425e-09 | 336965 | 33.497 |
| Average weekly beer plus cider intake | rs10446741 | 4 | 136025576 | A | C | 0.387 | -0.009 | 0.002 | 8.19993e-07 | 327634 | 24.309 |
| Average weekly beer plus cider intake | rs1057868 | 7 | 75615006 | T | C | 0.285 | -0.01 | 0.002 | 2.99999e-07 | 327634 | 26.243 |
| Average weekly beer plus cider intake | rs10812752 | 9 | 28186243 | G | A | 0.489 | -0.008 | 0.002 | 3.40001e-06 | 327634 | 21.578 |
| Average weekly beer plus cider intake | rs10822159 | 10 | 65096250 | T | C | 0.418 | -0.012 | 0.002 | 6.4003e-12 | 327634 | 47.194 |
| Average weekly beer plus cider intake | rs10894670 | 11 | 133221987 | A | C | 0.551 | -0.009 | 0.002 | 1.5e-07 | 327634 | 27.608 |
| Average weekly beer plus cider intake | rs10915840 | 1 | 225668524 | A | G | 0.273 | -0.01 | 0.002 | 5.30005e-07 | 327634 | 25.142 |
| Average weekly beer plus cider intake | rs10985396 | 9 | 124570555 | G | A | 0.579 | -0.009 | 0.002 | 2.59998e-07 | 327634 | 26.556 |
| Average weekly beer plus cider intake | rs113405475 | 10 | 120382042 | T | C | 0.283 | 0.009 | 0.002 | 2.30001e-06 | 327634 | 22.329 |
| Average weekly beer plus cider intake | rs113589940 | 15 | 87597742 | T | C | 0.007 | -0.049 | 0.01 | 2.5e-06 | 327634 | 22.203 |
| Average weekly beer plus cider intake | rs114185195 | 4 | 2371942 | T | C | 0.012 | -0.041 | 0.008 | 2.69998e-07 | 327634 | 26.463 |
| Average weekly beer plus cider intake | rs11615256 | 12 | 42843183 | T | C | 0.048 | 0.02 | 0.004 | 6.1e-07 | 327634 | 24.878 |
| Average weekly beer plus cider intake | rs11704314 | 22 | 40797933 | G | A | 0.19 | 0.01 | 0.002 | 2.5e-06 | 327634 | 22.18 |
| Average weekly beer plus cider intake | rs11987695 | 8 | 17781085 | C | T | 0.764 | 0.01 | 0.002 | 5.30005e-07 | 327634 | 25.158 |
| Average weekly beer plus cider intake | rs12046000 | 1 | 91192396 | T | G | 0.447 | -0.014 | 0.002 | 2.49977e-15 | 327634 | 62.613 |
| Average weekly beer plus cider intake | rs12065191 | 1 | 6803747 | C | T | 0.269 | -0.01 | 0.002 | 6.49995e-07 | 327634 | 24.747 |
| Average weekly beer plus cider intake | rs12135360 | 1 | 72228302 | T | G | 0.563 | -0.01 | 0.002 | 1.5e-08 | 327634 | 31.994 |
| Average weekly beer plus cider intake | rs1229984 | 4 | 100239319 | C | T | 0.977 | 0.048 | 0.006 | 6.00067e-18 | 327634 | 74.536 |
| Average weekly beer plus cider intake | rs12425997 | 12 | 195335 | C | T | 0.555 | 0.008 | 0.002 | 1.89998e-06 | 327634 | 22.653 |
| Average weekly beer plus cider intake | rs12513581 | 5 | 60533207 | C | T | 0.48 | -0.011 | 0.002 | 1.29999e-10 | 327634 | 41.26 |
| Average weekly beer plus cider intake | rs12596385 | 16 | 64720481 | T | C | 0.491 | 0.008 | 0.002 | 1.79999e-06 | 327634 | 22.796 |
| Average weekly beer plus cider intake | rs1283208 | 13 | 101316378 | G | A | 0.329 | 0.009 | 0.002 | 2.19999e-06 | 327634 | 22.376 |
| Average weekly beer plus cider intake | rs13130794 | 4 | 39422242 | C | T | 0.368 | -0.012 | 0.002 | 1.69981e-11 | 327634 | 45.237 |
| Average weekly beer plus cider intake | rs1317930 | 6 | 62386554 | G | A | 0.731 | 0.009 | 0.002 | 4.20001e-06 | 327634 | 21.158 |
| Average weekly beer plus cider intake | rs13184589 | 5 | 50471863 | T | G | 0.658 | 0.009 | 0.002 | 8.30004e-07 | 327634 | 24.294 |
| Average weekly beer plus cider intake | rs1387695 | 3 | 159191889 | G | T | 0.575 | 0.01 | 0.002 | 9.20005e-09 | 327634 | 33.011 |
| Average weekly beer plus cider intake | rs139171160 | 12 | 101273134 | G | A | 0.023 | -0.027 | 0.006 | 4.60002e-06 | 327634 | 21.006 |
| Average weekly beer plus cider intake | rs140525222 | 3 | 6503261 | T | G | 0.016 | 0.033 | 0.007 | 3.40001e-06 | 327634 | 21.565 |
| Average weekly beer plus cider intake | rs141265162 | 3 | 84411141 | A | G | 0.084 | -0.015 | 0.003 | 1.7e-06 | 327634 | 22.927 |
| Average weekly beer plus cider intake | rs141983215 | 8 | 18700020 | A | G | 0.099 | 0.014 | 0.003 | 1.89998e-06 | 327634 | 22.683 |
| Average weekly beer plus cider intake | rs1421085 | 16 | 53800954 | C | T | 0.402 | -0.015 | 0.002 | 8.30042e-19 | 327634 | 78.436 |
| Average weekly beer plus cider intake | rs149738491 | 1 | 34886282 | A | C | 0.014 | 0.038 | 0.007 | 4e-07 | 327634 | 25.684 |
| Average weekly beer plus cider intake | rs1514245 | 17 | 68335330 | T | C | 0.249 | 0.009 | 0.002 | 4.79999e-06 | 327634 | 20.913 |
| Average weekly beer plus cider intake | rs1520929 | 8 | 64700949 | C | T | 0.449 | 0.012 | 0.002 | 3.80014e-12 | 327634 | 48.227 |
| Average weekly beer plus cider intake | rs1531772 | 3 | 100367491 | T | C | 0.59 | 0.008 | 0.002 | 2.69998e-06 | 327634 | 22.053 |
| Average weekly beer plus cider intake | rs17478208 | 2 | 204450734 | A | G | 0.082 | -0.015 | 0.003 | 1.40001e-06 | 327634 | 23.33 |
| Average weekly beer plus cider intake | rs1940709 | 11 | 112849719 | A | G | 0.252 | 0.009 | 0.002 | 2.90001e-06 | 327634 | 21.867 |
| Average weekly beer plus cider intake | rs1986421 | 2 | 77766271 | A | G | 0.264 | -0.01 | 0.002 | 3.59998e-07 | 327634 | 25.894 |
| Average weekly beer plus cider intake | rs2072467 | 22 | 17288988 | C | T | 0.056 | -0.018 | 0.004 | 1.7e-06 | 327634 | 22.953 |
| Average weekly beer plus cider intake | rs2132747 | 12 | 40348056 | C | A | 0.267 | 0.01 | 0.002 | 5.80003e-07 | 327634 | 24.985 |
| Average weekly beer plus cider intake | rs2472297 | 15 | 75027880 | T | C | 0.263 | -0.01 | 0.002 | 2.19999e-07 | 327634 | 26.89 |
| Average weekly beer plus cider intake | rs2533273 | 7 | 153485282 | A | C | 0.486 | 0.011 | 0.002 | 1e-10 | 327634 | 41.757 |
| Average weekly beer plus cider intake | rs28478711 | 5 | 166842891 | G | A | 0.475 | -0.01 | 0.002 | 3.40001e-08 | 327634 | 30.492 |
| Average weekly beer plus cider intake | rs2857960 | 4 | 3740612 | A | C | 0.582 | 0.008 | 0.002 | 1.89998e-06 | 327634 | 22.683 |
| Average weekly beer plus cider intake | rs2883059 | 3 | 49902160 | C | T | 0.425 | 0.012 | 0.002 | 1.29987e-11 | 327634 | 45.839 |
| Average weekly beer plus cider intake | rs28929474 | 14 | 94844947 | T | C | 0.019 | -0.029 | 0.006 | 2e-06 | 327634 | 22.631 |
| Average weekly beer plus cider intake | rs34178057 | 4 | 100268602 | C | A | 0.099 | 0.017 | 0.003 | 7.69999e-09 | 327634 | 33.358 |
| Average weekly beer plus cider intake | rs34895146 | 12 | 65203978 | T | C | 0.037 | -0.022 | 0.005 | 1.6e-06 | 327634 | 23.083 |
| Average weekly beer plus cider intake | rs35528430 | 10 | 132982875 | A | G | 0.109 | 0.013 | 0.003 | 4.70002e-06 | 327634 | 20.94 |
| Average weekly beer plus cider intake | rs35782576 | 16 | 60714692 | C | A | 0.197 | -0.011 | 0.002 | 6.80002e-07 | 327634 | 24.662 |
| Average weekly beer plus cider intake | rs373687 | 21 | 46558780 | C | T | 0.36 | 0.009 | 0.002 | 4.70002e-07 | 327634 | 25.364 |
| Average weekly beer plus cider intake | rs4284036 | 8 | 78317705 | A | G | 0.391 | 0.009 | 0.002 | 6.4e-07 | 327634 | 24.787 |
| Average weekly beer plus cider intake | rs4401286 | 21 | 19681203 | C | T | 0.45 | -0.008 | 0.002 | 4.90004e-06 | 327634 | 20.885 |
| Average weekly beer plus cider intake | rs4516525 | 21 | 22969909 | A | G | 0.12 | 0.012 | 0.003 | 3.59998e-06 | 327634 | 21.467 |
| Average weekly beer plus cider intake | rs4648561 | 1 | 2465671 | C | T | 0.754 | -0.01 | 0.002 | 1.79999e-07 | 327634 | 27.235 |
| Average weekly beer plus cider intake | rs4675250 | 2 | 202914491 | G | A | 0.384 | -0.008 | 0.002 | 4.90004e-06 | 327634 | 20.894 |
| Average weekly beer plus cider intake | rs4688917 | 3 | 18174160 | G | A | 0.556 | 0.008 | 0.002 | 9.29994e-07 | 327634 | 24.065 |
| Average weekly beer plus cider intake | rs476849 | 8 | 130942962 | A | G | 0.461 | -0.009 | 0.002 | 6.29999e-07 | 327634 | 24.804 |
| Average weekly beer plus cider intake | rs4797776 | 18 | 13465234 | G | T | 0.183 | -0.01 | 0.002 | 2.59998e-06 | 327634 | 22.061 |
| Average weekly beer plus cider intake | rs4812281 | 20 | 59794157 | T | C | 0.742 | -0.009 | 0.002 | 2.69998e-06 | 327634 | 22.053 |
| Average weekly beer plus cider intake | rs4869870 | 6 | 156139323 | G | A | 0.128 | 0.013 | 0.003 | 8.99995e-07 | 327634 | 24.124 |
| Average weekly beer plus cider intake | rs493024 | 6 | 20034781 | T | C | 0.376 | 0.009 | 0.002 | 1.5e-06 | 327634 | 23.201 |
| Average weekly beer plus cider intake | rs4953150 | 2 | 45157336 | T | C | 0.346 | 0.011 | 0.002 | 5.39995e-09 | 327634 | 34.053 |
| Average weekly beer plus cider intake | rs4984738 | 16 | 1020343 | G | A | 0.332 | 0.008 | 0.002 | 4e-06 | 327634 | 21.272 |
| Average weekly beer plus cider intake | rs55747330 | 18 | 49957317 | T | C | 0.133 | 0.013 | 0.003 | 5.1e-07 | 327634 | 25.222 |
| Average weekly beer plus cider intake | rs56355243 | 17 | 79375687 | T | C | 0.36 | -0.009 | 0.002 | 6.4e-07 | 327634 | 24.787 |
| Average weekly beer plus cider intake | rs6020572 | 20 | 49128240 | A | G | 0.47 | -0.009 | 0.002 | 4.79999e-07 | 327634 | 25.335 |
| Average weekly beer plus cider intake | rs6044117 | 20 | 16541520 | A | G | 0.536 | 0.008 | 0.002 | 1.5e-06 | 327634 | 23.212 |
| Average weekly beer plus cider intake | rs60794128 | 1 | 111941590 | A | G | 0.027 | -0.026 | 0.005 | 6.4e-07 | 327634 | 24.797 |
| Average weekly beer plus cider intake | rs616157 | 12 | 120925795 | C | T | 0.17 | -0.011 | 0.002 | 1.89998e-06 | 327634 | 22.66 |
| Average weekly beer plus cider intake | rs61950313 | 13 | 21699729 | G | A | 0.14 | -0.012 | 0.002 | 1.29999e-06 | 327634 | 23.49 |
| Average weekly beer plus cider intake | rs62133869 | 2 | 32927360 | A | G | 0.019 | -0.03 | 0.006 | 2.19999e-06 | 327634 | 22.4 |
| Average weekly beer plus cider intake | rs6534338 | 4 | 123026869 | C | T | 0.703 | 0.009 | 0.002 | 1.89998e-06 | 327634 | 22.646 |
| Average weekly beer plus cider intake | rs6894360 | 5 | 114511505 | T | C | 0.335 | 0.01 | 0.002 | 9.20005e-08 | 327634 | 28.542 |
| Average weekly beer plus cider intake | rs7108260 | 11 | 95824761 | C | T | 0.558 | -0.009 | 0.002 | 3.59998e-07 | 327634 | 25.901 |
| Average weekly beer plus cider intake | rs722826 | 4 | 174098060 | A | G | 0.179 | 0.01 | 0.002 | 2.59998e-06 | 327634 | 22.075 |
| Average weekly beer plus cider intake | rs72681845 | 14 | 50624690 | T | G | 0.05 | -0.02 | 0.004 | 3.40001e-07 | 327634 | 25.982 |
| Average weekly beer plus cider intake | rs72792185 | 10 | 27033814 | T | C | 0.068 | -0.016 | 0.003 | 4.30002e-06 | 327634 | 21.143 |
| Average weekly beer plus cider intake | rs72822618 | 5 | 167747707 | T | G | 0.095 | -0.013 | 0.003 | 4.49997e-06 | 327634 | 21.03 |
| Average weekly beer plus cider intake | rs7299609 | 12 | 50024926 | T | C | 0.125 | -0.012 | 0.003 | 3.40001e-06 | 327634 | 21.556 |
| Average weekly beer plus cider intake | rs7334960 | 13 | 45893034 | G | A | 0.417 | 0.008 | 0.002 | 3.59998e-06 | 327634 | 21.486 |
| Average weekly beer plus cider intake | rs75413320 | 19 | 13149854 | C | T | 0.108 | 0.017 | 0.003 | 2e-09 | 327634 | 35.954 |
| Average weekly beer plus cider intake | rs76472635 | 6 | 98979585 | T | C | 0.017 | -0.033 | 0.007 | 2.69998e-06 | 327634 | 22.028 |
| Average weekly beer plus cider intake | rs7683596 | 4 | 113344431 | G | T | 0.439 | 0.008 | 0.002 | 1.6e-06 | 327634 | 23.024 |
| Average weekly beer plus cider intake | rs77373770 | 1 | 162589522 | A | G | 0.07 | 0.016 | 0.003 | 4e-06 | 327634 | 21.279 |
| Average weekly beer plus cider intake | rs7851830 | 9 | 109380084 | G | A | 0.234 | -0.013 | 0.002 | 4.49987e-11 | 327634 | 43.404 |
| Average weekly beer plus cider intake | rs79084783 | 2 | 81421464 | A | G | 0.027 | 0.026 | 0.005 | 1.2e-06 | 327634 | 23.573 |
| Average weekly beer plus cider intake | rs7916324 | 10 | 57190359 | A | G | 0.309 | 0.009 | 0.002 | 2.69998e-06 | 327634 | 22.021 |
| Average weekly beer plus cider intake | rs8014408 | 14 | 77681073 | C | A | 0.321 | 0.009 | 0.002 | 7.69999e-07 | 327634 | 24.429 |
| Average weekly beer plus cider intake | rs8044722 | 16 | 23878119 | T | G | 0.379 | -0.01 | 0.002 | 1.29999e-08 | 327634 | 32.36 |
| Average weekly beer plus cider intake | rs9570964 | 13 | 64216909 | C | T | 0.199 | -0.011 | 0.002 | 7.00003e-07 | 327634 | 24.607 |
| Average weekly beer plus cider intake | rs9777444 | 9 | 92518013 | G | A | 0.795 | 0.01 | 0.002 | 3.79997e-06 | 327634 | 21.362 |
| Average weekly beer plus cider intake | rs9824301 | 3 | 85682888 | C | A | 0.351 | -0.01 | 0.002 | 1e-08 | 327634 | 32.85 |
| Average weekly red wine intake | rs10006551 | 4 | 105433088 | T | C | 0.539 | 0.011 | 0.002 | 3.2e-07 | 327026 | 26.1 |
| Average weekly red wine intake | rs10156047 | 7 | 120109415 | T | G | 0.138 | -0.014 | 0.003 | 3.2e-06 | 327026 | 21.664 |
| Average weekly red wine intake | rs10210652 | 2 | 148469593 | A | G | 0.35 | -0.01 | 0.002 | 2.1e-06 | 327026 | 22.478 |
| Average weekly red wine intake | rs10280836 | 7 | 88730674 | G | T | 0.771 | -0.012 | 0.002 | 5.39995e-07 | 327026 | 25.112 |
| Average weekly red wine intake | rs10822129 | 10 | 64846841 | T | C | 0.407 | 0.013 | 0.002 | 2.69998e-10 | 327026 | 39.874 |
| Average weekly red wine intake | rs10865093 | 2 | 22459372 | C | T | 0.448 | -0.01 | 0.002 | 3.89996e-06 | 327026 | 21.293 |
| Average weekly red wine intake | rs10873070 | 14 | 23460556 | G | A | 0.624 | -0.01 | 0.002 | 3.50002e-06 | 327026 | 21.538 |
| Average weekly red wine intake | rs10925183 | 1 | 236812414 | A | G | 0.607 | -0.012 | 0.002 | 1.6e-08 | 327026 | 31.874 |
| Average weekly red wine intake | rs11021354 | 11 | 95672960 | G | A | 0.393 | 0.011 | 0.002 | 1.2e-07 | 327026 | 28.021 |
| Average weekly red wine intake | rs11024405 | 11 | 17820848 | G | A | 0.185 | -0.014 | 0.003 | 2.80001e-07 | 327026 | 26.414 |
| Average weekly red wine intake | rs11114498 | 12 | 80922026 | T | C | 0.674 | -0.01 | 0.002 | 3.50002e-06 | 327026 | 21.507 |
| Average weekly red wine intake | rs11126576 | 2 | 77162367 | T | C | 0.44 | 0.011 | 0.002 | 1.5e-07 | 327026 | 27.625 |
| Average weekly red wine intake | rs11221890 | 11 | 99786011 | G | A | 0.148 | 0.016 | 0.003 | 5.49997e-08 | 327026 | 29.54 |
| Average weekly red wine intake | rs114313565 | 1 | 211277546 | A | G | 0.027 | 0.032 | 0.007 | 9.59997e-07 | 327026 | 24.016 |
| Average weekly red wine intake | rs114795493 | 2 | 1932678 | A | G | 0.016 | 0.039 | 0.008 | 1.5e-06 | 327026 | 23.129 |
| Average weekly red wine intake | rs115189110 | 3 | 17680348 | T | C | 0.042 | 0.025 | 0.005 | 3.59998e-06 | 327026 | 21.473 |
| Average weekly red wine intake | rs11662234 | 18 | 50907289 | G | T | 0.524 | -0.009 | 0.002 | 4.79999e-06 | 327026 | 20.914 |
| Average weekly red wine intake | rs11705856 | 3 | 117324327 | A | G | 0.643 | 0.011 | 0.002 | 5.99998e-07 | 327026 | 24.923 |
| Average weekly red wine intake | rs11714337 | 3 | 71582521 | A | G | 0.433 | 0.012 | 0.002 | 2.90001e-09 | 327026 | 35.218 |
| Average weekly red wine intake | rs117331323 | 15 | 83564379 | T | C | 0.025 | 0.03 | 0.007 | 4.70002e-06 | 327026 | 20.967 |
| Average weekly red wine intake | rs11877758 | 18 | 35138110 | G | T | 0.311 | -0.011 | 0.002 | 1.5e-06 | 327026 | 23.127 |
| Average weekly red wine intake | rs1229984 | 4 | 100239319 | C | T | 0.977 | 0.061 | 0.007 | 1.50003e-19 | 327026 | 81.855 |
| Average weekly red wine intake | rs12373827 | 2 | 101043669 | A | G | 0.516 | 0.01 | 0.002 | 3.79997e-06 | 327026 | 21.346 |
| Average weekly red wine intake | rs12692596 | 2 | 161265910 | T | C | 0.371 | -0.012 | 0.002 | 4.49997e-08 | 327026 | 29.933 |
| Average weekly red wine intake | rs13034936 | 2 | 116592936 | C | T | 0.112 | -0.017 | 0.003 | 1.29999e-07 | 327026 | 27.822 |
| Average weekly red wine intake | rs1364603 | 14 | 80020811 | G | A | 0.625 | -0.011 | 0.002 | 7.59994e-07 | 327026 | 24.466 |
| Average weekly red wine intake | rs1441165 | 2 | 214009831 | G | A | 0.535 | -0.011 | 0.002 | 1.2e-07 | 327026 | 28.024 |
| Average weekly red wine intake | rs146821330 | 19 | 53270926 | C | T | 0.014 | -0.041 | 0.009 | 2.19999e-06 | 327026 | 22.405 |
| Average weekly red wine intake | rs164328 | 3 | 6800044 | T | G | 0.912 | 0.018 | 0.004 | 5.19996e-07 | 327026 | 25.191 |
| Average weekly red wine intake | rs16989140 | 4 | 33657126 | T | C | 0.089 | -0.019 | 0.004 | 1.79999e-07 | 327026 | 27.258 |
| Average weekly red wine intake | rs17817497 | 16 | 53815435 | C | T | 0.392 | 0.014 | 0.002 | 2.60016e-11 | 327026 | 44.426 |
| Average weekly red wine intake | rs1896297 | 2 | 60738227 | T | C | 0.656 | -0.012 | 0.002 | 5.60003e-08 | 327026 | 29.482 |
| Average weekly red wine intake | rs1916414 | 10 | 65670393 | G | A | 0.535 | 0.01 | 0.002 | 1.2e-06 | 327026 | 23.507 |
| Average weekly red wine intake | rs1966836 | 11 | 57982229 | G | A | 0.711 | 0.012 | 0.002 | 5.80003e-08 | 327026 | 29.425 |
| Average weekly red wine intake | rs211799 | 7 | 110076424 | G | A | 0.95 | -0.022 | 0.005 | 3.59998e-06 | 327026 | 21.443 |
| Average weekly red wine intake | rs2162277 | 1 | 93900247 | T | C | 0.875 | 0.015 | 0.003 | 2.39999e-06 | 327026 | 22.23 |
| Average weekly red wine intake | rs2291007 | 4 | 159828328 | C | T | 0.571 | 0.01 | 0.002 | 2.19999e-06 | 327026 | 22.375 |
| Average weekly red wine intake | rs2383361 | 4 | 180814355 | A | G | 0.576 | -0.01 | 0.002 | 2.1e-06 | 327026 | 22.536 |
| Average weekly red wine intake | rs2511225 | 11 | 66248431 | C | A | 0.354 | -0.012 | 0.002 | 6.19998e-08 | 327026 | 29.292 |
| Average weekly red wine intake | rs261046 | 5 | 169332729 | A | G | 0.245 | 0.011 | 0.002 | 4.20001e-06 | 327026 | 21.183 |
| Average weekly red wine intake | rs2789517 | 9 | 129835114 | A | G | 0.868 | 0.015 | 0.003 | 1.79999e-06 | 327026 | 22.755 |
| Average weekly red wine intake | rs28768122 | 12 | 123885974 | C | T | 0.758 | -0.013 | 0.002 | 1.89998e-07 | 327026 | 27.171 |
| Average weekly red wine intake | rs300918 | 4 | 144266979 | T | C | 0.547 | -0.01 | 0.002 | 2.80001e-06 | 327026 | 21.934 |
| Average weekly red wine intake | rs303753 | 18 | 21074922 | A | G | 0.345 | -0.018 | 0.002 | 6.00067e-17 | 327026 | 69.989 |
| Average weekly red wine intake | rs34198201 | 2 | 236663836 | A | C | 0.231 | 0.012 | 0.002 | 2.1e-06 | 327026 | 22.504 |
| Average weekly red wine intake | rs34863300 | 7 | 20826452 | G | T | 0.31 | -0.011 | 0.002 | 1.89998e-06 | 327026 | 22.733 |
| Average weekly red wine intake | rs35488630 | 7 | 29108030 | A | G | 0.178 | 0.013 | 0.003 | 1.2e-06 | 327026 | 23.656 |
| Average weekly red wine intake | rs35698271 | 1 | 96256387 | C | A | 0.18 | -0.015 | 0.003 | 1.29999e-08 | 327026 | 32.385 |
| Average weekly red wine intake | rs3783297 | 14 | 30064026 | C | T | 0.369 | -0.011 | 0.002 | 2.90001e-07 | 327026 | 26.327 |
| Average weekly red wine intake | rs3913960 | 5 | 25228227 | C | T | 0.626 | 0.01 | 0.002 | 4.30002e-06 | 327026 | 21.13 |
| Average weekly red wine intake | rs4757370 | 11 | 15885136 | A | G | 0.264 | -0.011 | 0.002 | 2.69998e-06 | 327026 | 22.016 |
| Average weekly red wine intake | rs4900965 | 14 | 50452055 | C | T | 0.307 | -0.01 | 0.002 | 4.79999e-06 | 327026 | 20.916 |
| Average weekly red wine intake | rs4953122 | 2 | 44861792 | A | G | 0.665 | -0.011 | 0.002 | 1.79999e-07 | 327026 | 27.186 |
| Average weekly red wine intake | rs55968191 | 8 | 142705706 | A | G | 0.247 | 0.013 | 0.002 | 4.70002e-08 | 327026 | 29.855 |
| Average weekly red wine intake | rs56006101 | 17 | 78888436 | T | C | 0.019 | 0.04 | 0.008 | 1.79999e-07 | 327026 | 27.259 |
| Average weekly red wine intake | rs568030 | 13 | 30654661 | T | G | 0.743 | 0.011 | 0.002 | 1.29999e-06 | 327026 | 23.394 |
| Average weekly red wine intake | rs60170726 | 2 | 185527505 | A | G | 0.143 | 0.014 | 0.003 | 2.39999e-06 | 327026 | 22.28 |
| Average weekly red wine intake | rs61958175 | 13 | 58680250 | G | A | 0.048 | 0.024 | 0.005 | 5.30005e-07 | 327026 | 25.144 |
| Average weekly red wine intake | rs62573521 | 9 | 22648200 | T | C | 0.04 | -0.032 | 0.005 | 7.49998e-10 | 327026 | 37.888 |
| Average weekly red wine intake | rs627685 | 18 | 53186092 | C | T | 0.303 | -0.013 | 0.002 | 2.99999e-08 | 327026 | 30.738 |
| Average weekly red wine intake | rs6442994 | 3 | 5849677 | T | C | 0.775 | -0.012 | 0.002 | 1e-06 | 327026 | 23.874 |
| Average weekly red wine intake | rs6552079 | 4 | 68026392 | T | G | 0.558 | 0.011 | 0.002 | 5.89997e-08 | 327026 | 29.397 |
| Average weekly red wine intake | rs6882046 | 5 | 87968864 | G | A | 0.269 | 0.016 | 0.002 | 4.10015e-12 | 327026 | 48.072 |
| Average weekly red wine intake | rs6978944 | 7 | 108619 | G | A | 0.203 | -0.012 | 0.003 | 2.1e-06 | 327026 | 22.512 |
| Average weekly red wine intake | rs7277942 | 21 | 39880854 | T | C | 0.173 | 0.013 | 0.003 | 3.79997e-06 | 327026 | 21.361 |
| Average weekly red wine intake | rs73373942 | 6 | 867824 | T | C | 0.148 | 0.014 | 0.003 | 1.7e-06 | 327026 | 22.919 |
| Average weekly red wine intake | rs7431028 | 3 | 36110865 | G | T | 0.526 | -0.01 | 0.002 | 1.6e-06 | 327026 | 23.002 |
| Average weekly red wine intake | rs7433378 | 3 | 82502402 | T | C | 0.232 | 0.012 | 0.002 | 2.5e-06 | 327026 | 22.163 |
| Average weekly red wine intake | rs74567946 | 4 | 7122623 | G | T | 0.029 | 0.031 | 0.006 | 5.89997e-07 | 327026 | 24.957 |
| Average weekly red wine intake | rs7460106 | 8 | 143534777 | C | T | 0.237 | 0.011 | 0.002 | 3.69999e-06 | 327026 | 21.395 |
| Average weekly red wine intake | rs77061979 | 15 | 57372714 | A | G | 0.03 | 0.028 | 0.006 | 4.60002e-06 | 327026 | 20.993 |
| Average weekly red wine intake | rs898751 | 17 | 2291863 | T | C | 0.492 | -0.012 | 0.002 | 2.1e-09 | 327026 | 35.869 |
| Average weekly red wine intake | rs927769 | 6 | 158430614 | A | C | 0.627 | 0.01 | 0.002 | 1.79999e-06 | 327026 | 22.851 |
| Average weekly red wine intake | rs9329343 | 10 | 14691614 | G | A | 0.451 | -0.01 | 0.002 | 4.49997e-06 | 327026 | 21.038 |
| Average weekly red wine intake | rs9585326 | 13 | 100688671 | G | A | 0.534 | 0.011 | 0.002 | 1.09999e-07 | 327026 | 28.232 |
| Average weekly red wine intake | rs9728289 | 1 | 28666536 | G | A | 0.411 | -0.01 | 0.002 | 2.19999e-06 | 327026 | 22.406 |
| Average weekly red wine intake | rs9899357 | 17 | 19800549 | C | T | 0.382 | -0.01 | 0.002 | 4.20001e-06 | 327026 | 21.19 |
| Coffee intake | rs10053913 | 5 | 164999502 | G | A | 0.356 | 0.008 | 0.002 | 8.30004e-07 | 428860 | 24.297 |
| Coffee intake | rs10193706 | 2 | 146316319 | C | A | 0.523 | 0.008 | 0.002 | 5.19996e-07 | 428860 | 25.189 |
| Coffee intake | rs10500356 | 16 | 7459614 | C | T | 0.442 | 0.008 | 0.002 | 1.40001e-06 | 428860 | 23.33 |
| Coffee intake | rs1057868 | 7 | 75615006 | T | C | 0.285 | 0.02 | 0.002 | 5.40008e-29 | 428860 | 124.9 |
| Coffee intake | rs10938398 | 4 | 45186139 | A | G | 0.434 | 0.008 | 0.002 | 4.09996e-06 | 428860 | 21.207 |
| Coffee intake | rs10992783 | 9 | 96311256 | T | C | 0.419 | -0.008 | 0.002 | 3.29997e-06 | 428860 | 21.612 |
| Coffee intake | rs11206378 | 1 | 40049184 | A | G | 0.644 | 0.008 | 0.002 | 2.5e-06 | 428860 | 22.2 |
| Coffee intake | rs11236225 | 11 | 74424075 | G | A | 0.202 | -0.01 | 0.002 | 5.89997e-07 | 428860 | 24.952 |
| Coffee intake | rs117514553 | 8 | 14842749 | G | A | 0.029 | 0.022 | 0.005 | 2.99999e-06 | 428860 | 21.838 |
| Coffee intake | rs117810762 | 10 | 135315795 | A | G | 0.018 | 0.036 | 0.006 | 6.19998e-09 | 428860 | 33.775 |
| Coffee intake | rs117968677 | 15 | 75174251 | A | G | 0.024 | -0.031 | 0.006 | 1.89998e-08 | 428860 | 31.645 |
| Coffee intake | rs1182580 | 1 | 104446782 | T | C | 0.474 | 0.008 | 0.002 | 9.80009e-07 | 428860 | 23.962 |
| Coffee intake | rs12514566 | 5 | 7391462 | A | G | 0.337 | -0.011 | 0.002 | 2.39994e-11 | 428860 | 44.651 |
| Coffee intake | rs12604773 | 18 | 19028164 | G | A | 0.663 | -0.009 | 0.002 | 4.30002e-07 | 428860 | 25.544 |
| Coffee intake | rs12649199 | 4 | 152639284 | A | G | 0.284 | -0.008 | 0.002 | 2.39999e-06 | 428860 | 22.233 |
| Coffee intake | rs12930957 | 16 | 83385118 | C | T | 0.642 | 0.008 | 0.002 | 9.40005e-07 | 428860 | 24.048 |
| Coffee intake | rs12989746 | 2 | 49368391 | T | G | 0.25 | 0.01 | 0.002 | 2.80001e-08 | 428860 | 30.821 |
| Coffee intake | rs13054099 | 22 | 41215672 | C | T | 0.261 | -0.011 | 0.002 | 4.30002e-09 | 428860 | 34.46 |
| Coffee intake | rs13096450 | 3 | 22172572 | G | A | 0.128 | -0.012 | 0.002 | 1.7e-06 | 428860 | 22.912 |
| Coffee intake | rs13163336 | 5 | 87943710 | A | C | 0.158 | 0.015 | 0.002 | 1.29987e-11 | 428860 | 45.742 |
| Coffee intake | rs13378244 | 13 | 40806355 | A | G | 0.419 | 0.008 | 0.002 | 1.5e-06 | 428860 | 23.172 |
| Coffee intake | rs1338549 | 6 | 98312143 | G | T | 0.534 | -0.009 | 0.002 | 5.60003e-09 | 428860 | 33.961 |
| Coffee intake | rs13387939 | 2 | 637498 | A | C | 0.828 | 0.017 | 0.002 | 9.79941e-15 | 428860 | 59.929 |
| Coffee intake | rs13437947 | 7 | 12098979 | A | G | 0.158 | 0.01 | 0.002 | 2.59998e-06 | 428860 | 22.097 |
| Coffee intake | rs1421085 | 16 | 53800954 | C | T | 0.404 | 0.019 | 0.002 | 1.69981e-29 | 428860 | 127.159 |
| Coffee intake | rs144620429 | 1 | 227527871 | T | C | 0.091 | 0.013 | 0.003 | 2.5e-06 | 428860 | 22.201 |
| Coffee intake | rs1447365 | 4 | 30861964 | G | A | 0.668 | 0.008 | 0.002 | 1.29999e-06 | 428860 | 23.459 |
| Coffee intake | rs1527961 | 2 | 62780440 | C | T | 0.135 | -0.013 | 0.002 | 1.7e-08 | 428860 | 31.808 |
| Coffee intake | rs16930598 | 8 | 64604836 | A | G | 0.043 | -0.019 | 0.004 | 2.99999e-06 | 428860 | 21.806 |
| Coffee intake | rs17144566 | 11 | 82798226 | T | C | 0.196 | -0.011 | 0.002 | 8.40001e-08 | 428860 | 28.711 |
| Coffee intake | rs17842490 | 22 | 24870527 | G | A | 0.014 | -0.045 | 0.007 | 3.29989e-11 | 428860 | 44.011 |
| Coffee intake | rs1942965 | 18 | 55032486 | C | T | 0.505 | -0.009 | 0.002 | 3.79997e-08 | 428860 | 30.236 |
| Coffee intake | rs2126069 | 11 | 115473374 | T | C | 0.485 | 0.009 | 0.002 | 5.49997e-08 | 428860 | 29.537 |
| Coffee intake | rs2189234 | 4 | 106075498 | G | T | 0.618 | 0.01 | 0.002 | 1.79999e-09 | 428860 | 36.172 |
| Coffee intake | rs2231142 | 4 | 89052323 | T | G | 0.113 | -0.013 | 0.003 | 7.90005e-07 | 428860 | 24.378 |
| Coffee intake | rs2350804 | 4 | 67075938 | T | C | 0.726 | -0.009 | 0.002 | 1.09999e-06 | 428860 | 23.778 |
| Coffee intake | rs2373834 | 7 | 88661253 | G | A | 0.805 | -0.009 | 0.002 | 4.49997e-06 | 428860 | 21.019 |
| Coffee intake | rs2400677 | 14 | 99957703 | C | T | 0.574 | -0.008 | 0.002 | 3.89996e-07 | 428860 | 25.722 |
| Coffee intake | rs2465037 | 6 | 51179260 | A | C | 0.343 | -0.011 | 0.002 | 4.79999e-10 | 428860 | 38.772 |
| Coffee intake | rs2472297 | 15 | 75027880 | T | C | 0.263 | 0.046 | 0.002 | 1.09901e-142 | 428860 | 646.732 |
| Coffee intake | rs2577374 | 10 | 117954101 | A | G | 0.587 | 0.009 | 0.002 | 1.6e-07 | 428860 | 27.509 |
| Coffee intake | rs2595223 | 2 | 129082091 | C | T | 0.595 | -0.008 | 0.002 | 7.19996e-07 | 428860 | 24.569 |
| Coffee intake | rs2597805 | 4 | 17424930 | T | C | 0.682 | 0.01 | 0.002 | 2e-08 | 428860 | 31.488 |
| Coffee intake | rs2842183 | 1 | 44020394 | C | T | 0.219 | -0.01 | 0.002 | 1e-06 | 428860 | 23.867 |
| Coffee intake | rs28490963 | 7 | 113463095 | A | G | 0.074 | 0.015 | 0.003 | 9.80009e-07 | 428860 | 23.974 |
| Coffee intake | rs329122 | 5 | 133864599 | A | G | 0.42 | -0.009 | 0.002 | 8.70001e-08 | 428860 | 28.651 |
| Coffee intake | rs34060476 | 7 | 73037956 | G | A | 0.134 | 0.018 | 0.002 | 7.50067e-15 | 428860 | 60.45 |
| Coffee intake | rs34276507 | 19 | 4300415 | A | G | 0.342 | 0.008 | 0.002 | 4.30002e-06 | 428860 | 21.12 |
| Coffee intake | rs34715167 | 3 | 29142706 | T | C | 0.376 | 0.008 | 0.002 | 7.49998e-07 | 428860 | 24.488 |
| Coffee intake | rs35303290 | 7 | 1669295 | T | C | 0.334 | -0.009 | 0.002 | 2.90001e-07 | 428860 | 26.313 |
| Coffee intake | rs4410790 | 7 | 17284577 | C | T | 0.632 | 0.039 | 0.002 | 1.1995e-120 | 428860 | 545.506 |
| Coffee intake | rs4615895 | 1 | 96274668 | A | G | 0.741 | 0.012 | 0.002 | 4.19952e-11 | 428860 | 43.519 |
| Coffee intake | rs476828 | 18 | 57852587 | C | T | 0.237 | 0.017 | 0.002 | 5.60015e-20 | 428860 | 83.75 |
| Coffee intake | rs4836963 | 9 | 126852356 | A | G | 0.098 | 0.013 | 0.003 | 2.1e-06 | 428860 | 22.526 |
| Coffee intake | rs4840338 | 8 | 8218967 | G | A | 0.522 | 0.008 | 0.002 | 3.29997e-06 | 428860 | 21.656 |
| Coffee intake | rs4938000 | 11 | 113060336 | C | T | 0.661 | -0.008 | 0.002 | 1e-06 | 428860 | 23.864 |
| Coffee intake | rs4970968 | 1 | 150612360 | T | G | 0.636 | -0.008 | 0.002 | 1.89998e-06 | 428860 | 22.653 |
| Coffee intake | rs4984636 | 16 | 1252441 | C | T | 0.321 | -0.008 | 0.002 | 2.30001e-06 | 428860 | 22.355 |
| Coffee intake | rs516636 | 1 | 177855517 | A | C | 0.209 | 0.012 | 0.002 | 4e-09 | 428860 | 34.632 |
| Coffee intake | rs55716899 | 3 | 71498561 | C | T | 0.429 | 0.008 | 0.002 | 1.29999e-06 | 428860 | 23.4 |
| Coffee intake | rs55780162 | 7 | 140169706 | G | A | 0.381 | 0.008 | 0.002 | 2.39999e-06 | 428860 | 22.239 |
| Coffee intake | rs55895231 | 8 | 93645556 | G | A | 0.116 | -0.012 | 0.003 | 3.09999e-06 | 428860 | 21.745 |
| Coffee intake | rs56113850 | 19 | 41353107 | C | T | 0.578 | 0.013 | 0.002 | 8.9002e-15 | 428860 | 60.129 |
| Coffee intake | rs56212739 | 9 | 23705541 | A | G | 0.195 | 0.01 | 0.002 | 1.2e-06 | 428860 | 23.505 |
| Coffee intake | rs56349356 | 6 | 139889377 | A | G | 0.021 | -0.027 | 0.006 | 4.60002e-06 | 428860 | 20.985 |
| Coffee intake | rs56791931 | 16 | 19977420 | T | G | 0.143 | -0.011 | 0.002 | 9.29994e-07 | 428860 | 24.074 |
| Coffee intake | rs57918684 | 17 | 60150383 | A | G | 0.155 | 0.013 | 0.002 | 8.60003e-09 | 428860 | 33.141 |
| Coffee intake | rs6030315 | 20 | 35569824 | A | C | 0.394 | -0.008 | 0.002 | 2.80001e-06 | 428860 | 21.977 |
| Coffee intake | rs6062682 | 20 | 62891820 | T | C | 0.465 | 0.01 | 0.002 | 2.5e-10 | 428860 | 40.02 |
| Coffee intake | rs6063085 | 20 | 45840459 | C | A | 0.373 | 0.01 | 0.002 | 4.49997e-10 | 428860 | 38.899 |
| Coffee intake | rs61928609 | 12 | 11316437 | C | A | 0.835 | -0.015 | 0.002 | 1.29987e-11 | 428860 | 45.853 |
| Coffee intake | rs62064918 | 17 | 46155786 | T | C | 0.245 | -0.01 | 0.002 | 4.09996e-08 | 428860 | 30.101 |
| Coffee intake | rs62121718 | 19 | 2552646 | G | A | 0.261 | -0.009 | 0.002 | 2.1e-06 | 428860 | 22.481 |
| Coffee intake | rs630194 | 18 | 40950954 | C | T | 0.343 | -0.011 | 0.002 | 2.29985e-11 | 428860 | 44.678 |
| Coffee intake | rs641727 | 11 | 56267616 | G | T | 0.312 | -0.009 | 0.002 | 7.29995e-08 | 428860 | 28.981 |
| Coffee intake | rs6469262 | 8 | 110443480 | C | T | 0.565 | -0.009 | 0.002 | 1.89998e-08 | 428860 | 31.576 |
| Coffee intake | rs6544966 | 2 | 23801620 | T | C | 0.498 | 0.008 | 0.002 | 1.40001e-06 | 428860 | 23.245 |
| Coffee intake | rs6691876 | 1 | 50524326 | A | C | 0.673 | -0.009 | 0.002 | 9.80009e-08 | 428860 | 28.419 |
| Coffee intake | rs6937318 | 6 | 32024832 | C | T | 0.509 | -0.008 | 0.002 | 1.29999e-07 | 428860 | 27.857 |
| Coffee intake | rs6969878 | 7 | 89649411 | G | A | 0.213 | -0.011 | 0.002 | 5.30005e-08 | 428860 | 29.608 |
| Coffee intake | rs6989976 | 8 | 76230087 | G | A | 0.612 | 0.008 | 0.002 | 1.5e-06 | 428860 | 23.169 |
| Coffee intake | rs71429826 | 13 | 23659244 | A | C | 0.222 | 0.009 | 0.002 | 3.29997e-06 | 428860 | 21.62 |
| Coffee intake | rs723145 | 9 | 8867519 | G | A | 0.798 | 0.01 | 0.002 | 1e-06 | 428860 | 23.878 |
| Coffee intake | rs730632 | 2 | 6566079 | T | C | 0.179 | 0.011 | 0.002 | 7.69999e-07 | 428860 | 24.42 |
| Coffee intake | rs73123665 | 3 | 83791546 | G | A | 0.058 | -0.016 | 0.003 | 2.30001e-06 | 428860 | 22.355 |
| Coffee intake | rs73228435 | 12 | 97413066 | A | G | 0.041 | -0.021 | 0.004 | 2e-07 | 428860 | 27.052 |
| Coffee intake | rs75347775 | 19 | 18495908 | A | G | 0.245 | 0.01 | 0.002 | 2.69998e-08 | 428860 | 30.933 |
| Coffee intake | rs7585210 | 2 | 141269025 | A | C | 0.704 | -0.008 | 0.002 | 4.49997e-06 | 428860 | 21.024 |
| Coffee intake | rs7590617 | 2 | 160631948 | G | A | 0.503 | 0.007 | 0.002 | 3.29997e-06 | 428860 | 21.606 |
| Coffee intake | rs76852278 | 8 | 121700140 | A | C | 0.023 | 0.027 | 0.006 | 7.90005e-07 | 428860 | 24.375 |
| Coffee intake | rs76881016 | 10 | 134196286 | G | A | 0.072 | 0.016 | 0.003 | 2.5e-07 | 428860 | 26.614 |
| Coffee intake | rs77350503 | 3 | 6835383 | C | T | 0.094 | 0.013 | 0.003 | 4.70002e-06 | 428860 | 20.969 |
| Coffee intake | rs7743687 | 6 | 164680854 | C | T | 0.335 | 0.008 | 0.002 | 8.30004e-07 | 428860 | 24.279 |
| Coffee intake | rs780093 | 2 | 27742603 | C | T | 0.616 | 0.013 | 0.002 | 1e-15 | 428860 | 64.366 |
| Coffee intake | rs7811609 | 7 | 32930597 | T | C | 0.375 | 0.009 | 0.002 | 4e-08 | 428860 | 30.137 |
| Coffee intake | rs78308709 | 1 | 56906358 | C | T | 0.064 | -0.017 | 0.003 | 3.50002e-07 | 428860 | 25.952 |
| Coffee intake | rs7989746 | 13 | 45882511 | C | A | 0.467 | -0.008 | 0.002 | 3.59998e-06 | 428860 | 21.482 |
| Coffee intake | rs8056750 | 16 | 70927078 | T | C | 0.359 | 0.011 | 0.002 | 1.29999e-09 | 428860 | 36.776 |
| Coffee intake | rs8068193 | 17 | 78930171 | A | G | 0.407 | 0.008 | 0.002 | 3.40001e-06 | 428860 | 21.574 |
| Coffee intake | rs9296406 | 6 | 42940810 | C | T | 0.541 | 0.008 | 0.002 | 1.7e-06 | 428860 | 22.944 |
| Coffee intake | rs9398171 | 6 | 108983527 | T | C | 0.711 | 0.011 | 0.002 | 1.09999e-09 | 428860 | 37.21 |
| Coffee intake | rs9650210 | 8 | 65496059 | A | C | 0.112 | 0.013 | 0.003 | 6.1e-07 | 428860 | 24.867 |
| Coffee intake | rs9953004 | 18 | 73002747 | T | C | 0.346 | 0.008 | 0.002 | 3.69999e-06 | 428860 | 21.412 |
| Tea intake | rs10082992 | 12 | 72826693 | A | C | 0.244 | -0.013 | 0.002 | 1.6e-07 | 447485 | 27.443 |
| Tea intake | rs1020111 | 2 | 201247780 | A | G | 0.539 | 0.011 | 0.002 | 6.59994e-07 | 447485 | 24.729 |
| Tea intake | rs10244642 | 7 | 140124826 | C | T | 0.111 | -0.016 | 0.003 | 2.59998e-06 | 447485 | 22.12 |
| Tea intake | rs10281069 | 7 | 75499311 | T | C | 0.483 | -0.01 | 0.002 | 3.50002e-06 | 447485 | 21.536 |
| Tea intake | rs10741694 | 11 | 16286183 | C | T | 0.628 | 0.015 | 0.002 | 7.89951e-12 | 447485 | 46.784 |
| Tea intake | rs10752269 | 10 | 12692902 | A | G | 0.506 | -0.013 | 0.002 | 1.29999e-09 | 447485 | 36.878 |
| Tea intake | rs10764990 | 10 | 129152608 | A | G | 0.607 | -0.012 | 0.002 | 1.89998e-08 | 447485 | 31.589 |
| Tea intake | rs10807652 | 7 | 157786074 | A | G | 0.837 | 0.014 | 0.003 | 2.69998e-06 | 447485 | 22.027 |
| Tea intake | rs10879354 | 12 | 72409782 | T | C | 0.61 | -0.011 | 0.002 | 5e-07 | 447485 | 25.257 |
| Tea intake | rs111285082 | 4 | 2142094 | A | G | 0.055 | -0.024 | 0.005 | 1.89998e-07 | 447485 | 27.179 |
| Tea intake | rs11131298 | 4 | 61982282 | A | G | 0.291 | 0.011 | 0.002 | 1.09999e-06 | 447485 | 23.736 |
| Tea intake | rs111628390 | 6 | 20297823 | T | C | 0.07 | 0.019 | 0.004 | 4.70002e-06 | 447485 | 20.964 |
| Tea intake | rs111747345 | 3 | 9541149 | T | C | 0.058 | 0.022 | 0.005 | 1.5e-06 | 447485 | 23.194 |
| Tea intake | rs1156588 | 2 | 58515375 | G | A | 0.21 | -0.015 | 0.003 | 2.90001e-09 | 447485 | 35.241 |
| Tea intake | rs1156954 | 7 | 117594838 | A | G | 0.324 | 0.01 | 0.002 | 3.89996e-06 | 447485 | 21.314 |
| Tea intake | rs11587444 | 1 | 150722844 | G | A | 0.393 | 0.014 | 0.002 | 1e-10 | 447485 | 41.788 |
| Tea intake | rs1160807 | 9 | 11379382 | T | C | 0.694 | 0.011 | 0.002 | 1.2e-06 | 447485 | 23.6 |
| Tea intake | rs11613633 | 12 | 112040904 | A | C | 0.165 | 0.015 | 0.003 | 7.39997e-07 | 447485 | 24.51 |
| Tea intake | rs11657857 | 17 | 15959537 | A | G | 0.148 | 0.014 | 0.003 | 4.30002e-06 | 447485 | 21.139 |
| Tea intake | rs11715828 | 3 | 71531708 | C | T | 0.481 | -0.01 | 0.002 | 1.09999e-06 | 447485 | 23.768 |
| Tea intake | rs11752836 | 6 | 11492836 | G | A | 0.536 | 0.011 | 0.002 | 2.1e-07 | 447485 | 26.983 |
| Tea intake | rs11878917 | 19 | 42588999 | A | G | 0.11 | 0.016 | 0.003 | 1.79999e-06 | 447485 | 22.796 |
| Tea intake | rs1197850 | 13 | 35930744 | G | T | 0.193 | 0.013 | 0.003 | 5.30005e-07 | 447485 | 25.155 |
| Tea intake | rs12357316 | 10 | 75760702 | G | A | 0.525 | 0.01 | 0.002 | 1.89998e-06 | 447485 | 22.722 |
| Tea intake | rs12446615 | 16 | 73915896 | G | A | 0.567 | -0.011 | 0.002 | 6.49995e-08 | 447485 | 29.202 |
| Tea intake | rs12591786 | 15 | 60902512 | T | C | 0.159 | -0.018 | 0.003 | 3.69999e-10 | 447485 | 39.274 |
| Tea intake | rs1275193 | 14 | 69466699 | A | C | 0.375 | 0.011 | 0.002 | 1.40001e-06 | 447485 | 23.275 |
| Tea intake | rs12752825 | 1 | 244015295 | G | A | 0.371 | -0.011 | 0.002 | 4.90004e-07 | 447485 | 25.284 |
| Tea intake | rs1285244 | 17 | 77797634 | G | A | 0.463 | 0.011 | 0.002 | 4.39997e-07 | 447485 | 25.495 |
| Tea intake | rs12920407 | 16 | 82867950 | A | G | 0.035 | -0.027 | 0.006 | 4.60002e-06 | 447485 | 20.986 |
| Tea intake | rs13270248 | 8 | 65428296 | G | A | 0.138 | 0.015 | 0.003 | 1.6e-06 | 447485 | 23.041 |
| Tea intake | rs13282783 | 8 | 22088975 | T | C | 0.286 | -0.014 | 0.002 | 7.90005e-09 | 447485 | 33.289 |
| Tea intake | rs13316505 | 3 | 96069913 | T | C | 0.071 | 0.019 | 0.004 | 2.90001e-06 | 447485 | 21.912 |
| Tea intake | rs140303651 | 3 | 127114320 | T | G | 0.008 | -0.06 | 0.013 | 3.79997e-06 | 447485 | 21.371 |
| Tea intake | rs141071726 | 7 | 17558580 | A | G | 0.027 | 0.041 | 0.007 | 2.19999e-09 | 447485 | 35.753 |
| Tea intake | rs1421488 | 10 | 107444714 | A | G | 0.382 | 0.011 | 0.002 | 9.69996e-07 | 447485 | 23.987 |
| Tea intake | rs142655113 | 9 | 34394209 | A | G | 0.152 | 0.014 | 0.003 | 4e-06 | 447485 | 21.266 |
| Tea intake | rs1428831 | 16 | 62904022 | G | A | 0.382 | 0.012 | 0.002 | 7.79992e-08 | 447485 | 28.855 |
| Tea intake | rs1433071 | 19 | 30913098 | G | T | 0.453 | -0.011 | 0.002 | 3.40001e-07 | 447485 | 26.015 |
| Tea intake | rs144412359 | 1 | 154559876 | C | T | 0.065 | 0.022 | 0.004 | 8.30004e-07 | 447485 | 24.291 |
| Tea intake | rs145322337 | 1 | 180067167 | A | G | 0.018 | 0.038 | 0.008 | 3.2e-06 | 447485 | 21.676 |
| Tea intake | rs1481012 | 4 | 89039082 | G | A | 0.112 | -0.026 | 0.003 | 5.30029e-15 | 447485 | 61.147 |
| Tea intake | rs149805207 | 6 | 137095269 | G | A | 0.009 | -0.072 | 0.013 | 1.09999e-08 | 447485 | 32.685 |
| Tea intake | rs1640297 | 22 | 20081852 | C | T | 0.496 | -0.01 | 0.002 | 1.2e-06 | 447485 | 23.654 |
| Tea intake | rs1712999 | 3 | 116131936 | G | A | 0.111 | 0.017 | 0.003 | 8.9e-07 | 447485 | 24.157 |
| Tea intake | rs17245213 | 11 | 1679769 | A | G | 0.208 | -0.015 | 0.003 | 2e-08 | 447485 | 31.521 |
| Tea intake | rs17576658 | 13 | 100272019 | A | G | 0.247 | -0.013 | 0.002 | 4.09996e-08 | 447485 | 30.116 |
| Tea intake | rs17655642 | 7 | 13329118 | C | T | 0.237 | 0.013 | 0.002 | 4.09996e-07 | 447485 | 25.629 |
| Tea intake | rs17685 | 7 | 75616105 | A | G | 0.278 | 0.023 | 0.002 | 1.59993e-22 | 447485 | 95.364 |
| Tea intake | rs1941356 | 18 | 31612400 | T | C | 0.638 | -0.01 | 0.002 | 3.79997e-06 | 447485 | 21.339 |
| Tea intake | rs1950709 | 14 | 29735938 | A | G | 0.541 | 0.01 | 0.002 | 9.59997e-07 | 447485 | 24.007 |
| Tea intake | rs2027239 | 1 | 95992206 | G | A | 0.873 | -0.016 | 0.003 | 7.29995e-07 | 447485 | 24.539 |
| Tea intake | rs2207055 | 6 | 78135205 | A | C | 0.351 | 0.011 | 0.002 | 8.40001e-07 | 447485 | 24.271 |
| Tea intake | rs224240 | 16 | 3316340 | A | G | 0.241 | -0.013 | 0.002 | 6.90001e-08 | 447485 | 29.079 |
| Tea intake | rs2251012 | 21 | 17633645 | C | T | 0.535 | -0.01 | 0.002 | 3.79997e-06 | 447485 | 21.357 |
| Tea intake | rs2279844 | 17 | 40819809 | A | G | 0.379 | -0.012 | 0.002 | 4e-08 | 447485 | 30.151 |
| Tea intake | rs2289002 | 1 | 214177979 | C | T | 0.287 | 0.011 | 0.002 | 4.09996e-06 | 447485 | 21.233 |
| Tea intake | rs2318540 | 4 | 67831581 | G | T | 0.573 | -0.01 | 0.002 | 2.1e-06 | 447485 | 22.512 |
| Tea intake | rs2351187 | 10 | 86850616 | A | G | 0.319 | 0.013 | 0.002 | 1.6e-08 | 447485 | 31.959 |
| Tea intake | rs2351822 | 10 | 87448297 | A | C | 0.505 | -0.011 | 0.002 | 2.39999e-07 | 447485 | 26.665 |
| Tea intake | rs2403304 | 5 | 103733250 | C | T | 0.371 | -0.011 | 0.002 | 4.30002e-07 | 447485 | 25.549 |
| Tea intake | rs2472297 | 15 | 75027880 | T | C | 0.262 | 0.053 | 0.002 | 2.30144e-109 | 447485 | 493.643 |
| Tea intake | rs2478875 | 6 | 51283110 | G | A | 0.209 | 0.022 | 0.003 | 5.10035e-17 | 447485 | 70.299 |
| Tea intake | rs2497305 | 10 | 94485621 | T | C | 0.476 | 0.01 | 0.002 | 7.29995e-07 | 447485 | 24.543 |
| Tea intake | rs2645929 | 13 | 56444529 | G | A | 0.813 | -0.015 | 0.003 | 3.50002e-08 | 447485 | 30.424 |
| Tea intake | rs2665811 | 17 | 61971635 | C | T | 0.638 | 0.011 | 0.002 | 5.49997e-07 | 447485 | 25.09 |
| Tea intake | rs2678228 | 2 | 50376687 | T | G | 0.506 | 0.01 | 0.002 | 2e-06 | 447485 | 22.625 |
| Tea intake | rs2839188 | 21 | 47692798 | T | C | 0.378 | 0.012 | 0.002 | 5.89997e-08 | 447485 | 29.404 |
| Tea intake | rs323065 | 16 | 2367878 | T | C | 0.174 | -0.013 | 0.003 | 2.99999e-06 | 447485 | 21.815 |
| Tea intake | rs34619 | 5 | 60465365 | A | G | 0.431 | 0.012 | 0.002 | 4.30002e-08 | 447485 | 30.021 |
| Tea intake | rs34940743 | 14 | 80102233 | G | A | 0.347 | 0.012 | 0.002 | 8.19993e-08 | 447485 | 28.748 |
| Tea intake | rs35596618 | 1 | 50504100 | G | A | 0.089 | -0.018 | 0.004 | 8.70001e-07 | 447485 | 24.205 |
| Tea intake | rs35970998 | 2 | 40558923 | G | A | 0.263 | -0.013 | 0.002 | 1.40001e-07 | 447485 | 27.778 |
| Tea intake | rs41135 | 5 | 96166026 | C | T | 0.472 | 0.01 | 0.002 | 4.49997e-06 | 447485 | 21.027 |
| Tea intake | rs4135060 | 12 | 104365450 | A | G | 0.109 | 0.017 | 0.003 | 8.30004e-07 | 447485 | 24.286 |
| Tea intake | rs4410790 | 7 | 17284577 | C | T | 0.631 | 0.041 | 0.002 | 3.40017e-76 | 447485 | 341.268 |
| Tea intake | rs457534 | 21 | 31343659 | C | T | 0.174 | -0.013 | 0.003 | 2.19999e-06 | 447485 | 22.443 |
| Tea intake | rs4683352 | 3 | 42213816 | C | T | 0.748 | 0.012 | 0.002 | 1.6e-06 | 447485 | 23.03 |
| Tea intake | rs4692053 | 4 | 28492865 | A | C | 0.35 | 0.011 | 0.002 | 2e-06 | 447485 | 22.587 |
| Tea intake | rs4808193 | 19 | 19410622 | C | T | 0.335 | 0.015 | 0.002 | 1.69981e-11 | 447485 | 45.24 |
| Tea intake | rs4817505 | 21 | 34343828 | C | T | 0.39 | 0.015 | 0.002 | 4.19952e-12 | 447485 | 48.012 |
| Tea intake | rs4862411 | 4 | 185664774 | C | T | 0.766 | 0.013 | 0.003 | 4e-07 | 447485 | 25.691 |
| Tea intake | rs56019762 | 10 | 10205581 | A | G | 0.265 | -0.012 | 0.002 | 2.90001e-07 | 447485 | 26.333 |
| Tea intake | rs56188862 | 1 | 174189269 | C | T | 0.387 | -0.016 | 0.002 | 4.30031e-13 | 447485 | 52.497 |
| Tea intake | rs57462170 | 3 | 50239803 | A | G | 0.109 | 0.019 | 0.003 | 1.89998e-08 | 447485 | 31.62 |
| Tea intake | rs57631352 | 19 | 4338173 | G | A | 0.297 | -0.013 | 0.002 | 1.7e-08 | 447485 | 31.868 |
| Tea intake | rs58864227 | 17 | 27658905 | T | C | 0.318 | 0.011 | 0.002 | 2.5e-06 | 447485 | 22.181 |
| Tea intake | rs6033239 | 20 | 11845930 | T | G | 0.362 | -0.012 | 0.002 | 1.79999e-07 | 447485 | 27.226 |
| Tea intake | rs603826 | 11 | 118970878 | C | A | 0.371 | 0.012 | 0.002 | 7.79992e-08 | 447485 | 28.867 |
| Tea intake | rs6129076 | 20 | 59787651 | T | C | 0.674 | 0.012 | 0.002 | 2.59998e-07 | 447485 | 26.554 |
| Tea intake | rs62092408 | 18 | 52753099 | A | G | 0.191 | 0.015 | 0.003 | 8.9e-08 | 447485 | 28.595 |
| Tea intake | rs6265 | 11 | 27679916 | T | C | 0.188 | -0.014 | 0.003 | 1.89998e-07 | 447485 | 27.103 |
| Tea intake | rs6822218 | 4 | 18246160 | G | A | 0.72 | -0.011 | 0.002 | 4.49997e-06 | 447485 | 21.043 |
| Tea intake | rs6829 | 13 | 111531264 | T | C | 0.596 | -0.012 | 0.002 | 3.69999e-08 | 447485 | 30.282 |
| Tea intake | rs7012814 | 8 | 9173358 | A | G | 0.474 | -0.011 | 0.002 | 3.79997e-07 | 447485 | 25.769 |
| Tea intake | rs7095819 | 10 | 131475845 | A | G | 0.305 | 0.011 | 0.002 | 1.7e-06 | 447485 | 22.889 |
| Tea intake | rs7172571 | 15 | 69590707 | A | G | 0.748 | 0.012 | 0.002 | 2.19999e-06 | 447485 | 22.421 |
| Tea intake | rs7248282 | 19 | 47769120 | T | C | 0.42 | -0.01 | 0.002 | 2.69998e-06 | 447485 | 22.019 |
| Tea intake | rs72733403 | 15 | 40630913 | T | C | 0.273 | 0.012 | 0.002 | 9.69996e-07 | 447485 | 23.98 |
| Tea intake | rs72748419 | 9 | 81821528 | G | T | 0.047 | 0.027 | 0.005 | 8.70001e-08 | 447485 | 28.638 |
| Tea intake | rs72797284 | 5 | 152031650 | G | A | 0.271 | -0.017 | 0.002 | 7.00003e-13 | 447485 | 51.558 |
| Tea intake | rs73187605 | 3 | 183990230 | T | C | 0.269 | 0.011 | 0.002 | 2.19999e-06 | 447485 | 22.412 |
| Tea intake | rs75396527 | 10 | 29998422 | A | G | 0.018 | 0.041 | 0.008 | 4e-07 | 447485 | 25.704 |
| Tea intake | rs7640488 | 3 | 69475108 | A | G | 0.637 | 0.011 | 0.002 | 6.80002e-07 | 447485 | 24.666 |
| Tea intake | rs7723040 | 5 | 142085997 | T | C | 0.091 | -0.018 | 0.004 | 8.30004e-07 | 447485 | 24.284 |
| Tea intake | rs7757102 | 6 | 137222671 | G | A | 0.555 | -0.012 | 0.002 | 3.09999e-08 | 447485 | 30.624 |
| Tea intake | rs7825157 | 8 | 93177709 | T | C | 0.766 | -0.013 | 0.003 | 2.39999e-07 | 447485 | 26.659 |
| Tea intake | rs7840343 | 8 | 103639193 | A | G | 0.287 | -0.011 | 0.002 | 2.80001e-06 | 447485 | 21.924 |
| Tea intake | rs79494947 | 5 | 178794691 | A | G | 0.047 | 0.026 | 0.005 | 8.09991e-07 | 447485 | 24.337 |
| Tea intake | rs80318442 | 8 | 34285545 | G | T | 0.048 | 0.025 | 0.005 | 5.39995e-07 | 447485 | 25.112 |
| Tea intake | rs897623 | 1 | 2721576 | G | A | 0.783 | -0.012 | 0.003 | 1.6e-06 | 447485 | 22.987 |
| Tea intake | rs901746 | 11 | 47260319 | G | A | 0.302 | 0.013 | 0.002 | 5.60003e-08 | 447485 | 29.511 |
| Tea intake | rs9262602 | 6 | 31020271 | G | A | 0.149 | 0.014 | 0.003 | 3.79997e-06 | 447485 | 21.345 |
| Tea intake | rs9438548 | 1 | 26757548 | T | C | 0.751 | 0.013 | 0.002 | 1.5e-07 | 447485 | 27.576 |
| Tea intake | rs9478663 | 6 | 155915741 | A | G | 0.215 | 0.012 | 0.003 | 2.69998e-06 | 447485 | 22.028 |
| Tea intake | rs9521405 | 13 | 89234115 | G | A | 0.625 | 0.01 | 0.002 | 2.1e-06 | 447485 | 22.504 |
| Tea intake | rs9624470 | 22 | 24820268 | A | G | 0.58 | 0.025 | 0.002 | 1.29987e-31 | 447485 | 136.839 |
| Tea intake | rs9648476 | 7 | 39293033 | A | G | 0.623 | 0.013 | 0.002 | 1.09999e-08 | 447485 | 32.722 |
| Tea intake | rs977474 | 12 | 11284772 | T | C | 0.834 | 0.022 | 0.003 | 2.39994e-14 | 447485 | 58.18 |
| Tea intake | rs984616 | 5 | 130891101 | T | C | 0.77 | 0.012 | 0.003 | 1.89998e-06 | 447485 | 22.662 |
| Tea intake | rs9937354 | 16 | 53799847 | A | G | 0.424 | -0.014 | 0.002 | 4.90004e-11 | 447485 | 43.231 |
| Moderate to vigorous physical activity levels | rs10145335 | 14 | 98547748 | A | G | 0.251 | 0.014 | 0.003 | 2.69998e-08 | 377234 | 30.878 |
| Moderate to vigorous physical activity levels | rs1043595 | 7 | 128410012 | A | G | 0.283 | -0.014 | 0.002 | 4.30002e-09 | 377234 | 34.481 |
| Moderate to vigorous physical activity levels | rs10506671 | 12 | 75522128 | A | G | 0.089 | 0.019 | 0.004 | 6.80002e-07 | 377234 | 24.665 |
| Moderate to vigorous physical activity levels | rs11048486 | 12 | 26524796 | A | C | 0.165 | -0.014 | 0.003 | 3.40001e-06 | 377234 | 21.572 |
| Moderate to vigorous physical activity levels | rs11048486 | 12 | 26524796 | A | C | 0.165 | -0.014 | 0.003 | 3.40001e-06 | 377234 | 21.572 |
| Moderate to vigorous physical activity levels | rs11081859 | 18 | 31926289 | A | G | 0.892 | -0.018 | 0.004 | 2.1e-07 | 377234 | 26.914 |
| Moderate to vigorous physical activity levels | rs112308536 | 17 | 2223997 | G | A | 0.321 | -0.011 | 0.002 | 4.90004e-06 | 377234 | 20.861 |
| Moderate to vigorous physical activity levels | rs114243593 | 5 | 155074831 | G | A | 0.053 | -0.027 | 0.005 | 5.49997e-08 | 377234 | 29.541 |
| Moderate to vigorous physical activity levels | rs115181288 | 1 | 114130641 | T | C | 0.055 | 0.023 | 0.005 | 2.30001e-06 | 377234 | 22.301 |
| Moderate to vigorous physical activity levels | rs11641100 | 16 | 52469793 | T | C | 0.444 | -0.012 | 0.002 | 1.6e-07 | 377234 | 27.413 |
| Moderate to vigorous physical activity levels | rs1186721 | 7 | 34974602 | A | G | 0.316 | 0.013 | 0.002 | 4.39997e-08 | 377234 | 29.984 |
| Moderate to vigorous physical activity levels | rs11913445 | 22 | 20142513 | A | C | 0.168 | -0.015 | 0.003 | 3.89996e-07 | 377234 | 25.759 |
| Moderate to vigorous physical activity levels | rs12912808 | 15 | 95292223 | T | C | 0.149 | -0.018 | 0.003 | 1.7e-08 | 377234 | 31.852 |
| Moderate to vigorous physical activity levels | rs12979056 | 19 | 17862131 | A | G | 0.458 | 0.01 | 0.002 | 2.30001e-06 | 377234 | 22.296 |
| Moderate to vigorous physical activity levels | rs1340704 | 13 | 76506657 | T | C | 0.598 | -0.01 | 0.002 | 4.70002e-06 | 377234 | 20.971 |
| Moderate to vigorous physical activity levels | rs139907649 | 14 | 90289275 | A | G | 0.009 | 0.059 | 0.012 | 1.5e-06 | 377234 | 23.114 |
| Moderate to vigorous physical activity levels | rs150863806 | 1 | 40949997 | A | G | 0.031 | -0.032 | 0.007 | 1.29999e-06 | 377234 | 23.414 |
| Moderate to vigorous physical activity levels | rs161344 | 7 | 137045731 | A | C | 0.432 | 0.01 | 0.002 | 4e-06 | 377234 | 21.266 |
| Moderate to vigorous physical activity levels | rs17183317 | 4 | 171734020 | G | A | 0.063 | 0.022 | 0.005 | 2.1e-06 | 377234 | 22.49 |
| Moderate to vigorous physical activity levels | rs17573850 | 13 | 44885589 | G | A | 0.259 | -0.012 | 0.003 | 1.7e-06 | 377234 | 22.909 |
| Moderate to vigorous physical activity levels | rs1873585 | 3 | 173641375 | G | A | 0.222 | 0.012 | 0.003 | 4.39997e-06 | 377234 | 21.091 |
| Moderate to vigorous physical activity levels | rs1921981 | 21 | 42422547 | A | G | 0.326 | -0.013 | 0.002 | 3.79997e-08 | 377234 | 30.224 |
| Moderate to vigorous physical activity levels | rs1972763 | 4 | 159860563 | T | C | 0.658 | -0.013 | 0.002 | 3.29997e-08 | 377234 | 30.526 |
| Moderate to vigorous physical activity levels | rs1974771 | 2 | 54278543 | A | G | 0.1 | 0.021 | 0.004 | 6.59994e-09 | 377234 | 33.654 |
| Moderate to vigorous physical activity levels | rs2035562 | 3 | 85056521 | G | A | 0.672 | 0.014 | 0.002 | 3.89996e-09 | 377234 | 34.687 |
| Moderate to vigorous physical activity levels | rs2114286 | 3 | 41194283 | G | A | 0.534 | 0.012 | 0.002 | 3.29997e-08 | 377234 | 30.501 |
| Moderate to vigorous physical activity levels | rs2148201 | 10 | 2323505 | A | G | 0.491 | 0.011 | 0.002 | 1.7e-06 | 377234 | 22.874 |
| Moderate to vigorous physical activity levels | rs2267443 | 22 | 42287454 | G | A | 0.619 | -0.011 | 0.002 | 3.40001e-06 | 377234 | 21.582 |
| Moderate to vigorous physical activity levels | rs2267549 | 12 | 4771682 | A | G | 0.454 | -0.011 | 0.002 | 2.80001e-06 | 377234 | 21.964 |
| Moderate to vigorous physical activity levels | rs2283249 | 11 | 17824372 | G | T | 0.248 | 0.013 | 0.003 | 1.09999e-06 | 377234 | 23.81 |
| Moderate to vigorous physical activity levels | rs2388195 | 6 | 98283960 | C | A | 0.439 | 0.012 | 0.002 | 1.7e-07 | 377234 | 27.401 |
| Moderate to vigorous physical activity levels | rs2451292 | 2 | 163924441 | G | T | 0.527 | 0.01 | 0.002 | 4e-06 | 377234 | 21.264 |
| Moderate to vigorous physical activity levels | rs2494664 | 1 | 154057802 | G | A | 0.46 | 0.012 | 0.002 | 8.9e-08 | 377234 | 28.606 |
| Moderate to vigorous physical activity levels | rs2638463 | 12 | 89669785 | A | G | 0.306 | 0.012 | 0.003 | 4.60002e-06 | 377234 | 21.016 |
| Moderate to vigorous physical activity levels | rs2721937 | 8 | 116633699 | T | C | 0.185 | 0.014 | 0.003 | 1.40001e-06 | 377234 | 23.301 |
| Moderate to vigorous physical activity levels | rs2764261 | 6 | 108927842 | G | A | 0.626 | -0.011 | 0.002 | 6.29999e-07 | 377234 | 24.833 |
| Moderate to vigorous physical activity levels | rs293567 | 20 | 31098565 | T | G | 0.331 | 0.011 | 0.002 | 1.79999e-06 | 377234 | 22.799 |
| Moderate to vigorous physical activity levels | rs2942127 | 1 | 204420067 | A | G | 0.825 | -0.016 | 0.003 | 3.29997e-08 | 377234 | 30.522 |
| Moderate to vigorous physical activity levels | rs2988004 | 9 | 37044388 | G | T | 0.442 | 0.013 | 0.002 | 4.09996e-09 | 377234 | 34.579 |
| Moderate to vigorous physical activity levels | rs35650232 | 19 | 49259973 | G | T | 0.221 | 0.013 | 0.003 | 2.5e-06 | 377234 | 22.14 |
| Moderate to vigorous physical activity levels | rs3810496 | 20 | 62406886 | C | T | 0.616 | -0.011 | 0.002 | 1.5e-06 | 377234 | 23.128 |
| Moderate to vigorous physical activity levels | rs3936938 | 1 | 181031797 | A | G | 0.597 | 0.011 | 0.002 | 1.6e-06 | 377234 | 23.044 |
| Moderate to vigorous physical activity levels | rs4281659 | 15 | 61844443 | C | T | 0.135 | -0.017 | 0.003 | 1e-07 | 377234 | 28.31 |
| Moderate to vigorous physical activity levels | rs429358 | 19 | 45411941 | C | T | 0.154 | 0.022 | 0.003 | 6.09958e-13 | 377234 | 51.824 |
| Moderate to vigorous physical activity levels | rs4304660 | 10 | 22295747 | A | G | 0.727 | 0.011 | 0.002 | 4.49997e-06 | 377234 | 21.045 |
| Moderate to vigorous physical activity levels | rs4787924 | 16 | 24264538 | A | G | 0.525 | 0.011 | 0.002 | 8.9e-07 | 377234 | 24.158 |
| Moderate to vigorous physical activity levels | rs4800990 | 18 | 53197944 | T | C | 0.566 | 0.011 | 0.002 | 1.89998e-06 | 377234 | 22.694 |
| Moderate to vigorous physical activity levels | rs4850842 | 2 | 199198626 | A | G | 0.57 | 0.012 | 0.002 | 2.19999e-07 | 377234 | 26.816 |
| Moderate to vigorous physical activity levels | rs4872389 | 8 | 25909499 | T | C | 0.23 | 0.012 | 0.003 | 4.70002e-06 | 377234 | 20.962 |
| Moderate to vigorous physical activity levels | rs4886868 | 15 | 74353561 | G | T | 0.586 | 0.012 | 0.002 | 3.50002e-08 | 377234 | 30.404 |
| Moderate to vigorous physical activity levels | rs4906229 | 14 | 103057511 | C | A | 0.755 | 0.013 | 0.003 | 5.49997e-07 | 377234 | 25.085 |
| Moderate to vigorous physical activity levels | rs527737 | 11 | 65884800 | T | C | 0.578 | 0.012 | 0.002 | 6.1e-08 | 377234 | 29.338 |
| Moderate to vigorous physical activity levels | rs61780535 | 1 | 31620930 | T | C | 0.202 | -0.014 | 0.003 | 2.99999e-07 | 377234 | 26.278 |
| Moderate to vigorous physical activity levels | rs61928076 | 12 | 53730164 | A | G | 0.045 | 0.024 | 0.005 | 4.39997e-06 | 377234 | 21.071 |
| Moderate to vigorous physical activity levels | rs61975407 | 14 | 55568276 | T | C | 0.548 | 0.011 | 0.002 | 2e-06 | 377234 | 22.567 |
| Moderate to vigorous physical activity levels | rs6497882 | 16 | 25692849 | G | A | 0.596 | -0.011 | 0.002 | 3.29997e-06 | 377234 | 21.649 |
| Moderate to vigorous physical activity levels | rs662638 | 11 | 57686764 | C | T | 0.556 | -0.012 | 0.002 | 1.29999e-07 | 377234 | 27.871 |
| Moderate to vigorous physical activity levels | rs67432617 | 8 | 104812820 | C | A | 0.195 | -0.015 | 0.003 | 5.1e-08 | 377234 | 29.691 |
| Moderate to vigorous physical activity levels | rs6743932 | 2 | 35005351 | A | G | 0.197 | 0.013 | 0.003 | 4.20001e-06 | 377234 | 21.186 |
| Moderate to vigorous physical activity levels | rs6812265 | 4 | 139726791 | T | C | 0.452 | -0.011 | 0.002 | 3.29997e-07 | 377234 | 26.086 |
| Moderate to vigorous physical activity levels | rs710187 | 22 | 38204089 | T | C | 0.558 | -0.01 | 0.002 | 2.19999e-06 | 377234 | 22.375 |
| Moderate to vigorous physical activity levels | rs719263 | 22 | 49685455 | C | T | 0.805 | 0.013 | 0.003 | 2.80001e-06 | 377234 | 21.977 |
| Moderate to vigorous physical activity levels | rs7233752 | 18 | 67399208 | G | A | 0.195 | -0.013 | 0.003 | 3.79997e-06 | 377234 | 21.381 |
| Moderate to vigorous physical activity levels | rs72638967 | 4 | 65176402 | T | C | 0.019 | 0.038 | 0.008 | 2.30001e-06 | 377234 | 22.314 |
| Moderate to vigorous physical activity levels | rs72920838 | 18 | 49514720 | T | C | 0.111 | -0.017 | 0.004 | 1.89998e-06 | 377234 | 22.668 |
| Moderate to vigorous physical activity levels | rs7326482 | 13 | 54037803 | T | G | 0.615 | 0.013 | 0.002 | 1.6e-08 | 377234 | 31.915 |
| Moderate to vigorous physical activity levels | rs749256 | 1 | 156884365 | C | T | 0.75 | -0.013 | 0.003 | 3.79997e-07 | 377234 | 25.785 |
| Moderate to vigorous physical activity levels | rs7508365 | 19 | 13151477 | C | T | 0.72 | 0.011 | 0.002 | 3.59998e-06 | 377234 | 21.487 |
| Moderate to vigorous physical activity levels | rs7652008 | 3 | 127160434 | C | T | 0.463 | 0.011 | 0.002 | 1.6e-06 | 377234 | 22.983 |
| Moderate to vigorous physical activity levels | rs7690846 | 4 | 113988045 | C | T | 0.516 | -0.01 | 0.002 | 2.69998e-06 | 377234 | 22.019 |
| Moderate to vigorous physical activity levels | rs77742115 | 5 | 18330424 | C | T | 0.138 | 0.018 | 0.003 | 9.59997e-09 | 377234 | 32.922 |
| Moderate to vigorous physical activity levels | rs7804463 | 7 | 133447651 | C | T | 0.47 | -0.015 | 0.002 | 1.20005e-11 | 377234 | 45.987 |
| Moderate to vigorous physical activity levels | rs80014012 | 10 | 86318511 | C | A | 0.075 | 0.02 | 0.004 | 2.30001e-06 | 377234 | 22.298 |
| Moderate to vigorous physical activity levels | rs862490 | 17 | 35850275 | C | T | 0.642 | 0.011 | 0.002 | 2.69998e-06 | 377234 | 22 |
| Moderate to vigorous physical activity levels | rs877483 | 3 | 53846741 | C | T | 0.567 | -0.012 | 0.002 | 4e-08 | 377234 | 30.132 |
| Moderate to vigorous physical activity levels | rs921915 | 7 | 50228581 | C | T | 0.588 | 0.014 | 0.002 | 5.69994e-10 | 377234 | 38.436 |
| Moderate to vigorous physical activity levels | rs9446663 | 6 | 73180747 | A | C | 0.296 | -0.013 | 0.002 | 1.6e-07 | 377234 | 27.459 |
| Moderate to vigorous physical activity levels | rs9579775 | 13 | 20616557 | C | A | 0.135 | -0.016 | 0.003 | 2.80001e-06 | 377234 | 21.95 |
| Moderate to vigorous physical activity levels | rs9584870 | 13 | 99245866 | C | T | 0.367 | 0.011 | 0.002 | 2.39999e-06 | 377234 | 22.238 |
| Moderate to vigorous physical activity levels | rs974471 | 14 | 29685328 | A | G | 0.771 | -0.014 | 0.003 | 2.19999e-07 | 377234 | 26.851 |
| Moderate to vigorous physical activity levels | rs9919769 | 12 | 126827971 | G | A | 0.226 | 0.012 | 0.003 | 2.5e-06 | 377234 | 22.142 |
| Active to sedentary transition probability | rs143524086 | 4 | 72790486 | G | T | 0.033 | -0.06 | 0.013 | 4.29794e-06 | 88411 | 21.127 |
| Active to sedentary transition probability | rs1572504 | 1 | 219859210 | T | C | 0.202 | 0.028 | 0.006 | 8.20012e-07 | 88411 | 24.31 |
| Active to sedentary transition probability | rs157595 | 19 | 45425460 | G | A | 0.62 | -0.023 | 0.005 | 4.58501e-06 | 88411 | 21.003 |
| Active to sedentary transition probability | rs1923227 | 1 | 73847345 | A | G | 0.831 | -0.03 | 0.006 | 1.27779e-06 | 88411 | 23.456 |
| Active to sedentary transition probability | rs2381684 | 2 | 145767243 | A | G | 0.254 | -0.025 | 0.005 | 4.4414e-06 | 88411 | 21.064 |
| Active to sedentary transition probability | rs3025316 | 9 | 136459543 | C | T | 0.115 | 0.035 | 0.007 | 1.70789e-06 | 88411 | 22.898 |
| Active to sedentary transition probability | rs306046 | 19 | 3041263 | G | T | 0.502 | 0.022 | 0.005 | 4.17657e-06 | 88411 | 21.182 |
| Active to sedentary transition probability | rs3218036 | 19 | 30305684 | A | G | 0.328 | 0.024 | 0.005 | 1.9139e-06 | 88411 | 22.679 |
| Active to sedentary transition probability | rs34910 | 3 | 10453423 | G | T | 0.275 | -0.031 | 0.005 | 4.31231e-09 | 88411 | 34.477 |
| Active to sedentary transition probability | rs446219 | 5 | 88005827 | C | T | 0.581 | -0.027 | 0.005 | 7.43413e-09 | 88411 | 33.417 |
| Active to sedentary transition probability | rs4951261 | 1 | 205717823 | C | A | 0.394 | 0.025 | 0.005 | 1.08186e-07 | 88411 | 28.221 |
| Active to sedentary transition probability | rs56044017 | 5 | 151663784 | T | C | 0.175 | 0.034 | 0.006 | 2.39718e-08 | 88411 | 31.142 |
| Active to sedentary transition probability | rs61776614 | 1 | 2166406 | T | C | 0.067 | -0.043 | 0.009 | 4.40788e-06 | 88411 | 21.078 |
| Active to sedentary transition probability | rs61953497 | 12 | 121909573 | C | T | 0.401 | -0.023 | 0.005 | 1.41104e-06 | 88411 | 23.265 |
| Active to sedentary transition probability | rs62256108 | 3 | 68927142 | A | G | 0.078 | 0.042 | 0.009 | 1.61332e-06 | 88411 | 23.007 |
| Active to sedentary transition probability | rs6600346 | 1 | 41035785 | G | A | 0.337 | -0.023 | 0.005 | 3.13856e-06 | 88411 | 21.729 |
| Active to sedentary transition probability | rs7272683 | 20 | 17620092 | C | T | 0.359 | 0.024 | 0.005 | 1.24598e-06 | 88411 | 23.504 |
| Active to sedentary transition probability | rs74981057 | 5 | 138171216 | G | A | 0.011 | 0.102 | 0.022 | 4.14639e-06 | 88411 | 21.195 |
| Active to sedentary transition probability | rs76385837 | 14 | 34257954 | A | G | 0.023 | -0.074 | 0.016 | 2.15357e-06 | 88411 | 22.452 |
| Sleeplessness / insomnia | rs10032268 | 4 | 172837476 | T | C | 0.435 | 0.007 | 0.002 | 3e-06 | 462341 | 21.816 |
| Sleeplessness / insomnia | rs10184819 | 2 | 35562699 | A | G | 0.707 | 0.007 | 0.002 | 4.5e-06 | 462341 | 21.044 |
| Sleeplessness / insomnia | rs10235198 | 7 | 88311477 | T | C | 0.116 | 0.013 | 0.002 | 7.7e-08 | 462341 | 28.872 |
| Sleeplessness / insomnia | rs10276441 | 7 | 52581385 | A | G | 0.785 | 0.01 | 0.002 | 1.1e-07 | 462341 | 28.198 |
| Sleeplessness / insomnia | rs1083562 | 5 | 153002796 | C | T | 0.284 | -0.009 | 0.002 | 2.3e-07 | 462341 | 26.759 |
| Sleeplessness / insomnia | rs10838708 | 11 | 47441513 | A | G | 0.459 | -0.009 | 0.002 | 2.9e-10 | 462341 | 39.769 |
| Sleeplessness / insomnia | rs10891492 | 11 | 112909745 | T | C | 0.388 | 0.007 | 0.002 | 2.5e-06 | 462341 | 22.177 |
| Sleeplessness / insomnia | rs10947690 | 6 | 37631768 | G | A | 0.259 | 0.009 | 0.002 | 5.7e-08 | 462341 | 29.456 |
| Sleeplessness / insomnia | rs11097861 | 4 | 105330133 | G | A | 0.716 | 0.01 | 0.002 | 1.1e-09 | 462341 | 37.077 |
| Sleeplessness / insomnia | rs11152363 | 18 | 53057188 | A | G | 0.186 | 0.016 | 0.002 | 4.5e-16 | 462341 | 65.992 |
| Sleeplessness / insomnia | rs11220675 | 11 | 99493578 | A | G | 0.665 | -0.008 | 0.002 | 9.2e-07 | 462341 | 24.094 |
| Sleeplessness / insomnia | rs113851554 | 2 | 66750564 | T | G | 0.057 | 0.047 | 0.003 | 2.9e-45 | 462341 | 199.344 |
| Sleeplessness / insomnia | rs11628001 | 14 | 36906292 | A | C | 0.265 | -0.009 | 0.002 | 1.6e-07 | 462341 | 27.429 |
| Sleeplessness / insomnia | rs11635495 | 15 | 67804682 | C | T | 0.512 | 0.009 | 0.001 | 2.8e-10 | 462341 | 39.837 |
| Sleeplessness / insomnia | rs117525783 | 12 | 41991725 | A | G | 0.012 | -0.034 | 0.007 | 1.6e-06 | 462341 | 22.973 |
| Sleeplessness / insomnia | rs11786846 | 8 | 12837011 | A | G | 0.275 | -0.008 | 0.002 | 2.2e-06 | 462341 | 22.445 |
| Sleeplessness / insomnia | rs11790060 | 9 | 96202932 | C | T | 0.331 | -0.01 | 0.002 | 5.8e-11 | 462341 | 42.9 |
| Sleeplessness / insomnia | rs118145996 | 7 | 21241077 | T | C | 0.089 | -0.012 | 0.003 | 4.7e-06 | 462341 | 20.969 |
| Sleeplessness / insomnia | rs11871607 | 17 | 29140186 | G | A | 0.192 | 0.009 | 0.002 | 6e-07 | 462341 | 24.92 |
| Sleeplessness / insomnia | rs11946613 | 4 | 43964898 | G | A | 0.71 | -0.008 | 0.002 | 1.5e-06 | 462341 | 23.115 |
| Sleeplessness / insomnia | rs11963889 | 6 | 46318568 | C | T | 0.503 | -0.008 | 0.001 | 3.3e-07 | 462341 | 26.092 |
| Sleeplessness / insomnia | rs12470989 | 2 | 200955823 | G | A | 0.204 | -0.01 | 0.002 | 2.8e-08 | 462341 | 30.826 |
| Sleeplessness / insomnia | rs12484368 | 22 | 22451791 | T | C | 0.015 | -0.032 | 0.006 | 1.8e-07 | 462341 | 27.186 |
| Sleeplessness / insomnia | rs12526086 | 6 | 14105897 | A | G | 0.222 | -0.008 | 0.002 | 2.8e-06 | 462341 | 21.979 |
| Sleeplessness / insomnia | rs12599233 | 16 | 77154842 | T | G | 0.079 | -0.013 | 0.003 | 3.1e-06 | 462341 | 21.762 |
| Sleeplessness / insomnia | rs12739601 | 1 | 15730693 | G | A | 0.147 | 0.01 | 0.002 | 1.9e-06 | 462341 | 22.661 |
| Sleeplessness / insomnia | rs12771201 | 10 | 117888887 | C | T | 0.398 | 0.007 | 0.002 | 1.2e-06 | 462341 | 23.51 |
| Sleeplessness / insomnia | rs12908707 | 15 | 69895237 | C | T | 0.152 | -0.01 | 0.002 | 4.3e-06 | 462341 | 21.139 |
| Sleeplessness / insomnia | rs12957494 | 18 | 24399982 | C | T | 0.068 | 0.014 | 0.003 | 4.1e-06 | 462341 | 21.216 |
| Sleeplessness / insomnia | rs12994048 | 2 | 164536886 | A | G | 0.681 | -0.008 | 0.002 | 1.8e-06 | 462341 | 22.85 |
| Sleeplessness / insomnia | rs13215045 | 6 | 153447516 | T | C | 0.3 | -0.008 | 0.002 | 5.6e-07 | 462341 | 25.052 |
| Sleeplessness / insomnia | rs13253336 | 8 | 103254690 | C | T | 0.285 | -0.008 | 0.002 | 3.9e-06 | 462341 | 21.302 |
| Sleeplessness / insomnia | rs140128342 | 4 | 92398283 | T | C | 0.135 | 0.011 | 0.002 | 8.2e-07 | 462341 | 24.318 |
| Sleeplessness / insomnia | rs142357434 | 12 | 120866011 | T | C | 0.099 | -0.012 | 0.002 | 1.5e-06 | 462341 | 23.204 |
| Sleeplessness / insomnia | rs1430205 | 5 | 87678585 | T | C | 0.462 | 0.009 | 0.001 | 2.1e-10 | 462341 | 40.379 |
| Sleeplessness / insomnia | rs1452374 | 4 | 82282233 | C | T | 0.314 | 0.008 | 0.002 | 1.1e-06 | 462341 | 23.755 |
| Sleeplessness / insomnia | rs145524905 | 10 | 46227201 | G | A | 0.021 | 0.027 | 0.006 | 4.7e-06 | 462341 | 20.944 |
| Sleeplessness / insomnia | rs148769295 | 11 | 72238072 | G | T | 0.034 | -0.021 | 0.004 | 6.7e-07 | 462341 | 24.708 |
| Sleeplessness / insomnia | rs1533522 | 2 | 215507078 | A | G | 0.14 | -0.01 | 0.002 | 1.5e-06 | 462341 | 23.131 |
| Sleeplessness / insomnia | rs1547630 | 13 | 112642528 | A | G | 0.652 | 0.009 | 0.002 | 5.8e-09 | 462341 | 33.894 |
| Sleeplessness / insomnia | rs15870 | 14 | 55619311 | G | A | 0.412 | -0.007 | 0.002 | 7.7e-07 | 462341 | 24.441 |
| Sleeplessness / insomnia | rs1670131 | 18 | 6315744 | T | G | 0.17 | 0.01 | 0.002 | 8.7e-07 | 462341 | 24.201 |
| Sleeplessness / insomnia | rs16854819 | 3 | 143736126 | G | A | 0.335 | 0.007 | 0.002 | 2e-06 | 462341 | 22.595 |
| Sleeplessness / insomnia | rs17139246 | 16 | 6106260 | C | T | 0.39 | 0.008 | 0.002 | 3.1e-07 | 462341 | 26.217 |
| Sleeplessness / insomnia | rs17151854 | 8 | 10236559 | T | G | 0.152 | 0.013 | 0.002 | 3.8e-10 | 462341 | 39.211 |
| Sleeplessness / insomnia | rs176644 | 15 | 89913632 | T | G | 0.405 | 0.008 | 0.002 | 1.1e-07 | 462341 | 28.107 |
| Sleeplessness / insomnia | rs17709610 | 10 | 104250278 | G | A | 0.298 | -0.01 | 0.002 | 9.5e-10 | 462341 | 37.431 |
| Sleeplessness / insomnia | rs1867814 | 2 | 116563438 | A | G | 0.503 | -0.008 | 0.001 | 2.4e-07 | 462341 | 26.674 |
| Sleeplessness / insomnia | rs1988337 | 4 | 91292200 | G | A | 0.552 | 0.008 | 0.001 | 2.1e-08 | 462341 | 31.413 |
| Sleeplessness / insomnia | rs2014830 | 3 | 50172397 | T | C | 0.304 | -0.012 | 0.002 | 8.9e-13 | 462341 | 51.08 |
| Sleeplessness / insomnia | rs2051821 | 17 | 46143515 | C | T | 0.207 | -0.009 | 0.002 | 3.3e-06 | 462341 | 21.658 |
| Sleeplessness / insomnia | rs2062113 | 16 | 59476179 | C | T | 0.568 | -0.01 | 0.002 | 1.6e-10 | 462341 | 40.953 |
| Sleeplessness / insomnia | rs2110119 | 19 | 2668611 | A | G | 0.324 | -0.008 | 0.002 | 3.5e-07 | 462341 | 25.925 |
| Sleeplessness / insomnia | rs224032 | 10 | 64521829 | A | G | 0.55 | 0.008 | 0.001 | 1.8e-08 | 462341 | 31.666 |
| Sleeplessness / insomnia | rs2270914 | 5 | 1442979 | T | C | 0.244 | -0.009 | 0.002 | 1.5e-07 | 462341 | 27.647 |
| Sleeplessness / insomnia | rs240112 | 6 | 101059253 | A | C | 0.55 | -0.008 | 0.001 | 2.9e-07 | 462341 | 26.31 |
| Sleeplessness / insomnia | rs2480336 | 10 | 19064736 | A | G | 0.254 | 0.009 | 0.002 | 5.2e-07 | 462341 | 25.17 |
| Sleeplessness / insomnia | rs2487512 | 10 | 22137880 | C | A | 0.697 | -0.008 | 0.002 | 2.8e-06 | 462341 | 21.928 |
| Sleeplessness / insomnia | rs2501279 | 1 | 22368342 | T | C | 0.603 | 0.008 | 0.002 | 5.8e-07 | 462341 | 24.976 |
| Sleeplessness / insomnia | rs2596704 | 9 | 125647367 | C | T | 0.872 | 0.01 | 0.002 | 4.3e-06 | 462341 | 21.116 |
| Sleeplessness / insomnia | rs2604551 | 4 | 15091201 | G | T | 0.64 | -0.008 | 0.002 | 4.7e-08 | 462341 | 29.846 |
| Sleeplessness / insomnia | rs27704 | 5 | 123146472 | A | G | 0.222 | 0.009 | 0.002 | 6.1e-07 | 462341 | 24.877 |
| Sleeplessness / insomnia | rs2838788 | 21 | 46542374 | C | T | 0.394 | -0.007 | 0.002 | 9.9e-07 | 462341 | 23.956 |
| Sleeplessness / insomnia | rs2881926 | 2 | 20391853 | T | C | 0.53 | 0.007 | 0.001 | 4e-06 | 462341 | 21.287 |
| Sleeplessness / insomnia | rs302165 | 7 | 18323899 | A | G | 0.784 | -0.008 | 0.002 | 4.6e-06 | 462341 | 20.985 |
| Sleeplessness / insomnia | rs304780 | 7 | 80014145 | A | C | 0.641 | -0.007 | 0.002 | 2.9e-06 | 462341 | 21.901 |
| Sleeplessness / insomnia | rs314280 | 6 | 105400837 | G | A | 0.547 | 0.01 | 0.001 | 7.3e-11 | 462341 | 42.424 |
| Sleeplessness / insomnia | rs324017 | 12 | 57487814 | C | A | 0.705 | -0.01 | 0.002 | 1.4e-09 | 462341 | 36.693 |
| Sleeplessness / insomnia | rs34117109 | 11 | 113770811 | C | A | 0.402 | 0.007 | 0.002 | 3e-06 | 462341 | 21.813 |
| Sleeplessness / insomnia | rs35198836 | 16 | 1244631 | T | C | 0.29 | 0.009 | 0.002 | 9.2e-08 | 462341 | 28.528 |
| Sleeplessness / insomnia | rs35267450 | 1 | 18429405 | C | T | 0.286 | 0.009 | 0.002 | 5.4e-08 | 462341 | 29.567 |
| Sleeplessness / insomnia | rs35300422 | 1 | 238264437 | G | A | 0.111 | 0.012 | 0.002 | 1.2e-06 | 462341 | 23.632 |
| Sleeplessness / insomnia | rs36116812 | 5 | 141277172 | C | T | 0.497 | 0.008 | 0.001 | 1.6e-07 | 462341 | 27.525 |
| Sleeplessness / insomnia | rs3862230 | 1 | 44855891 | C | T | 0.319 | 0.008 | 0.002 | 9.7e-07 | 462341 | 23.98 |
| Sleeplessness / insomnia | rs4312180 | 13 | 40240381 | C | T | 0.386 | 0.007 | 0.002 | 4.5e-06 | 462341 | 21.04 |
| Sleeplessness / insomnia | rs4572538 | 2 | 147423012 | T | C | 0.364 | -0.01 | 0.002 | 7.7e-10 | 462341 | 37.839 |
| Sleeplessness / insomnia | rs4577309 | 2 | 191288833 | G | A | 0.534 | -0.009 | 0.001 | 1e-08 | 462341 | 32.82 |
| Sleeplessness / insomnia | rs4744336 | 9 | 97147851 | A | G | 0.525 | 0.007 | 0.001 | 4.1e-06 | 462341 | 21.213 |
| Sleeplessness / insomnia | rs4748580 | 10 | 19744849 | T | C | 0.364 | 0.008 | 0.002 | 2.6e-07 | 462341 | 26.501 |
| Sleeplessness / insomnia | rs4831364 | 8 | 12613884 | C | T | 0.91 | -0.014 | 0.003 | 2.1e-07 | 462341 | 26.935 |
| Sleeplessness / insomnia | rs518143 | 11 | 83335764 | G | A | 0.361 | -0.008 | 0.002 | 3.9e-07 | 462341 | 25.73 |
| Sleeplessness / insomnia | rs533757 | 11 | 107397587 | C | T | 0.561 | 0.008 | 0.001 | 3.1e-07 | 462341 | 26.163 |
| Sleeplessness / insomnia | rs544566 | 11 | 18065861 | C | T | 0.55 | -0.008 | 0.001 | 9.6e-08 | 462341 | 28.452 |
| Sleeplessness / insomnia | rs55788848 | 2 | 24268721 | T | C | 0.203 | 0.009 | 0.002 | 6.3e-07 | 462341 | 24.83 |
| Sleeplessness / insomnia | rs56093896 | 2 | 114103966 | A | C | 0.214 | -0.012 | 0.002 | 7.7e-12 | 462341 | 46.835 |
| Sleeplessness / insomnia | rs56330606 | 19 | 37673953 | G | A | 0.379 | 0.009 | 0.002 | 1.2e-09 | 462341 | 37 |
| Sleeplessness / insomnia | rs56356382 | 19 | 4064057 | C | T | 0.192 | -0.009 | 0.002 | 1.5e-06 | 462341 | 23.093 |
| Sleeplessness / insomnia | rs56365214 | 2 | 58930167 | A | C | 0.156 | -0.015 | 0.002 | 5.6e-13 | 462341 | 51.97 |
| Sleeplessness / insomnia | rs56938914 | 15 | 96965827 | C | T | 0.435 | -0.007 | 0.002 | 3.2e-06 | 462341 | 21.686 |
| Sleeplessness / insomnia | rs57985356 | 17 | 7735063 | G | T | 0.085 | -0.013 | 0.003 | 5.6e-07 | 462341 | 25.048 |
| Sleeplessness / insomnia | rs58380455 | 2 | 230944033 | T | C | 0.157 | 0.01 | 0.002 | 1.6e-06 | 462341 | 22.984 |
| Sleeplessness / insomnia | rs61771936 | 1 | 73266056 | A | G | 0.171 | -0.01 | 0.002 | 1.3e-06 | 462341 | 23.401 |
| Sleeplessness / insomnia | rs62228474 | 3 | 25048097 | A | G | 0.281 | -0.008 | 0.002 | 1.3e-06 | 462341 | 23.481 |
| Sleeplessness / insomnia | rs641325 | 11 | 57681828 | G | T | 0.668 | -0.007 | 0.002 | 2.9e-06 | 462341 | 21.856 |
| Sleeplessness / insomnia | rs6486359 | 11 | 9465619 | C | T | 0.43 | -0.008 | 0.002 | 2.2e-07 | 462341 | 26.823 |
| Sleeplessness / insomnia | rs6690017 | 1 | 57842729 | G | T | 0.409 | -0.01 | 0.002 | 1.1e-11 | 462341 | 46.216 |
| Sleeplessness / insomnia | rs67072353 | 20 | 60940702 | A | G | 0.224 | -0.008 | 0.002 | 3.5e-06 | 462341 | 21.52 |
| Sleeplessness / insomnia | rs6744461 | 2 | 145299297 | C | A | 0.679 | 0.008 | 0.002 | 7.5e-07 | 462341 | 24.484 |
| Sleeplessness / insomnia | rs68094047 | 12 | 109855201 | T | C | 0.251 | 0.01 | 0.002 | 1.7e-09 | 462341 | 36.248 |
| Sleeplessness / insomnia | rs6833329 | 4 | 7318704 | G | A | 0.611 | -0.008 | 0.002 | 8.4e-08 | 462341 | 28.712 |
| Sleeplessness / insomnia | rs6875751 | 5 | 3104936 | A | G | 0.072 | -0.014 | 0.003 | 7.4e-07 | 462341 | 24.519 |
| Sleeplessness / insomnia | rs6938026 | 6 | 43185733 | G | A | 0.21 | 0.01 | 0.002 | 7e-08 | 462341 | 29.063 |
| Sleeplessness / insomnia | rs6975972 | 7 | 1070468 | G | A | 0.579 | -0.009 | 0.002 | 2e-09 | 462341 | 35.96 |
| Sleeplessness / insomnia | rs7029454 | 9 | 134778509 | C | A | 0.888 | -0.011 | 0.002 | 1.9e-06 | 462341 | 22.685 |
| Sleeplessness / insomnia | rs7138222 | 12 | 70806981 | C | A | 0.29 | 0.008 | 0.002 | 5.3e-07 | 462341 | 25.142 |
| Sleeplessness / insomnia | rs7170151 | 15 | 38846678 | T | C | 0.257 | 0.008 | 0.002 | 2.3e-06 | 462341 | 22.288 |
| Sleeplessness / insomnia | rs72924721 | 11 | 65585990 | T | C | 0.073 | 0.016 | 0.003 | 1.1e-08 | 462341 | 32.712 |
| Sleeplessness / insomnia | rs73064225 | 19 | 58974378 | A | G | 0.27 | 0.008 | 0.002 | 6.7e-07 | 462341 | 24.695 |
| Sleeplessness / insomnia | rs73230622 | 7 | 96491994 | C | T | 0.022 | 0.025 | 0.005 | 1.6e-06 | 462341 | 23.029 |
| Sleeplessness / insomnia | rs751727 | 6 | 33764158 | G | A | 0.805 | -0.009 | 0.002 | 2.5e-06 | 462341 | 22.147 |
| Sleeplessness / insomnia | rs7559077 | 2 | 3127643 | G | T | 0.518 | -0.007 | 0.001 | 2.3e-06 | 462341 | 22.289 |
| Sleeplessness / insomnia | rs7568599 | 2 | 53028060 | C | T | 0.466 | -0.007 | 0.002 | 7.4e-07 | 462341 | 24.503 |
| Sleeplessness / insomnia | rs75709417 | 3 | 87842436 | T | C | 0.121 | 0.012 | 0.002 | 6.3e-08 | 462341 | 29.283 |
| Sleeplessness / insomnia | rs7625896 | 3 | 44062561 | G | A | 0.347 | -0.008 | 0.002 | 1.7e-07 | 462341 | 27.334 |
| Sleeplessness / insomnia | rs76265753 | 1 | 161181134 | T | C | 0.036 | 0.021 | 0.004 | 1.3e-07 | 462341 | 27.936 |
| Sleeplessness / insomnia | rs7710039 | 5 | 80492967 | C | T | 0.279 | -0.008 | 0.002 | 2.2e-06 | 462341 | 22.382 |
| Sleeplessness / insomnia | rs7711696 | 5 | 135486536 | T | G | 0.305 | 0.011 | 0.002 | 4.1e-12 | 462341 | 48.065 |
| Sleeplessness / insomnia | rs776935 | 8 | 107747281 | G | A | 0.189 | -0.01 | 0.002 | 5.5e-07 | 462341 | 25.094 |
| Sleeplessness / insomnia | rs77748196 | 2 | 159302112 | T | C | 0.245 | -0.009 | 0.002 | 5.8e-07 | 462341 | 24.97 |
| Sleeplessness / insomnia | rs77887314 | 9 | 110353338 | A | C | 0.018 | -0.026 | 0.006 | 4e-06 | 462341 | 21.258 |
| Sleeplessness / insomnia | rs7817180 | 8 | 66206231 | T | C | 0.193 | 0.009 | 0.002 | 1.4e-06 | 462341 | 23.321 |
| Sleeplessness / insomnia | rs78887635 | 2 | 181215001 | C | T | 0.125 | -0.012 | 0.002 | 2.3e-07 | 462341 | 26.744 |
| Sleeplessness / insomnia | rs7904525 | 10 | 90529899 | T | C | 0.354 | 0.007 | 0.002 | 3.9e-06 | 462341 | 21.29 |
| Sleeplessness / insomnia | rs8029613 | 15 | 97123146 | G | T | 0.123 | -0.011 | 0.002 | 5.5e-07 | 462341 | 25.097 |
| Sleeplessness / insomnia | rs872369 | 15 | 70671550 | G | A | 0.438 | -0.008 | 0.002 | 2.1e-07 | 462341 | 26.92 |
| Sleeplessness / insomnia | rs948597 | 18 | 43893561 | G | A | 0.814 | 0.009 | 0.002 | 2.7e-06 | 462341 | 22.024 |
| Sleeplessness / insomnia | rs9516693 | 13 | 97081249 | G | A | 0.549 | 0.007 | 0.001 | 6.8e-07 | 462341 | 24.667 |
| Sleeplessness / insomnia | rs9570080 | 13 | 59832775 | C | T | 0.344 | -0.011 | 0.002 | 1.6e-11 | 462341 | 45.413 |
| Sleeplessness / insomnia | rs9576155 | 13 | 37600284 | A | G | 0.34 | 0.008 | 0.002 | 1.9e-07 | 462341 | 27.086 |
| Sleeplessness / insomnia | rs9610500 | 22 | 22221167 | G | A | 0.367 | -0.007 | 0.002 | 2.3e-06 | 462341 | 22.357 |
| Sleeplessness / insomnia | rs9611519 | 22 | 41613188 | T | C | 0.287 | 0.008 | 0.002 | 2.7e-06 | 462341 | 22.03 |
| Sleeplessness / insomnia | rs9649581 | 7 | 132526794 | A | G | 0.253 | 0.008 | 0.002 | 8.9e-07 | 462341 | 24.146 |
| Sleeplessness / insomnia | rs9770953 | 7 | 580682 | C | T | 0.675 | 0.008 | 0.002 | 2.7e-06 | 462341 | 22.03 |
| Sleeplessness / insomnia | rs9845387 | 3 | 116425935 | A | C | 0.04 | -0.022 | 0.004 | 7.1e-09 | 462341 | 33.494 |
| Sleeplessness / insomnia | rs9894577 | 17 | 43223292 | A | G | 0.318 | 0.013 | 0.002 | 1.3e-16 | 462341 | 68.382 |
| Sleeplessness / insomnia | rs9906181 | 17 | 21297686 | G | A | 0.688 | -0.009 | 0.002 | 2.4e-08 | 462341 | 31.18 |
| Bacon intake | rs116340177 | 6 | 147125077 | G | T | 0.047 | 0.05 | 0.01 | 7.39997e-07 | 64949 | 24.516 |
| Bacon intake | rs117334069 | 6 | 136980042 | T | C | 0.014 | 0.102 | 0.02 | 3.79997e-07 | 64949 | 25.815 |
| Bacon intake | rs141192377 | 17 | 26511740 | A | G | 0.012 | 0.098 | 0.02 | 1.09999e-06 | 64949 | 23.685 |
| Bacon intake | rs143523506 | 1 | 181798877 | G | T | 0.027 | 0.075 | 0.014 | 3.2e-08 | 64949 | 30.602 |
| Bacon intake | rs145150281 | 2 | 117576647 | C | T | 0.023 | 0.073 | 0.015 | 1.09999e-06 | 64949 | 23.686 |
| Bacon intake | rs6590783 | 11 | 134821821 | G | A | 0.448 | -0.024 | 0.004 | 2.19999e-08 | 64949 | 31.305 |
| Bacon intake | rs66626876 | 4 | 190101569 | G | T | 0.109 | 0.033 | 0.007 | 1.6e-06 | 64949 | 23.044 |
| Bacon intake | rs66799945 | 15 | 60567834 | A | G | 0.155 | -0.03 | 0.006 | 7.19996e-07 | 64949 | 24.557 |
| Bacon intake | rs6693446 | 1 | 21988976 | T | C | 0.113 | -0.032 | 0.007 | 3.89996e-06 | 64949 | 21.32 |
| Bacon intake | rs74871396 | 7 | 125406799 | G | T | 0.027 | 0.077 | 0.013 | 9.49992e-09 | 64949 | 32.95 |
| Bacon intake | rs8116059 | 20 | 39763534 | C | T | 0.288 | -0.023 | 0.005 | 2e-06 | 64949 | 22.642 |
| Beef intake | rs10123094 | 9 | 137096064 | A | G | 0.582 | -0.008 | 0.002 | 2.59998e-06 | 461053 | 22.112 |
| Beef intake | rs10247400 | 7 | 17583429 | G | T | 0.57 | -0.008 | 0.002 | 2.80001e-06 | 461053 | 21.979 |
| Beef intake | rs10283912 | 9 | 12285170 | T | C | 0.519 | 0.008 | 0.002 | 1.29999e-06 | 461053 | 23.467 |
| Beef intake | rs10789340 | 1 | 72940273 | G | A | 0.627 | -0.014 | 0.002 | 6.79986e-15 | 461053 | 60.652 |
| Beef intake | rs10959890 | 9 | 11526198 | C | T | 0.212 | -0.013 | 0.002 | 1.5e-09 | 461053 | 36.512 |
| Beef intake | rs1105388 | 1 | 205308591 | T | C | 0.3 | -0.011 | 0.002 | 1.29999e-09 | 461053 | 36.782 |
| Beef intake | rs11121250 | 1 | 8947587 | C | T | 0.749 | 0.009 | 0.002 | 3.59998e-06 | 461053 | 21.461 |
| Beef intake | rs112094422 | 11 | 95206468 | T | G | 0.032 | 0.025 | 0.005 | 3.09999e-07 | 461053 | 26.213 |
| Beef intake | rs112663712 | 19 | 47557075 | T | C | 0.043 | -0.02 | 0.004 | 3.79997e-06 | 461053 | 21.372 |
| Beef intake | rs115736207 | 3 | 81956915 | G | A | 0.023 | -0.029 | 0.006 | 8.99995e-07 | 461053 | 24.14 |
| Beef intake | rs11600756 | 11 | 126767481 | T | C | 0.158 | 0.011 | 0.002 | 1.7e-06 | 461053 | 22.862 |
| Beef intake | rs117117111 | 17 | 8025557 | T | C | 0.028 | -0.024 | 0.005 | 3.50002e-06 | 461053 | 21.515 |
| Beef intake | rs117340623 | 7 | 51686316 | A | G | 0.012 | 0.036 | 0.008 | 3.09999e-06 | 461053 | 21.781 |
| Beef intake | rs11749912 | 5 | 88065628 | G | A | 0.574 | -0.008 | 0.002 | 2.80001e-06 | 461053 | 21.924 |
| Beef intake | rs11878917 | 19 | 42588999 | A | G | 0.11 | 0.015 | 0.003 | 4.60002e-08 | 461053 | 29.867 |
| Beef intake | rs11904006 | 2 | 166942140 | T | C | 0.227 | 0.01 | 0.002 | 2.59998e-06 | 461053 | 22.08 |
| Beef intake | rs1191468 | 14 | 29502262 | A | G | 0.831 | -0.011 | 0.002 | 4.90004e-07 | 461053 | 25.291 |
| Beef intake | rs12113634 | 7 | 111996952 | C | T | 0.355 | 0.009 | 0.002 | 4.09996e-07 | 461053 | 25.624 |
| Beef intake | rs12257513 | 10 | 126249756 | G | A | 0.212 | 0.01 | 0.002 | 4.49997e-06 | 461053 | 21.06 |
| Beef intake | rs12652806 | 5 | 6473308 | C | T | 0.741 | -0.009 | 0.002 | 2.69998e-06 | 461053 | 21.985 |
| Beef intake | rs12670003 | 7 | 39083653 | A | G | 0.3 | 0.009 | 0.002 | 3.69999e-06 | 461053 | 21.429 |
| Beef intake | rs12829270 | 12 | 24025501 | A | G | 0.481 | -0.009 | 0.002 | 4.70002e-07 | 461053 | 25.397 |
| Beef intake | rs13145306 | 4 | 131039106 | T | C | 0.064 | 0.017 | 0.004 | 1.7e-06 | 461053 | 22.866 |
| Beef intake | rs132901 | 22 | 41797547 | T | C | 0.788 | 0.014 | 0.002 | 2.90001e-11 | 461053 | 44.271 |
| Beef intake | rs1343431 | 1 | 49433003 | C | A | 0.648 | -0.008 | 0.002 | 4.90004e-06 | 461053 | 20.874 |
| Beef intake | rs139552257 | 2 | 43536687 | T | C | 0.014 | -0.04 | 0.008 | 1.09999e-07 | 461053 | 28.171 |
| Beef intake | rs140748046 | 13 | 20006754 | C | T | 0.026 | -0.026 | 0.006 | 3.29997e-06 | 461053 | 21.616 |
| Beef intake | rs1421085 | 16 | 53800954 | C | T | 0.403 | -0.012 | 0.002 | 3.50026e-12 | 461053 | 48.391 |
| Beef intake | rs144160011 | 8 | 20785488 | G | A | 0.022 | -0.028 | 0.006 | 2.80001e-06 | 461053 | 21.916 |
| Beef intake | rs1442572 | 14 | 85668179 | A | G | 0.474 | -0.009 | 0.002 | 5.99998e-08 | 461053 | 29.355 |
| Beef intake | rs1555302 | 20 | 43430968 | C | A | 0.312 | -0.009 | 0.002 | 9.59997e-07 | 461053 | 24.003 |
| Beef intake | rs17274957 | 7 | 36324938 | A | G | 0.407 | -0.009 | 0.002 | 9.49992e-07 | 461053 | 24.029 |
| Beef intake | rs17413652 | 1 | 46767176 | A | G | 0.133 | -0.012 | 0.003 | 1.40001e-06 | 461053 | 23.328 |
| Beef intake | rs17501421 | 13 | 66919913 | A | G | 0.068 | -0.017 | 0.003 | 9.69996e-07 | 461053 | 23.986 |
| Beef intake | rs17624102 | 18 | 24570647 | T | C | 0.041 | 0.021 | 0.004 | 1.2e-06 | 461053 | 23.544 |
| Beef intake | rs187160427 | 10 | 114238724 | A | G | 0.058 | 0.019 | 0.004 | 3.29997e-07 | 461053 | 26.059 |
| Beef intake | rs2107601 | 7 | 88511233 | T | C | 0.111 | -0.013 | 0.003 | 9.49992e-07 | 461053 | 24.021 |
| Beef intake | rs2541303 | 12 | 22445690 | G | A | 0.259 | -0.01 | 0.002 | 2.1e-07 | 461053 | 26.945 |
| Beef intake | rs2567950 | 4 | 171877419 | G | T | 0.577 | 0.009 | 0.002 | 8.19993e-08 | 461053 | 28.754 |
| Beef intake | rs267733 | 1 | 150958836 | G | A | 0.161 | 0.011 | 0.002 | 1.2e-06 | 461053 | 23.571 |
| Beef intake | rs28570979 | 4 | 79429693 | G | A | 0.258 | 0.009 | 0.002 | 4.39997e-06 | 461053 | 21.083 |
| Beef intake | rs2965199 | 19 | 19475088 | A | G | 0.651 | -0.009 | 0.002 | 2.1e-06 | 461053 | 22.463 |
| Beef intake | rs34465895 | 11 | 75530557 | G | A | 0.039 | -0.022 | 0.005 | 1.89998e-06 | 461053 | 22.719 |
| Beef intake | rs34953822 | 2 | 205113767 | A | C | 0.001 | 0.121 | 0.026 | 2.80001e-06 | 461053 | 21.95 |
| Beef intake | rs34962644 | 12 | 130163903 | G | A | 0.047 | -0.022 | 0.004 | 4.09996e-07 | 461053 | 25.656 |
| Beef intake | rs35556085 | 6 | 141820659 | A | G | 0.133 | 0.012 | 0.003 | 3.89996e-06 | 461053 | 21.304 |
| Beef intake | rs386231 | 1 | 162325823 | T | C | 0.723 | 0.009 | 0.002 | 4.30002e-06 | 461053 | 21.124 |
| Beef intake | rs4131337 | 16 | 7974635 | G | A | 0.223 | 0.01 | 0.002 | 5.89997e-07 | 461053 | 24.938 |
| Beef intake | rs41476948 | 14 | 78788749 | A | G | 0.396 | 0.008 | 0.002 | 2.5e-06 | 461053 | 22.146 |
| Beef intake | rs429358 | 19 | 45411941 | C | T | 0.154 | -0.015 | 0.002 | 3.59998e-10 | 461053 | 39.319 |
| Beef intake | rs4585149 | 3 | 157493952 | C | T | 0.82 | -0.011 | 0.002 | 1.40001e-06 | 461053 | 23.294 |
| Beef intake | rs4676964 | 3 | 71034748 | T | C | 0.511 | 0.013 | 0.002 | 9.60064e-15 | 461053 | 59.967 |
| Beef intake | rs4714375 | 6 | 40724131 | G | T | 0.316 | -0.009 | 0.002 | 1.7e-06 | 461053 | 22.904 |
| Beef intake | rs4759074 | 12 | 54664097 | T | C | 0.41 | 0.009 | 0.002 | 4.20001e-07 | 461053 | 25.582 |
| Beef intake | rs4863708 | 4 | 140873893 | A | C | 0.293 | 0.009 | 0.002 | 4.20001e-06 | 461053 | 21.192 |
| Beef intake | rs61845699 | 10 | 43759795 | A | G | 0.496 | 0.008 | 0.002 | 2.30001e-06 | 461053 | 22.33 |
| Beef intake | rs62015188 | 15 | 68056480 | A | C | 0.018 | 0.033 | 0.006 | 2.99999e-07 | 461053 | 26.259 |
| Beef intake | rs62169335 | 2 | 147946069 | T | C | 0.543 | -0.01 | 0.002 | 2.39999e-08 | 461053 | 31.16 |
| Beef intake | rs645040 | 3 | 135926622 | T | G | 0.773 | 0.01 | 0.002 | 3.09999e-07 | 461053 | 26.201 |
| Beef intake | rs6669189 | 1 | 75011358 | T | C | 0.402 | -0.008 | 0.002 | 4.30002e-06 | 461053 | 21.143 |
| Beef intake | rs6739040 | 2 | 217951726 | G | A | 0.072 | 0.017 | 0.003 | 2.69998e-07 | 461053 | 26.438 |
| Beef intake | rs67616875 | 15 | 101143531 | A | C | 0.203 | -0.01 | 0.002 | 2.90001e-06 | 461053 | 21.872 |
| Beef intake | rs6791758 | 3 | 171323723 | C | T | 0.448 | 0.008 | 0.002 | 1.40001e-06 | 461053 | 23.219 |
| Beef intake | rs6851529 | 4 | 187932470 | T | C | 0.577 | 0.008 | 0.002 | 4.70002e-06 | 461053 | 20.97 |
| Beef intake | rs7018123 | 8 | 93667572 | G | A | 0.299 | -0.009 | 0.002 | 4.30002e-06 | 461053 | 21.135 |
| Beef intake | rs71559014 | 6 | 27122444 | G | A | 0.086 | 0.015 | 0.003 | 1e-06 | 461053 | 23.847 |
| Beef intake | rs7174015 | 15 | 50717068 | A | G | 0.405 | 0.008 | 0.002 | 1.79999e-06 | 461053 | 22.775 |
| Beef intake | rs7238302 | 18 | 65623877 | T | G | 0.484 | 0.008 | 0.002 | 4.09996e-06 | 461053 | 21.224 |
| Beef intake | rs72779821 | 16 | 9743591 | G | T | 0.198 | 0.01 | 0.002 | 1.29999e-06 | 461053 | 23.472 |
| Beef intake | rs74818437 | 5 | 124070735 | A | G | 0.046 | 0.02 | 0.004 | 1.7e-06 | 461053 | 22.866 |
| Beef intake | rs75191738 | 6 | 130348257 | T | C | 0.152 | 0.011 | 0.002 | 1.89998e-06 | 461053 | 22.655 |
| Beef intake | rs7597864 | 2 | 65403970 | T | C | 0.301 | 0.009 | 0.002 | 3.09999e-06 | 461053 | 21.758 |
| Beef intake | rs7601686 | 2 | 206958722 | T | C | 0.387 | -0.009 | 0.002 | 6.90001e-07 | 461053 | 24.637 |
| Beef intake | rs77862656 | 17 | 33669329 | T | C | 0.089 | 0.015 | 0.003 | 4.70002e-07 | 461053 | 25.368 |
| Beef intake | rs7791463 | 7 | 97819047 | A | G | 0.535 | 0.01 | 0.002 | 2.39999e-08 | 461053 | 31.157 |
| Beef intake | rs784251 | 18 | 53412903 | T | C | 0.478 | -0.01 | 0.002 | 1.7e-09 | 461053 | 36.277 |
| Beef intake | rs7902949 | 10 | 53932566 | T | C | 0.751 | -0.01 | 0.002 | 2e-06 | 461053 | 22.583 |
| Beef intake | rs7974084 | 12 | 39654700 | C | A | 0.127 | -0.012 | 0.003 | 3.2e-06 | 461053 | 21.673 |
| Beef intake | rs79809011 | 8 | 10126532 | A | G | 0.029 | -0.028 | 0.005 | 3.40001e-08 | 461053 | 30.451 |
| Beef intake | rs80321956 | 13 | 80571300 | A | G | 0.013 | 0.036 | 0.007 | 1.09999e-06 | 461053 | 23.681 |
| Beef intake | rs803370 | 1 | 44677803 | C | T | 0.703 | 0.01 | 0.002 | 3.40001e-07 | 461053 | 25.988 |
| Beef intake | rs897509 | 2 | 33150932 | G | A | 0.469 | -0.009 | 0.002 | 2.39999e-07 | 461053 | 26.652 |
| Beef intake | rs9319848 | 18 | 70304838 | A | G | 0.775 | -0.009 | 0.002 | 4.70002e-06 | 461053 | 20.949 |
| Beef intake | rs937813 | 2 | 28301540 | C | T | 0.11 | 0.015 | 0.003 | 9.09997e-08 | 461053 | 28.557 |
| Beef intake | rs952645 | 13 | 103989730 | C | T | 0.531 | 0.008 | 0.002 | 4.20001e-06 | 461053 | 21.182 |
| Beef intake | rs9615805 | 22 | 48613029 | A | G | 0.103 | 0.014 | 0.003 | 1.5e-06 | 461053 | 23.089 |
| Beef intake | rs9861346 | 3 | 117508846 | T | C | 0.854 | -0.012 | 0.002 | 3.59998e-07 | 461053 | 25.918 |
| Lamb/mutton intake | rs10098073 | 8 | 143309504 | A | C | 0.473 | -0.008 | 0.001 | 1e-07 | 460006 | 28.341 |
| Lamb/mutton intake | rs10118916 | 9 | 122672140 | C | A | 0.595 | -0.007 | 0.001 | 1.09999e-06 | 460006 | 23.82 |
| Lamb/mutton intake | rs10180960 | 2 | 621115 | T | C | 0.347 | -0.008 | 0.002 | 1.79999e-07 | 460006 | 27.225 |
| Lamb/mutton intake | rs10852981 | 17 | 54416747 | T | G | 0.526 | -0.007 | 0.001 | 2.90001e-06 | 460006 | 21.863 |
| Lamb/mutton intake | rs10883567 | 10 | 102755779 | C | T | 0.489 | -0.007 | 0.001 | 7.90005e-07 | 460006 | 24.389 |
| Lamb/mutton intake | rs10905778 | 10 | 10699419 | A | G | 0.444 | 0.007 | 0.001 | 4e-07 | 460006 | 25.698 |
| Lamb/mutton intake | rs10932201 | 2 | 208426257 | A | G | 0.461 | 0.007 | 0.001 | 8.60003e-07 | 460006 | 24.209 |
| Lamb/mutton intake | rs11019656 | 11 | 91575773 | G | T | 0.247 | -0.009 | 0.002 | 2.39999e-07 | 460006 | 26.708 |
| Lamb/mutton intake | rs11090045 | 22 | 41753603 | A | G | 0.307 | -0.011 | 0.002 | 2.99985e-11 | 460006 | 44.159 |
| Lamb/mutton intake | rs11100930 | 4 | 147591808 | A | G | 0.874 | 0.011 | 0.002 | 3.89996e-07 | 460006 | 25.728 |
| Lamb/mutton intake | rs114210747 | 5 | 144214142 | T | C | 0.084 | 0.012 | 0.003 | 3.2e-06 | 460006 | 21.684 |
| Lamb/mutton intake | rs115145470 | 1 | 95744298 | A | G | 0.03 | 0.021 | 0.004 | 9.69996e-07 | 460006 | 23.979 |
| Lamb/mutton intake | rs115687569 | 4 | 172402709 | A | G | 0.045 | 0.018 | 0.004 | 8.09991e-07 | 460006 | 24.338 |
| Lamb/mutton intake | rs11672035 | 19 | 3352401 | T | C | 0.211 | -0.009 | 0.002 | 4.79999e-07 | 460006 | 25.328 |
| Lamb/mutton intake | rs11673714 | 2 | 176130707 | C | T | 0.089 | -0.012 | 0.003 | 2.59998e-06 | 460006 | 22.077 |
| Lamb/mutton intake | rs11743441 | 5 | 88065637 | T | G | 0.574 | -0.009 | 0.001 | 2.69998e-09 | 460006 | 35.408 |
| Lamb/mutton intake | rs11756299 | 6 | 54951016 | G | A | 0.427 | 0.008 | 0.001 | 2.99999e-07 | 460006 | 26.249 |
| Lamb/mutton intake | rs11772832 | 7 | 135073047 | C | T | 0.4 | -0.007 | 0.001 | 8.70001e-07 | 460006 | 24.202 |
| Lamb/mutton intake | rs117817642 | 22 | 36426920 | A | G | 0.02 | -0.025 | 0.005 | 2.39999e-06 | 460006 | 22.229 |
| Lamb/mutton intake | rs1193333 | 7 | 127609301 | G | A | 0.643 | 0.007 | 0.002 | 2.1e-06 | 460006 | 22.493 |
| Lamb/mutton intake | rs12494242 | 3 | 85413180 | C | T | 0.319 | 0.008 | 0.002 | 1.29999e-06 | 460006 | 23.453 |
| Lamb/mutton intake | rs12595749 | 15 | 79432359 | G | A | 0.423 | 0.007 | 0.001 | 4.79999e-06 | 460006 | 20.913 |
| Lamb/mutton intake | rs12634740 | 3 | 175671450 | G | T | 0.252 | -0.01 | 0.002 | 2.80001e-09 | 460006 | 35.324 |
| Lamb/mutton intake | rs12908716 | 15 | 33520165 | G | T | 0.533 | -0.007 | 0.001 | 3.29997e-06 | 460006 | 21.606 |
| Lamb/mutton intake | rs12935033 | 16 | 89543188 | T | C | 0.38 | -0.007 | 0.002 | 4.20001e-06 | 460006 | 21.175 |
| Lamb/mutton intake | rs136548 | 22 | 27253353 | T | C | 0.377 | 0.01 | 0.002 | 2.90001e-10 | 460006 | 39.732 |
| Lamb/mutton intake | rs137969241 | 1 | 49662687 | T | G | 0.011 | -0.035 | 0.008 | 2.90001e-06 | 460006 | 21.876 |
| Lamb/mutton intake | rs139012851 | 9 | 83446447 | G | A | 0.008 | 0.04 | 0.008 | 1.29999e-06 | 460006 | 23.386 |
| Lamb/mutton intake | rs139237013 | 2 | 56662691 | A | G | 0.058 | 0.019 | 0.003 | 1.79999e-09 | 460006 | 36.224 |
| Lamb/mutton intake | rs141934869 | 8 | 27775477 | T | G | 0.01 | -0.035 | 0.008 | 2.69998e-06 | 460006 | 22.035 |
| Lamb/mutton intake | rs142325173 | 12 | 18822872 | A | G | 0.017 | -0.028 | 0.006 | 1.7e-06 | 460006 | 22.932 |
| Lamb/mutton intake | rs146908007 | 1 | 98559491 | T | C | 0.009 | -0.038 | 0.008 | 3.50002e-06 | 460006 | 21.515 |
| Lamb/mutton intake | rs1474864 | 1 | 187033589 | T | C | 0.044 | 0.017 | 0.004 | 1.7e-06 | 460006 | 22.959 |
| Lamb/mutton intake | rs17107024 | 14 | 69961658 | C | T | 0.129 | -0.01 | 0.002 | 1.5e-06 | 460006 | 23.126 |
| Lamb/mutton intake | rs1711171 | 3 | 136020541 | C | T | 0.75 | 0.009 | 0.002 | 1.89998e-07 | 460006 | 27.091 |
| Lamb/mutton intake | rs17235152 | 13 | 95906121 | C | T | 0.151 | -0.01 | 0.002 | 6.29999e-07 | 460006 | 24.808 |
| Lamb/mutton intake | rs17270057 | 19 | 42670527 | C | T | 0.113 | 0.013 | 0.002 | 4.30002e-08 | 460006 | 30.031 |
| Lamb/mutton intake | rs17547360 | 12 | 30169738 | C | T | 0.248 | 0.009 | 0.002 | 1.09999e-07 | 460006 | 28.135 |
| Lamb/mutton intake | rs17594338 | 5 | 93180544 | A | G | 0.333 | -0.007 | 0.002 | 2.30001e-06 | 460006 | 22.367 |
| Lamb/mutton intake | rs1958801 | 14 | 98872110 | G | A | 0.288 | -0.009 | 0.002 | 3.2e-08 | 460006 | 30.575 |
| Lamb/mutton intake | rs2018466 | 5 | 158162106 | A | G | 0.157 | -0.009 | 0.002 | 3.29997e-06 | 460006 | 21.654 |
| Lamb/mutton intake | rs2028935 | 5 | 19607413 | C | A | 0.485 | -0.007 | 0.001 | 4.60002e-07 | 460006 | 25.438 |
| Lamb/mutton intake | rs2101975 | 4 | 106216667 | G | A | 0.432 | -0.007 | 0.001 | 1.29999e-06 | 460006 | 23.48 |
| Lamb/mutton intake | rs2116439 | 2 | 55001405 | G | A | 0.339 | -0.008 | 0.002 | 1.09999e-06 | 460006 | 23.792 |
| Lamb/mutton intake | rs2135928 | 4 | 130934004 | T | G | 0.732 | -0.008 | 0.002 | 1.5e-06 | 460006 | 23.185 |
| Lamb/mutton intake | rs2222760 | 2 | 151345113 | A | G | 0.281 | -0.009 | 0.002 | 2.80001e-08 | 460006 | 30.844 |
| Lamb/mutton intake | rs224064 | 10 | 64504212 | C | T | 0.604 | 0.008 | 0.001 | 4.39997e-07 | 460006 | 25.525 |
| Lamb/mutton intake | rs2396885 | 6 | 48782953 | G | A | 0.839 | -0.01 | 0.002 | 3.50002e-07 | 460006 | 25.964 |
| Lamb/mutton intake | rs2439945 | 17 | 5124462 | T | C | 0.194 | 0.009 | 0.002 | 7.90005e-07 | 460006 | 24.389 |
| Lamb/mutton intake | rs2513324 | 11 | 112491227 | C | A | 0.267 | 0.009 | 0.002 | 5.69994e-08 | 460006 | 29.473 |
| Lamb/mutton intake | rs2678900 | 2 | 58177683 | G | T | 0.428 | 0.01 | 0.001 | 9.8992e-12 | 460006 | 46.353 |
| Lamb/mutton intake | rs2696532 | 17 | 44355602 | G | A | 0.22 | -0.009 | 0.002 | 2e-07 | 460006 | 27.023 |
| Lamb/mutton intake | rs270026 | 6 | 128172254 | G | A | 0.415 | -0.007 | 0.001 | 3.29997e-06 | 460006 | 21.629 |
| Lamb/mutton intake | rs2726033 | 16 | 28338039 | G | A | 0.422 | -0.009 | 0.001 | 1.5e-10 | 460006 | 41.09 |
| Lamb/mutton intake | rs276453 | 9 | 15573753 | C | A | 0.488 | -0.014 | 0.001 | 2.90001e-22 | 460006 | 94.154 |
| Lamb/mutton intake | rs2926119 | 16 | 64293179 | A | C | 0.569 | 0.008 | 0.001 | 4.39997e-08 | 460006 | 29.964 |
| Lamb/mutton intake | rs2966819 | 5 | 107541291 | G | A | 0.321 | 0.008 | 0.002 | 2.80001e-07 | 460006 | 26.411 |
| Lamb/mutton intake | rs3105056 | 13 | 55946152 | C | T | 0.733 | -0.012 | 0.002 | 1.80011e-12 | 460006 | 49.718 |
| Lamb/mutton intake | rs34503203 | 5 | 35335006 | G | A | 0.389 | 0.007 | 0.001 | 2e-06 | 460006 | 22.561 |
| Lamb/mutton intake | rs3737627 | 1 | 1722828 | A | G | 0.271 | -0.008 | 0.002 | 3.29997e-06 | 460006 | 21.627 |
| Lamb/mutton intake | rs3828661 | 5 | 127755996 | C | A | 0.221 | -0.008 | 0.002 | 3.09999e-06 | 460006 | 21.754 |
| Lamb/mutton intake | rs3909726 | 11 | 126587391 | A | G | 0.836 | 0.014 | 0.002 | 1.80011e-12 | 460006 | 49.715 |
| Lamb/mutton intake | rs3964074 | 16 | 74166991 | C | T | 0.547 | -0.008 | 0.001 | 3.2e-08 | 460006 | 30.581 |
| Lamb/mutton intake | rs416769 | 5 | 102114414 | G | A | 0.421 | 0.008 | 0.001 | 2e-07 | 460006 | 27.028 |
| Lamb/mutton intake | rs4272399 | 8 | 4836291 | A | C | 0.321 | -0.009 | 0.002 | 4.49997e-09 | 460006 | 34.406 |
| Lamb/mutton intake | rs429358 | 19 | 45411941 | C | T | 0.154 | -0.018 | 0.002 | 2.70023e-19 | 460006 | 80.611 |
| Lamb/mutton intake | rs4315028 | 11 | 47099125 | C | A | 0.232 | 0.008 | 0.002 | 2.69998e-06 | 460006 | 21.993 |
| Lamb/mutton intake | rs4422110 | 2 | 146114898 | T | C | 0.535 | -0.008 | 0.001 | 2.59998e-07 | 460006 | 26.56 |
| Lamb/mutton intake | rs4444956 | 5 | 178325515 | G | A | 0.49 | -0.008 | 0.001 | 1.2e-07 | 460006 | 28.047 |
| Lamb/mutton intake | rs4449668 | 6 | 30211894 | T | G | 0.159 | -0.01 | 0.002 | 9.49992e-07 | 460006 | 24.022 |
| Lamb/mutton intake | rs4486004 | 6 | 26167710 | T | G | 0.221 | -0.009 | 0.002 | 8.30004e-07 | 460006 | 24.285 |
| Lamb/mutton intake | rs4676964 | 3 | 71034748 | T | C | 0.511 | 0.008 | 0.001 | 1.40001e-07 | 460006 | 27.679 |
| Lamb/mutton intake | rs4759074 | 12 | 54664097 | T | C | 0.41 | 0.007 | 0.001 | 2.59998e-06 | 460006 | 22.099 |
| Lamb/mutton intake | rs4861436 | 4 | 181254500 | C | A | 0.61 | -0.007 | 0.002 | 6.1e-07 | 460006 | 24.872 |
| Lamb/mutton intake | rs55813438 | 16 | 5694999 | A | G | 0.763 | -0.011 | 0.002 | 4.70002e-11 | 460006 | 43.3 |
| Lamb/mutton intake | rs56394517 | 1 | 243582753 | G | A | 0.096 | -0.014 | 0.002 | 3.2e-08 | 460006 | 30.573 |
| Lamb/mutton intake | rs61836106 | 10 | 2639003 | A | G | 0.11 | -0.011 | 0.002 | 1.09999e-06 | 460006 | 23.728 |
| Lamb/mutton intake | rs62106258 | 2 | 417167 | C | T | 0.049 | 0.022 | 0.003 | 2e-10 | 460006 | 40.437 |
| Lamb/mutton intake | rs62169335 | 2 | 147946069 | T | C | 0.543 | -0.007 | 0.001 | 5.69994e-07 | 460006 | 25.004 |
| Lamb/mutton intake | rs62243496 | 3 | 62518227 | A | C | 0.215 | -0.009 | 0.002 | 5.89997e-07 | 460006 | 24.96 |
| Lamb/mutton intake | rs62266239 | 3 | 116750431 | T | C | 0.136 | 0.011 | 0.002 | 2.59998e-07 | 460006 | 26.536 |
| Lamb/mutton intake | rs62398404 | 6 | 37669616 | T | C | 0.127 | 0.013 | 0.002 | 4e-09 | 460006 | 34.642 |
| Lamb/mutton intake | rs6476606 | 9 | 37005561 | G | A | 0.629 | 0.007 | 0.002 | 3.40001e-06 | 460006 | 21.568 |
| Lamb/mutton intake | rs6500598 | 16 | 4470964 | T | G | 0.748 | -0.008 | 0.002 | 6.4e-07 | 460006 | 24.781 |
| Lamb/mutton intake | rs660880 | 1 | 6866978 | A | G | 0.513 | -0.009 | 0.001 | 6.80002e-10 | 460006 | 38.064 |
| Lamb/mutton intake | rs673696 | 11 | 31097262 | T | C | 0.081 | 0.016 | 0.003 | 3.69999e-09 | 460006 | 34.796 |
| Lamb/mutton intake | rs6829572 | 4 | 93984155 | A | G | 0.457 | 0.008 | 0.001 | 1.2e-08 | 460006 | 32.481 |
| Lamb/mutton intake | rs6960970 | 7 | 14207740 | C | T | 0.804 | -0.009 | 0.002 | 2.59998e-06 | 460006 | 22.103 |
| Lamb/mutton intake | rs7039466 | 9 | 2095330 | T | C | 0.112 | 0.011 | 0.002 | 4.20001e-06 | 460006 | 21.187 |
| Lamb/mutton intake | rs7183481 | 15 | 97179747 | A | G | 0.1 | 0.011 | 0.002 | 2.99999e-06 | 460006 | 21.828 |
| Lamb/mutton intake | rs7199285 | 16 | 19980931 | T | C | 0.184 | -0.009 | 0.002 | 2e-06 | 460006 | 22.609 |
| Lamb/mutton intake | rs7208329 | 17 | 33128801 | T | C | 0.027 | 0.022 | 0.005 | 1.79999e-06 | 460006 | 22.763 |
| Lamb/mutton intake | rs72637362 | 17 | 16578053 | C | T | 0.109 | -0.012 | 0.002 | 5.30005e-07 | 460006 | 25.148 |
| Lamb/mutton intake | rs72794909 | 16 | 76210598 | C | T | 0.039 | -0.02 | 0.004 | 1.6e-07 | 460006 | 27.409 |
| Lamb/mutton intake | rs73231956 | 12 | 123791649 | C | T | 0.138 | 0.011 | 0.002 | 1.89998e-07 | 460006 | 27.161 |
| Lamb/mutton intake | rs74032724 | 16 | 78719209 | G | A | 0.026 | 0.022 | 0.005 | 2.19999e-06 | 460006 | 22.426 |
| Lamb/mutton intake | rs7447465 | 5 | 164566362 | C | T | 0.619 | 0.01 | 0.002 | 2e-10 | 460006 | 40.474 |
| Lamb/mutton intake | rs74697766 | 4 | 105732919 | C | T | 0.083 | 0.013 | 0.003 | 4.60002e-07 | 460006 | 25.416 |
| Lamb/mutton intake | rs75188715 | 7 | 49859403 | A | G | 0.043 | 0.017 | 0.004 | 1.89998e-06 | 460006 | 22.672 |
| Lamb/mutton intake | rs7519530 | 1 | 25779236 | G | T | 0.558 | 0.007 | 0.001 | 3.89996e-06 | 460006 | 21.319 |
| Lamb/mutton intake | rs7649005 | 3 | 95229880 | C | T | 0.43 | -0.007 | 0.001 | 1.40001e-06 | 460006 | 23.236 |
| Lamb/mutton intake | rs77431173 | 7 | 14742864 | C | T | 0.031 | 0.022 | 0.004 | 2.80001e-07 | 460006 | 26.365 |
| Lamb/mutton intake | rs77691530 | 13 | 93616413 | T | C | 0.05 | -0.016 | 0.003 | 3.69999e-06 | 460006 | 21.396 |
| Lamb/mutton intake | rs78621251 | 18 | 63348042 | G | A | 0.035 | 0.019 | 0.004 | 1e-06 | 460006 | 23.917 |
| Lamb/mutton intake | rs8073904 | 17 | 79095144 | A | G | 0.867 | 0.011 | 0.002 | 1.79999e-07 | 460006 | 27.189 |
| Lamb/mutton intake | rs8107139 | 19 | 3462045 | C | T | 0.405 | 0.007 | 0.002 | 3.79997e-06 | 460006 | 21.378 |
| Lamb/mutton intake | rs9321151 | 6 | 129406577 | C | T | 0.146 | 0.01 | 0.002 | 3.69999e-06 | 460006 | 21.42 |
| Lamb/mutton intake | rs942038 | 13 | 112682875 | G | A | 0.7 | 0.008 | 0.002 | 1.09999e-06 | 460006 | 23.724 |
| Lamb/mutton intake | rs9545596 | 13 | 81680792 | T | C | 0.543 | 0.007 | 0.001 | 1.5e-06 | 460006 | 23.127 |
| Pork intake | rs10137783 | 14 | 97886219 | C | T | 0.01 | 0.038 | 0.007 | 4.39997e-07 | 460162 | 25.529 |
| Pork intake | rs10146532 | 14 | 33376513 | C | T | 0.221 | 0.009 | 0.002 | 9.59997e-07 | 460162 | 24.016 |
| Pork intake | rs1051921 | 7 | 73007943 | A | G | 0.197 | -0.01 | 0.002 | 9.40005e-08 | 460162 | 28.502 |
| Pork intake | rs10884893 | 10 | 111473404 | A | G | 0.497 | -0.007 | 0.001 | 1.79999e-06 | 460162 | 22.792 |
| Pork intake | rs10896701 | 11 | 57873135 | C | T | 0.648 | 0.007 | 0.002 | 1.5e-06 | 460162 | 23.092 |
| Pork intake | rs10931858 | 2 | 200096296 | G | A | 0.404 | -0.008 | 0.002 | 4.30002e-07 | 460162 | 25.533 |
| Pork intake | rs10972033 | 9 | 34269732 | T | G | 0.456 | 0.009 | 0.001 | 1.29999e-09 | 460162 | 36.853 |
| Pork intake | rs10988713 | 9 | 101884026 | T | C | 0.014 | -0.033 | 0.006 | 1.40001e-07 | 460162 | 27.79 |
| Pork intake | rs11031113 | 11 | 30515957 | A | G | 0.366 | -0.007 | 0.002 | 1.6e-06 | 460162 | 22.985 |
| Pork intake | rs1105714 | 15 | 83516968 | T | C | 0.136 | 0.01 | 0.002 | 2.59998e-06 | 460162 | 22.076 |
| Pork intake | rs11209948 | 1 | 72811904 | T | G | 0.604 | -0.008 | 0.002 | 1.09999e-07 | 460162 | 28.164 |
| Pork intake | rs11211124 | 1 | 45962409 | C | T | 0.231 | -0.01 | 0.002 | 1.40001e-08 | 460162 | 32.195 |
| Pork intake | rs115037803 | 2 | 34261988 | G | A | 0.022 | -0.025 | 0.005 | 1.40001e-06 | 460162 | 23.313 |
| Pork intake | rs11606837 | 11 | 115042837 | T | C | 0.528 | 0.008 | 0.001 | 1.5e-07 | 460162 | 27.536 |
| Pork intake | rs11635385 | 15 | 38662204 | A | G | 0.749 | 0.008 | 0.002 | 4.39997e-06 | 460162 | 21.071 |
| Pork intake | rs11787216 | 8 | 142615222 | T | C | 0.369 | 0.007 | 0.002 | 2.59998e-06 | 460162 | 22.077 |
| Pork intake | rs12097622 | 1 | 225028786 | G | A | 0.074 | 0.013 | 0.003 | 4.79999e-06 | 460162 | 20.926 |
| Pork intake | rs12516824 | 5 | 123972448 | T | C | 0.391 | -0.008 | 0.002 | 2.59998e-07 | 460162 | 26.547 |
| Pork intake | rs12932179 | 16 | 9072085 | G | A | 0.567 | 0.007 | 0.001 | 1.2e-06 | 460162 | 23.567 |
| Pork intake | rs13198572 | 6 | 27633328 | T | C | 0.105 | 0.011 | 0.002 | 2.5e-06 | 460162 | 22.196 |
| Pork intake | rs1323058 | 4 | 20287424 | G | A | 0.57 | -0.007 | 0.001 | 7.00003e-07 | 460162 | 24.603 |
| Pork intake | rs13262595 | 8 | 143316970 | G | A | 0.561 | 0.008 | 0.001 | 5.80003e-08 | 460162 | 29.415 |
| Pork intake | rs13335365 | 16 | 51684006 | G | A | 0.056 | -0.015 | 0.003 | 3.40001e-06 | 460162 | 21.564 |
| Pork intake | rs1355171 | 9 | 15681694 | A | C | 0.489 | -0.011 | 0.001 | 1e-13 | 460162 | 55.296 |
| Pork intake | rs138951160 | 5 | 93312271 | A | G | 0.011 | -0.034 | 0.007 | 3.89996e-06 | 460162 | 21.336 |
| Pork intake | rs1451077 | 2 | 147901207 | A | G | 0.584 | -0.008 | 0.001 | 8.9e-08 | 460162 | 28.593 |
| Pork intake | rs145789739 | 14 | 48600816 | T | C | 0.017 | 0.03 | 0.006 | 5e-07 | 460162 | 25.259 |
| Pork intake | rs147597513 | 5 | 126141761 | T | C | 0.011 | 0.033 | 0.007 | 2.5e-06 | 460162 | 22.15 |
| Pork intake | rs149747746 | 20 | 23674859 | T | C | 0.02 | -0.025 | 0.005 | 1.7e-06 | 460162 | 22.9 |
| Pork intake | rs1523138 | 4 | 31879574 | T | C | 0.2 | -0.009 | 0.002 | 1.7e-06 | 460162 | 22.886 |
| Pork intake | rs1590402 | 9 | 13680919 | T | C | 0.585 | 0.008 | 0.001 | 5e-07 | 460162 | 25.268 |
| Pork intake | rs17446175 | 13 | 107935480 | G | A | 0.503 | -0.007 | 0.001 | 3.79997e-06 | 460162 | 21.374 |
| Pork intake | rs1872885 | 11 | 104356788 | C | T | 0.549 | 0.007 | 0.001 | 7.49998e-07 | 460162 | 24.473 |
| Pork intake | rs1951289 | 14 | 29840613 | T | C | 0.634 | -0.007 | 0.002 | 3.50002e-06 | 460162 | 21.518 |
| Pork intake | rs2049997 | 1 | 210031138 | G | T | 0.597 | 0.007 | 0.002 | 2.19999e-06 | 460162 | 22.443 |
| Pork intake | rs2387807 | 12 | 38446861 | T | C | 0.078 | -0.015 | 0.003 | 4.09996e-08 | 460162 | 30.124 |
| Pork intake | rs2555050 | 7 | 135977444 | A | G | 0.42 | -0.007 | 0.001 | 2.80001e-06 | 460162 | 21.969 |
| Pork intake | rs2863724 | 11 | 46200912 | A | G | 0.492 | -0.007 | 0.001 | 4.49997e-07 | 460162 | 25.447 |
| Pork intake | rs34323725 | 14 | 81743718 | T | C | 0.099 | 0.012 | 0.002 | 1.5e-06 | 460162 | 23.104 |
| Pork intake | rs35934080 | 7 | 96707653 | A | G | 0.274 | -0.009 | 0.002 | 2e-07 | 460162 | 27.019 |
| Pork intake | rs36124222 | 18 | 74196202 | C | T | 0.433 | 0.008 | 0.002 | 2.1e-08 | 460162 | 31.395 |
| Pork intake | rs3964074 | 16 | 74166991 | C | T | 0.547 | -0.009 | 0.001 | 1.6e-09 | 460162 | 36.406 |
| Pork intake | rs4146837 | 15 | 93446869 | T | C | 0.456 | 0.009 | 0.001 | 4e-09 | 460162 | 34.615 |
| Pork intake | rs4605770 | 5 | 77645358 | A | G | 0.515 | -0.007 | 0.001 | 5.39995e-07 | 460162 | 25.107 |
| Pork intake | rs4658626 | 1 | 244509106 | C | A | 0.544 | 0.008 | 0.001 | 6.59994e-08 | 460162 | 29.182 |
| Pork intake | rs4672226 | 2 | 58203057 | T | C | 0.415 | 0.007 | 0.002 | 2.30001e-06 | 460162 | 22.324 |
| Pork intake | rs4704780 | 5 | 158246785 | C | A | 0.085 | 0.013 | 0.003 | 4.30002e-07 | 460162 | 25.544 |
| Pork intake | rs4738862 | 8 | 61996428 | A | C | 0.24 | -0.008 | 0.002 | 4.09996e-06 | 460162 | 21.229 |
| Pork intake | rs482202 | 11 | 28785216 | A | G | 0.56 | -0.007 | 0.001 | 3.50002e-06 | 460162 | 21.541 |
| Pork intake | rs62019153 | 15 | 50829185 | G | A | 0.209 | -0.009 | 0.002 | 8.09991e-07 | 460162 | 24.324 |
| Pork intake | rs62062058 | 17 | 11197508 | C | T | 0.213 | 0.01 | 0.002 | 1.2e-07 | 460162 | 28.02 |
| Pork intake | rs6466020 | 7 | 77727707 | T | C | 0.437 | -0.007 | 0.001 | 1.7e-06 | 460162 | 22.956 |
| Pork intake | rs66509940 | 7 | 99101503 | T | C | 0.089 | -0.014 | 0.003 | 8.9e-08 | 460162 | 28.59 |
| Pork intake | rs6909236 | 6 | 66249610 | C | T | 0.262 | -0.008 | 0.002 | 2.59998e-06 | 460162 | 22.055 |
| Pork intake | rs72909605 | 18 | 45819621 | T | C | 0.192 | 0.009 | 0.002 | 1.09999e-06 | 460162 | 23.726 |
| Pork intake | rs74533665 | 12 | 113225583 | A | G | 0.069 | 0.014 | 0.003 | 2.69998e-06 | 460162 | 22.048 |
| Pork intake | rs74639781 | 3 | 104888600 | T | C | 0.021 | -0.024 | 0.005 | 3.50002e-06 | 460162 | 21.541 |
| Pork intake | rs7641973 | 3 | 81910489 | A | G | 0.353 | 0.008 | 0.002 | 4.20001e-08 | 460162 | 30.042 |
| Pork intake | rs7644667 | 3 | 69040601 | C | T | 0.546 | 0.007 | 0.001 | 2.80001e-06 | 460162 | 21.978 |
| Pork intake | rs7969045 | 12 | 60260089 | T | C | 0.484 | 0.008 | 0.001 | 1.5e-07 | 460162 | 27.61 |
| Pork intake | rs79729037 | 3 | 62509517 | T | C | 0.023 | 0.023 | 0.005 | 2.39999e-06 | 460162 | 22.263 |
| Pork intake | rs838133 | 19 | 49259529 | G | A | 0.549 | 0.011 | 0.002 | 8.99912e-13 | 460162 | 51.041 |
| Pork intake | rs905112 | 1 | 37715396 | A | C | 0.531 | 0.007 | 0.001 | 6.49995e-07 | 460162 | 24.743 |
| Pork intake | rs9326286 | 11 | 126666201 | G | T | 0.39 | -0.007 | 0.002 | 1.79999e-06 | 460162 | 22.798 |
| Pork intake | rs9527954 | 13 | 59787228 | T | C | 0.268 | -0.008 | 0.002 | 3.2e-06 | 460162 | 21.666 |
| Pork intake | rs9696811 | 9 | 131923530 | C | T | 0.686 | 0.008 | 0.002 | 2.59998e-06 | 460162 | 22.086 |
| Pork intake | rs9783122 | 10 | 106766398 | A | G | 0.193 | 0.009 | 0.002 | 6.80002e-07 | 460162 | 24.679 |
| Pork intake | rs9914272 | 17 | 79062815 | G | T | 0.509 | -0.007 | 0.001 | 2.80001e-06 | 460162 | 21.926 |
| Pork intake | rs9973426 | 2 | 155751852 | G | A | 0.177 | 0.011 | 0.002 | 1e-08 | 460162 | 32.756 |
| Poultry intake | rs1000294 | 8 | 23080135 | A | G | 0.247 | 0.01 | 0.002 | 3.29997e-06 | 461900 | 21.659 |
| Poultry intake | rs10060023 | 5 | 124304677 | C | T | 0.666 | 0.009 | 0.002 | 7.79992e-07 | 461900 | 24.412 |
| Poultry intake | rs10133989 | 14 | 101879553 | A | G | 0.648 | 0.011 | 0.002 | 8.60003e-07 | 461900 | 24.211 |
| Poultry intake | rs10224539 | 7 | 105002002 | C | T | 0.461 | -0.009 | 0.002 | 1.40001e-06 | 461900 | 23.283 |
| Poultry intake | rs1051730 | 15 | 78894339 | A | G | 0.331 | -0.011 | 0.002 | 1.7e-08 | 461900 | 31.776 |
| Poultry intake | rs1062633 | 3 | 49924940 | C | T | 0.516 | 0.009 | 0.002 | 4e-07 | 461900 | 25.689 |
| Poultry intake | rs111536461 | 11 | 44837396 | A | G | 0.052 | 0.02 | 0.004 | 1.2e-06 | 461900 | 23.641 |
| Poultry intake | rs113631743 | 16 | 14530726 | A | G | 0.216 | -0.011 | 0.002 | 8.30004e-07 | 461900 | 24.276 |
| Poultry intake | rs114129389 | 2 | 228612544 | A | G | 0.074 | 0.017 | 0.004 | 3.29997e-06 | 461900 | 21.636 |
| Poultry intake | rs11586828 | 1 | 38023822 | A | G | 0.084 | 0.016 | 0.003 | 2.19999e-06 | 461900 | 22.446 |
| Poultry intake | rs116366915 | 5 | 97761102 | T | C | 0.024 | 0.029 | 0.006 | 1.89998e-06 | 461900 | 22.69 |
| Poultry intake | rs11678980 | 2 | 162101261 | A | G | 0.461 | 0.01 | 0.002 | 4e-07 | 461900 | 25.684 |
| Poultry intake | rs11698621 | 20 | 38764006 | G | A | 0.573 | -0.009 | 0.002 | 2.90001e-06 | 461900 | 21.885 |
| Poultry intake | rs11976316 | 7 | 142600456 | G | T | 0.056 | 0.019 | 0.004 | 1.6e-06 | 461900 | 22.987 |
| Poultry intake | rs12406278 | 1 | 190594968 | A | G | 0.187 | 0.012 | 0.002 | 5.60003e-07 | 461900 | 25.035 |
| Poultry intake | rs1256428 | 15 | 83521818 | T | C | 0.507 | -0.01 | 0.002 | 7.10003e-08 | 461900 | 29.027 |
| Poultry intake | rs1275183 | 13 | 30941805 | G | A | 0.864 | 0.013 | 0.003 | 1.79999e-06 | 461900 | 22.747 |
| Poultry intake | rs12928131 | 16 | 342771 | T | C | 0.167 | -0.011 | 0.002 | 2.5e-06 | 461900 | 22.185 |
| Poultry intake | rs13405357 | 2 | 227022036 | C | T | 0.339 | -0.009 | 0.002 | 9.40005e-07 | 461900 | 24.037 |
| Poultry intake | rs1367228 | 2 | 56112440 | A | C | 0.429 | 0.009 | 0.002 | 3.09999e-06 | 461900 | 21.762 |
| Poultry intake | rs144438383 | 6 | 80334700 | T | C | 0.013 | -0.04 | 0.008 | 1.6e-06 | 461900 | 23.075 |
| Poultry intake | rs146594921 | 5 | 35937088 | C | T | 0.017 | -0.034 | 0.007 | 1.79999e-06 | 461900 | 22.794 |
| Poultry intake | rs146816149 | 9 | 27783217 | C | T | 0.016 | -0.036 | 0.008 | 2.5e-06 | 461900 | 22.152 |
| Poultry intake | rs16855729 | 1 | 205494924 | T | C | 0.046 | 0.021 | 0.004 | 1.2e-06 | 461900 | 23.546 |
| Poultry intake | rs17767108 | 8 | 10274888 | T | C | 0.281 | -0.009 | 0.002 | 3.89996e-06 | 461900 | 21.328 |
| Poultry intake | rs186095 | 5 | 94250056 | C | A | 0.085 | 0.015 | 0.003 | 2.69998e-06 | 461900 | 22.027 |
| Poultry intake | rs1960425 | 5 | 167672105 | C | T | 0.541 | 0.008 | 0.002 | 3.89996e-06 | 461900 | 21.314 |
| Poultry intake | rs2195042 | 18 | 64551123 | A | C | 0.539 | -0.009 | 0.002 | 1.2e-06 | 461900 | 23.597 |
| Poultry intake | rs2426440 | 20 | 50999841 | G | A | 0.733 | 0.011 | 0.002 | 4.70002e-08 | 461900 | 29.849 |
| Poultry intake | rs2565017 | 18 | 21070280 | A | G | 0.373 | 0.011 | 0.002 | 5.89997e-09 | 461900 | 33.858 |
| Poultry intake | rs2696187 | 7 | 39043106 | C | T | 0.589 | 0.009 | 0.002 | 9.49992e-07 | 461900 | 24.02 |
| Poultry intake | rs28418529 | 7 | 153496441 | A | G | 0.501 | 0.009 | 0.002 | 2.99999e-06 | 461900 | 21.825 |
| Poultry intake | rs28790681 | 7 | 74035116 | T | C | 0.284 | -0.009 | 0.002 | 3.89996e-06 | 461900 | 21.3 |
| Poultry intake | rs2965200 | 19 | 19476365 | A | G | 0.64 | -0.01 | 0.002 | 4.20001e-08 | 461900 | 30.047 |
| Poultry intake | rs34344257 | 16 | 89740823 | T | C | 0.228 | -0.01 | 0.002 | 3.79997e-06 | 461900 | 21.389 |
| Poultry intake | rs34552305 | 1 | 11537441 | T | C | 0.133 | 0.014 | 0.003 | 9.80009e-08 | 461900 | 28.413 |
| Poultry intake | rs35758541 | 5 | 168257174 | C | T | 0.398 | 0.009 | 0.002 | 3.2e-06 | 461900 | 21.712 |
| Poultry intake | rs437029 | 13 | 36251925 | C | T | 0.722 | -0.01 | 0.002 | 2.39999e-06 | 461900 | 22.281 |
| Poultry intake | rs4428348 | 4 | 113423989 | A | G | 0.602 | -0.009 | 0.002 | 3.79997e-07 | 461900 | 25.816 |
| Poultry intake | rs4437085 | 3 | 138085910 | A | G | 0.772 | 0.011 | 0.002 | 3.50002e-07 | 461900 | 25.963 |
| Poultry intake | rs4547132 | 11 | 112832813 | T | C | 0.269 | -0.009 | 0.002 | 3.50002e-06 | 461900 | 21.504 |
| Poultry intake | rs4627402 | 17 | 43932741 | T | C | 0.221 | -0.011 | 0.002 | 4.90004e-07 | 461900 | 25.307 |
| Poultry intake | rs4700474 | 5 | 61415727 | A | G | 0.382 | 0.009 | 0.002 | 1.09999e-06 | 461900 | 23.67 |
| Poultry intake | rs4799106 | 18 | 77671112 | A | G | 0.431 | -0.009 | 0.002 | 2.30001e-06 | 461900 | 22.363 |
| Poultry intake | rs4855038 | 3 | 181468876 | A | C | 0.357 | 0.009 | 0.002 | 4e-06 | 461900 | 21.253 |
| Poultry intake | rs4927088 | 1 | 54775203 | T | C | 0.149 | 0.012 | 0.003 | 1.89998e-06 | 461900 | 22.664 |
| Poultry intake | rs56238942 | 22 | 51182399 | G | A | 0.079 | -0.018 | 0.003 | 1.7e-07 | 461900 | 27.348 |
| Poultry intake | rs59154329 | 20 | 21507645 | A | G | 0.171 | -0.011 | 0.002 | 4e-06 | 461900 | 21.258 |
| Poultry intake | rs61812638 | 1 | 188209948 | T | C | 0.342 | -0.01 | 0.002 | 6.59994e-07 | 461900 | 24.727 |
| Poultry intake | rs6803184 | 3 | 10605048 | A | G | 0.334 | 0.009 | 0.002 | 3.79997e-06 | 461900 | 21.382 |
| Poultry intake | rs7045589 | 9 | 127110354 | T | C | 0.549 | -0.01 | 0.002 | 1e-07 | 461900 | 28.366 |
| Poultry intake | rs71390729 | 16 | 64723209 | C | T | 0.016 | -0.038 | 0.007 | 2.80001e-07 | 461900 | 26.358 |
| Poultry intake | rs72686742 | 4 | 102791830 | G | A | 0.02 | -0.032 | 0.007 | 1.6e-06 | 461900 | 22.966 |
| Poultry intake | rs72904998 | 2 | 70174217 | A | G | 0.152 | 0.012 | 0.003 | 8.79995e-07 | 461900 | 24.18 |
| Poultry intake | rs7464104 | 8 | 34524410 | A | G | 0.658 | -0.009 | 0.002 | 3.29997e-06 | 461900 | 21.608 |
| Poultry intake | rs74979260 | 5 | 135341462 | T | C | 0.029 | -0.028 | 0.005 | 2e-07 | 461900 | 27.035 |
| Poultry intake | rs7531243 | 1 | 69279047 | C | T | 0.829 | 0.013 | 0.002 | 1.89998e-07 | 461900 | 27.142 |
| Poultry intake | rs76141078 | 15 | 74127882 | T | C | 0.021 | 0.029 | 0.006 | 4.70002e-06 | 461900 | 20.973 |
| Poultry intake | rs7625597 | 3 | 18152465 | T | C | 0.537 | 0.01 | 0.002 | 1.09999e-07 | 461900 | 28.241 |
| Poultry intake | rs76536752 | 3 | 87938167 | A | C | 0.107 | -0.015 | 0.003 | 4.39997e-07 | 461900 | 25.526 |
| Poultry intake | rs77094162 | 9 | 126494475 | A | G | 0.11 | -0.014 | 0.003 | 2e-06 | 461900 | 22.637 |
| Poultry intake | rs77118273 | 1 | 91001475 | A | C | 0.1 | -0.014 | 0.003 | 2.5e-06 | 461900 | 22.17 |
| Poultry intake | rs77779142 | 11 | 65599656 | T | C | 0.166 | 0.013 | 0.002 | 2.19999e-07 | 461900 | 26.852 |
| Poultry intake | rs780108 | 2 | 27684957 | C | T | 0.445 | -0.009 | 0.002 | 3.69999e-07 | 461900 | 25.848 |
| Poultry intake | rs7829800 | 8 | 144258705 | G | A | 0.671 | 0.011 | 0.002 | 3.69999e-09 | 461900 | 34.781 |
| Poultry intake | rs78693894 | 18 | 14424163 | T | C | 0.241 | 0.01 | 0.002 | 2.30001e-06 | 461900 | 22.351 |
| Poultry intake | rs7869395 | 9 | 137976605 | A | G | 0.186 | -0.011 | 0.002 | 1.6e-06 | 461900 | 23.018 |
| Poultry intake | rs78974715 | 20 | 59064185 | T | C | 0.231 | 0.01 | 0.002 | 4.90004e-06 | 461900 | 20.894 |
| Poultry intake | rs79175349 | 11 | 11968496 | G | A | 0.144 | -0.012 | 0.003 | 2.59998e-06 | 461900 | 22.128 |
| Poultry intake | rs8013402 | 14 | 73748498 | C | T | 0.188 | 0.011 | 0.002 | 4.60002e-06 | 461900 | 20.989 |
| Poultry intake | rs929175 | 7 | 126479218 | A | G | 0.357 | 0.009 | 0.002 | 2.99999e-06 | 461900 | 21.791 |
| Poultry intake | rs9300175 | 12 | 27726200 | T | C | 0.614 | 0.009 | 0.002 | 4.60002e-06 | 461900 | 20.99 |
| Poultry intake | rs9537989 | 13 | 58858430 | A | G | 0.212 | 0.011 | 0.002 | 5.49997e-07 | 461900 | 25.072 |
| Poultry intake | rs9923768 | 16 | 6163838 | A | G | 0.599 | 0.011 | 0.002 | 1.6e-08 | 461900 | 31.977 |
| Poultry intake | rs9997448 | 4 | 140870515 | T | C | 0.369 | -0.01 | 0.002 | 2.69998e-08 | 461900 | 30.889 |
| Processed meat intake | rs10109400 | 8 | 42455353 | C | T | 0.595 | -0.012 | 0.002 | 5.39995e-08 | 461981 | 29.585 |
| Processed meat intake | rs10128495 | 10 | 21123825 | T | G | 0.335 | 0.011 | 0.002 | 2.39999e-07 | 461981 | 26.66 |
| Processed meat intake | rs10203260 | 2 | 147451681 | T | C | 0.577 | 0.011 | 0.002 | 4.30002e-07 | 461981 | 25.576 |
| Processed meat intake | rs10411858 | 19 | 5036755 | A | G | 0.251 | -0.012 | 0.002 | 1.7e-06 | 461981 | 22.86 |
| Processed meat intake | rs10454812 | 5 | 52800358 | C | A | 0.103 | -0.02 | 0.003 | 6.69993e-09 | 461981 | 33.626 |
| Processed meat intake | rs1077875 | 15 | 79826072 | C | T | 0.542 | 0.01 | 0.002 | 3.2e-06 | 461981 | 21.664 |
| Processed meat intake | rs10910967 | 1 | 181543835 | G | A | 0.872 | -0.015 | 0.003 | 3.2e-06 | 461981 | 21.687 |
| Processed meat intake | rs10972056 | 9 | 34318708 | C | A | 0.794 | 0.012 | 0.003 | 2.39999e-06 | 461981 | 22.235 |
| Processed meat intake | rs111485956 | 14 | 72467825 | T | C | 0.019 | -0.039 | 0.008 | 1.5e-06 | 461981 | 23.207 |
| Processed meat intake | rs113443850 | 3 | 76332927 | A | G | 0.386 | 0.01 | 0.002 | 1.2e-06 | 461981 | 23.611 |
| Processed meat intake | rs114687117 | 1 | 107176622 | A | G | 0.029 | -0.029 | 0.006 | 2.5e-06 | 461981 | 22.132 |
| Processed meat intake | rs115608101 | 11 | 107106532 | T | C | 0.13 | -0.017 | 0.003 | 1.2e-07 | 461981 | 28.035 |
| Processed meat intake | rs117631241 | 14 | 46347595 | A | G | 0.014 | -0.045 | 0.009 | 1.29999e-06 | 461981 | 23.422 |
| Processed meat intake | rs117665299 | 9 | 3454966 | T | C | 0.027 | 0.033 | 0.007 | 7.79992e-07 | 461981 | 24.401 |
| Processed meat intake | rs11786089 | 8 | 21975521 | G | A | 0.46 | 0.011 | 0.002 | 1.2e-07 | 461981 | 28.067 |
| Processed meat intake | rs117999798 | 17 | 16330404 | G | T | 0.015 | 0.04 | 0.009 | 4.30002e-06 | 461981 | 21.12 |
| Processed meat intake | rs11887120 | 2 | 25485735 | T | C | 0.398 | 0.012 | 0.002 | 3.09999e-08 | 461981 | 30.657 |
| Processed meat intake | rs11894162 | 2 | 107197420 | T | C | 0.547 | 0.012 | 0.002 | 1.09999e-08 | 461981 | 32.702 |
| Processed meat intake | rs11930453 | 4 | 141710146 | C | A | 0.587 | -0.01 | 0.002 | 4.60002e-06 | 461981 | 21.008 |
| Processed meat intake | rs12143085 | 1 | 205073842 | G | A | 0.476 | -0.011 | 0.002 | 9.69996e-08 | 461981 | 28.44 |
| Processed meat intake | rs12153596 | 5 | 158410178 | T | C | 0.387 | 0.011 | 0.002 | 8.09991e-07 | 461981 | 24.328 |
| Processed meat intake | rs12245149 | 10 | 65321147 | A | C | 0.486 | 0.01 | 0.002 | 8e-07 | 461981 | 24.355 |
| Processed meat intake | rs1250597 | 10 | 81010250 | G | A | 0.595 | 0.01 | 0.002 | 1.09999e-06 | 461981 | 23.675 |
| Processed meat intake | rs12593984 | 15 | 92546412 | G | A | 0.335 | -0.01 | 0.002 | 2.69998e-06 | 461981 | 21.993 |
| Processed meat intake | rs12641854 | 4 | 115724466 | C | T | 0.107 | -0.017 | 0.003 | 7.49998e-07 | 461981 | 24.48 |
| Processed meat intake | rs12788387 | 11 | 57893347 | C | A | 0.3 | -0.011 | 0.002 | 1.6e-06 | 461981 | 23.03 |
| Processed meat intake | rs13134207 | 4 | 59848379 | T | G | 0.488 | -0.01 | 0.002 | 1.29999e-06 | 461981 | 23.428 |
| Processed meat intake | rs13165648 | 5 | 49865159 | C | A | 0.137 | -0.014 | 0.003 | 4.49997e-06 | 461981 | 21.023 |
| Processed meat intake | rs13403176 | 2 | 104042258 | C | T | 0.082 | -0.018 | 0.004 | 2.59998e-06 | 461981 | 22.069 |
| Processed meat intake | rs139911 | 22 | 40704052 | T | C | 0.576 | 0.01 | 0.002 | 1.7e-06 | 461981 | 22.877 |
| Processed meat intake | rs140221071 | 1 | 231760376 | A | G | 0.018 | 0.037 | 0.008 | 3.89996e-06 | 461981 | 21.32 |
| Processed meat intake | rs1422192 | 5 | 87959023 | A | G | 0.158 | 0.017 | 0.003 | 3.40001e-09 | 461981 | 34.923 |
| Processed meat intake | rs144497252 | 12 | 42990835 | C | T | 0.026 | -0.032 | 0.007 | 7.90005e-07 | 461981 | 24.388 |
| Processed meat intake | rs16881679 | 6 | 19085874 | A | G | 0.107 | 0.016 | 0.003 | 2.99999e-06 | 461981 | 21.807 |
| Processed meat intake | rs16919108 | 8 | 69629087 | C | T | 0.096 | -0.017 | 0.004 | 3.50002e-06 | 461981 | 21.52 |
| Processed meat intake | rs17327806 | 9 | 87398906 | T | G | 0.036 | 0.027 | 0.006 | 2.59998e-06 | 461981 | 22.065 |
| Processed meat intake | rs17404096 | 20 | 43391186 | C | T | 0.318 | -0.012 | 0.002 | 1.79999e-07 | 461981 | 27.258 |
| Processed meat intake | rs17755786 | 3 | 114940210 | T | C | 0.52 | 0.01 | 0.002 | 1.89998e-06 | 461981 | 22.716 |
| Processed meat intake | rs17836077 | 9 | 22474884 | G | A | 0.164 | 0.014 | 0.003 | 9.59997e-07 | 461981 | 24.012 |
| Processed meat intake | rs1820555 | 2 | 126468254 | A | G | 0.804 | 0.014 | 0.003 | 2.5e-07 | 461981 | 26.605 |
| Processed meat intake | rs188029213 | 10 | 946248 | C | T | 0.01 | 0.055 | 0.011 | 9.69996e-07 | 461981 | 23.986 |
| Processed meat intake | rs1961527 | 3 | 48764954 | G | A | 0.176 | -0.014 | 0.003 | 3.29997e-07 | 461981 | 26.074 |
| Processed meat intake | rs2029401 | 5 | 92891029 | G | A | 0.586 | 0.015 | 0.002 | 6.29941e-12 | 461981 | 47.22 |
| Processed meat intake | rs203319 | 22 | 41914593 | T | C | 0.205 | -0.016 | 0.003 | 2.80001e-10 | 461981 | 39.841 |
| Processed meat intake | rs2837899 | 21 | 42382043 | C | T | 0.154 | -0.014 | 0.003 | 1.7e-06 | 461981 | 22.894 |
| Processed meat intake | rs28654607 | 3 | 66084460 | A | G | 0.236 | -0.012 | 0.002 | 6.19998e-07 | 461981 | 24.836 |
| Processed meat intake | rs2873054 | 3 | 81888255 | C | A | 0.353 | 0.014 | 0.002 | 1.6e-10 | 461981 | 40.847 |
| Processed meat intake | rs34241936 | 17 | 74065908 | G | A | 0.037 | 0.033 | 0.006 | 1.09999e-08 | 461981 | 32.574 |
| Processed meat intake | rs35267287 | 15 | 44167187 | A | G | 0.151 | 0.014 | 0.003 | 2.80001e-06 | 461981 | 21.948 |
| Processed meat intake | rs35716147 | 17 | 36132131 | C | T | 0.094 | 0.017 | 0.004 | 1.5e-06 | 461981 | 23.093 |
| Processed meat intake | rs3733421 | 4 | 149002841 | C | T | 0.587 | -0.011 | 0.002 | 9.80009e-08 | 461981 | 28.413 |
| Processed meat intake | rs3762621 | 2 | 173306140 | T | C | 0.183 | -0.015 | 0.003 | 3.59998e-08 | 461981 | 30.377 |
| Processed meat intake | rs3809162 | 12 | 54674235 | G | A | 0.408 | 0.011 | 0.002 | 4.70002e-07 | 461981 | 25.396 |
| Processed meat intake | rs4016219 | 12 | 98618921 | A | G | 0.69 | 0.011 | 0.002 | 5.19996e-07 | 461981 | 25.188 |
| Processed meat intake | rs4077924 | 2 | 181991389 | C | T | 0.702 | 0.012 | 0.002 | 4.49997e-08 | 461981 | 29.928 |
| Processed meat intake | rs41286971 | 13 | 41026812 | A | G | 0.402 | -0.01 | 0.002 | 4.60002e-06 | 461981 | 21.006 |
| Processed meat intake | rs4133324 | 4 | 93322977 | C | T | 0.239 | 0.013 | 0.003 | 1.09999e-07 | 461981 | 28.106 |
| Processed meat intake | rs4240672 | 8 | 10767917 | A | G | 0.494 | 0.017 | 0.002 | 2.99985e-16 | 461981 | 66.836 |
| Processed meat intake | rs444558 | 9 | 102088185 | A | G | 0.45 | 0.01 | 0.002 | 8.40001e-07 | 461981 | 24.274 |
| Processed meat intake | rs4653392 | 1 | 34595736 | T | C | 0.389 | -0.011 | 0.002 | 5.99998e-07 | 461981 | 24.899 |
| Processed meat intake | rs4661151 | 1 | 155903408 | T | C | 0.675 | -0.011 | 0.002 | 7.59994e-07 | 461981 | 24.468 |
| Processed meat intake | rs4813338 | 20 | 18645033 | G | A | 0.517 | 0.01 | 0.002 | 6.59994e-07 | 461981 | 24.734 |
| Processed meat intake | rs4963586 | 12 | 24862473 | G | A | 0.423 | -0.011 | 0.002 | 5.89997e-07 | 461981 | 24.939 |
| Processed meat intake | rs4965927 | 15 | 98935186 | T | C | 0.63 | -0.01 | 0.002 | 2.99999e-06 | 461981 | 21.81 |
| Processed meat intake | rs56265815 | 4 | 66649923 | T | G | 0.1 | 0.017 | 0.003 | 1.2e-06 | 461981 | 23.6 |
| Processed meat intake | rs56281131 | 1 | 160094695 | G | T | 0.028 | 0.031 | 0.006 | 1e-06 | 461981 | 23.876 |
| Processed meat intake | rs6010651 | 20 | 62418243 | C | A | 0.379 | -0.012 | 0.002 | 1.09999e-08 | 461981 | 32.746 |
| Processed meat intake | rs6135282 | 20 | 14841065 | A | G | 0.421 | 0.01 | 0.002 | 4.70002e-06 | 461981 | 20.955 |
| Processed meat intake | rs614719 | 1 | 14536470 | T | C | 0.453 | 0.011 | 0.002 | 1.7e-07 | 461981 | 27.343 |
| Processed meat intake | rs62141888 | 2 | 58541063 | T | C | 0.191 | 0.015 | 0.003 | 5.99998e-08 | 461981 | 29.379 |
| Processed meat intake | rs62471101 | 7 | 109143783 | T | C | 0.12 | -0.015 | 0.003 | 2.19999e-06 | 461981 | 22.375 |
| Processed meat intake | rs627795 | 9 | 22663104 | G | A | 0.879 | 0.015 | 0.003 | 4.30002e-06 | 461981 | 21.125 |
| Processed meat intake | rs6484504 | 11 | 31424823 | C | T | 0.725 | 0.015 | 0.002 | 4.40048e-11 | 461981 | 43.429 |
| Processed meat intake | rs6587149 | 17 | 21290065 | G | A | 0.872 | -0.017 | 0.003 | 1.40001e-06 | 461981 | 23.238 |
| Processed meat intake | rs665404 | 1 | 88799915 | C | T | 0.712 | 0.011 | 0.002 | 2e-06 | 461981 | 22.558 |
| Processed meat intake | rs6765179 | 3 | 25276416 | A | G | 0.31 | -0.013 | 0.002 | 1.79999e-08 | 461981 | 31.709 |
| Processed meat intake | rs6786550 | 3 | 62560523 | C | T | 0.635 | 0.012 | 0.002 | 2.1e-08 | 461981 | 31.423 |
| Processed meat intake | rs6909218 | 6 | 40508393 | G | A | 0.442 | -0.011 | 0.002 | 3.2e-07 | 461981 | 26.115 |
| Processed meat intake | rs6961970 | 7 | 113901132 | A | C | 0.245 | -0.014 | 0.002 | 9.49992e-09 | 461981 | 32.951 |
| Processed meat intake | rs7174121 | 15 | 46566636 | G | A | 0.417 | -0.011 | 0.002 | 6.49995e-07 | 461981 | 24.766 |
| Processed meat intake | rs7176446 | 15 | 27065366 | G | A | 0.225 | -0.012 | 0.003 | 1.79999e-06 | 461981 | 22.766 |
| Processed meat intake | rs72681540 | 8 | 142174344 | A | G | 0.162 | -0.014 | 0.003 | 3.79997e-07 | 461981 | 25.77 |
| Processed meat intake | rs7297175 | 12 | 56473808 | C | T | 0.569 | 0.01 | 0.002 | 9.59997e-07 | 461981 | 24.014 |
| Processed meat intake | rs73137142 | 5 | 93079288 | A | G | 0.065 | 0.023 | 0.004 | 1.09999e-07 | 461981 | 28.127 |
| Processed meat intake | rs73181005 | 8 | 710588 | A | C | 0.07 | 0.022 | 0.004 | 5.49997e-08 | 461981 | 29.544 |
| Processed meat intake | rs73238074 | 7 | 127669584 | C | T | 0.134 | -0.015 | 0.003 | 6.80002e-07 | 461981 | 24.672 |
| Processed meat intake | rs7412 | 19 | 45412079 | T | C | 0.08 | 0.02 | 0.004 | 2.69998e-07 | 461981 | 26.477 |
| Processed meat intake | rs75129788 | 5 | 28343989 | C | T | 0.043 | -0.026 | 0.005 | 5.49997e-07 | 461981 | 25.079 |
| Processed meat intake | rs7521527 | 1 | 96872298 | A | G | 0.443 | -0.01 | 0.002 | 7.00003e-07 | 461981 | 24.604 |
| Processed meat intake | rs7531118 | 1 | 72837239 | C | T | 0.531 | -0.014 | 0.002 | 2.80027e-11 | 461981 | 44.331 |
| Processed meat intake | rs7560257 | 2 | 161085348 | T | C | 0.153 | 0.014 | 0.003 | 1e-06 | 461981 | 23.848 |
| Processed meat intake | rs76587391 | 16 | 7359167 | T | C | 0.1 | 0.017 | 0.004 | 8.40001e-07 | 461981 | 24.255 |
| Processed meat intake | rs76900637 | 11 | 13318789 | C | T | 0.035 | -0.03 | 0.006 | 1.6e-07 | 461981 | 27.465 |
| Processed meat intake | rs77165542 | 2 | 430975 | T | C | 0.035 | 0.034 | 0.006 | 3.29997e-09 | 461981 | 35.018 |
| Processed meat intake | rs77194050 | 16 | 70687185 | G | A | 0.052 | -0.023 | 0.005 | 2.30001e-06 | 461981 | 22.365 |
| Processed meat intake | rs77947964 | 10 | 43446294 | A | G | 0.099 | -0.017 | 0.004 | 1.5e-06 | 461981 | 23.156 |
| Processed meat intake | rs78397471 | 8 | 1742725 | C | T | 0.102 | 0.016 | 0.003 | 3.50002e-06 | 461981 | 21.518 |
| Processed meat intake | rs78553019 | 19 | 42675725 | G | A | 0.113 | 0.016 | 0.003 | 1.5e-06 | 461981 | 23.095 |
| Processed meat intake | rs8096167 | 18 | 34800257 | C | T | 0.193 | -0.015 | 0.003 | 4.70002e-08 | 461981 | 29.856 |
| Processed meat intake | rs838133 | 19 | 49259529 | G | A | 0.549 | 0.019 | 0.002 | 1.59993e-18 | 461981 | 77.089 |
| Processed meat intake | rs871974 | 2 | 65557287 | C | T | 0.297 | 0.011 | 0.002 | 2.39999e-06 | 461981 | 22.249 |
| Processed meat intake | rs9341169 | 2 | 217517150 | T | C | 0.014 | -0.052 | 0.01 | 4.70002e-07 | 461981 | 25.4 |
| Processed meat intake | rs9365786 | 6 | 165274798 | A | G | 0.257 | 0.011 | 0.002 | 3.69999e-06 | 461981 | 21.392 |
| Processed meat intake | rs9555936 | 13 | 90412722 | C | A | 0.391 | -0.011 | 0.002 | 4.70002e-07 | 461981 | 25.401 |
| Processed meat intake | rs959346 | 12 | 20852086 | A | G | 0.561 | -0.01 | 0.002 | 3.89996e-06 | 461981 | 21.318 |
| Processed meat intake | rs9809856 | 3 | 18227421 | G | A | 0.476 | 0.013 | 0.002 | 2.5e-10 | 461981 | 40.055 |
| Oily fish intake | rs10061973 | 5 | 166811498 | T | G | 0.514 | -0.011 | 0.002 | 1.5e-08 | 460443 | 32.081 |
| Oily fish intake | rs10076975 | 5 | 164518993 | C | T | 0.381 | 0.011 | 0.002 | 1.09999e-08 | 460443 | 32.643 |
| Oily fish intake | rs10137526 | 14 | 98886128 | T | G | 0.288 | -0.011 | 0.002 | 7.29995e-08 | 460443 | 28.99 |
| Oily fish intake | rs10510554 | 3 | 25099776 | C | T | 0.569 | 0.011 | 0.002 | 1.2e-08 | 460443 | 32.533 |
| Oily fish intake | rs10513136 | 3 | 141107612 | A | G | 0.065 | -0.023 | 0.004 | 1.6e-09 | 460443 | 36.451 |
| Oily fish intake | rs1053924 | 6 | 32120715 | C | T | 0.686 | -0.013 | 0.002 | 1.40001e-09 | 460443 | 36.713 |
| Oily fish intake | rs10744777 | 12 | 112233018 | C | T | 0.326 | -0.009 | 0.002 | 4.90004e-06 | 460443 | 20.866 |
| Oily fish intake | rs10819087 | 9 | 128695180 | G | A | 0.687 | -0.01 | 0.002 | 1.29999e-06 | 460443 | 23.461 |
| Oily fish intake | rs10864473 | 1 | 10883143 | A | G | 0.316 | -0.009 | 0.002 | 4.90004e-06 | 460443 | 20.88 |
| Oily fish intake | rs10880887 | 12 | 46412001 | T | C | 0.423 | -0.01 | 0.002 | 4.49997e-07 | 460443 | 25.448 |
| Oily fish intake | rs11025569 | 11 | 3160388 | T | C | 0.615 | -0.009 | 0.002 | 3.29997e-06 | 460443 | 21.632 |
| Oily fish intake | rs112139144 | 6 | 157106114 | A | G | 0.209 | -0.011 | 0.002 | 2e-06 | 460443 | 22.566 |
| Oily fish intake | rs11265908 | 9 | 92077132 | C | T | 0.387 | -0.01 | 0.002 | 2.80001e-07 | 460443 | 26.366 |
| Oily fish intake | rs114497213 | 3 | 115214586 | T | G | 0.055 | 0.027 | 0.004 | 1.09999e-10 | 460443 | 41.602 |
| Oily fish intake | rs11558171 | 16 | 58768129 | A | C | 0.14 | 0.013 | 0.003 | 1.79999e-06 | 460443 | 22.782 |
| Oily fish intake | rs11576565 | 1 | 72114787 | A | G | 0.489 | 0.01 | 0.002 | 4.20001e-07 | 460443 | 25.584 |
| Oily fish intake | rs11607886 | 11 | 83204132 | C | T | 0.584 | -0.012 | 0.002 | 2.90001e-09 | 460443 | 35.221 |
| Oily fish intake | rs11648715 | 16 | 65808760 | G | A | 0.132 | -0.013 | 0.003 | 2.5e-06 | 460443 | 22.164 |
| Oily fish intake | rs11740769 | 5 | 144467735 | T | C | 0.414 | -0.01 | 0.002 | 1.09999e-07 | 460443 | 28.139 |
| Oily fish intake | rs11747857 | 5 | 141426179 | T | C | 0.372 | -0.01 | 0.002 | 1.29999e-06 | 460443 | 23.368 |
| Oily fish intake | rs117551050 | 16 | 9541490 | C | A | 0.035 | 0.025 | 0.005 | 2.19999e-06 | 460443 | 22.38 |
| Oily fish intake | rs11767283 | 7 | 121947456 | G | A | 0.222 | 0.018 | 0.002 | 2.49977e-14 | 460443 | 58.06 |
| Oily fish intake | rs11777164 | 8 | 143297312 | T | C | 0.366 | 0.01 | 0.002 | 1.09999e-06 | 460443 | 23.791 |
| Oily fish intake | rs11859365 | 16 | 83683945 | C | A | 0.254 | 0.023 | 0.002 | 9.3994e-25 | 460443 | 105.527 |
| Oily fish intake | rs1201289 | 3 | 176822676 | G | T | 0.395 | -0.011 | 0.002 | 4.39997e-08 | 460443 | 29.966 |
| Oily fish intake | rs1229984 | 4 | 100239319 | C | T | 0.973 | -0.027 | 0.006 | 3.59998e-06 | 460443 | 21.492 |
| Oily fish intake | rs12576625 | 11 | 126596003 | C | T | 0.839 | 0.014 | 0.003 | 5.99998e-08 | 460443 | 29.359 |
| Oily fish intake | rs1260326 | 2 | 27730940 | C | T | 0.604 | -0.009 | 0.002 | 1.7e-06 | 460443 | 22.929 |
| Oily fish intake | rs1263459 | 14 | 83454485 | A | G | 0.426 | -0.009 | 0.002 | 2.59998e-06 | 460443 | 22.083 |
| Oily fish intake | rs12663865 | 6 | 88103149 | A | G | 0.758 | 0.013 | 0.002 | 1.09999e-08 | 460443 | 32.732 |
| Oily fish intake | rs12855717 | 13 | 101252635 | T | C | 0.527 | -0.012 | 0.002 | 2e-10 | 460443 | 40.457 |
| Oily fish intake | rs12906493 | 15 | 35933286 | C | T | 0.211 | 0.011 | 0.002 | 3.29997e-06 | 460443 | 21.633 |
| Oily fish intake | rs12906962 | 15 | 95312071 | C | T | 0.32 | 0.01 | 0.002 | 2.19999e-06 | 460443 | 22.437 |
| Oily fish intake | rs12966327 | 18 | 73208445 | T | C | 0.329 | -0.011 | 0.002 | 6.19998e-08 | 460443 | 29.305 |
| Oily fish intake | rs12983532 | 19 | 18467322 | T | C | 0.251 | -0.013 | 0.002 | 2e-09 | 460443 | 35.944 |
| Oily fish intake | rs13023088 | 2 | 100576304 | T | C | 0.149 | 0.014 | 0.003 | 4.39997e-07 | 460443 | 25.53 |
| Oily fish intake | rs13058717 | 22 | 27267178 | C | T | 0.432 | 0.009 | 0.002 | 2.1e-06 | 460443 | 22.513 |
| Oily fish intake | rs13113700 | 4 | 132569965 | A | C | 0.19 | -0.011 | 0.002 | 2.90001e-06 | 460443 | 21.908 |
| Oily fish intake | rs13138393 | 4 | 3381990 | A | G | 0.282 | -0.01 | 0.002 | 1.29999e-06 | 460443 | 23.358 |
| Oily fish intake | rs1339229 | 6 | 73158346 | G | A | 0.483 | 0.01 | 0.002 | 1.5e-07 | 460443 | 27.58 |
| Oily fish intake | rs1361016 | 13 | 54220229 | G | T | 0.845 | 0.015 | 0.003 | 1.7e-08 | 460443 | 31.784 |
| Oily fish intake | rs139348162 | 14 | 98092019 | T | G | 0.031 | -0.028 | 0.006 | 1.89998e-06 | 460443 | 22.737 |
| Oily fish intake | rs1421085 | 16 | 53800954 | C | T | 0.403 | 0.018 | 0.002 | 2.49977e-21 | 460443 | 89.942 |
| Oily fish intake | rs1427781 | 3 | 140173355 | T | C | 0.143 | 0.013 | 0.003 | 2.99999e-06 | 460443 | 21.823 |
| Oily fish intake | rs144377835 | 3 | 70581216 | T | C | 0.039 | -0.026 | 0.005 | 3.50002e-07 | 460443 | 25.976 |
| Oily fish intake | rs1532480 | 1 | 74236278 | T | G | 0.543 | 0.01 | 0.002 | 7.59994e-07 | 460443 | 24.454 |
| Oily fish intake | rs1558542 | 7 | 55134272 | T | C | 0.189 | 0.011 | 0.002 | 2.69998e-06 | 460443 | 22.023 |
| Oily fish intake | rs16850013 | 3 | 165785992 | A | G | 0.169 | -0.012 | 0.003 | 2.1e-06 | 460443 | 22.506 |
| Oily fish intake | rs16891727 | 6 | 26488860 | A | C | 0.13 | -0.024 | 0.003 | 6.79986e-17 | 460443 | 69.74 |
| Oily fish intake | rs17050031 | 2 | 59488019 | T | C | 0.48 | -0.012 | 0.002 | 3.50002e-10 | 460443 | 39.355 |
| Oily fish intake | rs17057158 | 6 | 129670548 | T | C | 0.053 | 0.02 | 0.004 | 2.59998e-06 | 460443 | 22.061 |
| Oily fish intake | rs170795 | 2 | 89153249 | G | T | 0.11 | -0.015 | 0.003 | 3.09999e-06 | 460443 | 21.728 |
| Oily fish intake | rs17414123 | 11 | 80768250 | T | C | 0.493 | 0.009 | 0.002 | 2.59998e-06 | 460443 | 22.083 |
| Oily fish intake | rs1821849 | 2 | 164747772 | C | T | 0.74 | -0.01 | 0.002 | 2.90001e-06 | 460443 | 21.887 |
| Oily fish intake | rs1876245 | 3 | 71534763 | C | T | 0.431 | 0.015 | 0.002 | 5.00035e-15 | 460443 | 61.266 |
| Oily fish intake | rs1925517 | 16 | 20144475 | A | G | 0.787 | -0.011 | 0.002 | 2.30001e-06 | 460443 | 22.362 |
| Oily fish intake | rs1951286 | 14 | 29766012 | G | T | 0.645 | -0.015 | 0.002 | 2.99985e-13 | 460443 | 53.196 |
| Oily fish intake | rs2092910 | 20 | 12150042 | T | C | 0.603 | -0.009 | 0.002 | 4.30002e-06 | 460443 | 21.105 |
| Oily fish intake | rs2271308 | 17 | 37817482 | C | T | 0.733 | 0.012 | 0.002 | 3.09999e-08 | 460443 | 30.63 |
| Oily fish intake | rs2591847 | 19 | 34807541 | A | G | 0.561 | -0.01 | 0.002 | 4.49997e-07 | 460443 | 25.465 |
| Oily fish intake | rs2601759 | 10 | 33845729 | C | T | 0.67 | -0.01 | 0.002 | 2.69998e-06 | 460443 | 22.055 |
| Oily fish intake | rs275160 | 2 | 145967878 | C | T | 0.701 | 0.012 | 0.002 | 8e-09 | 460443 | 33.272 |
| Oily fish intake | rs2827161 | 21 | 23340050 | G | T | 0.423 | 0.011 | 0.002 | 3.2e-08 | 460443 | 30.574 |
| Oily fish intake | rs28533540 | 15 | 47867762 | A | G | 0.534 | 0.015 | 0.002 | 2.80027e-14 | 460443 | 57.869 |
| Oily fish intake | rs303817 | 12 | 52176235 | G | A | 0.751 | 0.014 | 0.002 | 8e-10 | 460443 | 37.77 |
| Oily fish intake | rs3124402 | 13 | 55975115 | G | A | 0.733 | -0.022 | 0.002 | 1.9002e-24 | 460443 | 104.087 |
| Oily fish intake | rs321243 | 1 | 96476240 | G | T | 0.593 | 0.01 | 0.002 | 7.69999e-08 | 460443 | 28.892 |
| Oily fish intake | rs35287743 | 12 | 110057250 | T | G | 0.116 | -0.028 | 0.003 | 7.00003e-21 | 460443 | 87.857 |
| Oily fish intake | rs37013 | 5 | 102143618 | A | G | 0.635 | -0.01 | 0.002 | 5.19996e-07 | 460443 | 25.195 |
| Oily fish intake | rs3781441 | 10 | 126692423 | A | C | 0.168 | -0.013 | 0.003 | 8.9e-07 | 460443 | 24.161 |
| Oily fish intake | rs3847799 | 12 | 94545898 | C | A | 0.798 | -0.012 | 0.002 | 3.09999e-07 | 460443 | 26.185 |
| Oily fish intake | rs3923087 | 17 | 63549261 | C | T | 0.779 | 0.011 | 0.002 | 3.40001e-06 | 460443 | 21.571 |
| Oily fish intake | rs4002471 | 19 | 49215095 | T | C | 0.547 | -0.019 | 0.002 | 1.50003e-23 | 460443 | 99.999 |
| Oily fish intake | rs4131293 | 18 | 22772069 | T | C | 0.451 | 0.01 | 0.002 | 4.49997e-07 | 460443 | 25.454 |
| Oily fish intake | rs4261301 | 11 | 58114984 | T | C | 0.453 | 0.009 | 0.002 | 4.60002e-06 | 460443 | 20.998 |
| Oily fish intake | rs4419275 | 2 | 137447448 | C | T | 0.61 | 0.01 | 0.002 | 2.1e-07 | 460443 | 26.918 |
| Oily fish intake | rs45501495 | 1 | 204596454 | T | C | 0.236 | 0.016 | 0.002 | 3.69999e-12 | 460443 | 48.261 |
| Oily fish intake | rs4579097 | 4 | 150210668 | A | G | 0.225 | -0.011 | 0.002 | 2e-06 | 460443 | 22.635 |
| Oily fish intake | rs4778971 | 15 | 79472522 | G | A | 0.589 | -0.01 | 0.002 | 2e-07 | 460443 | 27.061 |
| Oily fish intake | rs4817505 | 21 | 34343828 | C | T | 0.39 | 0.009 | 0.002 | 3.40001e-06 | 460443 | 21.55 |
| Oily fish intake | rs4851487 | 2 | 102302978 | T | C | 0.397 | -0.009 | 0.002 | 2.39999e-06 | 460443 | 22.247 |
| Oily fish intake | rs4869859 | 6 | 155847549 | C | T | 0.45 | 0.014 | 0.002 | 3.10027e-13 | 460443 | 53.128 |
| Oily fish intake | rs4970581 | 1 | 38737005 | C | A | 0.468 | -0.01 | 0.002 | 3.29997e-07 | 460443 | 26.051 |
| Oily fish intake | rs4974084 | 3 | 48939080 | A | G | 0.666 | 0.009 | 0.002 | 3.59998e-06 | 460443 | 21.463 |
| Oily fish intake | rs4982738 | 14 | 23650191 | A | G | 0.583 | 0.011 | 0.002 | 3.50002e-08 | 460443 | 30.414 |
| Oily fish intake | rs552234 | 9 | 106772283 | A | G | 0.495 | -0.012 | 0.002 | 1.09999e-09 | 460443 | 37.089 |
| Oily fish intake | rs55688158 | 5 | 41396930 | G | T | 0.23 | -0.011 | 0.002 | 4.09996e-06 | 460443 | 21.214 |
| Oily fish intake | rs55701338 | 18 | 22701299 | C | A | 0.367 | -0.009 | 0.002 | 3.29997e-06 | 460443 | 21.626 |
| Oily fish intake | rs55930451 | 2 | 49039237 | T | C | 0.108 | -0.017 | 0.003 | 2.90001e-08 | 460443 | 30.744 |
| Oily fish intake | rs55985303 | 2 | 77224951 | A | G | 0.241 | 0.013 | 0.002 | 6.59994e-09 | 460443 | 33.657 |
| Oily fish intake | rs56096330 | 7 | 77791389 | A | G | 0.182 | -0.012 | 0.002 | 4.70002e-07 | 460443 | 25.368 |
| Oily fish intake | rs59355765 | 18 | 53431951 | T | C | 0.16 | -0.016 | 0.003 | 4.70002e-10 | 460443 | 38.784 |
| Oily fish intake | rs59733471 | 1 | 88436992 | T | G | 0.182 | 0.013 | 0.002 | 4.70002e-07 | 460443 | 25.388 |
| Oily fish intake | rs6000943 | 22 | 38324323 | C | T | 0.368 | 0.01 | 0.002 | 4.09996e-07 | 460443 | 25.64 |
| Oily fish intake | rs6021076 | 20 | 49868671 | G | T | 0.709 | 0.01 | 0.002 | 3.79997e-06 | 460443 | 21.339 |
| Oily fish intake | rs6033437 | 20 | 12495731 | A | C | 0.257 | 0.012 | 0.002 | 1.7e-08 | 460443 | 31.836 |
| Oily fish intake | rs6059844 | 20 | 33036482 | G | A | 0.495 | 0.011 | 0.002 | 9.20005e-09 | 460443 | 33.012 |
| Oily fish intake | rs6074041 | 20 | 44780150 | A | G | 0.229 | 0.012 | 0.002 | 4.49997e-07 | 460443 | 25.471 |
| Oily fish intake | rs6089753 | 20 | 61154107 | T | C | 0.531 | -0.012 | 0.002 | 1.79999e-09 | 460443 | 36.198 |
| Oily fish intake | rs6119946 | 20 | 31329704 | A | G | 0.425 | -0.01 | 0.002 | 5.89997e-07 | 460443 | 24.936 |
| Oily fish intake | rs61882686 | 11 | 46390680 | A | C | 0.085 | 0.02 | 0.003 | 8e-09 | 460443 | 33.264 |
| Oily fish intake | rs62458738 | 7 | 44066905 | A | G | 0.278 | 0.01 | 0.002 | 1.6e-06 | 460443 | 23.079 |
| Oily fish intake | rs6465487 | 7 | 95760694 | G | A | 0.4 | -0.012 | 0.002 | 2.69998e-10 | 460443 | 39.876 |
| Oily fish intake | rs6667502 | 1 | 177111162 | C | T | 0.475 | 0.01 | 0.002 | 1.6e-07 | 460443 | 27.513 |
| Oily fish intake | rs673604 | 1 | 35687815 | C | T | 0.083 | -0.016 | 0.003 | 2.80001e-06 | 460443 | 21.924 |
| Oily fish intake | rs6769454 | 3 | 117390399 | A | G | 0.038 | -0.025 | 0.005 | 4.30002e-07 | 460443 | 25.573 |
| Oily fish intake | rs6842641 | 4 | 30831942 | T | C | 0.214 | -0.012 | 0.002 | 1.09999e-07 | 460443 | 28.27 |
| Oily fish intake | rs6951862 | 7 | 111308595 | G | A | 0.662 | -0.01 | 0.002 | 1.89998e-06 | 460443 | 22.704 |
| Oily fish intake | rs6960970 | 7 | 14207740 | C | T | 0.804 | -0.013 | 0.002 | 5.30005e-08 | 460443 | 29.587 |
| Oily fish intake | rs6965147 | 7 | 3475240 | T | G | 0.453 | -0.01 | 0.002 | 2e-07 | 460443 | 27.006 |
| Oily fish intake | rs6988032 | 8 | 51493517 | T | G | 0.738 | -0.012 | 0.002 | 1.09999e-07 | 460443 | 28.176 |
| Oily fish intake | rs7147583 | 14 | 25536371 | A | G | 0.565 | -0.009 | 0.002 | 3.50002e-06 | 460443 | 21.543 |
| Oily fish intake | rs7225002 | 17 | 44189067 | G | A | 0.414 | -0.014 | 0.002 | 8.10028e-13 | 460443 | 51.253 |
| Oily fish intake | rs7243428 | 18 | 35156177 | G | A | 0.225 | -0.013 | 0.002 | 1.5e-08 | 460443 | 32.06 |
| Oily fish intake | rs72484727 | 11 | 7938306 | A | G | 0.394 | 0.01 | 0.002 | 2.1e-07 | 460443 | 26.978 |
| Oily fish intake | rs7254235 | 19 | 22212505 | G | A | 0.577 | -0.011 | 0.002 | 4.30002e-08 | 460443 | 30.022 |
| Oily fish intake | rs72821178 | 17 | 31764917 | C | T | 0.094 | -0.017 | 0.003 | 2.90001e-07 | 460443 | 26.285 |
| Oily fish intake | rs733069 | 9 | 8141845 | C | T | 0.256 | 0.01 | 0.002 | 3.40001e-06 | 460443 | 21.564 |
| Oily fish intake | rs7338243 | 13 | 72748285 | A | G | 0.244 | 0.011 | 0.002 | 6.90001e-07 | 460443 | 24.633 |
| Oily fish intake | rs7431970 | 3 | 88581056 | C | T | 0.811 | -0.013 | 0.002 | 1.7e-07 | 460443 | 27.339 |
| Oily fish intake | rs74575358 | 17 | 77539767 | C | T | 0.141 | -0.013 | 0.003 | 1.29999e-06 | 460443 | 23.42 |
| Oily fish intake | rs75887709 | 19 | 37438329 | G | A | 0.136 | -0.016 | 0.003 | 1.6e-08 | 460443 | 31.95 |
| Oily fish intake | rs77258251 | 3 | 111226958 | G | A | 0.052 | -0.021 | 0.004 | 7.00003e-07 | 460443 | 24.616 |
| Oily fish intake | rs7749444 | 6 | 57340919 | T | C | 0.47 | -0.009 | 0.002 | 3.09999e-06 | 460443 | 21.728 |
| Oily fish intake | rs77869596 | 9 | 72936849 | G | A | 0.028 | 0.031 | 0.006 | 1.2e-07 | 460443 | 28.076 |
| Oily fish intake | rs790564 | 8 | 64604218 | C | A | 0.723 | 0.015 | 0.002 | 7.89951e-12 | 460443 | 46.789 |
| Oily fish intake | rs79059303 | 10 | 64839681 | C | T | 0.238 | -0.011 | 0.002 | 1.29999e-06 | 460443 | 23.365 |
| Oily fish intake | rs7919624 | 10 | 2269933 | T | C | 0.38 | -0.01 | 0.002 | 3.59998e-07 | 460443 | 25.923 |
| Oily fish intake | rs7965154 | 12 | 23697725 | A | C | 0.313 | -0.01 | 0.002 | 4e-07 | 460443 | 25.672 |
| Oily fish intake | rs79655964 | 4 | 46757604 | C | T | 0.098 | -0.016 | 0.003 | 5.89997e-07 | 460443 | 24.944 |
| Oily fish intake | rs79785912 | 7 | 10809637 | C | T | 0.181 | -0.013 | 0.002 | 1.7e-07 | 460443 | 27.294 |
| Oily fish intake | rs903676 | 1 | 201754720 | A | G | 0.212 | 0.013 | 0.002 | 5.1e-08 | 460443 | 29.66 |
| Oily fish intake | rs9301837 | 13 | 93477312 | A | C | 0.143 | -0.016 | 0.003 | 8.10009e-09 | 460443 | 33.242 |
| Oily fish intake | rs957523 | 7 | 114349656 | C | T | 0.035 | 0.024 | 0.005 | 4.60002e-06 | 460443 | 20.985 |
| Oily fish intake | rs9597870 | 13 | 59412819 | G | T | 0.246 | -0.013 | 0.002 | 1.09999e-08 | 460443 | 32.65 |
| Oily fish intake | rs9606833 | 22 | 31750013 | C | T | 0.244 | 0.017 | 0.002 | 2.70023e-14 | 460443 | 57.971 |
| Oily fish intake | rs9831061 | 3 | 185835281 | A | G | 0.76 | 0.01 | 0.002 | 4.09996e-06 | 460443 | 21.204 |
| Oily fish intake | rs9841174 | 3 | 167184878 | C | T | 0.374 | 0.015 | 0.002 | 8.49963e-14 | 460443 | 55.692 |
| Oily fish intake | rs9860241 | 3 | 157845169 | A | G | 0.408 | 0.009 | 0.002 | 4.60002e-06 | 460443 | 21 |
| Oily fish intake | rs9889161 | 16 | 51495068 | T | G | 0.358 | -0.013 | 0.002 | 2.80027e-11 | 460443 | 44.29 |
| Oily fish intake | rs9958909 | 18 | 1841371 | G | T | 0.14 | 0.016 | 0.003 | 1.40001e-08 | 460443 | 32.189 |
| Non-oily fish intake | rs10421668 | 19 | 3226966 | A | G | 0.332 | -0.008 | 0.002 | 3.89996e-06 | 460880 | 21.299 |
| Non-oily fish intake | rs10844370 | 12 | 32984330 | C | T | 0.09 | 0.013 | 0.003 | 2.90001e-06 | 460880 | 21.856 |
| Non-oily fish intake | rs11165780 | 1 | 97564729 | A | G | 0.349 | -0.008 | 0.002 | 4.79999e-06 | 460880 | 20.9 |
| Non-oily fish intake | rs11183176 | 12 | 46099573 | T | G | 0.653 | -0.009 | 0.002 | 4e-07 | 460880 | 25.706 |
| Non-oily fish intake | rs112858262 | 18 | 11133481 | C | T | 0.277 | 0.008 | 0.002 | 4.20001e-06 | 460880 | 21.152 |
| Non-oily fish intake | rs113253914 | 22 | 42689945 | T | G | 0.081 | -0.015 | 0.003 | 1.79999e-06 | 460880 | 22.818 |
| Non-oily fish intake | rs116616457 | 1 | 116862180 | A | C | 0.012 | 0.038 | 0.008 | 4.39997e-06 | 460880 | 21.101 |
| Non-oily fish intake | rs11680516 | 2 | 208205711 | C | T | 0.202 | 0.012 | 0.002 | 1.40001e-09 | 460880 | 36.68 |
| Non-oily fish intake | rs11723103 | 4 | 62128825 | A | G | 0.442 | 0.008 | 0.002 | 1.89998e-06 | 460880 | 22.728 |
| Non-oily fish intake | rs117418277 | 11 | 82139196 | G | A | 0.039 | -0.022 | 0.004 | 6.59994e-07 | 460880 | 24.733 |
| Non-oily fish intake | rs117427411 | 8 | 144690402 | T | C | 0.023 | -0.028 | 0.006 | 1.09999e-06 | 460880 | 23.749 |
| Non-oily fish intake | rs11859365 | 16 | 83683945 | C | A | 0.254 | 0.01 | 0.002 | 1.89998e-07 | 460880 | 27.112 |
| Non-oily fish intake | rs12555454 | 9 | 116584812 | C | T | 0.373 | 0.009 | 0.002 | 1.5e-07 | 460880 | 27.572 |
| Non-oily fish intake | rs1260326 | 2 | 27730940 | C | T | 0.604 | -0.01 | 0.002 | 7.90005e-09 | 460880 | 33.31 |
| Non-oily fish intake | rs12638455 | 3 | 25122201 | A | C | 0.578 | 0.008 | 0.002 | 2.30001e-06 | 460880 | 22.349 |
| Non-oily fish intake | rs12912887 | 15 | 41257608 | T | G | 0.698 | 0.008 | 0.002 | 4.39997e-06 | 460880 | 21.091 |
| Non-oily fish intake | rs1445978 | 5 | 60855070 | C | T | 0.636 | -0.009 | 0.002 | 8.50002e-08 | 460880 | 28.699 |
| Non-oily fish intake | rs144833909 | 2 | 178267355 | T | C | 0.012 | -0.035 | 0.008 | 3.50002e-06 | 460880 | 21.518 |
| Non-oily fish intake | rs150075454 | 2 | 86212682 | A | G | 0.019 | -0.03 | 0.006 | 9.29994e-07 | 460880 | 24.069 |
| Non-oily fish intake | rs1603515 | 4 | 44870444 | G | A | 0.94 | 0.018 | 0.003 | 2e-07 | 460880 | 27.071 |
| Non-oily fish intake | rs16822430 | 2 | 144147475 | C | T | 0.233 | 0.012 | 0.002 | 1.40001e-09 | 460880 | 36.72 |
| Non-oily fish intake | rs17295822 | 10 | 32168803 | C | T | 0.081 | 0.016 | 0.003 | 9.59997e-08 | 460880 | 28.456 |
| Non-oily fish intake | rs17317920 | 7 | 25517103 | G | A | 0.479 | 0.009 | 0.002 | 2.80001e-08 | 460880 | 30.851 |
| Non-oily fish intake | rs17382631 | 1 | 43356211 | A | C | 0.17 | -0.011 | 0.002 | 1.7e-06 | 460880 | 22.943 |
| Non-oily fish intake | rs2400169 | 5 | 144526632 | C | T | 0.6 | -0.009 | 0.002 | 2.19999e-07 | 460880 | 26.869 |
| Non-oily fish intake | rs2447091 | 17 | 2296014 | C | T | 0.39 | -0.008 | 0.002 | 4.90004e-07 | 460880 | 25.291 |
| Non-oily fish intake | rs2702668 | 11 | 19422772 | G | A | 0.634 | 0.009 | 0.002 | 1.7e-07 | 460880 | 27.314 |
| Non-oily fish intake | rs35287743 | 12 | 110057250 | T | G | 0.116 | -0.018 | 0.003 | 3.59998e-12 | 460880 | 48.306 |
| Non-oily fish intake | rs35522344 | 14 | 100283856 | T | C | 0.385 | -0.008 | 0.002 | 1.40001e-06 | 460880 | 23.326 |
| Non-oily fish intake | rs3799077 | 6 | 69996217 | G | T | 0.31 | -0.011 | 0.002 | 1e-09 | 460880 | 37.3 |
| Non-oily fish intake | rs3809717 | 17 | 37886986 | A | C | 0.306 | 0.009 | 0.002 | 8.79995e-08 | 460880 | 28.627 |
| Non-oily fish intake | rs4318925 | 6 | 32761506 | T | C | 0.177 | -0.015 | 0.002 | 1.29987e-12 | 460880 | 50.281 |
| Non-oily fish intake | rs4415102 | 5 | 167710877 | T | C | 0.113 | -0.012 | 0.003 | 2.99999e-06 | 460880 | 21.831 |
| Non-oily fish intake | rs4953152 | 2 | 45170153 | A | G | 0.31 | -0.009 | 0.002 | 5.1e-07 | 460880 | 25.234 |
| Non-oily fish intake | rs4993942 | 3 | 76733503 | G | T | 0.47 | -0.009 | 0.002 | 1.09999e-07 | 460880 | 28.163 |
| Non-oily fish intake | rs55774808 | 15 | 95181973 | C | T | 0.171 | -0.011 | 0.002 | 1e-06 | 460880 | 23.882 |
| Non-oily fish intake | rs55858845 | 8 | 8991079 | C | T | 0.206 | 0.01 | 0.002 | 3.29997e-07 | 460880 | 26.081 |
| Non-oily fish intake | rs55900434 | 15 | 35789592 | C | T | 0.046 | 0.02 | 0.004 | 2.19999e-07 | 460880 | 26.855 |
| Non-oily fish intake | rs56094641 | 16 | 53806453 | G | A | 0.405 | 0.013 | 0.002 | 2.49977e-14 | 460880 | 58.122 |
| Non-oily fish intake | rs56399747 | 8 | 84643371 | G | A | 0.292 | 0.008 | 0.002 | 2.69998e-06 | 460880 | 22.004 |
| Non-oily fish intake | rs56848830 | 7 | 111112699 | A | C | 0.504 | 0.007 | 0.002 | 3.79997e-06 | 460880 | 21.367 |
| Non-oily fish intake | rs56864124 | 18 | 58859363 | A | C | 0.287 | -0.008 | 0.002 | 3.50002e-06 | 460880 | 21.505 |
| Non-oily fish intake | rs60132501 | 4 | 23854482 | T | C | 0.012 | 0.036 | 0.008 | 4e-06 | 460880 | 21.268 |
| Non-oily fish intake | rs6127088 | 20 | 52693624 | T | C | 0.173 | 0.01 | 0.002 | 1.6e-06 | 460880 | 23.04 |
| Non-oily fish intake | rs62034298 | 15 | 87935526 | T | C | 0.067 | 0.016 | 0.003 | 1.29999e-06 | 460880 | 23.374 |
| Non-oily fish intake | rs67646802 | 10 | 104303748 | T | C | 0.253 | -0.009 | 0.002 | 3.89996e-07 | 460880 | 25.747 |
| Non-oily fish intake | rs6953095 | 7 | 154213552 | C | A | 0.26 | 0.009 | 0.002 | 1e-06 | 460880 | 23.894 |
| Non-oily fish intake | rs6957745 | 7 | 73056750 | C | T | 0.203 | -0.012 | 0.002 | 1.79999e-09 | 460880 | 36.233 |
| Non-oily fish intake | rs7148387 | 14 | 29775132 | G | A | 0.591 | -0.009 | 0.002 | 1.7e-08 | 460880 | 31.804 |
| Non-oily fish intake | rs7326846 | 13 | 28664016 | T | C | 0.626 | 0.009 | 0.002 | 2.1e-07 | 460880 | 26.973 |
| Non-oily fish intake | rs7432182 | 3 | 115105933 | A | G | 0.672 | -0.008 | 0.002 | 1.7e-06 | 460880 | 22.927 |
| Non-oily fish intake | rs76477280 | 13 | 50147180 | G | A | 0.003 | 0.073 | 0.016 | 2.59998e-06 | 460880 | 22.079 |
| Non-oily fish intake | rs776472 | 7 | 114352862 | T | C | 0.567 | -0.008 | 0.002 | 2.1e-06 | 460880 | 22.517 |
| Non-oily fish intake | rs7791045 | 7 | 124385842 | C | T | 0.49 | 0.007 | 0.002 | 4e-06 | 460880 | 21.277 |
| Non-oily fish intake | rs77956594 | 11 | 8003669 | C | T | 0.132 | -0.013 | 0.002 | 1.89998e-07 | 460880 | 27.086 |
| Non-oily fish intake | rs78744083 | 11 | 110179047 | A | G | 0.017 | -0.029 | 0.006 | 4.90004e-06 | 460880 | 20.86 |
| Non-oily fish intake | rs78769094 | 2 | 204697332 | T | C | 0.017 | -0.03 | 0.006 | 2.39999e-06 | 460880 | 22.279 |
| Non-oily fish intake | rs79262908 | 3 | 185803594 | T | C | 0.192 | -0.01 | 0.002 | 5.49997e-07 | 460880 | 25.095 |
| Non-oily fish intake | rs838133 | 19 | 49259529 | G | A | 0.549 | 0.016 | 0.002 | 4.70002e-22 | 460880 | 93.216 |
| Non-oily fish intake | rs9674606 | 17 | 3638344 | A | G | 0.439 | 0.008 | 0.002 | 2.5e-06 | 460880 | 22.141 |
| Non-oily fish intake | rs9951835 | 18 | 1575201 | G | T | 0.293 | -0.008 | 0.002 | 4.30002e-06 | 460880 | 21.123 |
| Fresh fruit intake | rs10059461 | 5 | 108479058 | T | G | 0.298 | 0.007 | 0.001 | 2.80001e-07 | 446462 | 26.381 |
| Fresh fruit intake | rs10064431 | 5 | 92950673 | C | T | 0.522 | -0.008 | 0.001 | 5.99998e-10 | 446462 | 38.311 |
| Fresh fruit intake | rs10192394 | 2 | 146298007 | T | C | 0.529 | -0.008 | 0.001 | 4.49997e-10 | 446462 | 38.875 |
| Fresh fruit intake | rs10249294 | 7 | 143723137 | A | G | 0.373 | 0.02 | 0.001 | 4.10015e-54 | 446462 | 239.939 |
| Fresh fruit intake | rs10271924 | 7 | 153495206 | T | C | 0.493 | -0.007 | 0.001 | 2e-08 | 446462 | 31.481 |
| Fresh fruit intake | rs1035390 | 19 | 47763127 | C | T | 0.42 | -0.006 | 0.001 | 3.79997e-06 | 446462 | 21.349 |
| Fresh fruit intake | rs10412051 | 19 | 30914944 | T | C | 0.533 | 0.006 | 0.001 | 4.39997e-06 | 446462 | 21.092 |
| Fresh fruit intake | rs10501667 | 11 | 88265307 | G | A | 0.351 | -0.006 | 0.001 | 2e-06 | 446462 | 22.609 |
| Fresh fruit intake | rs1051547 | 16 | 19279380 | C | T | 0.562 | -0.008 | 0.001 | 1.09999e-09 | 446462 | 37.224 |
| Fresh fruit intake | rs10828266 | 10 | 22098701 | G | A | 0.716 | 0.012 | 0.001 | 8.10028e-20 | 446462 | 83.023 |
| Fresh fruit intake | rs10838724 | 11 | 47527052 | T | G | 0.368 | 0.009 | 0.001 | 2.09991e-12 | 446462 | 49.344 |
| Fresh fruit intake | rs10840126 | 11 | 8825774 | G | A | 0.376 | -0.008 | 0.001 | 1.89998e-09 | 446462 | 36.028 |
| Fresh fruit intake | rs10875917 | 12 | 49467330 | T | G | 0.433 | 0.006 | 0.001 | 1.09999e-06 | 446462 | 23.673 |
| Fresh fruit intake | rs11032362 | 11 | 33759092 | A | G | 0.091 | 0.012 | 0.002 | 5.30005e-09 | 446462 | 34.063 |
| Fresh fruit intake | rs11085749 | 19 | 10961273 | A | G | 0.387 | -0.008 | 0.001 | 7.10003e-10 | 446462 | 37.987 |
| Fresh fruit intake | rs11130633 | 3 | 58370792 | C | T | 0.666 | 0.006 | 0.001 | 5.30005e-07 | 446462 | 25.163 |
| Fresh fruit intake | rs11147593 | 13 | 36480042 | A | G | 0.741 | 0.007 | 0.001 | 2.99999e-06 | 446462 | 21.824 |
| Fresh fruit intake | rs111526888 | 18 | 57971625 | G | A | 0.28 | 0.01 | 0.001 | 3.69999e-14 | 446462 | 57.33 |
| Fresh fruit intake | rs11165976 | 1 | 98755006 | T | C | 0.326 | -0.007 | 0.001 | 5.99998e-08 | 446462 | 29.362 |
| Fresh fruit intake | rs115163723 | 2 | 43975647 | T | C | 0.029 | 0.017 | 0.004 | 2.99999e-06 | 446462 | 21.835 |
| Fresh fruit intake | rs115165749 | 2 | 236601676 | T | C | 0.056 | -0.012 | 0.003 | 4.49997e-06 | 446462 | 21.047 |
| Fresh fruit intake | rs11622939 | 14 | 93819216 | T | C | 0.248 | -0.007 | 0.001 | 1.09999e-06 | 446462 | 23.76 |
| Fresh fruit intake | rs11649274 | 16 | 30026469 | A | G | 0.401 | 0.007 | 0.001 | 1.29999e-07 | 446462 | 27.908 |
| Fresh fruit intake | rs117061004 | 10 | 84296245 | A | G | 0.033 | 0.016 | 0.003 | 4.60002e-06 | 446462 | 20.989 |
| Fresh fruit intake | rs1172553 | 13 | 48724166 | A | G | 0.936 | -0.012 | 0.002 | 3.50002e-06 | 446462 | 21.542 |
| Fresh fruit intake | rs11758313 | 6 | 7307342 | T | C | 0.314 | 0.007 | 0.001 | 5.39995e-07 | 446462 | 25.127 |
| Fresh fruit intake | rs11896330 | 2 | 60235568 | A | G | 0.633 | -0.008 | 0.001 | 3.40017e-11 | 446462 | 43.919 |
| Fresh fruit intake | rs11903377 | 2 | 10974489 | T | C | 0.162 | 0.008 | 0.002 | 1.40001e-06 | 446462 | 23.243 |
| Fresh fruit intake | rs11931740 | 4 | 40966909 | A | G | 0.255 | 0.007 | 0.001 | 2.80001e-06 | 446462 | 21.945 |
| Fresh fruit intake | rs11990258 | 8 | 15119313 | A | G | 0.246 | 0.007 | 0.001 | 1.5e-06 | 446462 | 23.104 |
| Fresh fruit intake | rs12044599 | 1 | 204564714 | G | A | 0.21 | 0.009 | 0.002 | 3.69999e-10 | 446462 | 39.271 |
| Fresh fruit intake | rs12446517 | 16 | 6936908 | G | T | 0.345 | -0.006 | 0.001 | 5e-07 | 446462 | 25.276 |
| Fresh fruit intake | rs12641371 | 4 | 59882235 | T | C | 0.433 | 0.008 | 0.001 | 1.40001e-10 | 446462 | 41.147 |
| Fresh fruit intake | rs12695010 | 2 | 241193279 | A | G | 0.379 | 0.006 | 0.001 | 3.40001e-06 | 446462 | 21.588 |
| Fresh fruit intake | rs12780952 | 10 | 107577033 | A | G | 0.286 | 0.007 | 0.001 | 3.40001e-08 | 446462 | 30.492 |
| Fresh fruit intake | rs13072255 | 3 | 21038260 | C | A | 0.494 | 0.009 | 0.001 | 2.09991e-13 | 446462 | 53.867 |
| Fresh fruit intake | rs133377 | 22 | 42466950 | T | C | 0.675 | -0.006 | 0.001 | 1.79999e-06 | 446462 | 22.834 |
| Fresh fruit intake | rs1356292 | 3 | 185824903 | T | C | 0.808 | 0.009 | 0.002 | 3.50002e-09 | 446462 | 34.89 |
| Fresh fruit intake | rs1375566 | 3 | 85642479 | A | G | 0.627 | -0.008 | 0.001 | 6.1e-10 | 446462 | 38.289 |
| Fresh fruit intake | rs139042899 | 17 | 58443095 | C | A | 0.013 | 0.036 | 0.006 | 3.2e-09 | 446462 | 35.037 |
| Fresh fruit intake | rs1411451 | 9 | 124893355 | T | C | 0.082 | -0.011 | 0.002 | 2.1e-06 | 446462 | 22.543 |
| Fresh fruit intake | rs1466781 | 15 | 47594706 | C | T | 0.42 | -0.006 | 0.001 | 3.89996e-06 | 446462 | 21.299 |
| Fresh fruit intake | rs1478254 | 1 | 241141391 | T | C | 0.512 | -0.006 | 0.001 | 3.29997e-06 | 446462 | 21.605 |
| Fresh fruit intake | rs149449 | 5 | 95902093 | A | G | 0.489 | 0.007 | 0.001 | 2.39999e-09 | 446462 | 35.655 |
| Fresh fruit intake | rs1620977 | 1 | 72729142 | G | A | 0.731 | -0.013 | 0.001 | 1.10002e-21 | 446462 | 91.452 |
| Fresh fruit intake | rs163543 | 3 | 3080527 | T | C | 0.941 | 0.012 | 0.003 | 4.39997e-06 | 446462 | 21.074 |
| Fresh fruit intake | rs17049185 | 2 | 58072660 | T | G | 0.268 | 0.008 | 0.001 | 7.29995e-09 | 446462 | 33.444 |
| Fresh fruit intake | rs17056301 | 5 | 158271680 | C | T | 0.257 | 0.006 | 0.001 | 4e-06 | 446462 | 21.266 |
| Fresh fruit intake | rs1723625 | 7 | 109687420 | A | C | 0.572 | -0.006 | 0.001 | 4.49997e-06 | 446462 | 21.043 |
| Fresh fruit intake | rs17238607 | 15 | 56396132 | A | G | 0.105 | -0.01 | 0.002 | 6.29999e-07 | 446462 | 24.803 |
| Fresh fruit intake | rs17258783 | 9 | 34253097 | T | C | 0.216 | -0.007 | 0.001 | 1.40001e-06 | 446462 | 23.342 |
| Fresh fruit intake | rs1746137 | 9 | 116140667 | T | C | 0.217 | 0.008 | 0.002 | 5e-07 | 446462 | 25.246 |
| Fresh fruit intake | rs1866823 | 8 | 57436577 | A | G | 0.544 | 0.007 | 0.001 | 2.1e-09 | 446462 | 35.876 |
| Fresh fruit intake | rs1916383 | 10 | 68674026 | T | C | 0.449 | 0.006 | 0.001 | 1.40001e-06 | 446462 | 23.241 |
| Fresh fruit intake | rs1964272 | 19 | 46190268 | A | G | 0.484 | 0.008 | 0.001 | 1.10002e-11 | 446462 | 46.132 |
| Fresh fruit intake | rs2093654 | 9 | 5780121 | G | A | 0.388 | 0.007 | 0.001 | 1.5e-08 | 446462 | 32.078 |
| Fresh fruit intake | rs2143081 | 6 | 50782834 | A | G | 0.54 | 0.008 | 0.001 | 1.29987e-11 | 446462 | 45.844 |
| Fresh fruit intake | rs216526 | 14 | 60650086 | G | A | 0.409 | -0.006 | 0.001 | 1.29999e-06 | 446462 | 23.427 |
| Fresh fruit intake | rs2189620 | 7 | 8792148 | C | T | 0.681 | -0.006 | 0.001 | 1.29999e-06 | 446462 | 23.387 |
| Fresh fruit intake | rs2254860 | 2 | 112995810 | G | A | 0.298 | -0.007 | 0.001 | 2.99999e-07 | 446462 | 26.222 |
| Fresh fruit intake | rs2425840 | 20 | 44904838 | C | A | 0.41 | 0.007 | 0.001 | 1.5e-07 | 446462 | 27.55 |
| Fresh fruit intake | rs2472297 | 15 | 75027880 | T | C | 0.261 | -0.007 | 0.001 | 1.89998e-07 | 446462 | 27.147 |
| Fresh fruit intake | rs2609858 | 3 | 146262021 | A | G | 0.174 | 0.008 | 0.002 | 2.30001e-06 | 446462 | 22.312 |
| Fresh fruit intake | rs2790688 | 1 | 153992909 | T | C | 0.154 | 0.011 | 0.002 | 1.50003e-11 | 446462 | 45.544 |
| Fresh fruit intake | rs28479795 | 14 | 79943606 | T | C | 0.221 | 0.011 | 0.001 | 2.49977e-14 | 446462 | 58.113 |
| Fresh fruit intake | rs2867113 | 2 | 651365 | A | G | 0.131 | -0.014 | 0.002 | 1.50003e-12 | 446462 | 49.996 |
| Fresh fruit intake | rs2899700 | 15 | 64937848 | G | A | 0.946 | -0.013 | 0.003 | 1.09999e-06 | 446462 | 23.828 |
| Fresh fruit intake | rs3110611 | 17 | 46752387 | T | C | 0.103 | 0.009 | 0.002 | 3.59998e-06 | 446462 | 21.487 |
| Fresh fruit intake | rs329274 | 7 | 35078743 | G | A | 0.486 | 0.007 | 0.001 | 2.80001e-08 | 446462 | 30.822 |
| Fresh fruit intake | rs34162196 | 14 | 22038125 | T | C | 0.101 | -0.018 | 0.002 | 4.00037e-19 | 446462 | 79.89 |
| Fresh fruit intake | rs34627141 | 2 | 171922537 | T | C | 0.355 | 0.006 | 0.001 | 4.49997e-07 | 446462 | 25.469 |
| Fresh fruit intake | rs3752712 | 7 | 1542124 | A | G | 0.405 | 0.006 | 0.001 | 4e-07 | 446462 | 25.704 |
| Fresh fruit intake | rs3768380 | 1 | 226849603 | G | A | 0.187 | 0.007 | 0.002 | 4.39997e-06 | 446462 | 21.092 |
| Fresh fruit intake | rs3806137 | 6 | 16519398 | G | A | 0.334 | 0.006 | 0.001 | 2.99999e-06 | 446462 | 21.813 |
| Fresh fruit intake | rs387722 | 6 | 92340664 | T | C | 0.601 | 0.006 | 0.001 | 1.2e-06 | 446462 | 23.561 |
| Fresh fruit intake | rs4302893 | 9 | 1734863 | A | G | 0.334 | 0.007 | 0.001 | 1.29999e-08 | 446462 | 32.323 |
| Fresh fruit intake | rs4513099 | 16 | 65597931 | G | A | 0.377 | 0.006 | 0.001 | 2.69998e-06 | 446462 | 22.04 |
| Fresh fruit intake | rs4585695 | 7 | 49625649 | C | T | 0.438 | 0.006 | 0.001 | 1.5e-07 | 446462 | 27.595 |
| Fresh fruit intake | rs4707358 | 6 | 87994572 | A | G | 0.527 | -0.006 | 0.001 | 1.2e-07 | 446462 | 28.05 |
| Fresh fruit intake | rs4708180 | 6 | 69275508 | A | G | 0.542 | -0.006 | 0.001 | 1.7e-07 | 446462 | 27.297 |
| Fresh fruit intake | rs4863749 | 4 | 138882529 | G | T | 0.543 | -0.006 | 0.001 | 6.90001e-07 | 446462 | 24.635 |
| Fresh fruit intake | rs4870866 | 8 | 124761727 | T | C | 0.116 | 0.009 | 0.002 | 3.79997e-06 | 446462 | 21.349 |
| Fresh fruit intake | rs4953150 | 2 | 45157336 | T | C | 0.344 | -0.008 | 0.001 | 6.59933e-11 | 446462 | 42.621 |
| Fresh fruit intake | rs55860703 | 2 | 59278195 | G | T | 0.098 | -0.01 | 0.002 | 4.09996e-06 | 446462 | 21.207 |
| Fresh fruit intake | rs56013513 | 5 | 101375195 | C | T | 0.075 | 0.012 | 0.002 | 3.29997e-07 | 446462 | 26.05 |
| Fresh fruit intake | rs56016114 | 20 | 31020883 | C | A | 0.009 | -0.032 | 0.007 | 1.09999e-06 | 446462 | 23.674 |
| Fresh fruit intake | rs5749227 | 22 | 31581166 | G | A | 0.756 | -0.008 | 0.001 | 7.90005e-08 | 446462 | 28.839 |
| Fresh fruit intake | rs586346 | 6 | 31875712 | C | T | 0.635 | -0.007 | 0.001 | 4.49997e-08 | 446462 | 29.9 |
| Fresh fruit intake | rs60452247 | 11 | 63981507 | A | G | 0.363 | 0.008 | 0.001 | 3.40001e-10 | 446462 | 39.422 |
| Fresh fruit intake | rs6137015 | 20 | 20369855 | A | G | 0.525 | -0.006 | 0.001 | 2.59998e-07 | 446462 | 26.49 |
| Fresh fruit intake | rs62109907 | 19 | 13210620 | T | C | 0.037 | 0.015 | 0.003 | 4.90004e-06 | 446462 | 20.859 |
| Fresh fruit intake | rs62334820 | 4 | 176855221 | T | C | 0.201 | -0.007 | 0.002 | 4.49997e-06 | 446462 | 21.047 |
| Fresh fruit intake | rs62420599 | 6 | 98780531 | C | T | 0.505 | -0.007 | 0.001 | 9.20005e-08 | 446462 | 28.531 |
| Fresh fruit intake | rs642544 | 11 | 105737020 | G | T | 0.414 | 0.006 | 0.001 | 4.20001e-06 | 446462 | 21.162 |
| Fresh fruit intake | rs6475724 | 9 | 23274223 | T | C | 0.727 | 0.008 | 0.001 | 1.89998e-08 | 446462 | 31.579 |
| Fresh fruit intake | rs6477901 | 9 | 114992714 | T | C | 0.779 | -0.008 | 0.001 | 1.89998e-07 | 446462 | 27.175 |
| Fresh fruit intake | rs67056409 | 6 | 35383699 | G | A | 0.191 | -0.007 | 0.002 | 3.29997e-06 | 446462 | 21.62 |
| Fresh fruit intake | rs67250895 | 5 | 2303160 | G | A | 0.155 | -0.008 | 0.002 | 7.10003e-07 | 446462 | 24.583 |
| Fresh fruit intake | rs6758494 | 2 | 172780086 | C | T | 0.129 | 0.009 | 0.002 | 1e-06 | 446462 | 23.838 |
| Fresh fruit intake | rs67667422 | 20 | 11893242 | G | T | 0.355 | -0.006 | 0.001 | 2.90001e-06 | 446462 | 21.878 |
| Fresh fruit intake | rs7014421 | 8 | 25675709 | G | T | 0.372 | 0.006 | 0.001 | 2.69998e-06 | 446462 | 22.001 |
| Fresh fruit intake | rs7124442 | 11 | 27677041 | T | C | 0.679 | -0.006 | 0.001 | 4.39997e-06 | 446462 | 21.08 |
| Fresh fruit intake | rs71320537 | 21 | 44088683 | T | C | 0.087 | 0.01 | 0.002 | 2.59998e-06 | 446462 | 22.067 |
| Fresh fruit intake | rs7256200 | 19 | 45415935 | T | G | 0.127 | 0.009 | 0.002 | 2e-06 | 446462 | 22.559 |
| Fresh fruit intake | rs72897648 | 2 | 188728752 | T | C | 0.137 | -0.008 | 0.002 | 1.89998e-06 | 446462 | 22.691 |
| Fresh fruit intake | rs7313901 | 12 | 51924030 | C | T | 0.142 | -0.009 | 0.002 | 3.29997e-07 | 446462 | 26.079 |
| Fresh fruit intake | rs739320 | 19 | 49261368 | C | T | 0.606 | -0.009 | 0.001 | 1.9002e-12 | 446462 | 49.635 |
| Fresh fruit intake | rs74976306 | 5 | 141302226 | A | G | 0.058 | -0.012 | 0.003 | 4.70002e-06 | 446462 | 20.96 |
| Fresh fruit intake | rs7554485 | 1 | 65945906 | C | T | 0.612 | -0.008 | 0.001 | 1.7e-10 | 446462 | 40.793 |
| Fresh fruit intake | rs7555067 | 1 | 174266642 | T | C | 0.451 | 0.006 | 0.001 | 2.5e-06 | 446462 | 22.138 |
| Fresh fruit intake | rs7579662 | 2 | 161915810 | A | G | 0.393 | -0.006 | 0.001 | 2.39999e-06 | 446462 | 22.21 |
| Fresh fruit intake | rs7703932 | 5 | 68044531 | T | C | 0.478 | -0.006 | 0.001 | 2.5e-06 | 446462 | 22.195 |
| Fresh fruit intake | rs7778914 | 7 | 4989005 | A | G | 0.895 | 0.01 | 0.002 | 6.69993e-07 | 446462 | 24.703 |
| Fresh fruit intake | rs7818437 | 8 | 10209623 | C | T | 0.236 | -0.008 | 0.001 | 2.99999e-08 | 446462 | 30.701 |
| Fresh fruit intake | rs7864529 | 9 | 128501879 | C | A | 0.633 | -0.006 | 0.001 | 3.59998e-06 | 446462 | 21.445 |
| Fresh fruit intake | rs7869969 | 9 | 96217447 | G | A | 0.331 | 0.008 | 0.001 | 5.69994e-09 | 446462 | 33.927 |
| Fresh fruit intake | rs7982441 | 13 | 55924013 | C | T | 0.732 | -0.008 | 0.001 | 9.80009e-10 | 446462 | 37.371 |
| Fresh fruit intake | rs8095324 | 18 | 24131659 | G | A | 0.404 | -0.007 | 0.001 | 2.69998e-08 | 446462 | 30.927 |
| Fresh fruit intake | rs817223 | 2 | 104094008 | C | T | 0.481 | -0.007 | 0.001 | 2.80001e-09 | 446462 | 35.304 |
| Fresh fruit intake | rs862227 | 16 | 73602926 | G | A | 0.458 | -0.01 | 0.001 | 1.10002e-16 | 446462 | 68.702 |
| Fresh fruit intake | rs898751 | 17 | 2291863 | T | C | 0.493 | -0.006 | 0.001 | 2e-06 | 446462 | 22.607 |
| Fresh fruit intake | rs9320934 | 6 | 123758414 | A | G | 0.082 | 0.011 | 0.002 | 1.79999e-06 | 446462 | 22.819 |
| Fresh fruit intake | rs9347904 | 6 | 158453211 | T | C | 0.365 | 0.006 | 0.001 | 5.69994e-07 | 446462 | 25 |
| Fresh fruit intake | rs938914 | 3 | 116309057 | C | T | 0.456 | 0.006 | 0.001 | 4.79999e-07 | 446462 | 25.342 |
| Fresh fruit intake | rs9517948 | 13 | 100650708 | T | C | 0.451 | 0.007 | 0.001 | 1.7e-08 | 446462 | 31.807 |
| Fresh fruit intake | rs9635715 | 17 | 42517354 | C | T | 0.449 | 0.007 | 0.001 | 7.39997e-08 | 446462 | 28.945 |
| Fresh fruit intake | rs9919429 | 10 | 65313819 | G | A | 0.486 | -0.007 | 0.001 | 3.79997e-08 | 446462 | 30.226 |
| Dried fruit intake | rs1000214 | 8 | 18053598 | G | T | 0.84 | -0.012 | 0.002 | 2.19999e-07 | 421764 | 26.855 |
| Dried fruit intake | rs10026792 | 4 | 2862190 | A | G | 0.29 | 0.011 | 0.002 | 3.89996e-09 | 421764 | 34.662 |
| Dried fruit intake | rs10040819 | 5 | 147964658 | A | C | 0.149 | 0.011 | 0.002 | 1.6e-06 | 421764 | 23.061 |
| Dried fruit intake | rs10087278 | 8 | 86314855 | A | G | 0.591 | 0.009 | 0.002 | 1.29999e-07 | 421764 | 27.922 |
| Dried fruit intake | rs10129747 | 14 | 77433198 | G | A | 0.53 | 0.009 | 0.002 | 2.59998e-08 | 421764 | 30.988 |
| Dried fruit intake | rs10156491 | 9 | 82886585 | A | G | 0.374 | 0.009 | 0.002 | 1.2e-07 | 421764 | 28.076 |
| Dried fruit intake | rs10178071 | 2 | 49417986 | T | C | 0.521 | 0.009 | 0.002 | 7.19996e-08 | 421764 | 29.019 |
| Dried fruit intake | rs10192800 | 2 | 72502270 | A | G | 0.343 | 0.009 | 0.002 | 1.29999e-06 | 421764 | 23.415 |
| Dried fruit intake | rs10262162 | 7 | 44389625 | A | G | 0.18 | 0.01 | 0.002 | 2.69998e-06 | 421764 | 22.003 |
| Dried fruit intake | rs10268994 | 7 | 21892846 | A | G | 0.664 | -0.008 | 0.002 | 2.90001e-06 | 421764 | 21.881 |
| Dried fruit intake | rs10896126 | 11 | 66292908 | G | A | 0.304 | -0.015 | 0.002 | 1.59993e-16 | 421764 | 68.068 |
| Dried fruit intake | rs11073575 | 15 | 98314128 | C | T | 0.58 | -0.009 | 0.002 | 1.79999e-07 | 421764 | 27.28 |
| Dried fruit intake | rs11082011 | 18 | 35145122 | T | C | 0.668 | 0.009 | 0.002 | 2.5e-07 | 421764 | 26.568 |
| Dried fruit intake | rs11085749 | 19 | 10961273 | A | G | 0.387 | -0.009 | 0.002 | 6.1e-08 | 421764 | 29.334 |
| Dried fruit intake | rs11134477 | 5 | 167314580 | A | G | 0.554 | 0.008 | 0.002 | 2.59998e-06 | 421764 | 22.125 |
| Dried fruit intake | rs11152349 | 18 | 60233646 | A | G | 0.303 | 0.01 | 0.002 | 4.90004e-08 | 421764 | 29.74 |
| Dried fruit intake | rs11253371 | 10 | 774516 | G | A | 0.307 | -0.009 | 0.002 | 2.90001e-06 | 421764 | 21.884 |
| Dried fruit intake | rs112887546 | 6 | 84113685 | A | C | 0.193 | -0.011 | 0.002 | 4.49997e-07 | 421764 | 25.464 |
| Dried fruit intake | rs115141576 | 4 | 37346857 | T | C | 0.024 | -0.026 | 0.005 | 1.5e-06 | 421764 | 23.111 |
| Dried fruit intake | rs116385168 | 3 | 115037579 | C | T | 0.06 | 0.019 | 0.004 | 6.90001e-08 | 421764 | 29.094 |
| Dried fruit intake | rs11692435 | 2 | 98275354 | A | G | 0.083 | -0.017 | 0.003 | 7.90005e-08 | 421764 | 28.823 |
| Dried fruit intake | rs117451379 | 7 | 102389213 | T | C | 0.069 | 0.016 | 0.003 | 3.2e-06 | 421764 | 21.68 |
| Dried fruit intake | rs12137234 | 1 | 72270797 | T | C | 0.304 | 0.01 | 0.002 | 2.80001e-08 | 421764 | 30.847 |
| Dried fruit intake | rs12462942 | 19 | 30110805 | C | T | 0.539 | -0.008 | 0.002 | 1.6e-06 | 421764 | 23.028 |
| Dried fruit intake | rs12516172 | 5 | 125564863 | T | C | 0.022 | 0.027 | 0.006 | 4.90004e-06 | 421764 | 20.868 |
| Dried fruit intake | rs12600070 | 16 | 82842132 | A | C | 0.102 | 0.014 | 0.003 | 1e-06 | 421764 | 23.9 |
| Dried fruit intake | rs12708665 | 16 | 24728227 | G | A | 0.715 | -0.009 | 0.002 | 4.39997e-06 | 421764 | 21.09 |
| Dried fruit intake | rs12727104 | 1 | 171423167 | A | G | 0.094 | -0.013 | 0.003 | 4.70002e-06 | 421764 | 20.973 |
| Dried fruit intake | rs12797895 | 11 | 79883835 | C | T | 0.52 | 0.008 | 0.002 | 7.79992e-07 | 421764 | 24.399 |
| Dried fruit intake | rs1289870 | 1 | 117864047 | T | C | 0.576 | 0.009 | 0.002 | 4.60002e-07 | 421764 | 25.411 |
| Dried fruit intake | rs13016580 | 2 | 172646528 | A | G | 0.32 | -0.009 | 0.002 | 8.79995e-07 | 421764 | 24.164 |
| Dried fruit intake | rs13018443 | 2 | 170944488 | C | T | 0.366 | -0.009 | 0.002 | 3.40001e-07 | 421764 | 25.993 |
| Dried fruit intake | rs13029034 | 2 | 213196561 | A | G | 0.056 | 0.017 | 0.004 | 2.19999e-06 | 421764 | 22.372 |
| Dried fruit intake | rs13071694 | 3 | 177492986 | A | G | 0.278 | 0.009 | 0.002 | 4.70002e-06 | 421764 | 20.956 |
| Dried fruit intake | rs13111442 | 4 | 57815766 | C | A | 0.654 | -0.008 | 0.002 | 1.6e-06 | 421764 | 23.006 |
| Dried fruit intake | rs13361944 | 5 | 154771346 | C | T | 0.091 | 0.014 | 0.003 | 2.69998e-06 | 421764 | 22.021 |
| Dried fruit intake | rs1419750 | 7 | 111329102 | G | A | 0.677 | -0.009 | 0.002 | 2e-07 | 421764 | 27.078 |
| Dried fruit intake | rs142119647 | 1 | 17604875 | G | A | 0.026 | 0.025 | 0.005 | 2.1e-06 | 421764 | 22.468 |
| Dried fruit intake | rs149367378 | 1 | 32415997 | T | G | 0.037 | -0.022 | 0.005 | 2.30001e-06 | 421764 | 22.302 |
| Dried fruit intake | rs1549212 | 5 | 166996722 | T | C | 0.626 | 0.009 | 0.002 | 7.49998e-07 | 421764 | 24.488 |
| Dried fruit intake | rs1560525 | 13 | 47700334 | G | T | 0.563 | -0.008 | 0.002 | 4.90004e-06 | 421764 | 20.89 |
| Dried fruit intake | rs1582322 | 16 | 52105988 | G | A | 0.605 | 0.01 | 0.002 | 6.80002e-09 | 421764 | 33.588 |
| Dried fruit intake | rs1622515 | 11 | 95523433 | G | A | 0.485 | 0.01 | 0.002 | 2.90001e-09 | 421764 | 35.232 |
| Dried fruit intake | rs1648404 | 4 | 37175523 | T | C | 0.476 | 0.009 | 0.002 | 1.79999e-08 | 421764 | 31.655 |
| Dried fruit intake | rs17175518 | 18 | 57850583 | A | C | 0.233 | 0.011 | 0.002 | 5.89997e-09 | 421764 | 33.877 |
| Dried fruit intake | rs17184650 | 14 | 67939796 | T | C | 0.122 | -0.012 | 0.003 | 2.80001e-06 | 421764 | 21.921 |
| Dried fruit intake | rs17184707 | 2 | 166183577 | T | C | 0.213 | -0.011 | 0.002 | 2.1e-08 | 421764 | 31.434 |
| Dried fruit intake | rs1735720 | 21 | 23077546 | G | T | 0.192 | -0.011 | 0.002 | 4.39997e-07 | 421764 | 25.502 |
| Dried fruit intake | rs17592481 | 19 | 32157499 | T | G | 0.165 | -0.01 | 0.002 | 3.2e-06 | 421764 | 21.666 |
| Dried fruit intake | rs1801140 | 20 | 10625804 | G | T | 0.098 | 0.013 | 0.003 | 1.7e-06 | 421764 | 22.862 |
| Dried fruit intake | rs1812195 | 17 | 25576289 | G | A | 0.401 | 0.009 | 0.002 | 7.39997e-07 | 421764 | 24.519 |
| Dried fruit intake | rs193003548 | 1 | 151371547 | T | C | 0.015 | -0.036 | 0.007 | 5.19996e-07 | 421764 | 25.184 |
| Dried fruit intake | rs1950826 | 14 | 38039969 | T | C | 0.59 | 0.008 | 0.002 | 1.79999e-06 | 421764 | 22.75 |
| Dried fruit intake | rs2165150 | 2 | 188879205 | A | C | 0.744 | 0.01 | 0.002 | 1.79999e-07 | 421764 | 27.247 |
| Dried fruit intake | rs2235792 | 1 | 6156915 | A | G | 0.108 | 0.013 | 0.003 | 6.19998e-07 | 421764 | 24.844 |
| Dried fruit intake | rs2328887 | 6 | 25430149 | C | T | 0.899 | 0.019 | 0.003 | 8.80035e-12 | 421764 | 46.588 |
| Dried fruit intake | rs2353857 | 14 | 62444178 | A | C | 0.476 | -0.008 | 0.002 | 2e-06 | 421764 | 22.579 |
| Dried fruit intake | rs2389238 | 13 | 96058183 | G | A | 0.886 | 0.013 | 0.003 | 1.5e-06 | 421764 | 23.091 |
| Dried fruit intake | rs2508819 | 11 | 115693410 | C | T | 0.462 | 0.008 | 0.002 | 1.09999e-06 | 421764 | 23.692 |
| Dried fruit intake | rs2533273 | 7 | 153485282 | A | C | 0.485 | -0.01 | 0.002 | 3.89996e-09 | 421764 | 34.68 |
| Dried fruit intake | rs261809 | 1 | 241054465 | G | A | 0.541 | -0.01 | 0.002 | 9.80009e-09 | 421764 | 32.886 |
| Dried fruit intake | rs3101339 | 1 | 72748669 | C | A | 0.603 | 0.014 | 0.002 | 6.20012e-17 | 421764 | 69.907 |
| Dried fruit intake | rs322672 | 3 | 25332732 | C | T | 0.351 | 0.008 | 0.002 | 2.90001e-06 | 421764 | 21.871 |
| Dried fruit intake | rs34162196 | 14 | 22038125 | T | C | 0.101 | -0.022 | 0.003 | 7.10068e-16 | 421764 | 65.097 |
| Dried fruit intake | rs34419562 | 16 | 51208161 | A | G | 0.095 | -0.014 | 0.003 | 1.6e-06 | 421764 | 23.006 |
| Dried fruit intake | rs34819186 | 8 | 143363229 | C | A | 0.472 | -0.008 | 0.002 | 1.7e-06 | 421764 | 22.893 |
| Dried fruit intake | rs35456421 | 2 | 28288020 | T | C | 0.109 | -0.013 | 0.003 | 7.79992e-07 | 421764 | 24.415 |
| Dried fruit intake | rs35792972 | 3 | 2568403 | G | A | 0.173 | 0.01 | 0.002 | 4.90004e-06 | 421764 | 20.894 |
| Dried fruit intake | rs36198109 | 5 | 60559156 | A | G | 0.384 | -0.009 | 0.002 | 8.9e-08 | 421764 | 28.589 |
| Dried fruit intake | rs3764002 | 12 | 108618630 | T | C | 0.261 | 0.013 | 0.002 | 5.10035e-12 | 421764 | 47.653 |
| Dried fruit intake | rs3796183 | 3 | 35684594 | C | T | 0.21 | -0.01 | 0.002 | 2.69998e-06 | 421764 | 22.048 |
| Dried fruit intake | rs3819391 | 4 | 55526702 | G | A | 0.559 | 0.008 | 0.002 | 2.1e-06 | 421764 | 22.469 |
| Dried fruit intake | rs4073960 | 6 | 56548359 | A | G | 0.228 | -0.011 | 0.002 | 6.59994e-08 | 421764 | 29.187 |
| Dried fruit intake | rs4140799 | 14 | 72170969 | A | G | 0.532 | 0.009 | 0.002 | 1.79999e-08 | 421764 | 31.743 |
| Dried fruit intake | rs4149513 | 2 | 101022726 | A | G | 0.494 | 0.012 | 0.002 | 2.19989e-12 | 421764 | 49.254 |
| Dried fruit intake | rs4269101 | 3 | 18763543 | G | T | 0.719 | -0.014 | 0.002 | 1.10002e-13 | 421764 | 55.172 |
| Dried fruit intake | rs429358 | 19 | 45411941 | C | T | 0.154 | 0.02 | 0.002 | 6.70039e-18 | 421764 | 74.312 |
| Dried fruit intake | rs4740634 | 9 | 1666496 | G | A | 0.331 | 0.009 | 0.002 | 2e-07 | 421764 | 27.04 |
| Dried fruit intake | rs4769824 | 13 | 30883339 | T | C | 0.437 | 0.008 | 0.002 | 2.59998e-06 | 421764 | 22.092 |
| Dried fruit intake | rs4800488 | 18 | 21117571 | A | C | 0.49 | 0.012 | 0.002 | 7.70016e-13 | 421764 | 51.368 |
| Dried fruit intake | rs4808846 | 19 | 18814330 | G | A | 0.481 | 0.009 | 0.002 | 2.59998e-07 | 421764 | 26.515 |
| Dried fruit intake | rs4866437 | 5 | 3226096 | A | G | 0.531 | 0.008 | 0.002 | 8.70001e-07 | 421764 | 24.188 |
| Dried fruit intake | rs4963390 | 11 | 61514821 | G | A | 0.139 | 0.013 | 0.002 | 7.79992e-08 | 421764 | 28.859 |
| Dried fruit intake | rs565226 | 3 | 59788525 | G | T | 0.885 | 0.013 | 0.003 | 1e-06 | 421764 | 23.897 |
| Dried fruit intake | rs57499472 | 3 | 147239337 | C | T | 0.404 | 0.01 | 0.002 | 8.10009e-09 | 421764 | 33.262 |
| Dried fruit intake | rs58563230 | 2 | 115835313 | G | A | 0.114 | 0.013 | 0.003 | 1.7e-06 | 421764 | 22.857 |
| Dried fruit intake | rs597941 | 1 | 57757524 | T | C | 0.374 | 0.009 | 0.002 | 9.40005e-08 | 421764 | 28.494 |
| Dried fruit intake | rs60574043 | 3 | 89095401 | T | C | 0.117 | -0.014 | 0.003 | 1.89998e-07 | 421764 | 27.11 |
| Dried fruit intake | rs6126123 | 20 | 49522796 | G | A | 0.272 | 0.009 | 0.002 | 1.40001e-06 | 421764 | 23.235 |
| Dried fruit intake | rs62007687 | 14 | 103903763 | T | G | 0.344 | 0.009 | 0.002 | 4.70002e-07 | 421764 | 25.372 |
| Dried fruit intake | rs62023502 | 15 | 53107255 | A | G | 0.098 | -0.013 | 0.003 | 4.39997e-06 | 421764 | 21.062 |
| Dried fruit intake | rs62057232 | 16 | 30820866 | C | T | 0.39 | 0.008 | 0.002 | 2.99999e-06 | 421764 | 21.787 |
| Dried fruit intake | rs62162747 | 2 | 85638865 | C | T | 0.334 | -0.009 | 0.002 | 2.30001e-07 | 421764 | 26.75 |
| Dried fruit intake | rs6438468 | 3 | 118390575 | A | C | 0.687 | -0.008 | 0.002 | 3.09999e-06 | 421764 | 21.766 |
| Dried fruit intake | rs6533183 | 4 | 106133184 | T | C | 0.657 | -0.01 | 0.002 | 5.80003e-08 | 421764 | 29.42 |
| Dried fruit intake | rs6712271 | 2 | 207918695 | A | G | 0.398 | -0.008 | 0.002 | 1.6e-06 | 421764 | 22.971 |
| Dried fruit intake | rs67518212 | 14 | 20648951 | C | T | 0.213 | 0.011 | 0.002 | 8.40001e-08 | 421764 | 28.712 |
| Dried fruit intake | rs6765212 | 3 | 43931484 | T | C | 0.271 | 0.011 | 0.002 | 5.60003e-09 | 421764 | 33.982 |
| Dried fruit intake | rs7045666 | 9 | 87252243 | A | G | 0.457 | -0.008 | 0.002 | 1e-06 | 421764 | 23.905 |
| Dried fruit intake | rs7047847 | 9 | 115089045 | T | C | 0.779 | -0.011 | 0.002 | 1.29999e-07 | 421764 | 27.88 |
| Dried fruit intake | rs7119021 | 11 | 44868967 | A | G | 0.025 | 0.028 | 0.005 | 1.2e-07 | 421764 | 27.965 |
| Dried fruit intake | rs7203102 | 16 | 7247154 | T | C | 0.631 | -0.008 | 0.002 | 1.2e-06 | 421764 | 23.504 |
| Dried fruit intake | rs72720396 | 1 | 91191582 | G | A | 0.229 | 0.011 | 0.002 | 8.70001e-09 | 421764 | 33.123 |
| Dried fruit intake | rs72784460 | 5 | 92608809 | T | C | 0.16 | -0.012 | 0.002 | 4.90004e-07 | 421764 | 25.317 |
| Dried fruit intake | rs72842424 | 11 | 1041967 | A | C | 0.224 | -0.01 | 0.002 | 5.1e-07 | 421764 | 25.231 |
| Dried fruit intake | rs7334175 | 13 | 112215941 | T | C | 0.707 | -0.009 | 0.002 | 2.39999e-06 | 421764 | 22.239 |
| Dried fruit intake | rs742877 | 14 | 97460465 | A | C | 0.122 | 0.013 | 0.003 | 6.1e-07 | 421764 | 24.878 |
| Dried fruit intake | rs74611672 | 18 | 23200504 | C | T | 0.047 | 0.019 | 0.004 | 2e-06 | 421764 | 22.574 |
| Dried fruit intake | rs75265668 | 20 | 48824878 | A | G | 0.018 | -0.031 | 0.007 | 3.09999e-06 | 421764 | 21.754 |
| Dried fruit intake | rs75641275 | 1 | 98327133 | C | A | 0.143 | -0.014 | 0.002 | 2.90001e-09 | 421764 | 35.25 |
| Dried fruit intake | rs7582086 | 2 | 60231826 | T | G | 0.468 | -0.01 | 0.002 | 8.79995e-09 | 421764 | 33.088 |
| Dried fruit intake | rs7599488 | 2 | 60718347 | T | C | 0.426 | -0.01 | 0.002 | 6.69993e-10 | 421764 | 38.104 |
| Dried fruit intake | rs76352965 | 6 | 88212666 | C | T | 0.039 | -0.021 | 0.004 | 9.40005e-07 | 421764 | 24.05 |
| Dried fruit intake | rs7730004 | 5 | 43191033 | T | C | 0.678 | -0.008 | 0.002 | 4.60002e-06 | 421764 | 21.004 |
| Dried fruit intake | rs7742849 | 6 | 62639359 | A | G | 0.342 | -0.008 | 0.002 | 1.79999e-06 | 421764 | 22.802 |
| Dried fruit intake | rs7749708 | 6 | 153375907 | T | C | 0.294 | -0.009 | 0.002 | 1.2e-06 | 421764 | 23.65 |
| Dried fruit intake | rs7808471 | 7 | 132716502 | C | T | 0.322 | -0.012 | 0.002 | 1.09999e-10 | 421764 | 41.72 |
| Dried fruit intake | rs7810473 | 7 | 136596457 | G | A | 0.419 | 0.008 | 0.002 | 2.99999e-06 | 421764 | 21.844 |
| Dried fruit intake | rs7829800 | 8 | 144258705 | G | A | 0.671 | -0.01 | 0.002 | 5.1e-09 | 421764 | 34.169 |
| Dried fruit intake | rs7835197 | 8 | 31982676 | C | T | 0.881 | 0.013 | 0.003 | 5.99998e-07 | 421764 | 24.92 |
| Dried fruit intake | rs7923809 | 10 | 87857822 | C | T | 0.078 | -0.016 | 0.003 | 1.5e-07 | 421764 | 27.646 |
| Dried fruit intake | rs7941325 | 11 | 28466083 | A | G | 0.291 | -0.009 | 0.002 | 1.6e-06 | 421764 | 23.025 |
| Dried fruit intake | rs8081370 | 17 | 1373612 | T | C | 0.91 | -0.017 | 0.003 | 1.40001e-08 | 421764 | 32.181 |
| Dried fruit intake | rs830605 | 3 | 71609276 | T | C | 0.495 | -0.008 | 0.002 | 3.79997e-06 | 421764 | 21.362 |
| Dried fruit intake | rs862227 | 16 | 73602926 | G | A | 0.458 | -0.009 | 0.002 | 4.30002e-08 | 421764 | 30.03 |
| Dried fruit intake | rs893856 | 10 | 126723567 | A | G | 0.149 | -0.013 | 0.002 | 1.29999e-08 | 421764 | 32.346 |
| Dried fruit intake | rs898751 | 17 | 2291863 | T | C | 0.493 | -0.009 | 0.002 | 8.40001e-08 | 421764 | 28.714 |
| Dried fruit intake | rs907237 | 4 | 152601146 | G | T | 0.49 | 0.008 | 0.002 | 1.7e-06 | 421764 | 22.878 |
| Dried fruit intake | rs9298741 | 9 | 15837531 | G | A | 0.51 | -0.008 | 0.002 | 2.90001e-06 | 421764 | 21.852 |
| Dried fruit intake | rs9300104 | 11 | 10551106 | C | T | 0.65 | -0.008 | 0.002 | 4.39997e-06 | 421764 | 21.063 |
| Dried fruit intake | rs9310545 | 3 | 18197008 | C | A | 0.516 | -0.009 | 0.002 | 1.5e-07 | 421764 | 27.607 |
| Dried fruit intake | rs9385269 | 6 | 98547979 | T | C | 0.525 | 0.012 | 0.002 | 7.19946e-13 | 421764 | 51.482 |
| Dried fruit intake | rs9527691 | 13 | 58322621 | A | G | 0.264 | -0.01 | 0.002 | 3.59998e-07 | 421764 | 25.886 |
| Dried fruit intake | rs954212 | 1 | 154659342 | G | A | 0.177 | 0.011 | 0.002 | 5.19996e-07 | 421764 | 25.171 |
| Dried fruit intake | rs9609271 | 22 | 31775982 | T | G | 0.288 | 0.009 | 0.002 | 3.89996e-07 | 421764 | 25.738 |
| Dried fruit intake | rs979678 | 12 | 73309402 | G | A | 0.218 | 0.011 | 0.002 | 7.59994e-08 | 421764 | 28.908 |
| Dried fruit intake | rs982671 | 16 | 64365361 | A | G | 0.258 | -0.009 | 0.002 | 2.80001e-06 | 421764 | 21.969 |
| Dried fruit intake | rs9860336 | 3 | 188879596 | T | C | 0.266 | -0.009 | 0.002 | 2.30001e-06 | 421764 | 22.323 |
| Cooked vegetable intake | rs10129582 | 14 | 99704427 | G | A | 0.433 | -0.009 | 0.002 | 6.1e-08 | 448651 | 29.321 |
| Cooked vegetable intake | rs10156602 | 9 | 96345328 | G | A | 0.361 | 0.011 | 0.002 | 1.80011e-11 | 448651 | 45.212 |
| Cooked vegetable intake | rs10161952 | 13 | 59474383 | C | A | 0.313 | -0.01 | 0.002 | 1.29999e-08 | 448651 | 32.289 |
| Cooked vegetable intake | rs10248006 | 7 | 126034791 | A | G | 0.687 | -0.008 | 0.002 | 3.50002e-06 | 448651 | 21.545 |
| Cooked vegetable intake | rs11016841 | 10 | 129026189 | G | A | 0.151 | 0.011 | 0.002 | 8.50002e-07 | 448651 | 24.246 |
| Cooked vegetable intake | rs111608385 | 3 | 108119473 | T | C | 0.189 | -0.009 | 0.002 | 4.49997e-06 | 448651 | 21.038 |
| Cooked vegetable intake | rs11162019 | 1 | 87913176 | T | C | 0.362 | -0.008 | 0.002 | 1.79999e-06 | 448651 | 22.804 |
| Cooked vegetable intake | rs113993820 | 2 | 102766634 | T | G | 0.019 | -0.028 | 0.006 | 8.40001e-07 | 448651 | 24.253 |
| Cooked vegetable intake | rs114380720 | 2 | 157475464 | A | G | 0.027 | -0.024 | 0.005 | 6.49995e-07 | 448651 | 24.755 |
| Cooked vegetable intake | rs115403726 | 3 | 139439220 | T | C | 0.021 | -0.028 | 0.006 | 4.09996e-07 | 448651 | 25.629 |
| Cooked vegetable intake | rs117024221 | 8 | 82846017 | A | C | 0.011 | 0.036 | 0.008 | 2.30001e-06 | 448651 | 22.318 |
| Cooked vegetable intake | rs11710570 | 3 | 158056654 | C | T | 0.448 | -0.008 | 0.002 | 4.30002e-07 | 448651 | 25.572 |
| Cooked vegetable intake | rs11924675 | 3 | 74768098 | T | G | 0.489 | 0.008 | 0.002 | 9.40005e-08 | 448651 | 28.485 |
| Cooked vegetable intake | rs12550717 | 8 | 10266706 | A | G | 0.372 | 0.009 | 0.002 | 1.40001e-08 | 448651 | 32.203 |
| Cooked vegetable intake | rs12629972 | 3 | 25121444 | C | T | 0.588 | 0.012 | 0.002 | 1.20005e-13 | 448651 | 55.017 |
| Cooked vegetable intake | rs12642480 | 4 | 188980863 | C | T | 0.648 | 0.008 | 0.002 | 2.39999e-06 | 448651 | 22.269 |
| Cooked vegetable intake | rs12682588 | 8 | 27889972 | C | T | 0.474 | -0.008 | 0.002 | 3.2e-07 | 448651 | 26.101 |
| Cooked vegetable intake | rs13346101 | 19 | 30012512 | A | G | 0.136 | 0.011 | 0.002 | 2.30001e-06 | 448651 | 22.289 |
| Cooked vegetable intake | rs1352243 | 5 | 113435505 | A | G | 0.156 | -0.011 | 0.002 | 6.4e-07 | 448651 | 24.782 |
| Cooked vegetable intake | rs140868590 | 5 | 5948588 | C | T | 0.016 | 0.031 | 0.007 | 2.59998e-06 | 448651 | 22.062 |
| Cooked vegetable intake | rs1421085 | 16 | 53800954 | C | T | 0.403 | 0.01 | 0.002 | 8.30042e-11 | 448651 | 42.193 |
| Cooked vegetable intake | rs1444405 | 15 | 63544106 | A | G | 0.36 | -0.008 | 0.002 | 1.09999e-06 | 448651 | 23.7 |
| Cooked vegetable intake | rs150671711 | 3 | 136844366 | T | C | 0.01 | 0.037 | 0.008 | 4.09996e-06 | 448651 | 21.207 |
| Cooked vegetable intake | rs1534749 | 1 | 190028576 | C | T | 0.53 | -0.007 | 0.002 | 2.69998e-06 | 448651 | 22.054 |
| Cooked vegetable intake | rs1541913 | 5 | 164685326 | T | C | 0.299 | -0.008 | 0.002 | 2.30001e-06 | 448651 | 22.326 |
| Cooked vegetable intake | rs16996551 | 20 | 15773571 | T | C | 0.379 | -0.009 | 0.002 | 7.19996e-08 | 448651 | 29 |
| Cooked vegetable intake | rs17653477 | 3 | 71170319 | G | A | 0.03 | -0.022 | 0.005 | 8.70001e-07 | 448651 | 24.198 |
| Cooked vegetable intake | rs17784909 | 6 | 165748148 | G | A | 0.212 | 0.009 | 0.002 | 1.7e-06 | 448651 | 22.893 |
| Cooked vegetable intake | rs1794191 | 11 | 123407544 | G | T | 0.964 | 0.02 | 0.004 | 3.2e-06 | 448651 | 21.69 |
| Cooked vegetable intake | rs1816263 | 5 | 141217079 | C | T | 0.28 | 0.01 | 0.002 | 3.69999e-08 | 448651 | 30.299 |
| Cooked vegetable intake | rs1875517 | 3 | 117307567 | A | G | 0.429 | -0.008 | 0.002 | 7.90005e-07 | 448651 | 24.372 |
| Cooked vegetable intake | rs2033597 | 1 | 213549960 | C | T | 0.611 | 0.008 | 0.002 | 3.09999e-07 | 448651 | 26.16 |
| Cooked vegetable intake | rs2052063 | 10 | 118040352 | T | C | 0.516 | -0.009 | 0.002 | 1.6e-09 | 448651 | 36.385 |
| Cooked vegetable intake | rs2102738 | 2 | 172525884 | C | A | 0.172 | -0.012 | 0.002 | 5.30005e-09 | 448651 | 34.066 |
| Cooked vegetable intake | rs2222134 | 2 | 18518590 | T | C | 0.983 | 0.03 | 0.006 | 3.2e-06 | 448651 | 21.673 |
| Cooked vegetable intake | rs2252508 | 1 | 153913770 | G | A | 0.48 | 0.009 | 0.002 | 5.69994e-09 | 448651 | 33.949 |
| Cooked vegetable intake | rs2271424 | 1 | 204403311 | C | T | 0.15 | 0.012 | 0.002 | 8.30004e-08 | 448651 | 28.744 |
| Cooked vegetable intake | rs2339744 | 5 | 172450020 | A | G | 0.259 | 0.009 | 0.002 | 1.29999e-06 | 448651 | 23.388 |
| Cooked vegetable intake | rs264820 | 8 | 78376182 | T | G | 0.062 | 0.015 | 0.003 | 2.19999e-06 | 448651 | 22.382 |
| Cooked vegetable intake | rs2746089 | 20 | 34646012 | C | T | 0.867 | 0.012 | 0.002 | 1.5e-07 | 448651 | 27.62 |
| Cooked vegetable intake | rs28420942 | 15 | 48009488 | A | G | 0.229 | 0.009 | 0.002 | 7.00003e-07 | 448651 | 24.615 |
| Cooked vegetable intake | rs28437380 | 8 | 113926117 | G | A | 0.337 | -0.008 | 0.002 | 2.80001e-07 | 448651 | 26.399 |
| Cooked vegetable intake | rs2844672 | 6 | 31005139 | A | G | 0.624 | -0.01 | 0.002 | 2.1e-09 | 448651 | 35.901 |
| Cooked vegetable intake | rs2844793 | 6 | 30080496 | A | G | 0.171 | -0.01 | 0.002 | 2.39999e-06 | 448651 | 22.238 |
| Cooked vegetable intake | rs28450747 | 4 | 42137653 | A | G | 0.233 | -0.01 | 0.002 | 4.30002e-08 | 448651 | 30.01 |
| Cooked vegetable intake | rs28711392 | 11 | 13349559 | C | T | 0.367 | -0.011 | 0.002 | 4.60045e-11 | 448651 | 43.345 |
| Cooked vegetable intake | rs2890620 | 4 | 38736550 | C | T | 0.281 | 0.008 | 0.002 | 1.89998e-06 | 448651 | 22.659 |
| Cooked vegetable intake | rs2917705 | 16 | 64339094 | A | G | 0.141 | -0.01 | 0.002 | 3.69999e-06 | 448651 | 21.422 |
| Cooked vegetable intake | rs29938 | 19 | 34311481 | C | T | 0.659 | 0.008 | 0.002 | 3.89996e-06 | 448651 | 21.337 |
| Cooked vegetable intake | rs333537 | 5 | 24921272 | T | C | 0.553 | -0.007 | 0.002 | 2.99999e-06 | 448651 | 21.793 |
| Cooked vegetable intake | rs335352 | 6 | 155623709 | A | G | 0.661 | 0.008 | 0.002 | 4.09996e-06 | 448651 | 21.229 |
| Cooked vegetable intake | rs34006433 | 11 | 104486062 | G | A | 0.202 | -0.009 | 0.002 | 2.19999e-06 | 448651 | 22.44 |
| Cooked vegetable intake | rs34155012 | 22 | 48977215 | T | C | 0.227 | 0.011 | 0.002 | 3.89996e-08 | 448651 | 30.175 |
| Cooked vegetable intake | rs34299746 | 5 | 151905461 | T | G | 0.421 | -0.008 | 0.002 | 1.40001e-06 | 448651 | 23.265 |
| Cooked vegetable intake | rs34903 | 3 | 10449459 | T | C | 0.459 | 0.008 | 0.002 | 1e-06 | 448651 | 23.923 |
| Cooked vegetable intake | rs4072717 | 8 | 33088271 | T | C | 0.264 | -0.008 | 0.002 | 2.39999e-06 | 448651 | 22.276 |
| Cooked vegetable intake | rs4730098 | 7 | 77814476 | A | G | 0.629 | 0.008 | 0.002 | 8e-07 | 448651 | 24.348 |
| Cooked vegetable intake | rs4851029 | 2 | 104159785 | G | T | 0.527 | 0.01 | 0.002 | 7.8001e-11 | 448651 | 42.307 |
| Cooked vegetable intake | rs4852492 | 2 | 79702840 | T | C | 0.671 | -0.009 | 0.002 | 2.69998e-07 | 448651 | 26.48 |
| Cooked vegetable intake | rs56026156 | 2 | 241910466 | T | C | 0.012 | -0.035 | 0.007 | 9.09997e-07 | 448651 | 24.113 |
| Cooked vegetable intake | rs56814511 | 12 | 125789014 | T | C | 0.297 | -0.009 | 0.002 | 1.09999e-06 | 448651 | 23.736 |
| Cooked vegetable intake | rs58413281 | 17 | 27055311 | T | C | 0.087 | -0.013 | 0.003 | 4.30002e-06 | 448651 | 21.13 |
| Cooked vegetable intake | rs60123882 | 3 | 51689306 | G | A | 0.206 | -0.01 | 0.002 | 5.39995e-07 | 448651 | 25.11 |
| Cooked vegetable intake | rs6088332 | 20 | 32466983 | T | C | 0.127 | 0.011 | 0.002 | 2.69998e-06 | 448651 | 21.989 |
| Cooked vegetable intake | rs62000008 | 15 | 33396219 | G | T | 0.115 | 0.013 | 0.002 | 1.6e-07 | 448651 | 27.406 |
| Cooked vegetable intake | rs62291414 | 3 | 180624900 | C | T | 0.217 | 0.009 | 0.002 | 3.89996e-06 | 448651 | 21.311 |
| Cooked vegetable intake | rs62485872 | 7 | 103668474 | T | G | 0.214 | -0.009 | 0.002 | 1.6e-06 | 448651 | 22.994 |
| Cooked vegetable intake | rs6532382 | 4 | 93786775 | A | G | 0.32 | 0.009 | 0.002 | 2.90001e-07 | 448651 | 26.325 |
| Cooked vegetable intake | rs6606711 | 12 | 109849297 | G | A | 0.345 | -0.008 | 0.002 | 2e-06 | 448651 | 22.571 |
| Cooked vegetable intake | rs67201082 | 2 | 116017266 | G | T | 0.161 | -0.01 | 0.002 | 4.90004e-06 | 448651 | 20.886 |
| Cooked vegetable intake | rs67476894 | 15 | 29803243 | A | G | 0.296 | 0.009 | 0.002 | 4.79999e-07 | 448651 | 25.333 |
| Cooked vegetable intake | rs72815648 | 2 | 98316794 | A | G | 0.014 | -0.03 | 0.007 | 4.09996e-06 | 448651 | 21.212 |
| Cooked vegetable intake | rs74542895 | 9 | 114781676 | T | C | 0.024 | -0.024 | 0.005 | 4.90004e-06 | 448651 | 20.885 |
| Cooked vegetable intake | rs7513532 | 1 | 245354548 | A | G | 0.103 | -0.013 | 0.003 | 1.7e-06 | 448651 | 22.935 |
| Cooked vegetable intake | rs7667638 | 4 | 10543372 | G | A | 0.786 | 0.009 | 0.002 | 1.09999e-06 | 448651 | 23.729 |
| Cooked vegetable intake | rs7743389 | 6 | 8906594 | T | C | 0.883 | -0.011 | 0.002 | 3.29997e-06 | 448651 | 21.616 |
| Cooked vegetable intake | rs7803655 | 7 | 21400762 | A | G | 0.459 | 0.008 | 0.002 | 1.09999e-06 | 448651 | 23.806 |
| Cooked vegetable intake | rs7975681 | 12 | 24240259 | C | T | 0.501 | -0.008 | 0.002 | 1.2e-07 | 448651 | 28.056 |
| Cooked vegetable intake | rs7984840 | 13 | 90719301 | T | G | 0.228 | 0.009 | 0.002 | 1.29999e-06 | 448651 | 23.498 |
| Cooked vegetable intake | rs8015697 | 14 | 80339440 | T | G | 0.803 | -0.01 | 0.002 | 1.6e-07 | 448651 | 27.496 |
| Cooked vegetable intake | rs838133 | 19 | 49259529 | G | A | 0.55 | 0.012 | 0.002 | 4.49987e-13 | 448651 | 52.396 |
| Cooked vegetable intake | rs9400763 | 6 | 115155798 | T | C | 0.761 | -0.01 | 0.002 | 1.7e-07 | 448651 | 27.38 |
| Cooked vegetable intake | rs9630374 | 14 | 32473139 | T | C | 0.321 | -0.008 | 0.002 | 5e-07 | 448651 | 25.245 |
| Salad / raw vegetable intake | rs1004549 | 13 | 108709340 | C | A | 0.91 | -0.012 | 0.003 | 4.20001e-06 | 435435 | 21.15 |
| Salad / raw vegetable intake | rs1011530 | 9 | 13755456 | A | C | 0.705 | -0.008 | 0.002 | 5.60003e-07 | 435435 | 25.052 |
| Salad / raw vegetable intake | rs1011939 | 16 | 19992996 | A | G | 0.716 | -0.008 | 0.002 | 1.40001e-07 | 435435 | 27.681 |
| Salad / raw vegetable intake | rs1052352 | 16 | 31195279 | T | C | 0.524 | 0.008 | 0.001 | 1e-08 | 435435 | 32.797 |
| Salad / raw vegetable intake | rs10819082 | 9 | 128645617 | A | G | 0.667 | -0.009 | 0.002 | 1.40001e-09 | 435435 | 36.671 |
| Salad / raw vegetable intake | rs10960118 | 9 | 11709678 | A | G | 0.252 | -0.008 | 0.002 | 5.39995e-07 | 435435 | 25.1 |
| Salad / raw vegetable intake | rs11125813 | 2 | 59991047 | A | G | 0.219 | 0.009 | 0.002 | 2.39999e-07 | 435435 | 26.684 |
| Salad / raw vegetable intake | rs11209952 | 1 | 72837500 | T | C | 0.598 | 0.007 | 0.001 | 2e-06 | 435435 | 22.61 |
| Salad / raw vegetable intake | rs112179509 | 14 | 79203148 | T | G | 0.008 | 0.038 | 0.008 | 1.79999e-06 | 435435 | 22.85 |
| Salad / raw vegetable intake | rs11223775 | 11 | 134247895 | T | C | 0.341 | -0.007 | 0.002 | 2e-06 | 435435 | 22.606 |
| Salad / raw vegetable intake | rs115312977 | 1 | 35794177 | T | G | 0.067 | -0.013 | 0.003 | 4.20001e-06 | 435435 | 21.174 |
| Salad / raw vegetable intake | rs11609549 | 12 | 49899635 | T | C | 0.069 | 0.014 | 0.003 | 4.60002e-07 | 435435 | 25.44 |
| Salad / raw vegetable intake | rs116493405 | 3 | 114733556 | A | G | 0.054 | 0.015 | 0.003 | 3.2e-06 | 435435 | 21.672 |
| Salad / raw vegetable intake | rs117443106 | 14 | 98327132 | C | T | 0.053 | 0.015 | 0.003 | 1.09999e-06 | 435435 | 23.697 |
| Salad / raw vegetable intake | rs11824377 | 11 | 11787253 | G | A | 0.561 | -0.007 | 0.001 | 2.30001e-07 | 435435 | 26.755 |
| Salad / raw vegetable intake | rs12203592 | 6 | 396321 | T | C | 0.219 | -0.01 | 0.002 | 1.29999e-09 | 435435 | 36.866 |
| Salad / raw vegetable intake | rs12324270 | 15 | 96274470 | C | A | 0.011 | -0.032 | 0.007 | 3.40001e-06 | 435435 | 21.575 |
| Salad / raw vegetable intake | rs12373799 | 2 | 27293982 | A | G | 0.096 | -0.013 | 0.002 | 3.29997e-07 | 435435 | 26.053 |
| Salad / raw vegetable intake | rs12517711 | 5 | 60754661 | C | T | 0.391 | -0.007 | 0.001 | 4e-06 | 435435 | 21.241 |
| Salad / raw vegetable intake | rs12630752 | 3 | 44303185 | G | A | 0.233 | -0.008 | 0.002 | 7.00003e-07 | 435435 | 24.614 |
| Salad / raw vegetable intake | rs12908495 | 15 | 97011280 | A | C | 0.243 | -0.009 | 0.002 | 2e-08 | 435435 | 31.507 |
| Salad / raw vegetable intake | rs12910046 | 15 | 78191757 | C | T | 0.267 | 0.008 | 0.002 | 6.4e-07 | 435435 | 24.773 |
| Salad / raw vegetable intake | rs13020607 | 2 | 127163515 | C | T | 0.166 | -0.01 | 0.002 | 1.40001e-07 | 435435 | 27.755 |
| Salad / raw vegetable intake | rs13167468 | 5 | 149153043 | C | T | 0.109 | 0.011 | 0.002 | 1.09999e-06 | 435435 | 23.77 |
| Salad / raw vegetable intake | rs140806757 | 11 | 56181053 | C | T | 0.014 | -0.028 | 0.006 | 4e-06 | 435435 | 21.265 |
| Salad / raw vegetable intake | rs146555011 | 10 | 80416410 | T | C | 0.038 | 0.019 | 0.004 | 4.39997e-07 | 435435 | 25.532 |
| Salad / raw vegetable intake | rs1528458 | 2 | 157110464 | G | T | 0.333 | -0.007 | 0.002 | 4.20001e-06 | 435435 | 21.178 |
| Salad / raw vegetable intake | rs16869875 | 6 | 32192217 | T | C | 0.013 | 0.029 | 0.006 | 3.79997e-06 | 435435 | 21.388 |
| Salad / raw vegetable intake | rs17457620 | 1 | 96700240 | T | C | 0.491 | 0.007 | 0.001 | 8.09991e-07 | 435435 | 24.324 |
| Salad / raw vegetable intake | rs2183298 | 9 | 92178487 | T | C | 0.345 | 0.007 | 0.002 | 8.40001e-07 | 435435 | 24.27 |
| Salad / raw vegetable intake | rs2289322 | 18 | 1884329 | T | G | 0.147 | 0.01 | 0.002 | 2.1e-06 | 435435 | 22.538 |
| Salad / raw vegetable intake | rs2314765 | 3 | 1594678 | G | T | 0.04 | -0.017 | 0.004 | 4.60002e-06 | 435435 | 21.01 |
| Salad / raw vegetable intake | rs2395441 | 10 | 78602758 | A | G | 0.542 | -0.007 | 0.001 | 4.20001e-06 | 435435 | 21.157 |
| Salad / raw vegetable intake | rs2447090 | 17 | 2298974 | G | A | 0.361 | -0.007 | 0.001 | 1e-06 | 435435 | 23.878 |
| Salad / raw vegetable intake | rs2658193 | 10 | 12159472 | C | A | 0.875 | 0.01 | 0.002 | 1.2e-06 | 435435 | 23.587 |
| Salad / raw vegetable intake | rs266070 | 2 | 104076655 | G | A | 0.536 | -0.007 | 0.001 | 1.2e-06 | 435435 | 23.566 |
| Salad / raw vegetable intake | rs2866121 | 3 | 118420088 | A | C | 0.491 | 0.007 | 0.001 | 1.89998e-07 | 435435 | 27.085 |
| Salad / raw vegetable intake | rs2897290 | 8 | 35314837 | T | C | 0.405 | 0.008 | 0.002 | 1.2e-07 | 435435 | 28.011 |
| Salad / raw vegetable intake | rs2924716 | 8 | 4849150 | G | T | 0.512 | -0.008 | 0.001 | 7.90005e-08 | 435435 | 28.842 |
| Salad / raw vegetable intake | rs338925 | 1 | 58862172 | C | T | 0.675 | -0.007 | 0.002 | 2.39999e-06 | 435435 | 22.242 |
| Salad / raw vegetable intake | rs3879447 | 1 | 109891403 | T | C | 0.71 | 0.008 | 0.002 | 1.29999e-06 | 435435 | 23.365 |
| Salad / raw vegetable intake | rs402360 | 5 | 166408664 | C | T | 0.363 | 0.007 | 0.002 | 3.79997e-06 | 435435 | 21.364 |
| Salad / raw vegetable intake | rs4291983 | 18 | 38106410 | A | C | 0.518 | -0.008 | 0.001 | 3.69999e-09 | 435435 | 34.756 |
| Salad / raw vegetable intake | rs4381643 | 17 | 30577131 | A | G | 0.087 | 0.012 | 0.003 | 9.69996e-07 | 435435 | 23.988 |
| Salad / raw vegetable intake | rs4762485 | 12 | 98778360 | T | C | 0.257 | -0.008 | 0.002 | 3.09999e-07 | 435435 | 26.19 |
| Salad / raw vegetable intake | rs4782717 | 16 | 82609717 | C | T | 0.716 | 0.008 | 0.002 | 7.00003e-07 | 435435 | 24.608 |
| Salad / raw vegetable intake | rs4923594 | 11 | 21433028 | T | C | 0.688 | 0.008 | 0.002 | 1.09999e-06 | 435435 | 23.712 |
| Salad / raw vegetable intake | rs56367474 | 13 | 59454139 | T | C | 0.313 | -0.008 | 0.002 | 3.40001e-07 | 435435 | 26.032 |
| Salad / raw vegetable intake | rs58214669 | 5 | 56814463 | T | C | 0.049 | -0.016 | 0.003 | 6.90001e-07 | 435435 | 24.636 |
| Salad / raw vegetable intake | rs59128343 | 13 | 48976738 | T | C | 0.565 | -0.007 | 0.001 | 1.2e-06 | 435435 | 23.639 |
| Salad / raw vegetable intake | rs6090392 | 20 | 62008975 | T | G | 0.372 | 0.007 | 0.001 | 5.39995e-07 | 435435 | 25.113 |
| Salad / raw vegetable intake | rs61884825 | 11 | 43619977 | A | G | 0.085 | -0.012 | 0.003 | 1.29999e-06 | 435435 | 23.438 |
| Salad / raw vegetable intake | rs62107261 | 2 | 422144 | C | T | 0.048 | -0.017 | 0.003 | 3.2e-07 | 435435 | 26.106 |
| Salad / raw vegetable intake | rs62109696 | 2 | 20386297 | A | G | 0.124 | -0.01 | 0.002 | 3.09999e-06 | 435435 | 21.727 |
| Salad / raw vegetable intake | rs62132810 | 19 | 49279227 | A | G | 0.113 | 0.012 | 0.002 | 1.89998e-07 | 435435 | 27.091 |
| Salad / raw vegetable intake | rs62150280 | 2 | 100772180 | G | T | 0.02 | -0.028 | 0.005 | 6.80002e-08 | 435435 | 29.114 |
| Salad / raw vegetable intake | rs62258905 | 3 | 104375385 | T | G | 0.242 | -0.008 | 0.002 | 2.30001e-06 | 435435 | 22.292 |
| Salad / raw vegetable intake | rs62461186 | 7 | 77730153 | C | A | 0.18 | -0.011 | 0.002 | 1e-09 | 435435 | 37.303 |
| Salad / raw vegetable intake | rs638089 | 11 | 111340452 | T | C | 0.518 | -0.007 | 0.001 | 1.6e-07 | 435435 | 27.456 |
| Salad / raw vegetable intake | rs6441954 | 3 | 46332840 | C | T | 0.468 | -0.007 | 0.001 | 4e-06 | 435435 | 21.285 |
| Salad / raw vegetable intake | rs6460754 | 7 | 10858638 | C | T | 0.539 | 0.007 | 0.001 | 1.40001e-06 | 435435 | 23.299 |
| Salad / raw vegetable intake | rs6482190 | 10 | 22037809 | G | A | 0.719 | 0.011 | 0.002 | 1.39991e-12 | 435435 | 50.241 |
| Salad / raw vegetable intake | rs6508486 | 18 | 24999015 | A | G | 0.467 | 0.007 | 0.001 | 1.40001e-06 | 435435 | 23.299 |
| Salad / raw vegetable intake | rs67497633 | 10 | 103815495 | A | G | 0.168 | 0.01 | 0.002 | 3.69999e-07 | 435435 | 25.822 |
| Salad / raw vegetable intake | rs6824864 | 4 | 42170528 | T | C | 0.272 | -0.007 | 0.002 | 4.60002e-06 | 435435 | 20.987 |
| Salad / raw vegetable intake | rs6879110 | 5 | 164640976 | C | A | 0.19 | -0.009 | 0.002 | 9.69996e-07 | 435435 | 23.996 |
| Salad / raw vegetable intake | rs7045800 | 9 | 75924896 | C | T | 0.745 | -0.008 | 0.002 | 1.7e-06 | 435435 | 22.901 |
| Salad / raw vegetable intake | rs71466817 | 15 | 35925895 | T | C | 0.212 | 0.009 | 0.002 | 1.29999e-07 | 435435 | 27.886 |
| Salad / raw vegetable intake | rs71645290 | 1 | 176467395 | T | C | 0.05 | 0.015 | 0.003 | 3.29997e-06 | 435435 | 21.661 |
| Salad / raw vegetable intake | rs72974269 | 2 | 225454907 | T | C | 0.317 | 0.007 | 0.002 | 1.29999e-06 | 435435 | 23.372 |
| Salad / raw vegetable intake | rs7433849 | 3 | 69043012 | C | T | 0.541 | -0.007 | 0.001 | 8.60003e-07 | 435435 | 24.212 |
| Salad / raw vegetable intake | rs7458242 | 7 | 114184098 | A | G | 0.602 | -0.007 | 0.001 | 1.2e-06 | 435435 | 23.535 |
| Salad / raw vegetable intake | rs75248709 | 6 | 92348945 | T | C | 0.046 | -0.02 | 0.004 | 2.19999e-08 | 435435 | 31.292 |
| Salad / raw vegetable intake | rs76566997 | 16 | 75050068 | A | G | 0.191 | 0.008 | 0.002 | 3.2e-06 | 435435 | 21.684 |
| Salad / raw vegetable intake | rs78706681 | 10 | 596939 | A | G | 0.054 | -0.016 | 0.003 | 5.1e-07 | 435435 | 25.207 |
| Salad / raw vegetable intake | rs790561 | 8 | 64618026 | G | A | 0.704 | 0.012 | 0.002 | 1.39991e-15 | 435435 | 63.775 |
| Salad / raw vegetable intake | rs79199842 | 4 | 16887894 | A | G | 0.015 | -0.027 | 0.006 | 4.30002e-06 | 435435 | 21.128 |
| Salad / raw vegetable intake | rs7970482 | 12 | 109862335 | A | G | 0.311 | -0.008 | 0.002 | 1.7e-07 | 435435 | 27.298 |
| Salad / raw vegetable intake | rs8130508 | 21 | 19049865 | A | G | 0.29 | 0.009 | 0.002 | 2.99999e-08 | 435435 | 30.681 |
| Salad / raw vegetable intake | rs817555 | 17 | 67423274 | A | G | 0.455 | -0.007 | 0.001 | 4.49997e-07 | 435435 | 25.463 |
| Salad / raw vegetable intake | rs9323534 | 14 | 20586432 | T | C | 0.433 | -0.007 | 0.001 | 3.79997e-06 | 435435 | 21.383 |
| Salad / raw vegetable intake | rs9342711 | 6 | 69197250 | G | A | 0.301 | 0.008 | 0.002 | 4.70002e-07 | 435435 | 25.399 |
| Salad / raw vegetable intake | rs9383456 | 6 | 19058692 | G | A | 0.785 | 0.009 | 0.002 | 4.30002e-07 | 435435 | 25.567 |
| Salad / raw vegetable intake | rs9387295 | 6 | 115510990 | A | G | 0.271 | -0.008 | 0.002 | 2.39999e-06 | 435435 | 22.254 |
| Salad / raw vegetable intake | rs9790574 | 4 | 46734901 | T | C | 0.18 | -0.009 | 0.002 | 9.40005e-07 | 435435 | 24.047 |
| Salad / raw vegetable intake | rs9837462 | 3 | 85158566 | A | C | 0.343 | 0.008 | 0.002 | 5.1e-08 | 435435 | 29.687 |
| Milk intake | rs115526621 | 1 | 2256290 | T | C | 0.027 | 0.036 | 0.008 | 4.70002e-06 | 64943 | 20.936 |
| Milk intake | rs117951732 | 19 | 385145 | G | T | 0.031 | 0.035 | 0.007 | 3.2e-06 | 64943 | 21.669 |
| Milk intake | rs12947049 | 17 | 30124654 | A | C | 0.6 | -0.012 | 0.003 | 3.69999e-06 | 64943 | 21.408 |
| Milk intake | rs13072001 | 3 | 85494102 | C | A | 0.014 | 0.052 | 0.011 | 2.30001e-06 | 64943 | 22.294 |
| Milk intake | rs143934486 | 5 | 142952674 | G | A | 0.015 | 0.05 | 0.011 | 2.90001e-06 | 64943 | 21.863 |
| Milk intake | rs145140220 | 14 | 95557284 | G | A | 0.024 | 0.039 | 0.008 | 4.49997e-06 | 64943 | 21.053 |
| Milk intake | rs1549862 | 7 | 10390312 | C | A | 0.318 | -0.013 | 0.003 | 2.80001e-06 | 64943 | 21.933 |
| Milk intake | rs191749866 | 2 | 39603005 | C | T | 0.011 | 0.064 | 0.012 | 1.40001e-07 | 64943 | 27.66 |
| Milk intake | rs191821864 | 7 | 152092773 | G | A | 0.021 | 0.043 | 0.009 | 2.80001e-06 | 64943 | 21.927 |
| Milk intake | rs2807888 | 6 | 137592006 | A | G | 0.842 | -0.017 | 0.003 | 1.2e-06 | 64943 | 23.52 |
| Milk intake | rs34980790 | 18 | 56667779 | C | T | 0.362 | 0.013 | 0.003 | 8.9e-07 | 64943 | 24.158 |
| Milk intake | rs3744761 | 17 | 43196013 | T | C | 0.044 | 0.03 | 0.006 | 8.30004e-07 | 64943 | 24.287 |
| Milk intake | rs56674454 | 18 | 39827563 | C | T | 0.219 | 0.015 | 0.003 | 6.90001e-07 | 64943 | 24.638 |
| Milk intake | rs6025776 | 20 | 56351895 | A | C | 0.274 | 0.013 | 0.003 | 4.09996e-06 | 64943 | 21.197 |
| Milk intake | rs62435191 | 6 | 77803688 | A | G | 0.138 | 0.018 | 0.004 | 8.99995e-07 | 64943 | 24.138 |
| Milk intake | rs6489968 | 12 | 115138644 | A | G | 0.888 | -0.018 | 0.004 | 3.79997e-06 | 64943 | 21.364 |
| Milk intake | rs73164589 | 22 | 37755022 | A | G | 0.032 | 0.033 | 0.007 | 4.20001e-06 | 64943 | 21.148 |
| Milk intake | rs781770 | 1 | 44974119 | T | C | 0.191 | -0.015 | 0.003 | 1.09999e-06 | 64943 | 23.723 |
| Milk intake | rs9342975 | 6 | 73561338 | A | C | 0.693 | -0.013 | 0.003 | 2.59998e-06 | 64943 | 22.125 |
| Cheese intake | rs10138053 | 14 | 51615152 | C | T | 0.719 | 0.012 | 0.002 | 3.09999e-06 | 451486 | 21.759 |
| Cheese intake | rs10175930 | 2 | 215153099 | A | G | 0.296 | -0.012 | 0.002 | 1.09999e-06 | 451486 | 23.695 |
| Cheese intake | rs10269830 | 7 | 70109813 | G | A | 0.6 | -0.011 | 0.002 | 1.2e-06 | 451486 | 23.61 |
| Cheese intake | rs10499731 | 7 | 54411063 | G | T | 0.314 | 0.013 | 0.002 | 1.40001e-07 | 451486 | 27.781 |
| Cheese intake | rs1073242 | 13 | 58715219 | A | G | 0.554 | 0.016 | 0.002 | 6.70039e-12 | 451486 | 47.107 |
| Cheese intake | rs10896050 | 11 | 65577516 | T | G | 0.193 | -0.018 | 0.003 | 7.19946e-11 | 451486 | 42.461 |
| Cheese intake | rs10919061 | 1 | 169082364 | T | C | 0.149 | -0.016 | 0.003 | 6.19998e-07 | 451486 | 24.839 |
| Cheese intake | rs10938397 | 4 | 45182527 | G | A | 0.434 | -0.013 | 0.002 | 1.79999e-08 | 451486 | 31.683 |
| Cheese intake | rs11104660 | 12 | 88303151 | A | G | 0.665 | 0.012 | 0.002 | 4.60002e-07 | 451486 | 25.437 |
| Cheese intake | rs11130789 | 3 | 60838518 | T | C | 0.715 | 0.013 | 0.002 | 3.69999e-07 | 451486 | 25.833 |
| Cheese intake | rs111423440 | 3 | 58701720 | A | G | 0.024 | 0.038 | 0.008 | 2.30001e-06 | 451486 | 22.291 |
| Cheese intake | rs11213287 | 11 | 110033860 | C | T | 0.473 | -0.011 | 0.002 | 5.39995e-07 | 451486 | 25.132 |
| Cheese intake | rs11264431 | 1 | 156022802 | T | C | 0.554 | -0.011 | 0.002 | 1.79999e-06 | 451486 | 22.828 |
| Cheese intake | rs113067014 | 1 | 217544273 | A | G | 0.045 | 0.026 | 0.006 | 2.19999e-06 | 451486 | 22.444 |
| Cheese intake | rs113367286 | 7 | 140144414 | T | C | 0.278 | 0.015 | 0.002 | 1.29999e-09 | 451486 | 36.872 |
| Cheese intake | rs113811945 | 5 | 3667173 | C | T | 0.117 | 0.018 | 0.004 | 9.69996e-07 | 451486 | 23.995 |
| Cheese intake | rs113829457 | 5 | 27120060 | C | T | 0.168 | -0.014 | 0.003 | 2.5e-06 | 451486 | 22.179 |
| Cheese intake | rs114121746 | 3 | 159158671 | T | G | 0.099 | 0.018 | 0.004 | 8.9e-07 | 451486 | 24.155 |
| Cheese intake | rs114534035 | 3 | 84747224 | G | A | 0.035 | -0.028 | 0.006 | 4e-06 | 451486 | 21.245 |
| Cheese intake | rs114964798 | 3 | 187489547 | T | C | 0.108 | -0.018 | 0.004 | 9.90011e-07 | 451486 | 23.945 |
| Cheese intake | rs11620149 | 13 | 89816082 | C | T | 0.143 | -0.018 | 0.003 | 3.59998e-08 | 451486 | 30.345 |
| Cheese intake | rs11669334 | 19 | 32200518 | T | G | 0.18 | 0.014 | 0.003 | 8.9e-07 | 451486 | 24.155 |
| Cheese intake | rs11674347 | 2 | 233941388 | G | A | 0.024 | -0.034 | 0.007 | 2.1e-06 | 451486 | 22.476 |
| Cheese intake | rs11735256 | 4 | 106138146 | C | T | 0.641 | -0.012 | 0.002 | 2.90001e-07 | 451486 | 26.342 |
| Cheese intake | rs117371901 | 9 | 34361732 | T | C | 0.078 | -0.019 | 0.004 | 3.29997e-06 | 451486 | 21.638 |
| Cheese intake | rs11756671 | 6 | 69149444 | A | C | 0.341 | -0.011 | 0.002 | 4.49997e-06 | 451486 | 21.045 |
| Cheese intake | rs118054760 | 9 | 31833678 | C | T | 0.006 | 0.071 | 0.016 | 4.79999e-06 | 451486 | 20.927 |
| Cheese intake | rs12296440 | 12 | 49927148 | A | G | 0.17 | 0.019 | 0.003 | 2.80001e-10 | 451486 | 39.779 |
| Cheese intake | rs1235070 | 2 | 81984166 | T | C | 0.528 | -0.011 | 0.002 | 4.39997e-07 | 451486 | 25.51 |
| Cheese intake | rs12447542 | 16 | 7744180 | A | G | 0.126 | 0.02 | 0.003 | 6.80002e-09 | 451486 | 33.596 |
| Cheese intake | rs12467836 | 2 | 144685258 | A | G | 0.334 | 0.012 | 0.002 | 1.2e-06 | 451486 | 23.561 |
| Cheese intake | rs12653706 | 5 | 124247103 | T | C | 0.094 | 0.018 | 0.004 | 4.30002e-06 | 451486 | 21.118 |
| Cheese intake | rs12666766 | 7 | 21057473 | A | C | 0.692 | 0.011 | 0.002 | 4.39997e-06 | 451486 | 21.1 |
| Cheese intake | rs12672200 | 7 | 115461436 | A | G | 0.326 | -0.014 | 0.002 | 8.99995e-09 | 451486 | 33.037 |
| Cheese intake | rs1291145 | 20 | 35528475 | C | T | 0.686 | -0.02 | 0.002 | 4.40048e-17 | 451486 | 70.583 |
| Cheese intake | rs13107325 | 4 | 103188709 | T | C | 0.075 | -0.029 | 0.004 | 7.00003e-12 | 451486 | 47.025 |
| Cheese intake | rs13181793 | 5 | 138408956 | A | G | 0.446 | -0.011 | 0.002 | 7.90005e-07 | 451486 | 24.378 |
| Cheese intake | rs13266268 | 8 | 142611971 | T | C | 0.389 | -0.012 | 0.002 | 4.09996e-07 | 451486 | 25.645 |
| Cheese intake | rs136309 | 22 | 31175525 | A | C | 0.205 | -0.014 | 0.003 | 4.20001e-07 | 451486 | 25.591 |
| Cheese intake | rs141547796 | 6 | 50615935 | A | G | 0.082 | 0.021 | 0.004 | 2.69998e-07 | 451486 | 26.489 |
| Cheese intake | rs141707337 | 7 | 341167 | T | C | 0.129 | -0.023 | 0.004 | 6.59994e-08 | 451486 | 29.165 |
| Cheese intake | rs1434511 | 18 | 44829435 | T | C | 0.455 | 0.013 | 0.002 | 9.49992e-09 | 451486 | 32.946 |
| Cheese intake | rs145345105 | 11 | 30089136 | A | G | 0.011 | 0.052 | 0.011 | 2e-06 | 451486 | 22.586 |
| Cheese intake | rs1473677 | 6 | 136767530 | A | G | 0.854 | 0.015 | 0.003 | 4.49997e-06 | 451486 | 21.039 |
| Cheese intake | rs1473781 | 15 | 41818917 | A | G | 0.346 | -0.012 | 0.002 | 5.80003e-07 | 451486 | 24.964 |
| Cheese intake | rs150702313 | 11 | 77270442 | A | G | 0.083 | -0.02 | 0.004 | 5.30005e-07 | 451486 | 25.161 |
| Cheese intake | rs150830434 | 8 | 85910509 | T | G | 0.055 | -0.026 | 0.005 | 2.80001e-07 | 451486 | 26.391 |
| Cheese intake | rs1514755 | 2 | 166299635 | G | A | 0.24 | 0.016 | 0.003 | 3.89996e-10 | 451486 | 39.166 |
| Cheese intake | rs1519043 | 9 | 109942883 | G | T | 0.581 | -0.011 | 0.002 | 7.69999e-07 | 451486 | 24.428 |
| Cheese intake | rs1536456 | 14 | 68674793 | G | A | 0.642 | 0.011 | 0.002 | 1.5e-06 | 451486 | 23.206 |
| Cheese intake | rs1647396 | 11 | 57401183 | A | G | 0.539 | -0.012 | 0.002 | 7.39997e-08 | 451486 | 28.961 |
| Cheese intake | rs17088737 | 8 | 22761463 | C | T | 0.175 | 0.014 | 0.003 | 3.2e-06 | 451486 | 21.706 |
| Cheese intake | rs17115145 | 14 | 30122409 | T | C | 0.401 | -0.013 | 0.002 | 1.79999e-08 | 451486 | 31.649 |
| Cheese intake | rs17207890 | 11 | 95490754 | A | G | 0.344 | 0.012 | 0.002 | 7.69999e-07 | 451486 | 24.432 |
| Cheese intake | rs17342130 | 6 | 157313129 | G | A | 0.268 | -0.012 | 0.003 | 1.09999e-06 | 451486 | 23.697 |
| Cheese intake | rs17481439 | 12 | 24174817 | A | G | 0.21 | 0.013 | 0.003 | 2e-06 | 451486 | 22.561 |
| Cheese intake | rs17704703 | 10 | 55566406 | G | T | 0.314 | -0.011 | 0.002 | 4.60002e-06 | 451486 | 20.998 |
| Cheese intake | rs1806771 | 10 | 63682387 | G | T | 0.088 | -0.022 | 0.004 | 4.09996e-08 | 451486 | 30.125 |
| Cheese intake | rs1873036 | 7 | 95551919 | T | C | 0.176 | 0.014 | 0.003 | 9.49992e-07 | 451486 | 24.026 |
| Cheese intake | rs1886032 | 13 | 100561834 | C | T | 0.028 | -0.037 | 0.007 | 7.59994e-08 | 451486 | 28.907 |
| Cheese intake | rs1931805 | 6 | 62630863 | C | T | 0.5 | 0.013 | 0.002 | 1.6e-08 | 451486 | 31.94 |
| Cheese intake | rs1965353 | 3 | 177333565 | C | A | 0.816 | 0.014 | 0.003 | 1.2e-06 | 451486 | 23.625 |
| Cheese intake | rs2029401 | 5 | 92891029 | G | A | 0.586 | 0.012 | 0.002 | 3.09999e-07 | 451486 | 26.215 |
| Cheese intake | rs2092412 | 1 | 6279118 | C | T | 0.032 | -0.03 | 0.006 | 2.90001e-06 | 451486 | 21.869 |
| Cheese intake | rs221418 | 14 | 80038474 | G | T | 0.369 | 0.011 | 0.002 | 4.90004e-06 | 451486 | 20.87 |
| Cheese intake | rs2245959 | 6 | 29943970 | A | G | 0.358 | 0.012 | 0.002 | 6.59994e-07 | 451486 | 24.741 |
| Cheese intake | rs2302351 | 3 | 44039889 | A | G | 0.47 | 0.011 | 0.002 | 5.39995e-07 | 451486 | 25.116 |
| Cheese intake | rs2330698 | 5 | 31300815 | A | G | 0.301 | -0.011 | 0.002 | 4.09996e-06 | 451486 | 21.216 |
| Cheese intake | rs2339928 | 2 | 24049453 | A | G | 0.704 | 0.015 | 0.002 | 1.2e-09 | 451486 | 36.929 |
| Cheese intake | rs2372904 | 3 | 81498737 | G | A | 0.466 | -0.011 | 0.002 | 4e-07 | 451486 | 25.703 |
| Cheese intake | rs242643 | 18 | 58853324 | G | A | 0.303 | -0.012 | 0.002 | 1.6e-06 | 451486 | 23.036 |
| Cheese intake | rs2644109 | 1 | 201797535 | T | C | 0.336 | -0.011 | 0.002 | 2.1e-06 | 451486 | 22.505 |
| Cheese intake | rs2802530 | 1 | 98546134 | A | G | 0.877 | 0.019 | 0.003 | 4.20001e-08 | 451486 | 30.065 |
| Cheese intake | rs281219 | 15 | 47711652 | G | A | 0.788 | -0.013 | 0.003 | 9.40005e-07 | 451486 | 24.041 |
| Cheese intake | rs2824098 | 21 | 18235587 | G | A | 0.625 | 0.011 | 0.002 | 1.6e-06 | 451486 | 23.065 |
| Cheese intake | rs2832747 | 21 | 31646807 | T | C | 0.469 | -0.01 | 0.002 | 4.60002e-06 | 451486 | 21.008 |
| Cheese intake | rs2838986 | 21 | 47145374 | G | A | 0.816 | -0.015 | 0.003 | 2.59998e-07 | 451486 | 26.554 |
| Cheese intake | rs28415940 | 16 | 89294381 | C | T | 0.894 | -0.019 | 0.004 | 2e-07 | 451486 | 27.056 |
| Cheese intake | rs2854175 | 17 | 61998469 | A | C | 0.257 | 0.017 | 0.003 | 3.69999e-11 | 451486 | 43.771 |
| Cheese intake | rs2870681 | 4 | 94073948 | C | A | 0.368 | 0.011 | 0.002 | 1.6e-06 | 451486 | 23.015 |
| Cheese intake | rs2960578 | 18 | 21143739 | G | T | 0.496 | 0.017 | 0.002 | 2.60016e-14 | 451486 | 57.998 |
| Cheese intake | rs2968940 | 4 | 130846669 | A | G | 0.682 | 0.012 | 0.002 | 3.09999e-07 | 451486 | 26.168 |
| Cheese intake | rs3204347 | 20 | 61443660 | A | G | 0.261 | -0.013 | 0.003 | 6.1e-07 | 451486 | 24.878 |
| Cheese intake | rs34198643 | 7 | 2203808 | T | C | 0.224 | -0.017 | 0.003 | 4.49997e-10 | 451486 | 38.874 |
| Cheese intake | rs34205186 | 5 | 142888276 | T | C | 0.43 | -0.011 | 0.002 | 2.80001e-06 | 451486 | 21.975 |
| Cheese intake | rs34423638 | 20 | 40025314 | G | A | 0.223 | 0.014 | 0.003 | 1.89998e-07 | 451486 | 27.094 |
| Cheese intake | rs35270670 | 15 | 83481880 | G | A | 0.218 | 0.016 | 0.003 | 1.5e-09 | 451486 | 36.537 |
| Cheese intake | rs35392738 | 10 | 107654922 | C | A | 0.292 | 0.012 | 0.002 | 2.5e-06 | 451486 | 22.132 |
| Cheese intake | rs35625885 | 15 | 96957969 | G | A | 0.125 | -0.017 | 0.003 | 9.69996e-07 | 451486 | 23.989 |
| Cheese intake | rs35760956 | 14 | 41134168 | G | A | 0.405 | -0.011 | 0.002 | 1.7e-06 | 451486 | 22.956 |
| Cheese intake | rs3733421 | 4 | 149002841 | C | T | 0.587 | -0.011 | 0.002 | 2.1e-06 | 451486 | 22.536 |
| Cheese intake | rs376580 | 6 | 165434818 | A | G | 0.611 | 0.011 | 0.002 | 4.20001e-06 | 451486 | 21.182 |
| Cheese intake | rs3911016 | 9 | 88029000 | G | T | 0.121 | 0.021 | 0.003 | 5.30005e-10 | 451486 | 38.548 |
| Cheese intake | rs40019 | 5 | 67942681 | G | A | 0.151 | 0.015 | 0.003 | 2e-06 | 451486 | 22.615 |
| Cheese intake | rs4140685 | 2 | 162852079 | T | G | 0.311 | 0.013 | 0.002 | 1.29999e-07 | 451486 | 27.835 |
| Cheese intake | rs4282974 | 11 | 11573407 | T | C | 0.908 | 0.018 | 0.004 | 4.30002e-06 | 451486 | 21.106 |
| Cheese intake | rs4296548 | 3 | 36915814 | G | T | 0.61 | 0.013 | 0.002 | 1.2e-08 | 451486 | 32.41 |
| Cheese intake | rs4503172 | 9 | 124640841 | T | C | 0.608 | 0.013 | 0.002 | 1.6e-08 | 451486 | 31.943 |
| Cheese intake | rs4655801 | 1 | 66240228 | G | T | 0.32 | -0.012 | 0.002 | 4.20001e-07 | 451486 | 25.593 |
| Cheese intake | rs4675530 | 2 | 206346565 | G | A | 0.587 | 0.011 | 0.002 | 9.59997e-07 | 451486 | 24.008 |
| Cheese intake | rs4692708 | 4 | 170228542 | C | A | 0.253 | 0.015 | 0.003 | 1.29999e-08 | 451486 | 32.368 |
| Cheese intake | rs4721694 | 7 | 18158464 | G | T | 0.2 | -0.013 | 0.003 | 2.99999e-06 | 451486 | 21.793 |
| Cheese intake | rs4860341 | 4 | 61251106 | C | T | 0.929 | 0.024 | 0.004 | 2.19999e-08 | 451486 | 31.351 |
| Cheese intake | rs4886168 | 13 | 60073056 | C | T | 0.161 | -0.014 | 0.003 | 4.60002e-06 | 451486 | 21.011 |
| Cheese intake | rs504675 | 2 | 45154689 | T | C | 0.353 | 0.027 | 0.002 | 1e-31 | 451486 | 137.314 |
| Cheese intake | rs524468 | 12 | 371786 | G | A | 0.261 | -0.014 | 0.003 | 2.39999e-08 | 451486 | 31.154 |
| Cheese intake | rs531358 | 1 | 93676011 | T | C | 0.65 | 0.013 | 0.002 | 1.79999e-08 | 451486 | 31.733 |
| Cheese intake | rs59784053 | 5 | 166109907 | G | A | 0.247 | -0.012 | 0.003 | 1.89998e-06 | 451486 | 22.672 |
| Cheese intake | rs6089798 | 20 | 62859179 | G | A | 0.129 | 0.016 | 0.003 | 2.99999e-06 | 451486 | 21.808 |
| Cheese intake | rs6126641 | 20 | 51296552 | A | G | 0.336 | 0.013 | 0.002 | 3.29997e-08 | 451486 | 30.498 |
| Cheese intake | rs614132 | 18 | 40985651 | G | A | 0.196 | 0.015 | 0.003 | 7.90005e-08 | 451486 | 28.829 |
| Cheese intake | rs61734410 | 16 | 1252369 | T | C | 0.255 | 0.017 | 0.003 | 2.19999e-10 | 451486 | 40.319 |
| Cheese intake | rs61757207 | 16 | 70358495 | G | A | 0.015 | -0.048 | 0.009 | 1.6e-07 | 451486 | 27.417 |
| Cheese intake | rs61953351 | 12 | 121456616 | T | G | 0.25 | 0.015 | 0.003 | 1.5e-08 | 451486 | 31.994 |
| Cheese intake | rs62034322 | 16 | 28535834 | A | G | 0.38 | -0.014 | 0.002 | 1.40001e-09 | 451486 | 36.715 |
| Cheese intake | rs62098037 | 18 | 50894725 | T | C | 0.525 | -0.011 | 0.002 | 9.69996e-07 | 451486 | 23.991 |
| Cheese intake | rs62236533 | 22 | 41992169 | A | G | 0.109 | 0.025 | 0.004 | 1.10002e-11 | 451486 | 46.105 |
| Cheese intake | rs6498981 | 16 | 65157443 | G | A | 0.709 | -0.012 | 0.002 | 2.19999e-06 | 451486 | 22.431 |
| Cheese intake | rs6539284 | 12 | 79592680 | C | T | 0.429 | 0.012 | 0.002 | 9.49992e-08 | 451486 | 28.477 |
| Cheese intake | rs6544743 | 2 | 44390369 | G | T | 0.809 | -0.013 | 0.003 | 3.2e-06 | 451486 | 21.676 |
| Cheese intake | rs660240 | 1 | 109817838 | C | T | 0.785 | -0.013 | 0.003 | 2.30001e-06 | 451486 | 22.343 |
| Cheese intake | rs6602662 | 10 | 13547709 | G | A | 0.737 | 0.012 | 0.003 | 2e-06 | 451486 | 22.613 |
| Cheese intake | rs66468220 | 5 | 96206563 | T | C | 0.159 | 0.016 | 0.003 | 1.29999e-07 | 451486 | 27.937 |
| Cheese intake | rs66510028 | 2 | 35059642 | C | T | 0.145 | -0.016 | 0.003 | 4.79999e-07 | 451486 | 25.356 |
| Cheese intake | rs6685323 | 1 | 154295592 | T | C | 0.309 | -0.013 | 0.002 | 4.79999e-08 | 451486 | 29.801 |
| Cheese intake | rs66905828 | 7 | 104576670 | C | A | 0.296 | 0.011 | 0.002 | 3.50002e-06 | 451486 | 21.522 |
| Cheese intake | rs6694085 | 1 | 33231380 | G | T | 0.169 | 0.014 | 0.003 | 3.29997e-06 | 451486 | 21.633 |
| Cheese intake | rs6706648 | 2 | 60722040 | T | C | 0.316 | 0.012 | 0.002 | 1.29999e-06 | 451486 | 23.47 |
| Cheese intake | rs67238148 | 11 | 7946480 | T | G | 0.217 | 0.017 | 0.003 | 1.09999e-09 | 451486 | 37.146 |
| Cheese intake | rs6774906 | 3 | 194803510 | C | A | 0.041 | 0.032 | 0.006 | 2.5e-08 | 451486 | 31.089 |
| Cheese intake | rs678349 | 11 | 105826990 | T | C | 0.54 | -0.01 | 0.002 | 3.69999e-06 | 451486 | 21.402 |
| Cheese intake | rs67967503 | 4 | 133515076 | G | A | 0.391 | -0.011 | 0.002 | 3.29997e-06 | 451486 | 21.633 |
| Cheese intake | rs683996 | 11 | 63874943 | T | C | 0.224 | 0.014 | 0.003 | 2.59998e-07 | 451486 | 26.494 |
| Cheese intake | rs6857 | 19 | 45392254 | T | C | 0.17 | -0.015 | 0.003 | 3.50002e-07 | 451486 | 25.931 |
| Cheese intake | rs6873324 | 5 | 153597288 | C | A | 0.426 | -0.012 | 0.002 | 3.89996e-08 | 451486 | 30.19 |
| Cheese intake | rs6897252 | 5 | 62952337 | T | C | 0.605 | -0.012 | 0.002 | 7.39997e-08 | 451486 | 28.953 |
| Cheese intake | rs7012814 | 8 | 9173358 | A | G | 0.474 | -0.019 | 0.002 | 2.09991e-16 | 451486 | 67.489 |
| Cheese intake | rs71386942 | 16 | 71992817 | A | C | 0.269 | 0.014 | 0.003 | 9.90011e-09 | 451486 | 32.851 |
| Cheese intake | rs71454634 | 12 | 120694815 | T | C | 0.047 | 0.027 | 0.005 | 4e-07 | 451486 | 25.703 |
| Cheese intake | rs7185391 | 16 | 68323115 | G | T | 0.725 | -0.013 | 0.003 | 1.09999e-07 | 451486 | 28.134 |
| Cheese intake | rs7236339 | 18 | 77579773 | A | G | 0.228 | -0.012 | 0.003 | 3.2e-06 | 451486 | 21.665 |
| Cheese intake | rs7259537 | 19 | 4582702 | T | C | 0.364 | -0.011 | 0.002 | 3.09999e-06 | 451486 | 21.749 |
| Cheese intake | rs72810360 | 2 | 58405715 | T | C | 0.175 | 0.017 | 0.003 | 1.29999e-08 | 451486 | 32.359 |
| Cheese intake | rs72814382 | 2 | 60062234 | A | G | 0.007 | 0.069 | 0.014 | 1.7e-06 | 451486 | 22.925 |
| Cheese intake | rs72829857 | 6 | 16966052 | G | A | 0.234 | 0.012 | 0.003 | 3.89996e-06 | 451486 | 21.29 |
| Cheese intake | rs72845670 | 10 | 104106200 | C | T | 0.392 | 0.012 | 0.002 | 3.29997e-07 | 451486 | 26.083 |
| Cheese intake | rs72970243 | 2 | 136484232 | A | G | 0.12 | 0.022 | 0.003 | 6.70039e-11 | 451486 | 42.596 |
| Cheese intake | rs7298331 | 12 | 22322789 | C | A | 0.605 | -0.013 | 0.002 | 1.09999e-08 | 451486 | 32.646 |
| Cheese intake | rs73096946 | 4 | 17788715 | C | T | 0.157 | -0.021 | 0.003 | 1.9002e-11 | 451486 | 45.086 |
| Cheese intake | rs73335955 | 10 | 106766879 | C | T | 0.053 | 0.028 | 0.005 | 2.39999e-08 | 451486 | 31.123 |
| Cheese intake | rs7386207 | 8 | 144246027 | T | C | 0.564 | -0.012 | 0.002 | 3.59998e-08 | 451486 | 30.33 |
| Cheese intake | rs7439876 | 4 | 80801878 | A | G | 0.571 | 0.012 | 0.002 | 2.30001e-07 | 451486 | 26.733 |
| Cheese intake | rs7515509 | 1 | 77949123 | A | G | 0.392 | -0.012 | 0.002 | 6.90001e-08 | 451486 | 29.104 |
| Cheese intake | rs75499503 | 6 | 26145217 | T | C | 0.22 | 0.014 | 0.003 | 2.30001e-07 | 451486 | 26.723 |
| Cheese intake | rs7607369 | 2 | 219279097 | G | A | 0.567 | 0.012 | 0.002 | 1.7e-07 | 451486 | 27.34 |
| Cheese intake | rs7730004 | 5 | 43191033 | T | C | 0.678 | -0.011 | 0.002 | 2.19999e-06 | 451486 | 22.382 |
| Cheese intake | rs77742462 | 3 | 107637010 | G | A | 0.021 | -0.047 | 0.008 | 9.80009e-09 | 451486 | 32.879 |
| Cheese intake | rs77837876 | 20 | 18958756 | C | T | 0.02 | 0.039 | 0.008 | 2.59998e-06 | 451486 | 22.081 |
| Cheese intake | rs78370515 | 6 | 153498987 | T | C | 0.05 | -0.028 | 0.005 | 1.2e-07 | 451486 | 27.98 |
| Cheese intake | rs78876700 | 1 | 4670487 | A | G | 0.137 | 0.018 | 0.003 | 3.40001e-08 | 451486 | 30.467 |
| Cheese intake | rs78898535 | 10 | 73953646 | A | G | 0.038 | 0.028 | 0.006 | 2.99999e-06 | 451486 | 21.803 |
| Cheese intake | rs79147413 | 3 | 141209594 | G | A | 0.067 | -0.022 | 0.004 | 6.1e-07 | 451486 | 24.874 |
| Cheese intake | rs7936836 | 11 | 43633645 | A | C | 0.418 | 0.016 | 0.002 | 2.60016e-12 | 451486 | 48.962 |
| Cheese intake | rs79398870 | 6 | 121712292 | C | T | 0.091 | -0.019 | 0.004 | 1.5e-06 | 451486 | 23.172 |
| Cheese intake | rs8059277 | 16 | 8270531 | C | T | 0.533 | 0.012 | 0.002 | 1.7e-07 | 451486 | 27.36 |
| Cheese intake | rs9377684 | 6 | 105445364 | G | A | 0.196 | 0.014 | 0.003 | 1.2e-06 | 451486 | 23.599 |
| Cheese intake | rs9504123 | 6 | 4478474 | C | A | 0.275 | 0.014 | 0.003 | 1.5e-08 | 451486 | 32.03 |
| Cheese intake | rs9735764 | 11 | 131841878 | G | A | 0.203 | -0.013 | 0.003 | 2.30001e-06 | 451486 | 22.289 |
| Cheese intake | rs975303 | 6 | 19028788 | G | A | 0.181 | 0.021 | 0.003 | 2.49977e-13 | 451486 | 53.564 |
| Cheese intake | rs992903 | 2 | 146849925 | T | C | 0.631 | -0.011 | 0.002 | 4.09996e-06 | 451486 | 21.222 |
| Cheese intake | rs9996848 | 4 | 182214915 | A | C | 0.116 | 0.017 | 0.004 | 1.79999e-06 | 451486 | 22.762 |
| Cereal intake | rs10057775 | 5 | 87469663 | C | T | 0.894 | 0.02 | 0.003 | 4.49987e-12 | 441640 | 47.907 |
| Cereal intake | rs10122671 | 9 | 9840211 | T | C | 0.031 | 0.024 | 0.005 | 4.30002e-06 | 441640 | 21.147 |
| Cereal intake | rs10124390 | 9 | 86549939 | A | C | 0.506 | 0.008 | 0.002 | 3.79997e-06 | 441640 | 21.358 |
| Cereal intake | rs10190125 | 2 | 2294426 | A | G | 0.35 | 0.009 | 0.002 | 6.69993e-07 | 441640 | 24.713 |
| Cereal intake | rs10260232 | 7 | 78120735 | T | C | 0.504 | -0.009 | 0.002 | 2.1e-06 | 441640 | 22.538 |
| Cereal intake | rs10737693 | 1 | 197599030 | C | T | 0.781 | 0.011 | 0.002 | 4.49997e-07 | 441640 | 25.451 |
| Cereal intake | rs10799311 | 1 | 225650626 | A | G | 0.709 | 0.01 | 0.002 | 5.39995e-07 | 441640 | 25.117 |
| Cereal intake | rs10809287 | 9 | 11018582 | T | C | 0.644 | 0.01 | 0.002 | 1.09999e-07 | 441640 | 28.113 |
| Cereal intake | rs10812904 | 9 | 28974908 | G | A | 0.711 | 0.01 | 0.002 | 6.1e-07 | 441640 | 24.88 |
| Cereal intake | rs10857964 | 1 | 113097633 | C | T | 0.205 | 0.014 | 0.002 | 1.7e-10 | 441640 | 40.801 |
| Cereal intake | rs10879791 | 12 | 74886325 | T | C | 0.333 | 0.009 | 0.002 | 4e-06 | 441640 | 21.267 |
| Cereal intake | rs10931025 | 2 | 183470617 | T | C | 0.439 | -0.008 | 0.002 | 3.89996e-06 | 441640 | 21.302 |
| Cereal intake | rs11038810 | 11 | 46210773 | G | A | 0.644 | 0.011 | 0.002 | 2.30001e-09 | 441640 | 35.709 |
| Cereal intake | rs11097340 | 4 | 93465297 | T | C | 0.4 | -0.012 | 0.002 | 2.1e-10 | 441640 | 40.379 |
| Cereal intake | rs11113399 | 12 | 107966276 | C | T | 0.293 | 0.01 | 0.002 | 2.5e-07 | 441640 | 26.592 |
| Cereal intake | rs11119497 | 1 | 210606369 | A | G | 0.455 | 0.01 | 0.002 | 5.39995e-08 | 441640 | 29.569 |
| Cereal intake | rs11258417 | 10 | 13533053 | T | C | 0.403 | 0.009 | 0.002 | 2.1e-06 | 441640 | 22.465 |
| Cereal intake | rs1158065 | 5 | 166674933 | A | C | 0.172 | -0.012 | 0.002 | 4.60002e-07 | 441640 | 25.411 |
| Cereal intake | rs11643192 | 16 | 72214276 | A | C | 0.39 | -0.009 | 0.002 | 6.19998e-07 | 441640 | 24.834 |
| Cereal intake | rs11670024 | 19 | 18818335 | G | A | 0.116 | 0.016 | 0.003 | 1.09999e-08 | 441640 | 32.712 |
| Cereal intake | rs11678980 | 2 | 162101261 | A | G | 0.462 | -0.009 | 0.002 | 1.40001e-06 | 441640 | 23.305 |
| Cereal intake | rs117477397 | 22 | 20209974 | T | G | 0.045 | 0.022 | 0.004 | 2.5e-07 | 441640 | 26.567 |
| Cereal intake | rs11764446 | 7 | 102102343 | C | T | 0.919 | 0.016 | 0.003 | 1.40001e-06 | 441640 | 23.234 |
| Cereal intake | rs117753937 | 22 | 32595919 | T | C | 0.084 | 0.015 | 0.003 | 2.80001e-06 | 441640 | 21.958 |
| Cereal intake | rs11780710 | 8 | 31169385 | T | G | 0.511 | -0.009 | 0.002 | 2e-06 | 441640 | 22.604 |
| Cereal intake | rs11922147 | 3 | 108311784 | C | T | 0.049 | 0.021 | 0.004 | 9.59997e-07 | 441640 | 24.017 |
| Cereal intake | rs11940694 | 4 | 39414993 | G | A | 0.604 | -0.013 | 0.002 | 5.00035e-12 | 441640 | 47.672 |
| Cereal intake | rs12354267 | 1 | 44248272 | C | T | 0.309 | 0.012 | 0.002 | 1.7e-09 | 441640 | 36.314 |
| Cereal intake | rs12414768 | 10 | 2291059 | G | A | 0.084 | 0.016 | 0.003 | 5.49997e-07 | 441640 | 25.074 |
| Cereal intake | rs12887676 | 14 | 98818823 | C | T | 0.518 | -0.008 | 0.002 | 4e-06 | 441640 | 21.26 |
| Cereal intake | rs12892571 | 14 | 101005236 | G | A | 0.39 | -0.009 | 0.002 | 4.30002e-07 | 441640 | 25.54 |
| Cereal intake | rs13066686 | 3 | 94075026 | A | C | 0.406 | 0.009 | 0.002 | 2.59998e-06 | 441640 | 22.117 |
| Cereal intake | rs13234131 | 7 | 73025975 | G | A | 0.128 | 0.017 | 0.003 | 1.6e-10 | 441640 | 40.894 |
| Cereal intake | rs138306181 | 5 | 12715834 | T | C | 0.011 | 0.043 | 0.009 | 2.99999e-06 | 441640 | 21.79 |
| Cereal intake | rs1397440 | 4 | 140892872 | C | T | 0.675 | -0.009 | 0.002 | 1.09999e-06 | 441640 | 23.785 |
| Cereal intake | rs141407081 | 10 | 114890672 | T | C | 0.019 | 0.033 | 0.007 | 1.2e-06 | 441640 | 23.503 |
| Cereal intake | rs141612194 | 2 | 226966172 | G | A | 0.021 | -0.031 | 0.006 | 1.2e-06 | 441640 | 23.541 |
| Cereal intake | rs143747365 | 15 | 37450983 | G | A | 0.01 | -0.046 | 0.01 | 3.29997e-06 | 441640 | 21.651 |
| Cereal intake | rs143747365 | 15 | 37450983 | G | A | 0.01 | -0.046 | 0.01 | 3.29997e-06 | 441640 | 21.651 |
| Cereal intake | rs1455349 | 2 | 199497285 | G | A | 0.463 | -0.008 | 0.002 | 2.59998e-06 | 441640 | 22.092 |
| Cereal intake | rs149604870 | 10 | 10203159 | T | C | 0.088 | -0.015 | 0.003 | 4.39997e-06 | 441640 | 21.096 |
| Cereal intake | rs1500962 | 1 | 66530055 | A | C | 0.081 | 0.018 | 0.003 | 6.80002e-08 | 441640 | 29.122 |
| Cereal intake | rs1604918 | 8 | 91881334 | G | T | 0.296 | -0.009 | 0.002 | 3.09999e-06 | 441640 | 21.776 |
| Cereal intake | rs17115145 | 14 | 30122409 | T | C | 0.401 | -0.009 | 0.002 | 1.89998e-06 | 441640 | 22.66 |
| Cereal intake | rs17599026 | 5 | 137763798 | T | C | 0.098 | 0.014 | 0.003 | 4.09996e-06 | 441640 | 21.235 |
| Cereal intake | rs17732534 | 7 | 91479659 | G | T | 0.073 | -0.016 | 0.004 | 4.70002e-06 | 441640 | 20.94 |
| Cereal intake | rs184643 | 2 | 45189441 | A | G | 0.567 | -0.012 | 0.002 | 1.59993e-11 | 441640 | 45.437 |
| Cereal intake | rs1853931 | 6 | 125170409 | A | G | 0.531 | -0.011 | 0.002 | 3.79997e-10 | 441640 | 39.223 |
| Cereal intake | rs186852039 | 9 | 35742002 | T | C | 0.032 | 0.025 | 0.005 | 5.19996e-07 | 441640 | 25.195 |
| Cereal intake | rs1966434 | 16 | 54030749 | A | G | 0.388 | 0.01 | 0.002 | 1.2e-07 | 441640 | 28.002 |
| Cereal intake | rs2005143 | 9 | 110038538 | C | T | 0.288 | 0.009 | 0.002 | 2.59998e-06 | 441640 | 22.081 |
[truncated: 168,296 more chars]
